# Multi-ancestry genetic architecture of heart failure subtypes

**DOI:** 10.1038/s41467-026-76438-y

**Published:** 2026-08-13

**Authors:** Chang Liu, Qin Hui, Gregorio V. Linchangco, Quinn S. Wells, Eric Farber-Eger, Danielle Rasooly, J. Michael Gaziano, Peter W. F. Wilson, Arshed A. Quyyumi, Viola Vaccarino, Yi-Juan Hu, David Benkeser, Kaoru Ito, Nobuyuki Enzan, VA Million Veteran Program, Qin Hui, Danielle Rasooly, J. Michael Gaziano, Lawrence S. Phillips, Jacob Joseph, Yan V. Sun, Lawrence S. Phillips, Jacob Joseph, Yan V. Sun

**Affiliations:** 1 Department of Epidemiology, Emory University Rollins School of Public Health, Atlanta, GA USA; 2 Atlanta VA Healthcare System, Decatur, GA USA; 3 Division of Cardiovascular Medicine, Department of Medicine, Vanderbilt University School of Medicine, Nashville, TN USA; 4 Division of Aging, Department of Medicine, Brigham and Women’s Hospital, Harvard Medical School, Boston, MA USA; 5 Massachusetts Veterans Epidemiology Research and Information Center (MAVERIC), VA Boston Healthcare System, Boston, MA USA; 6 Emory University School of Medicine, Atlanta, GA USA; 7 Department of Biostatistics and Bioinformatics, Emory University Rollins School of Public Health, Atlanta, GA USA; 8 Laboratory for Cardiovascular Genomics and Informatics, RIKEN Center for Integrative Medical Sciences, Yokohama, Japan; 9 Department of Advanced Biomedical Data Science, Graduate School of Medicine, Chiba University, Chiba, Japan; 10 Cardiovascular Research Center, Massachusetts General Hospital, Boston, MA USA; 11 Cardiovascular Disease Initiative, The Broad Institute of MIT and Harvard, Cambridge, MA USA; 12 VA Providence Healthcare System, Providence, RI USA; 13 The Warren Alpert Medical School of Brown University, Providence, RI USA

**Keywords:** Cardiovascular genetics, Personalized medicine

## Abstract

Heart failure (HF) affects 6.7 million people in the US and includes two major subtypes, HF with reduced ejection fraction (HFrEF) and HF with preserved ejection fraction (HFpEF), with distinct genetic architectures. We meta-analyze genome-wide association studies (GWAS) of 38,781 HFrEF cases, 38,163 HFpEF cases, and 526,135 controls across European, African, Hispanic, and Asian ancestries using the Million Veteran Program and Vanderbilt University DNA Databank (BioVU). We identify 46 genome-wide significant loci for HFrEF (9 novel) and 3 loci for HFpEF (1 novel). Four HFrEF loci are detected in African ancestry participants near *CD36, SPI1, TRIM48*, and *SPNS3*, with lead SNPs showing low risk-allele frequencies in European populations. In the all-cause HF meta-analysis (200,070 cases, 2,076,466 controls), we identify 136 loci (12 novel). Gene-based tests, tissue enrichment, transcriptome-wide association, and fine-mapping implicate vascular, metabolic, and TGF-β/Smad signaling pathways and nominate candidate causal genes, clarifying shared and subtype-specific risk across ancestries.

## Abstract (web summary)

This study maps the genetic basis of major heart failure subtypes across diverse populations, identifying shared and subtype-specific risk variants that may inform biology, risk prediction and future therapies.

## Introduction

Heart failure (HF) affects an estimated 6.7 million people in the US^1,2^, contributing to high morbidity, mortality, and healthcare costs^3^. The burden of HF in the US has been growing continuously, with a projection of over 8 million people affected and an estimated annual cost of 53 billion US dollars (USD) in healthcare expenditure by 2030^3,4^. The clinical subtypes of HF are defined by left ventricular ejection fraction (LVEF). HF with reduced ejection fraction (HFrEF) is defined as LVEF ≤ 40%, and HF with preserved ejection fraction (HFpEF) is defined as LVEF ≥ 50%^5^, with different risk profiles and clinical manifestations for each subtype. HFpEF (i.e., diastolic HF) is featured by impaired left ventricular filling, while HFrEF (i.e., systolic HF) is most commonly caused by heart disease related to structural remodeling and contractile dysfunction^6,7^.

Genetic factors contribute to HF risk, supported by an estimated heritability of ~26% for all-cause HF^8^, 25% for HFrEF and 22% for HFpEF^9^. Recent large genome-wide association study (GWAS) of all-cause HF among >90,000 cases and >1 million controls of European ancestry identified 39 genomic loci associated with all-cause HF^10,11^, and a multi-ancestry GWAS including >200,000 cases and >2 million controls identified 176 loci^12–14^. The genetic architecture of HFpEF and HFrEF can be different, given their distinct pathophysiology and risk profile^9^. The recent GWAS of HF subtypes among Europeans, which included 19,495 HFrEF cases, 19,589 HFpEF cases, and 258,943 controls, identified 13 loci associated with HFrEF but only one associated with HFpEF, despite a comparable sample size between the two subtypes^9^. These findings shed light on the differences in the genetic architecture of HFrEF and HFpEF and emphasize the need for expanding discoveries among diverse populations to better understand the genetic basis of the HF subtypes, especially HFpEF, which remains poorly understood and more common among African populations^15^. In this study of a diverse population, we aim to identify ancestry-specific and cross-ancestry genetic loci associated with HFrEF and HFpEF, and to better understand the biological mechanisms of each subtype.

In this work, we perform a multi-ancestry GWAS meta-analysis of HFrEF and HFpEF across European, African, Hispanic, and Asian ancestry participants from the Million Veteran Program and BioVU. We identify 46 genome-wide significant loci for HFrEF and three loci for HFpEF. We further identify 136 loci for all-cause HF and use gene-based testing, tissue-enrichment analysis, transcriptome-wide association studies, and fine-mapping to nominate candidate causal genes and biological pathways. Together, these findings clarify shared and subtype-specific genetic risk for HF across ancestries and highlight biological mechanisms that may contribute to differences between HFrEF and HFpEF.

## Results

### Identification of genome-wide significant (GWS) loci

**Fig. 1:**
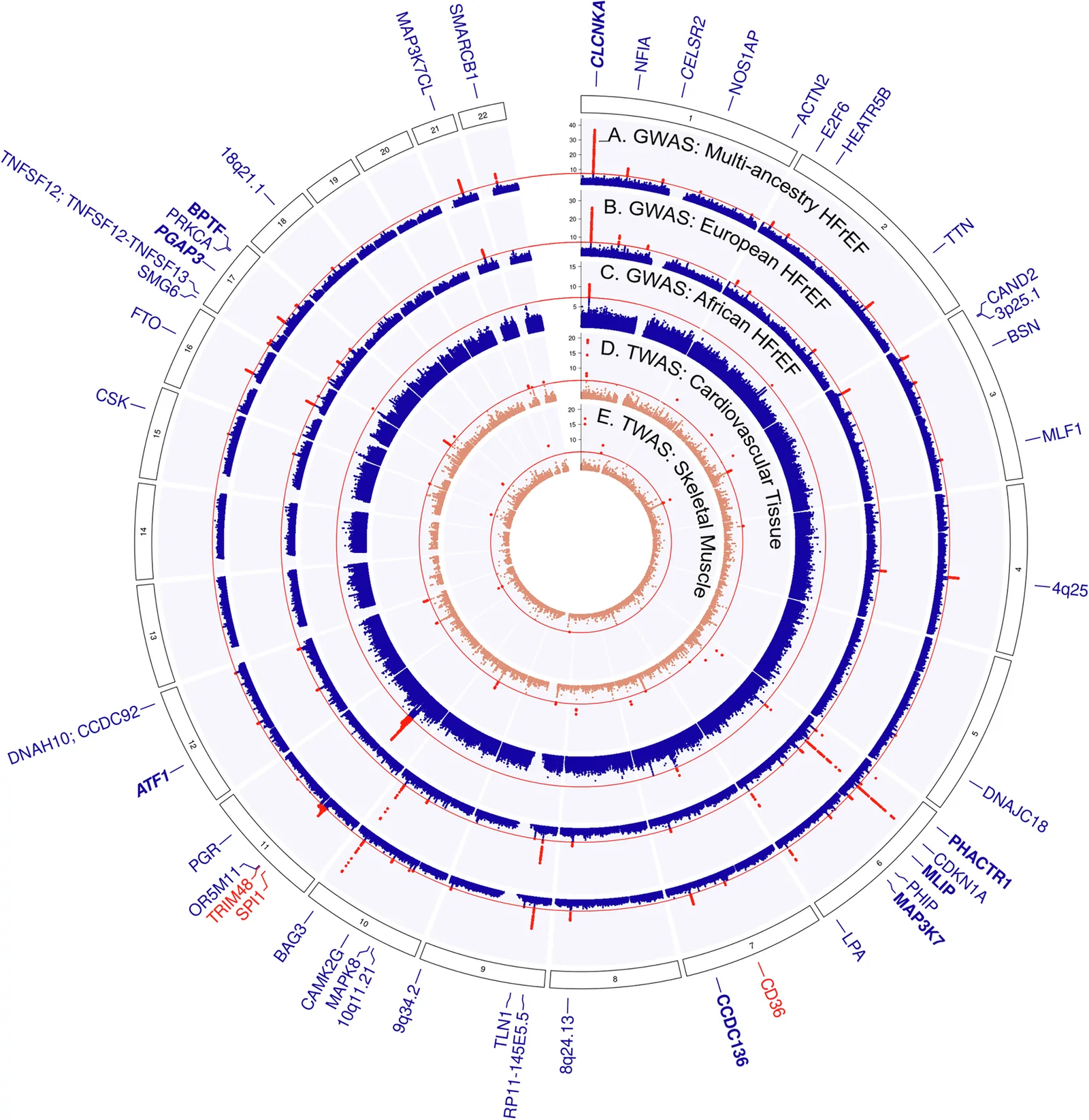
Manhattan Plots for the meta-analysis of GWAS on HFrEF and TWAS. Track A: Manhattan Plot of the multi-ancestry GWAS meta-analysis on HFrEF. Track B: Manhattan Plot of the European-ancestry GWAS meta-analysis on HFrEF. Track C: Manhattan Plot of the African-ancestry GWAS meta-analysis on HFrEF. Track D: Manhattan Plot of the TWAS on HFrEF in cardiovascular tissue types. Track E: Manhattan Plot of the TWAS on HFrEF in skeletal muscle tissue type. The genome-wide significant loci of HFrEF are labeled, with red color indicating African-specific genome-wide significant loci. Bold font indicates significant enrichment in cardiovascular tissue types. Italic font indicates significant enrichment in skeletal muscle tissue type. Bold italic font indicates significant enrichment in both cardiovascular and skeletal muscle tissue types.

To identify genetic loci associated with HF and to distinguish signals shared across ancestries from those that may be ancestry-specific, we conducted multi-ancestry and ancestry-specific GWAS meta-analyses of HFrEF, HFpEF, and all-cause HF. Genome-wide significance was defined as *p* < 5 × 10^−8^. The overall sample size and workflow for GWAS meta-analyses are shown in Supplementary Data 1A, B, and Supplementary Fig. 1. In the multi-ancestry meta-analysis of HFrEF, 46 GWS loci were identified, including nine loci, *NOS1AP, TTN, MAP3K7, CD36, TLN1, 10q11.21, MAPK8, CSK*, and *18q21.1*, that were not reported in previous HF GWAS^9–14^, Table 1, Supplementary Data 2, and Fig. 1. For HFpEF, three GWS loci were identified, including one locus *21q21.1* that was not reported in previous HF GWAS^9–14^, Table 1, Supplementary Data 2, and Supplementary Fig. 2. For all-cause HF, we identified 136 GWS loci, with 12 loci not previously reported^9–14^, Supplementary Data 3 and Supplementary Fig. 3. The Venn diagram of the GWS loci across all-cause HF and subtypes are shown in Supplementary Fig. 4.

**Table 1:** Genome-wide significant loci for the multi-ancestry meta-analysis of GWAS on HFrEF and HFpEF

rsID	Chr.	Position (hg19)	Gene	Novel^a^	EA	NEA	Meta-analysis		Biobank Japan validation		
							OR (95% CI)	*P*	EAF	OR (95% CI)	*P*
The 46 genome-wide significant loci for HFrEF											
rs945425	1	16348412	*CLCNKA*	No	C	T	1.11 (1.1, 1.13)	1.37E-37	–	–	–
rs1997998	1	61883027	*NFIA*	No	T	G	1.06 (1.05, 1.08)	3.12E-13	0.03	1.06 (0.91, 1.23)	4.71E-01
rs12740374	1	109817590	*CELSR2*	No	G	T	1.06 (1.04, 1.08)	6.41E-10	–	–	–
rs10429888	1	162024987	*NOS1AP*	Yes	G	A	1.08 (1.05, 1.11)	4.57E-09	0.62	1.05 (1, 1.1)	4.74E-02
rs524517	1	236839990	*ACTN2*	No	T	A	1.07 (1.05, 1.1)	1.83E-08	0.81	1.1 (1.04, 1.17)	8.55E-04
rs34514099	2	11571966	*E2F6*	No	T	C	1.06 (1.04, 1.08)	2.74E-11	0.59	1.11 (1.06, 1.16)	1.70E-05
rs62133082	2	37233937	*HEATR5B*	No	T	C	1.06 (1.04, 1.09)	1.30E-09	0.43	1.08 (1.03, 1.13)	1.56E-03
rs17076	2	179595117	*TTN*	Yes	C	G	1.08 (1.06, 1.1)	4.94E-14	0.34	1.17 (1.12, 1.23)	3.41E-11
rs4642101	3	12842223	*CAND2*	No	G	T	1.05 (1.03, 1.07)	4.18E-09	0.3	1.15 (1.1, 1.21)	5.41E-09
rs6807275	3	14274451	*3p25.1*	No	G	A	1.07 (1.05, 1.09)	4.72E-13	0.3	1.14 (1.09, 1.2)	5.98E-08
rs34989545	3	49622632	*BSN*	No	G	GT	1.06 (1.04, 1.08)	1.33E-09	–	–	–
rs2082838	3	158305894	*MLF1*	No	A	G	1.06 (1.04, 1.08)	4.00E-09	0.06	1.01 (0.92, 1.11)	8.30E-01
rs17042098	4	111664158	*4q25*	No	A	G	1.1 (1.07, 1.12)	4.34E-15	0.31	1.09 (1.04, 1.14)	7.96E-04
rs11242464	5	138746334	*DNAJC18*	No	C	G	1.05 (1.03, 1.07)	2.32E-08	0.41	1.13 (1.08, 1.18)	3.27E-07
rs9349379	6	12903957	*PHACTR1*	No	G	A	1.06 (1.05, 1.08)	4.63E-14	0.66	1.01 (0.97, 1.07)	5.68E-01
rs146170154	6	36646768	*CDKN1A*	No	C	CTA	1.14 (1.12, 1.16)	5.14E-41	0.89	1.24 (1.15, 1.35)	4.05E-08
rs1407229	6	53971921	*MLIP*	No	T	C	1.06 (1.04, 1.07)	2.97E-11	0.34	1.08 (1.03, 1.13)	2.54E-03
rs66577603	6	79749166	*PHIP*	No	C	CTT	1.04 (1.03, 1.06)	4.77E-08	0.63	1.03 (0.98, 1.08)	2.60E-01
rs282064	6	91303512	*MAP3K7*	Yes	T	G	1.05 (1.04, 1.07)	2.44E-10	0.33	1.06 (1.01, 1.11)	1.34E-02
rs10455872	6	161010118	*LPA*	No	G	A	1.15 (1.12, 1.19)	2.29E-18	–	–	–
rs3211938	7	80300449	*CD36*	Yes	G	T	1.2 (1.14, 1.28)	3.92E-10	–	–	–
rs2270590	7	128455916	*CCDC136*	No	G	A	1.08 (1.06, 1.1)	5.28E-15	0.44	1.06 (1.02, 1.11)	8.85E-03
rs7461129	8	125861374	*8q24.13*	No	C	T	1.07 (1.05, 1.09)	2.03E-14	0.5	1.14 (1.09, 1.2)	7.86E-09
rs4977575	9	22124744	*RP11-145E5.5*	No	G	C	1.08 (1.07, 1.1)	5.37E-22	0.66	1.07 (1.02, 1.12)	6.56E-03
rs10814273	9	35723234	*TLN1*	Yes	T	G	1.05 (1.03, 1.07)	3.12E-08	0.05	1.12 (1.01, 1.23)	2.95E-02
rs600038	9	136151806	*9q34.2*	No	C	T	1.06 (1.04, 1.08)	2.06E-09	0.28	1.05 (1, 1.11)	4.44E-02
rs485838	10	44750414	*10q11.21*	Yes	G	T	1.06 (1.04, 1.08)	1.94E-08	0.67	1.02 (0.97, 1.07)	3.59E-01
rs148307915	10	49528021	*MAPK8*	Yes	A	G	1.08 (1.05, 1.1)	2.29E-09	–	–	–
rs2242254	10	75585307	*CAMK2G*	No	G	A	1.07 (1.05, 1.1)	3.48E-10	0.79	1.04 (0.98, 1.1)	2.01E-01
rs375034445	10	121424815	*BAG3*	No	A	AT	1.14 (1.12, 1.16)	5.09E-34	–	–	–
rs113123267	11	47379410	*SPI1*	No	T	C	1.35 (1.26, 1.46)	2.74E-15	–	–	–
rs111786504	11	55047396	*TRIM48*	No	G	A	1.61 (1.39, 1.86)	1.57E-10	–	–	–
rs60504091	11	56329980	*OR5M11*	No	A	G	1.26 (1.18, 1.36)	3.56E-10	–	–	–
rs658286	11	100942792	*PGR*	No	T	G	1.05 (1.03, 1.07)	1.01E-08	–	–	–
rs11169562	12	51187290	*ATF1*	No	T	C	1.05 (1.03, 1.07)	1.80E-09	0.36	1.02 (0.98, 1.07)	3.11E-01
rs4575361	12	124410529	*DNAH10; CCDC92*	No	A	T	1.05 (1.03, 1.07)	9.36E-09	0.84	1.03 (0.97, 1.1)	3.35E-01
rs62006566	15	75086534	*CSK*	Yes	C	T	1.05 (1.03, 1.07)	2.69E-08	–	–	–
rs7188250	16	53834607	*FTO*	No	C	T	1.06 (1.05, 1.08)	1.55E-13	0.21	1.05 (0.99, 1.11)	1.10E-01
rs2224770	17	2205923	*SMG6*	No	T	C	1.05 (1.03, 1.07)	1.76E-09	0.24	1.08 (1.03, 1.14)	2.92E-03
rs9907657	17	7460957	*TNFSF12; TNFSF12-TNFSF13*	No	T	G	1.06 (1.04, 1.09)	6.09E-09	0.21	1 (0.95, 1.06)	9.36E-01
rs2517953	17	37841211	*PGAP3*	No	G	C	1.06 (1.05, 1.08)	5.84E-14	0.5	1.02 (0.98, 1.07)	3.49E-01
rs9912468	17	64318357	*PRKCA*	No	G	C	1.05 (1.03, 1.06)	2.49E-09	0.41	1.06 (1.01, 1.11)	1.03E-02
rs145606454	17	65893289	*BPTF*	No	A	AC	1.06 (1.04, 1.08)	3.97E-09	0.68	1.02 (0.97, 1.07)	5.58E-01
rs7229491	18	46516424	*18q21.1*	Yes	C	G	1.05 (1.03, 1.07)	4.82E-09	0.46	1.01 (0.97, 1.06)	6.38E-01
rs56968346	21	30540165	*MAP3K7CL*	No	T	C	1.09 (1.07, 1.11)	4.17E-17	0.1	1.16 (1.08, 1.25)	5.28E-05
rs9608201	22	24166788	*SMARCB1*	No	A	G	1.07 (1.05, 1.09)	3.24E-14	0.61	1.17 (1.11, 1.22)	1.19E-10
The 3 genome-wide significant loci for HFpEF											
rs62048402	16	53803223	*FTO*	No	A	G	1.06 (1.05, 1.08)	1.10E-13	0.2	1.06 (1.02, 1.11)	6.34E-03
rs75716431	17	66382647	*ARSG*	No	T	C	1.12 (1.08, 1.17)	4.27E-08	0.12	1.02 (0.96, 1.07)	5.39E-01
rs113752382	21	18617840	*21q21.1*	Yes	T	C	1.12 (1.08, 1.17)	3.39E-08	–	–	–

Association testing was performed under an additive genetic model using logistic regression, followed by random-effects meta-analysis using GWAMA. All GWAS association *P* values were two-sided. Genome-wide significance was defined as *P* < 5 × 10^−8^. No additional adjustment for multiple comparisons was applied to comparisons restricted to the identified genome-wide significant loci. For results externally evaluated in Biobank Japan, replication was defined as a two-sided *P* < 0.05 with a concordant direction of effect. No additional multiple-testing correction was applied to the replication analyses.

*Chr.* chromosome, *EA* effect allele, *NEA* non-effect allele, *EAF* effect allele frequency, *OR* odds ratio, *CI* confidence interval.

^a^A total of 9 novel HFrEF loci and 1 novel HFpEF locus as compared to previous all-cause HF GWAS (PMID: 31919418, 36376295, 36517512, 37429843, 37503172, and 40038546), defined as non-overlapping physical distance of >500 kb on both sides of the variant.

To evaluate the transferability of the identified associations to an independent population, we examined the lead variants in BioBank Japan using a prespecified nominal replication criterion of *p* < 0.05 with a concordant direction of effect. Out of the 46 GWS loci identified in the multi-ancestry meta-analysis of HFrEF, 34 loci were available in Biobank Japan; among these, 21 (62%) loci replicated at *P* < 0.05 with the same direction of genetic effect. For HFpEF, 1 of the 3 GWS loci was available in Biobank Japan and replicated at *P* < 0.05 with concordant effect direction. For all-cause HF, out of the 136 GWS loci, 106 loci were available in Biobank Japan, of which 46 (43%) replicated at *P* < 0.05 with concordant effect direction, Table 1 and Supplementary Data 2, 3. Collectively, the ancestry- and study-specific internal results, together with the substantial proportion of loci that replicate in an independent East Asian population with consistent effect directions, support the robustness and generalizability of our findings.

We next performed ancestry-specific meta-analyses to identify loci that might be obscured in the combined analysis because of ancestry differences in allele frequency, LD structure, or genetic effect. In the ancestry-specific meta-analyses, for European ancestry, we identified 29 GWS HFrEF loci and 1 GWS HFpEF locus, *FTO*, Supplementary Data 4. For African ancestry, we identified 7 GWS HFrEF loci and 2 GWS HFpEF loci, Supplementary Data 5. Additionally, the all-cause HF GWAS meta-analyses revealed 102 GWS loci for European ancestry and 3 GWS loci for African ancestry, Supplementary Data 6 and 7.

**Fig. 2:**
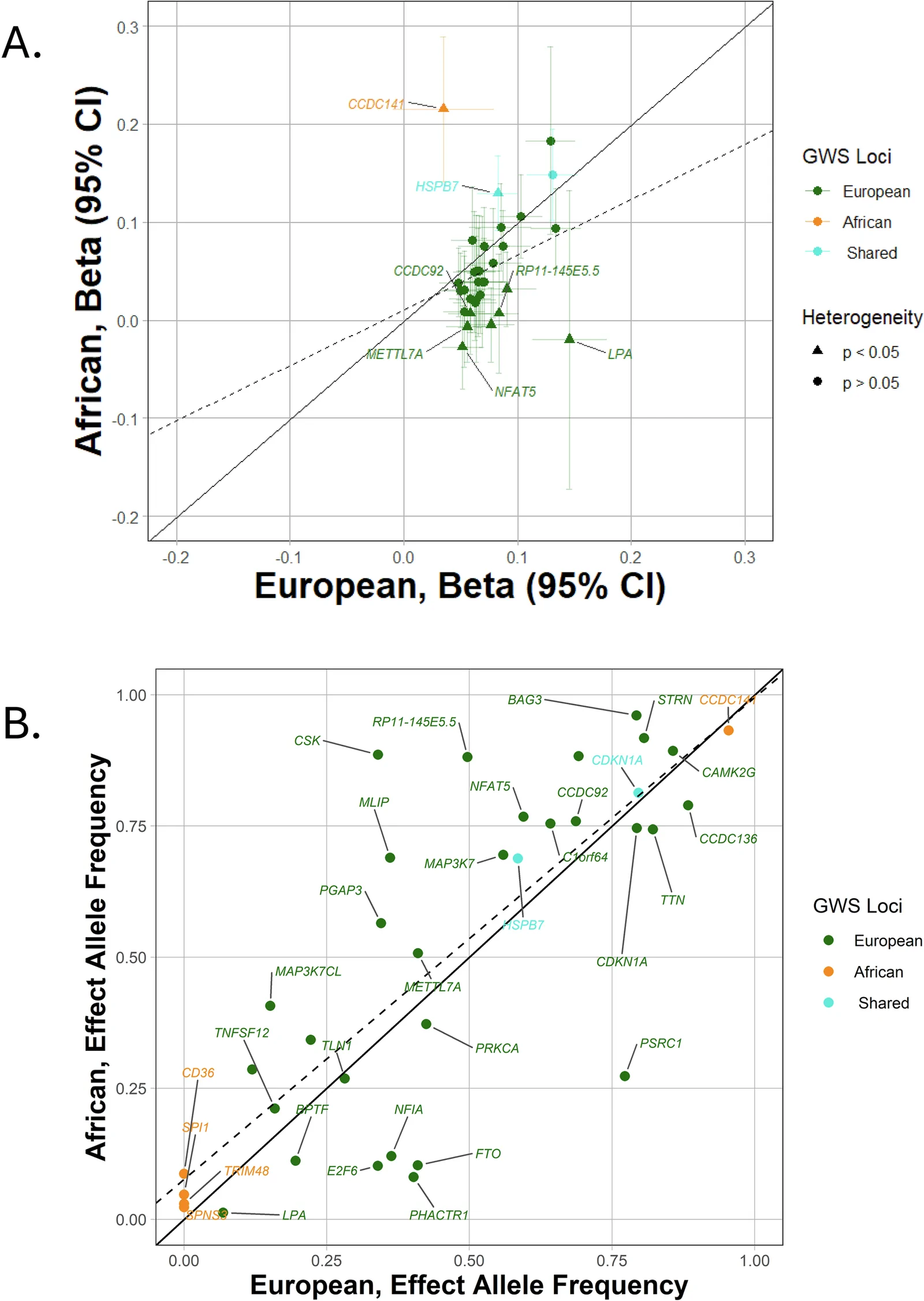
Scatter plot for comparison of the estimates for the genome-wide significant loci for the meta-analysis of GWAS on HFrEF among European ancestry and African ancestry. **A** Comparison of effect sizes. Pearson correlation coefficient = 0.27 (95% CI −0.09, 0.57). Horizontal and vertical error bars indicate 95% confidence intervals. European-ancestry analyses included 29,062 HFrEF cases and 380,050 controls; African-ancestry analyses included 7637 HFrEF cases and 96,026 controls. Loci with heterogeneity (Cochran’s Q *p* < 0.05) are labeled. **B** Comparison of the effect allele frequency.

**Fig. 3:**
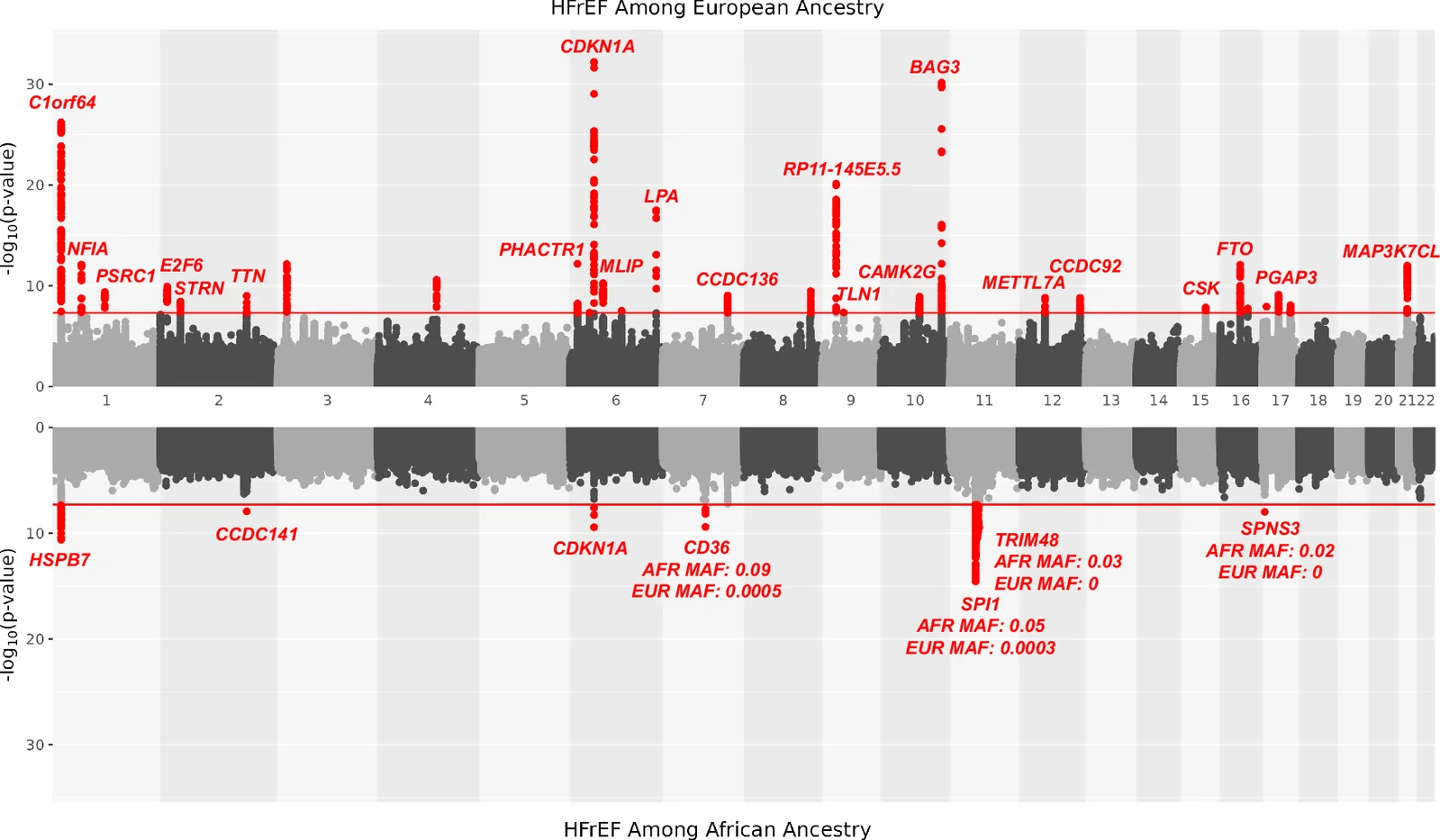
Miami Plot for comparison of the genome-wide significant loci for the meta-analysis of GWAS on HFrEF among European and African ancestry. Association testing was performed under an additive genetic model using logistic regression, followed by random-effects meta-analysis using GWAMA. All GWAS association *P* values were two-sided. Genome-wide significance was defined as *P* < 5 × 10^−8^.

Comparing the estimates for the GWS loci across ancestral populations revealed mostly similar genetic effects, with some ancestry-specific associations. Of the 29 HFrEF loci in the European ancestry, eight loci showed small or null effects in African ancestry, Table 2 and Fig. 2. Of the 7 HFrEF GWS loci in the African ancestry, two loci showed small or null effects in the European ancestry, and four loci (*CD36*, *SPI1*, *TRIM48*, and *SPNS3)* were rare in the European ancestry, with MAF ≤ 0.05%, Table 2 and Figs. 2, 3. The two lead SNPs in other loci, *CSK* (effect allele frequency 0.34 in Europeans vs. 0.89 in Africans) and *PSRC1* (0.77 in Europeans vs. 0.27 in Africans), demonstrated allele frequency differences of ≥50% between European and African populations. For HFpEF, the effect for the risk alleles at the *FTO* and *14q32.13* loci were heterogeneous across European and African ancestries, with statistical significance from the Cochran’s Q test *p* value of 0.015 and 3.2 × 10^−8^, respectively, Table 2.

**Table 2:** Comparison of the estimates for the genome-wide significant loci for the meta-analysis of GWAS on HFrEF and HFpEF across ancestries

rsID	Chr.	Position (hg19)	Gene	EA	NEA	European estimates			African estimates			Heterogeneity *P*^a^
						EAF	OR (95% CI)	*P*	EAF	OR (95% CI)	*P*	
The 29 HFrEF loci identified in individuals with European genetic ancestry												
rs34033692	1	16335302	*C1orf64*	C	CA	0.64	1.11 (1.09, 1.13)	6.13E-27	0.75	1.11 (1.07, 1.16)	8.75E-07	0.9170
rs1997998	1	61883027	*NFIA*	T	G	0.36	1.07 (1.05, 1.09)	8.00E-13	0.12	1.05 (0.99, 1.11)	0.0884	0.5864
rs599839	1	109822166	*PSRC1*	A	G	0.77	1.07 (1.05, 1.09)	4.17E-10	0.27	1.03 (0.99, 1.07)	0.2023	0.0767
rs34514099	2	11571966	*E2F6*	T	C	0.34	1.06 (1.04, 1.08)	1.22E-10	0.1	1.05 (0.99, 1.12)	0.1218	0.6849
rs3770770	2	37192866	*STRN*	C	T	0.81	1.07 (1.05, 1.1)	3.64E-09	0.92	1.04 (0.97, 1.11)	0.2482	0.4142
rs17076	2	179595117	*TTN*	C	G	0.82	1.07 (1.05, 1.1)	1.01E-09	0.74	1.08 (1.04, 1.12)	0.0002	0.8571
rs4685090	3	14280870	*3p25.1*	T	G	0.22	1.08 (1.06, 1.1)	7.18E-13	0.34	1 (0.96, 1.03)	0.8074	0.0002
rs10026140	4	111642851	*4q25*	G	T	0.12	1.09 (1.07, 1.12)	2.54E-11	0.29	1.03 (0.99, 1.07)	0.1069	0.0132
rs9349379	6	12903957	*PHACTR1*	G	A	0.4	1.07 (1.05, 1.08)	6.66E-13	0.08	1.02 (0.96, 1.09)	0.5194	0.2071
rs4151702	6	36645988	*CDKN1A*	G	C	0.79	1.14 (1.12, 1.17)	6.73E-33	0.75	1.1 (1.05, 1.14)	4.88E-06	0.0839
rs1407229	6	53971921	*MLIP*	T	C	0.36	1.06 (1.05, 1.09)	5.47E-11	0.69	1.02 (0.98, 1.06)	0.3671	0.0398
rs6454815	6	91305608	*MAP3K7*	G	A	0.56	1.05 (1.03, 1.07)	3.06E-08	0.7	1.03 (0.99, 1.07)	0.1202	0.3565
rs10455872	6	161010118	*LPA*	G	A	0.07	1.16 (1.12, 1.2)	3.17E-18	0.01	0.98 (0.84, 1.14)	0.7982	0.0370
rs56377531	7	128460745	*CCDC136*	C	T	0.88	1.09 (1.06, 1.12)	9.37E-10	0.79	1.1 (1.05, 1.15)	2.65E-05	0.7201
rs55679363	8	125867834	*8q24.13*	A	T	0.69	1.06 (1.04, 1.08)	3.44E-10	0.88	1.09 (1.03, 1.15)	0.0033	0.4671
rs4977575	9	22124744	*RP11-145E5.5*	G	C	0.5	1.09 (1.07, 1.11)	7.38E-21	0.88	1.01 (0.95, 1.07)	0.8300	0.0176
rs2737275	9	35726397	*TLN1*	G	C	0.28	1.06 (1.04, 1.08)	4.43E-08	0.27	1.03 (0.99, 1.07)	0.1278	0.3048
rs2242254	10	75585307	*CAMK2G*	G	A	0.86	1.08 (1.05, 1.11)	1.18E-09	0.89	1.06 (1, 1.12)	0.0399	0.5046
rs375034445	10	121424815	*BAG3*	A	AT	0.79	1.14 (1.11, 1.16)	6.92E-31	0.96	1.2 (1.09, 1.32)	0.0002	0.2810
–	12	51315031	*METTL7A*	C	T	0.41	1.06 (1.04, 1.08)	1.52E-09	0.51	0.99 (0.96, 1.03)	0.7156	0.0022
rs35825716	12	124427524	*CCDC92*	G	C	0.69	1.06 (1.04, 1.08)	1.58E-09	0.76	1.01 (0.97, 1.05)	0.7494	0.0261
rs1378942	15	75077367	*CSK*	C	A	0.34	1.05 (1.04, 1.07)	1.46E-08	0.89	1.01 (0.95, 1.07)	0.7615	0.1412
rs7188250	16	53834607	*FTO*	C	T	0.41	1.07 (1.05, 1.08)	8.22E-13	0.1	1.05 (0.99, 1.11)	0.0851	0.6428
rs11332955	16	69564990	*NFAT5*	GA	G	0.59	1.05 (1.03, 1.07)	1.78E-08	0.77	0.97 (0.93, 1.01)	0.1994	0.0007
rs9907657	17	7460957	*TNFSF12; TNFSF12-TNFSF13*	T	G	0.16	1.07 (1.05, 1.1)	1.19E-08	0.21	1.04 (0.99, 1.09)	0.0907	0.2175
rs732084	17	37834357	*PGAP3*	A	C	0.35	1.06 (1.04, 1.08)	7.64E-10	0.56	1.02 (0.99, 1.06)	0.2204	0.0664
rs9912468	17	64318357	*PRKCA*	G	C	0.42	1.05 (1.03, 1.07)	4.43E-08	0.37	1.04 (1, 1.08)	0.0305	0.6135
rs12601921	17	65825354	*BPTF*	T	C	0.2	1.07 (1.04, 1.09)	8.40E-09	0.11	1.04 (0.98, 1.1)	0.1700	0.3779
rs1999323	21	30534128	*MAP3K7CL*	T	C	0.15	1.09 (1.07, 1.12)	1.01E-12	0.41	1.08 (1.04, 1.12)	4.53E-05	0.5788
The 7 HFrEF loci identified in individuals with African genetic ancestry												
rs1572381	1	16344494	*HSPB7*	A	G	0.58	1.09 (1.07, 1.11)	9.71E-20	0.69	1.14 (1.1, 1.18)	2.42E-11	0.0291
rs7564036	2	179704973	*CCDC141*	G	A	0.95	1.04 (0.99, 1.08)	0.124313	0.93	1.24 (1.15, 1.34)	1.16E-08	4.31E-05
rs3176326	6	36647289	*CDKN1A*	G	A	0.8	1.14 (1.11, 1.17)	9.16E-30	0.81	1.16 (1.11, 1.22)	3.66E-10	0.4989
rs3211938	7	80300449	*CD36*	G	T	0.0005	–	–	0.09	1.2 (1.14, 1.28)	3.92E-10	–
rs113123267	11	47379410	*SPI1*	T	C	0.0003	–	–	0.05	1.35 (1.26, 1.46)	2.74E-15	–
rs111786504	11	55047396	*TRIM48*	G	A	0	–	–	0.03	1.61 (1.39, 1.86)	1.57E-10	–
rs75531264	17	4339916	*SPNS3*	G	C	0	–	–	0.02	1.41 (1.25, 1.58)	1.03E-08	–
The 1 HFpEF locus identified in individuals with European genetic ancestry												
rs1558902	16	53803574	*FTO*	A	T	0.4	1.06 (1.05, 1.08)	5.68E-12	0.1	1.08 (1.01, 1.15)	0.0152	0.6591
The 2 HFpEF loci identified in individuals with African genetic ancestry												
rs12586269	14	95115214	*14q32.13*	G	A	0.17	0.99 (0.97, 1.02)	0.5146	0.03	1.33 (1.2, 1.48)	3.27E-08	3.17E-08
rs75716431	17	66382647	*ARSG*	T	C	0.02	1.08 (1, 1.16)	0.0574	0.18	1.15 (1.09, 1.21)	4.16E-08	0.1415

Association testing was performed under an additive genetic model using logistic regression, followed by random-effects meta-analysis using GWAMA. All GWAS association *P* values were two-sided. Genome-wide significance was defined as *P* < 5 × 10^−8^. Heterogeneity was assessed using Cochran’s Q test. No additional adjustment for multiple comparisons was applied to comparisons restricted to the identified genome-wide significant loci. For results externally evaluated in Biobank Japan, replication was defined as a two-sided *P* < 0.05 with a concordant direction of effect. No additional multiple-testing correction was applied to the replication analyses.

*Chr.* chromosome, *EA* effect allele, *NEA* non-effect allele, *EAF* effect allele frequency, *OR* odds ratio, *CI* confidence interval.

^a^Heterogeneity *P* value tests the difference in effect sizes between European and African ancestries.

**Fig. 4:**
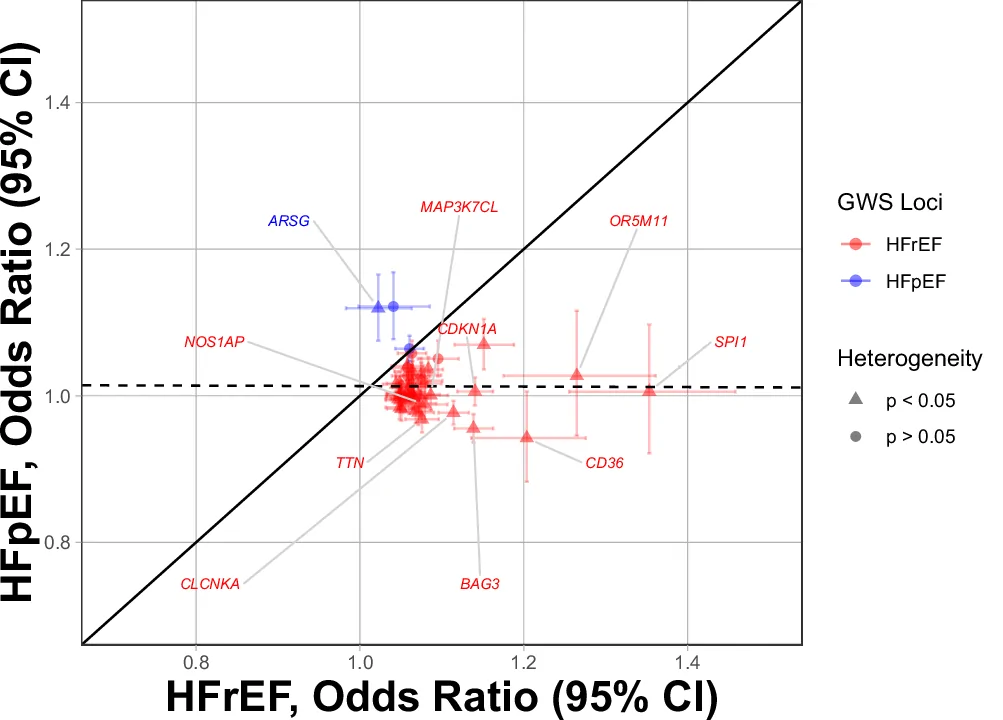
Scatter plot for comparison of the estimates for the genome-wide significant loci for the meta-analysis of GWAS on HFrEF and HFpEF among a multi-ancestry cohort. Pearson correlation coefficient = − 0.003 (95% CI −0.107, 0.100). Horizontal and vertical error bars indicate 95% confidence intervals. The HFrEF analysis included 38,781 cases and 526,135 controls; the HFpEF analysis included 38,163 cases and 526,135 controls. Loci with heterogeneity (Cochran’s Q *p* < 0.05) between HFrEF and HFpEF. Effect estimates are indicated by triangles. The top 10 loci with the largest differences in effect size are labeled.

To assess whether HFrEF and HFpEF share the same genetic determinants, we compared the effects of the lead loci across the two subtypes. Genetic loci are mostly specific to HFrEF and HFpEF. Of the 46 multi-ancestry HFrEF loci, 42 showed heterogeneous effects in HFpEF, Table 3 and Fig. 4. Among these, several HFrEF risk loci showed a protective effect in relation to HFpEF, including *CLCNKA*, *TTN, 3p25.1*, *8q24.13*, *BAG3, DNAH10/CCDC92*, Table 3. Of the three multi-ancestry HFpEF loci, *FTO* and *21q21.1* exhibited directionally concordant and risk-increasing effects on HFrEF, whereas ARSG showed no evidence of association with HFrEF, Table 3. Similar findings were identified in ancestry-specific meta-analyses, Supplementary Data 8 and Supplementary Figs. 5, 6.

**Table 3:** Comparison of the estimates for the genome-wide significant loci for the multi-ancestry meta-analysis of GWAS across HFrEF and HFpEF

rsID	Chr.	Position (hg19)	Gene	EA	NEA	HFrEF estimates			HFpEF estimates			Heterogeneity *P*^a^
						EAF	OR (95% CI)	*P*	EAF	OR (95% CI)	*P*	
The 46 multi-ancestry HFrEF loci												
rs945425	1	16348412	*CLCNKA*	C	T	0.68	1.11 (1.1, 1.13)	1.37E-37	0.68	0.98 (0.96, 0.99)	0.0054	1.10E-23
rs1997998	1	61883027	*NFIA*	T	G	0.31	1.06 (1.05, 1.08)	3.12E-13	0.3	1 (0.98, 1.01)	0.7617	2.19E-05
rs12740374	1	109817590	*CELSR2*	G	T	0.78	1.06 (1.04, 1.08)	6.41E-10	0.78	1.04 (1.02, 1.06)	4.38E-05	0.5167
rs10429888	1	162024987	*NOS1AP*	G	A	0.88	1.08 (1.05, 1.11)	4.57E-09	0.88	0.99 (0.96, 1.01)	0.3552	2.36E-05
rs524517	1	236839990	*ACTN2*	T	A	0.87	1.07 (1.05, 1.1)	1.83E-08	0.87	1 (0.98, 1.03)	0.8968	0.0008
rs34514099	2	11571966	*E2F6*	T	C	0.31	1.06 (1.04, 1.08)	2.74E-11	0.31	1 (0.99, 1.02)	0.5927	4.10E-06
rs62133082	2	37233937	*HEATR5B*	T	C	0.82	1.06 (1.04, 1.09)	1.30E-09	0.82	1.03 (1.01, 1.05)	0.0101	0.0200
rs17076	2	179595117	*TTN*	C	G	0.79	1.08 (1.06, 1.1)	4.94E-14	0.79	0.97 (0.95, 0.99)	0.0007	2.81E-13
rs4642101	3	12842223	*CAND2*	G	T	0.63	1.05 (1.03, 1.07)	4.18E-09	0.63	1 (0.98, 1.02)	0.9818	9.05E-06
rs6807275	3	14274451	*3p25.1*	G	A	0.72	1.07 (1.05, 1.09)	4.72E-13	0.72	0.98 (0.96, 1)	0.0240	6.48E-10
rs34989545	3	49622632	*BSN*	G	GT	0.19	1.06 (1.04, 1.08)	1.33E-09	0.19	1 (0.98, 1.02)	0.8235	1.89E-05
rs2082838	3	158305894	*MLF1*	A	G	0.2	1.06 (1.04, 1.08)	4.00E-09	0.2	1.01 (0.99, 1.03)	0.2538	0.0031
rs17042098	4	111664158	*4q25*	A	G	0.13	1.1 (1.07, 1.12)	4.34E-15	0.13	1.05 (1.03, 1.08)	3.24E-05	0.1400
rs11242464	5	138746334	*DNAJC18*	C	G	0.6	1.05 (1.03, 1.07)	2.32E-08	0.6	1 (0.98, 1.02)	0.8524	5.71E-05
rs9349379	6	12903957	*PHACTR1*	G	A	0.34	1.06 (1.05, 1.08)	4.63E-14	0.34	1 (0.98, 1.02)	0.9070	2.81E-07
rs146170154	6	36646768	*CDKN1A*	C	CTA	0.8	1.14 (1.12, 1.16)	5.14E-41	0.8	1.01 (0.99, 1.02)	0.5529	1.75E-18
rs1407229	6	53971921	*MLIP*	T	C	0.43	1.06 (1.04, 1.07)	2.97E-11	0.43	1 (0.99, 1.02)	0.7934	1.55E-07
rs66577603	6	79749166	*PHIP*	C	CTT	0.5	1.04 (1.03, 1.06)	4.77E-08	0.5	1.02 (1, 1.03)	0.0514	0.0362
rs282064	6	91303512	*MAP3K7*	T	G	0.33	1.05 (1.04, 1.07)	2.44E-10	0.33	1 (0.98, 1.01)	0.7016	0.0002
rs10455872	6	161010118	*LPA*	G	A	0.06	1.15 (1.12, 1.19)	2.29E-18	0.06	1.07 (1.04, 1.1)	4.14E-05	0.0229
rs3211938	7	80300449	*CD36*	G	T	0.09	1.2 (1.14, 1.28)	3.92E-10	0.09	0.94 (0.88, 1.01)	0.0744	3.20E-05
rs2270590	7	128455916	*CCDC136*	G	A	0.75	1.08 (1.06, 1.1)	5.28E-15	0.75	1.02 (1, 1.04)	0.0870	2.96E-06
rs7461129	8	125861374	*8q24.13*	C	T	0.73	1.07 (1.05, 1.09)	2.03E-14	0.73	0.98 (0.96, 0.99)	0.0097	1.82E-12
rs4977575	9	22124744	*RP11-145E5.5*	G	C	0.57	1.08 (1.07, 1.1)	5.37E-22	0.57	1.04 (1.02, 1.05)	2.39E-05	2.08E-06
rs10814273	9	35723234	*TLN1*	T	G	0.27	1.05 (1.03, 1.07)	3.12E-08	0.27	0.99 (0.98, 1.01)	0.4836	2.96E-07
rs600038	9	136151806	*9q34.2*	C	T	0.2	1.06 (1.04, 1.08)	2.06E-09	0.2	1.03 (1.01, 1.05)	0.0051	0.3167
rs485838	10	44750414	*10q11.21*	G	T	0.82	1.06 (1.04, 1.08)	1.94E-08	0.82	1 (0.98, 1.02)	0.6417	0.0080
rs148307915	10	49528021	*MAPK8*	A	G	0.12	1.08 (1.05, 1.1)	2.29E-09	0.12	1.02 (1, 1.05)	0.0969	0.0046
rs2242254	10	75585307	*CAMK2G*	G	A	0.87	1.07 (1.05, 1.1)	3.48E-10	0.87	1.03 (1.01, 1.05)	0.0159	0.0267
rs375034445	10	121424815	*BAG3*	A	AT	0.83	1.14 (1.12, 1.16)	5.09E-34	0.83	0.96 (0.94, 0.98)	1.06E-05	1.11E-30
rs113123267	11	47379410	*SPI1*	T	C	0.05	1.35 (1.26, 1.46)	2.74E-15	0.05	1.01 (0.92, 1.1)	0.8995	5.62E-05
rs111786504	11	55047396	*TRIM48*	G	A	0.03	1.61 (1.39, 1.86)	1.57E-10	0.03	1.04 (0.88, 1.23)	0.6498	0.0085
rs60504091	11	56329980	*OR5M11*	A	G	0.05	1.26 (1.18, 1.36)	3.56E-10	0.05	1.03 (0.95, 1.12)	0.5259	0.0028
rs658286	11	100942792	*PGR*	T	G	0.57	1.05 (1.03, 1.07)	1.01E-08	0.57	1 (0.99, 1.02)	0.6302	0.0011
rs11169562	12	51187290	*ATF1*	T	C	0.37	1.05 (1.03, 1.07)	1.80E-09	0.37	1 (0.99, 1.02)	0.8455	0.0001
rs4575361	12	124410529	*DNAH10; CCDC92*	A	T	0.7	1.05 (1.03, 1.07)	9.36E-09	0.7	0.98 (0.97, 1)	0.0390	1.79E-09
rs62006566	15	75086534	*CSK*	C	T	0.47	1.05 (1.03, 1.07)	2.69E-08	0.47	0.98 (0.97, 1)	0.0701	1.23E-07
rs7188250	16	53834607	*FTO*	C	T	0.34	1.06 (1.05, 1.08)	1.55E-13	0.34	1.06 (1.04, 1.08)	1.53E-11	0.9252
rs2224770	17	2205923	*SMG6*	T	C	0.43	1.05 (1.03, 1.07)	1.76E-09	0.43	1.02 (1, 1.03)	0.0369	0.0215
rs9907657	17	7460957	*TNFSF12; TNFSF12-TNFSF13*	T	G	0.17	1.06 (1.04, 1.09)	6.09E-09	0.17	1.03 (1.01, 1.06)	0.0024	0.0310
rs2517953	17	37841211	*PGAP3*	G	C	0.44	1.06 (1.05, 1.08)	5.84E-14	0.44	1.02 (1, 1.03)	0.0554	4.58E-05
rs9912468	17	64318357	*PRKCA*	G	C	0.42	1.05 (1.03, 1.06)	2.49E-09	0.42	1 (0.98, 1.01)	0.5849	1.61E-05
rs145606454	17	65893289	*BPTF*	A	AC	0.22	1.06 (1.04, 1.08)	3.97E-09	0.22	1.04 (1.02, 1.06)	4.28E-05	0.0183
rs7229491	18	46516424	*18q21.1*	C	G	0.68	1.05 (1.03, 1.07)	4.82E-09	0.68	1 (0.99, 1.02)	0.7260	0.0017
rs56968346	21	30540165	*MAP3K7CL*	T	C	0.2	1.09 (1.07, 1.11)	4.17E-17	0.2	1 (0.98, 1.02)	0.9518	1.09E-07
rs9608201	22	24166788	*SMARCB1*	A	G	0.72	1.07 (1.05, 1.09)	3.24E-14	0.72	0.99 (0.97, 1.01)	0.2530	1.80E-09
The 3 multi-ancestry HFpEF loci												
rs62048402	16	53803223	*FTO*	A	G	0.33	1.06 (1.04, 1.08)	2.33E-12	0.33	1.06 (1.05, 1.08)	1.10E-13	0.4632
rs75716431	17	66382647	*ARSG*	T	C	0.05	1.02 (0.98, 1.06)	0.2676	0.05	1.12 (1.08, 1.17)	4.27E-08	0.0362
rs113752382	21	18617840	*21q21.1*	T	C	0.06	1.04 (1, 1.09)	0.0596	0.06	1.12 (1.08, 1.17)	3.39E-08	0.1910

Association testing was performed under an additive genetic model using logistic regression, followed by random-effects meta-analysis using GWAMA. All GWAS association *P* values were two-sided. Genome-wide significance was defined as *P* < 5 × 10^−8^. Heterogeneity was assessed using Cochran’s Q test. No additional adjustment for multiple comparisons was applied to comparisons restricted to the identified genome-wide significant loci. For results externally evaluated in Biobank Japan, replication was defined as a two-sided *P* < 0.05 with a concordant direction of effect. No additional multiple-testing correction was applied to the replication analyses.

*Chr.* chromosome, *EA* effect allele, *NEA* non-effect allele, *EAF* effect allele frequency, *OR* odds ratio, *CI* confidence interval.

^a^Heterogeneity *P* value tests the difference in effect sizes between HFrEF and HFpEF.

Finally, we evaluated genomic inflation and LD score regression intercepts to determine whether elevation of the GWAS test statistics was attributable to population stratification or other confounding. For non-European ancestries, the inflation factors were close to one, indicating minimal inflation of test statistics. We observed moderate inflation for European GWAS (HFrEF: MVP 1.12; HFpEF: MVP 1.10; all-cause HF: MVP 1.18, HERMES 1.08, FinnGen 1.11; Levin et al. 1.12). We further calculated LD score regression intercepts, which were close to one, suggesting minimal confounding due to population stratification Supplementary Data 9. The same analyses performed on the GWAS meta-analysis showed similar findings, Supplementary Data 10.

### Heritability and genetic correlations

To quantify the contribution of common genetic variation to HF risk within ancestry groups, we estimated liability-scale SNP-based heritability using GREML-LDMS in MVP participants with individual-level genotype data. Among individuals of European ancestry, the liability-scale *h*^2^ was 0.31 for HFrEF (SE 0.06; 95% CI: 0.19–0.43), 0.26 for HFpEF (SE 0.06; 95% CI: 0.14–0.38), and 0.31 for all-cause HF (SE 0.04; 95% CI: 0.23–0.39). Among individuals of African ancestry, the corresponding estimates were 0.12 for HFrEF (SE 0.06; 95% CI: 0–0.25), 0.28 for HFpEF (SE 0.07; 95% CI: 0.14–0.42), and 0.21 for all-cause HF (SE 0.05; 95% CI: 0.12–0.30). Genetic correlations were estimated between HF subtypes and all-cause HF, with 0.55 (standard error [SE] 0.05, *p* < 0.0001) between HFrEF and HFpEF, 0.92 (SE 0.02, *p* < 0.0001) and 0.83 (SE 0.02, *p* < 0.0001) between all-cause HF and HFrEF, and all-cause HF and HFpEF, respectively.

### PheWAS of African-specific loci

To characterize the broader clinical associations and potential pleiotropic effects of HF-associated variants identified among African-ancestry participants, we conducted an exploratory PheWAS across 1709 phecode-based phenotypes. Statistical significance was defined as FDR-adjusted *q* < 0.05. A total of 54 gene-phenotype associations were identified for HFrEF loci, while no statistically significant associations were found for HFpEF, Supplementary Data 11. The four African-specific HFrEF loci *CD36*, *SPI1*, *TRIM48*, and *SPNS3* were associated with various cardiovascular phenotypes related to HF, such as cardiomyopathy, hypertensive heart disease, and other related cardiac disorders, Supplementary Data 11 and Supplementary Fig. 7A–L. A total of 19 statistically significant gene-phenotype associations for all-cause HF were observed.

### Gene-based analysis, tissue expression, enrichment analysis and overlap with enhancers

The gene-based analysis of multi-ancestry GWS loci of HFrEF and HFpEF revealed 74 and six genes, respectively. The analysis for all-cause HF identified 141 genes, Supplementary Data 12A–C. These findings aggregate association signals across variants within genes and therefore provide complementary evidence for candidate genes at HF-associated loci.

To investigate the tissues in which HF-associated variants may influence gene regulation, we examined tissue-specific cis-eQTL annotations from GTEx. We found that multi-ancestry HFrEF loci harbored significant *cis*-eQTLs in the tibial artery, the coronary artery and the aorta, indicating the potential mechanism of narrowing or blockage of the arteries, coronary artery disease, and arterial stiffness of the aorta. In contrast, multi-ancestry HFpEF loci showed significant eQTLs in the pituitary gland, adrenal gland, and thyroid, suggesting mechanisms in fluid balance, blood pressure regulation, and cardiac function, Supplementary Data 13 and 14.

Gene-set analysis using DEPICT for the GWS loci of HFrEF showed SMAD protein–protein interaction contributing to various cellular processes in development and disease^16^. For HFpEF, no gene sets reached significance when restricting the analysis to the GWS loci. Therefore, in an exploratory analysis, we included nominally significant loci with *p* < 1 × 10^−4^, which resulted in gene sets in relation to abnormal muscle regeneration and abnormal vascular smooth muscle morphology; however, not reaching statistical significance, Supplementary Data 15A–C. Pathway enrichment analysis did not result in statistically significant tissue types after correcting for multiple testing, Supplementary Data 16A–C.

To determine whether HF-associated variants were also associated with circulating protein abundance, using protein-quantitative trait loci from the Fenland proteogenomic study of 4775 protein targets^17^, we examined the risk loci for all-cause HF and its subtypes to explore potential molecular mechanisms. These analyses indicated that genetic variants at HF risk loci may potentially influence circulating protein expression levels. For example, rs600038 (*9q34.2*) and rs653178 (*ATXN2*) were each associated with the levels of more than 170 protein targets, Supplementary Data 17A–C. To further investigate potential regulatory mechanisms, we overlapped the lead GWAS variants with tissue-specific enhancer annotations derived from the EnhancerAtlas 2.0 database^18^, which included 295 human tissue and cell-specific enhancers. A substantial fraction of the HF loci fell within predicted enhancer regions: 35 of the 46 multi-ancestry HFrEF loci, all 3 HFpEF loci, and 112 of the 136 all-cause HF loci overlapped at least one enhancer region in various cell types, Supplementary Data 18A–C. Notable examples include HFrEF loci at *ACTN2, BAG3, PHACTR1, PRKCA, MAP3K7CL*, and *SMARCB1*, as well as the HFpEF loci *FTO* and *ARSG*, all of which map to enhancer regions that are active in cardiac and vascular cell types.

### TWAS, colocalization analysis, and gene prioritization

To identify genes whose genetically predicted expression was associated with HF and to move beyond proximity-based assignment of GWAS loci, we performed tissue-specific TWAS. TWAS of HFrEF loci suggested genes of diverse functions and are implicated in different biological processes, including *CLCNKA*, *ATF1,* and *PGAP3*, in both cardiovascular and skeletal muscle tissue types. For HFpEF, four genes, including *FTO*, *CRHR1,* and *LIME1,* are enriched in skeletal muscle, and *ARHGAP27* is enriched in the atrial appendage. Most of the TWAS findings (79%, 50% and 67% for HFrEF, HFpEF and all-cause HF, respectively) had a posterior probability of colocalization >0.75, indicating a potential functional relationship or shared genetic basis, Supplementary Data 19A–C, Fig. 1, and Supplementary Fig. 2.

To further prioritize candidate genes at HF-associated loci, we applied PoPS^19^ to the multi-ancestry meta-analysis results for HFrEF, HFpEF, and all-cause HF. Across 18,383 tested genes, PoPS scores were generated separately for each HF phenotype. For each sentinel GWS locus, we identified genes located within ±500 kb of the sentinel variant and selected the gene with the highest PoPS score as the prioritized candidate gene for that locus. The PoPS-prioritized genes are presented in Supplementary Data 20. Several PoPS-prioritized genes overlapped with loci or candidate genes highlighted in our GWAS and downstream analyses. For HFrEF, these included *NFIA, MAPK8, PHACTR1, CAMK2G, CCDC136, FTO, BPTF*, and *SMG6*. For HFpEF, overlapping genes included *FTO* and *ARSG*. For all-cause HF, overlapping genes included *NFIA, SMG6, PHACTR1*, and *FTO*.

### Functional annotation and chromatin state analysis

To assess whether HF-associated loci localize to regulatory elements active in cardiac tissues, we integrated the GWAS findings with chromatin-state annotations from Roadmap Epigenomics and ENCODE. This analysis provides an independent line of functional evidence by identifying variants located within promoters, enhancers, or other regulatory regions. For HFrEF, several loci localized to enhancer or promoter states, including *CDKN1A* in active TSS across atrial and ventricular tissues and *TTN* within genic enhancers, Supplementary Data 21A. For HFpEF, *ARSG* was consistently annotated as enhancers across cardiac tissues, Supplementary Data 21B. For all-cause HF, regulatory enrichment was observed at *NKX2-5*, *SYNPO2L,* and *BAG*3 in enhancer regions, Supplementary Data 21C.

### Credible set analysis and fine-mapping

Credible set analyses for the ancestry-specific and multi-ancestry GWS loci of all-cause HF and its subtypes identified sets of SNPs with a cumulative posterior probability of 0.9 or 0.95 of including the causal variant, thereby prioritizing a limited number of variants for future functional investigation, Supplementary Data 22A–C. Among the 46 GWS loci identified in the multi-ancestry GWAS of HFrEF, ancestry-specific and shared-ancestry fine mapping revealed that 36 formed 95% credible sets. Seven loci contained potential causal SNPs with PIP >0.5, including *NOS1AP, TTN, PHACTR1, CDKN1A, MAPK8, SPI1*, and *18q21.1*. For HFpEF, 2 of the 3 GWS loci formed 95% credible sets, but no SNP had PIP >0.5, Supplementary Data 23.

### Therapeutic target profile

TWAS-prioritized genes showed varying levels of tractability in open targets. *FTO* exhibited evidence from advanced clinical studies, while *CLCNKA, PHACTR1, ATF1, MAP3K7, BPTF, NPC1, CAMK2D*, and *BDNF* had preclinical or experimental support for druggability, Supplementary Data 24A.

Overall, the queries from the DrugBank indicate that the genetic architecture of HF identified in our GWAS is not simply a reflection of existing pharmacologic pathways targeted by current HF guideline-directed medical therapy. Instead, the gene-drug links we identified are primarily hypothesis-generating signals that highlight potential avenues for drug repurposing and motivate further experimental work, Supplementary Data 24B.

### Mendelian randomization (MR)

Genetically predicted coronary artery disease, body mass index, low-density lipoprotein cholesterol, systolic blood pressure, and diastolic blood pressure were associated with HFrEF, providing MR evidence supporting their causal roles in HFrEF. In contrast, MR analyses did not provide evidence supporting causal roles of high-density lipoprotein cholesterol or total cholesterol in HFrEF. Genetically predicted type 2 diabetes, triglycerides, and pulse pressure showed suggestive associations with HFrEF, but these findings were less consistent across sensitivity analyses and should be interpreted cautiously. For HFpEF, genetically predicted coronary artery disease, body mass index, systolic blood pressure, diastolic blood pressure, and pulse pressure were associated with HFpEF, providing MR evidence supporting their causal roles. MR analyses did not provide evidence supporting causal roles of lipid traits in HFpEF, Supplementary Data 25A, B.

## Discussion

The results of our study provide important insights into the genetic architecture of all-cause HF and its subtypes, HFrEF and HFpEF. In this large multi-ancestry study, we identified 46 GWS loci for HFrEF, 3 loci for HFpEF, and 136 loci for all-cause HF. Of these, 9 HFrEF loci, 1 HFpEF locus, and 12 all-cause HF loci were potentially novel compared with previously reported HF GWAS^9–13^. When we compared effect estimates between subtypes at the lead SNPs for the 46 multi-ancestry HFrEF loci, 42 showed evidence of heterogeneous effects between HFrEF and HFpEF (Cochran’s Q *p* < 0.05, Table 3). Taken together with the limited overlap in GWS loci for HFpEF, these findings add to prior clinical and genetic evidence that HFrEF and HFpEF have only partially shared genetic determinants and likely involve partly distinct underlying biological mechanisms.

The *FTO* locus, originally identified as the first common obesity-susceptibility locus in BMI GWAS and repeatedly implicated in large consortia and mechanistic studies, supports a shared genetic etiology between obesity and both HFrEF and HFpEF^20–23^. Several HFrEF loci showed a reversed effect on HFpEF, including *TTN, BAG3,* and *CLCNKA*, which can be explained by their established associations with LVEF or LV end-diastolic volume in the general population^24^. Since the HF subtypes were defined based on LVEF, the effect alleles of these loci showed a higher risk in HFrEF with lower LVEF or LV end-diastolic volume, and a lower risk in HFpEF, with higher LVEF or LV end-diastolic volume. The HFrEF-associated locus at *DNAH10/CCDC92* plays key roles in adipocyte function and differentiation^25^, with *CCDC92* previously identified as a risk loci for type 2 diabetes and coronary heart disease^26^, and *DNAH10* identified as a key gene in modulating high-density lipoprotein cholesterol^27^.

Gene-set enrichment analysis using DEPICT showed that two HFrEF loci, including *CDKN1A* and *MAP3K7*, mapped to protein-protein interaction subnetworks centered on Smad (Sma- and Mad-related) signal transduction proteins, key mediators of transforming growth factor-β (TGF-β) signaling, suggesting a potential contribution of TGF-β/SMAD pathways to HFrEF pathogenesis^28,29^. Further, TGF-β/Smad signaling in macrophages after myocardial infarction can regulate repair and remodeling of the heart^30^. Modulating the TGF-β/Smad pathway or targeting specific components within this pathway may offer potential therapeutic approaches for mitigating cardiac remodeling and managing HFrEF.

The African-specific HFrEF locus *CD36* has an established role in mediating a range of critical cellular processes. It acts as a multifunctional receptor, plays a role in lipid handling, platelet function, inflammation, angiogenesis and fatty acid transport within the cardiovascular system^31^. *CD36* acts as a long-chain fatty acid transporter in cardiac myocytes and has function as a receptor for various ligands in endothelial cells, with a specific emphasis on its role in the context of diabetic cardiomyopathy^31–35^. *SPI1* is implicated in cardiovascular processes due to its role in gene expression regulation and impacts pathways related to inflammation and cardiac remodeling^36^. The observation that the 4 HFrEF loci, *CD36*, *SPI1*, *TRIM48*, and *SPNS3* were rare within the European population provides valuable insights into the genetic diversity of HFrEF. Loci, such as *PSRC1* and *CSK,* highlight alleles with cross-ancestry relevance but marked differences in population frequencies. Variants at *PSRC1* have been consistently associated with lipid metabolism and coronary artery disease across multi-ethnic cohorts^37^. Similarly, *CSK* variants influence blood pressure regulation and hypertension risk, with stronger effects in non-European populations where the risk allele is more common^38^. Further, the HFpEF locus *ARSG* was previously identified as a risk locus for blood pressure and hypertension risk in Korean populations^39^, and its role in HFpEF among African ancestry may be through regulating blood pressure^39^. In addition, among individuals of African ancestry, two HFrEF loci, *HSPB7* and *CCDC141,* showed larger effect sizes compared to the European ancestry, highlighting the roles of heat shock protein and heart rate in HF among African populations^40,41^.

Ancestry-specific and shared-ancestry fine mapping identified the majority of the HFrEF loci with 95% credible sets and a high PIP, indicating a strong level of confidence in the potential causal genetic effects. For instance, *TTN* is a well-established gene in the context of HF and dilated cardiomyopathy and has been linked to structural defects in the heart muscle, contributing to myocardial dysfunction and the progression of HF^14,42,43^. Another notable gene is *NOS1AP*, which plays a crucial role in nitric oxide signaling and vascular function. Dysfunctional nitric oxide signaling has been implicated in various cardiovascular diseases, including HF^44^. The identification of causal SNPs in *NOS1AP* further supports its potential relevance in HFrEF, as it may contribute to impaired endothelial function and myocardial remodeling. *MAPK8* is a component of the mitogen-activated protein kinase signaling pathway and has been extensively studied in the context of cellular stress, inflammation, and cardiac remodeling^45^. The identification of potential causal SNPs in *MAPK8* suggests that dysregulated stress responses may contribute to the pathophysiology of HFrEF by promoting adverse heart remodeling.

One of the major strengths of our study is the utilization of a large and diverse sample from multiple cohorts with rich phenotyping of HF subtypes, offering a comprehensive view of the genetic architecture of HF and its subtypes across diverse backgrounds. By incorporating data from diverse populations, including European, African, Hispanic, and Asian ancestries, our study achieved high diversity and statistical power, enabling the detection of novel genetic variants and risk loci associated with all-cause HF, HFrEF and HFpEF. The limited number of identified HFpEF loci as compared to HFrEF and the challenges posed by the heterogeneity of HFpEF underscore the need for further research and precision in sub-phenotyping of HFpEF as a complex and heterogeneous clinical syndrome, characterized by a diverse range of underlying pathophysiological mechanisms.

Our study has several limitations. The MVP cohort was predominantly composed of male veterans, who accounted for 92% of participants. This limits our power for sex-stratified analyses, particularly among females, and may constrain the generalizability of our findings to females. Larger and more demographically diverse cohorts, including those with a higher proportion of females, will be needed to fully characterize sex-specific genetic architecture and to confirm the applicability of these findings across broader clinical populations. Although our study included participants of European, African, Hispanic, and Asian ancestry, the numbers of HFrEF and HFpEF cases of Hispanic and Asian ancestry were modest. As a result, no loci reached genome-wide significance in ancestry-specific analyses for these groups, and Hispanic and Asian participants contributed primarily through the multi-ancestry meta-analyses. Larger HF cohorts in Hispanic- and Asian-ancestry populations will be needed to achieve adequate power for ancestry-specific discovery and to more fully characterize genetic risk in these groups. The heritability estimate for HFpEF was higher than that for HFrEF among African-ancestry participants. However, the confidence intervals were wide and overlapping, indicating substantial uncertainty around the relative magnitude of subtype-specific heritability estimates. In addition, differences in sample size, age and sex distributions, phenotype heterogeneity, and case ascertainment across HF subtypes and ancestry groups may influence SNP-based heritability estimates. Therefore, these differences should be interpreted cautiously and should not be taken as definitive evidence that HFpEF has a greater genetic contribution than HFrEF in African-ancestry participants. Further, several identified loci, including *TTN*, overlap genes known to harbor rare pathogenic variants for inherited cardiomyopathies, particularly dilated cardiomyopathy^46^. Although the identified GWAS signals were driven by common or low-frequency sentinel variants, we cannot exclude the possibility of synthetic associations, whereby unmeasured rare pathogenic variants contribute to the observed association signals^47^. In addition, although we compared effect estimates across ancestries and reported heterogeneity statistics, we did not apply a formal power-adjusted framework for non-European populations. Therefore, some loci that appear non-significant or attenuated in non-European ancestries may simply reflect limited statistical power rather than a true ancestry-specific absence of effect. Finally, although we demonstrate internal consistency across ancestries and cohorts and external replication in BioBank Japan, further validation in independent, deeply phenotyped non-European populations with detailed HF subtyping will be important to confirm and extend our findings.

## Methods

### Study cohorts, genotyping, and phenotyping

#### The Million Veteran Program (MVP) cohort

The MVP is a large, ongoing, multi-ancestry mega-biobank prospective cohort study that began enrollment in 2011, with participants recruited from over 60 sites across the US^48^. The source population of the MVP is all patients of the Veterans Health Administration who were able to provide informed consent. In-depth phenotyping, including demographics, health condition, lifestyle, medical history, medication use, family history, diet and environmental exposure are available for this cohort^48^. Affymetrix Axiom array was used for genotyping, respectively, then imputed to the 1000 Genomes phase 3 (version 5) reference panel^49^. Genetic variants were mapped to the human genome reference assembly version GRCh37/hg19. Individual ancestries for European, African, Hispanic and Asian were estimated and defined using the algorithm of harmonizing genetic ancestry and self-identified race/ethnicity (HARE), which incorporated both self-reported race/ethnicity and genetic profile and successfully assigned ancestries for >98% participants to the following four non-overlapping ancestry groups: non-Hispanic white (European), non-Hispanic Black (African), Hispanic, and non-Hispanic Asian participants. We therefore use the terms “European ancestry,” “African ancestry,” “Hispanic ancestry,” and “Asian ancestry” to refer specifically to these HARE-defined groups within MVP, which combine self-identified race/ethnicity with genetic information. For the GWAS, single-nucleotide polymorphisms (SNP) were retained for analysis if imputation quality >0.3, Hardy–Weinberg equilibrium (HWE) *p* > 10^−20^, missing rate <0.05, and minor allele frequency (MAF) >0.01. All-cause HF, HFrEF, and HFpEF included both prevalent and incident cases, which were determined based on International Classification of Diseases (ICD) codes 428.x for ICD-9 and I50.x for ICD-10, along with an echocardiographic assessment conducted within 6 months of diagnosis. We utilized a comprehensive, physician-reviewed algorithm to detect cases within the electronic health records and distinguish HF into HFrEF and HFpEF. We implemented a natural language processing method to analyze both structured and unstructured components of the electronic health records, extracting LVEF values from echocardiography reports, including those within the VA system and external healthcare facilities, reducing the risk of misclassification due to missing LVEF data^50^. In brief, the NLP component uses rule-based pattern matching to identify and extract numeric LVEF values, measurement context (e.g., transthoracic vs. transesophageal echocardiography), and timing from free-text echocardiography reports, and then links these values to HF encounters in the structured electronic health records. This algorithm has been previously developed and validated, demonstrating high positive predictive value for HFpEF case identification in an independent cohort^50^. Individuals with an HF diagnosis were categorized as having HFpEF if their earliest documented LVEF was ≥50%, and as HFrEF if their initial recorded LVEF was ≤40%. The accuracy of the phenotyping algorithm was confirmed through blinded physician assessment of medical records, achieving a positive predictive value of 96%. The classification and refinement of HFpEF and HFrEF groups have also been corroborated in epidemiological and genetic research^9,50,51^.

The MVP study was approved by the U.S. Department of Veterans Affairs Central Institutional Review Board. All MVP participants provided written informed consent.

#### The Vanderbilt University DNA Databank (BioVU)

Enrollment in the BioVU started in 2004, and this large, multi-ancestry biorepository was constructed through the donation of excess blood samples from clinical testing conducted on Vanderbilt clinic patients, with informed consent obtained. Phenotyping of this cohort was derived by linking to electronic medical records at Vanderbilt University^52^. Genotyping was performed using the Illumina MEGA-Ex array, then imputed to the same 1000 Genomes Project Phase 3 reference panel, and mapped to the same human genome reference assembly version as in the MVP, GRCh37/hg19^49^. In BioVU, genetic ancestry was inferred using STRUCTURE 2.2^53^ applied to a panel of 360 ancestry-informative markers genotyped on the Illumina DNA test panel, as previously described. Briefly, for each individual, probabilities in ancestry clusters were estimated corresponding to reference European and African populations; participants were assigned to European- or African-ancestry groups based on their highest posterior membership probability^54,55^. SNPs were retained for analysis if imputation quality >0.3, HWE *p* > 10^−6^, missing rate <0.05, and MAF >0.01. The phenotyping protocol for all-cause HF, HFrEF, and HFpEF was the same as in the MVP^9^.

The BioVU study was approved by the Vanderbilt University Medical Center Institutional Review Board. All BioVU participants provided written informed consent.

#### The HEart Failure Molecular Epidemiology for Therapeutic TargetS (HERMES) consortium

The HERMES is a large consortium which includes 51 studies from 11 countries^56^. We used the GWAS meta-analysis summary statistics for all-cause HF, which includes 26 cohorts of European ancestry, totaling 47,309 all-cause HF cases and 930,014 controls^10^. Cases were participants with a clinical diagnosis of HF of any etiology, without selection based on left ventricular ejection fraction, and controls were participants without HF.

#### FinnGen

FinnGen is a research initiative that collaborates with both public and private entities and uses imputed genotype data from Finnish biobanks linked to nationwide digital health records^57^. This study population represents a genetically distinct population within Europe. We used the GWAS summary statistics for all-cause HF among European ancestry participants in FinnGen, which included 23,622 all-cause HF cases and 317,939 controls^57^. The software LiftOver^58^ was used to map genomic positions from GRCh38/hg38 to GRCh37/hg19. Variants that could not be uniquely mapped to GRCh37/hg19 or could not be harmonized by chromosome, position, and effect allele were excluded before meta-analysis.

Additionally, we used the GWAS summary data from a recent multi-ancestry all-cause HF meta-analysis by Levin et al., which included 115,150 cases and 1,550,331 control individuals of diverse genetic ancestry^12^. The number of cases and controls, genotyping quality control filters, and the total number of genetic variants passed quality control by study and ancestry are shown in Supplementary Data 1A.

### GWAS of HFrEF, HFpEF, and all-cause HF

For the MVP and BioVU, autosomal SNPs were analyzed using logistic mixed models implemented in REGENIE^59^ for HFrEF, HFpEF, and all-cause HF, separately by study and ancestry. Under an additive genetic model, REGENIE was run in its standard two-step framework. We first fitted a whole-genome ridge regression model using high-quality genotyped autosomal variants after linkage disequilibrium (LD) pruning (window size 50 SNPs, shift window size 5 SNPs, LD threshold 0.5). Then, we tested imputed SNP dosages for association with each HF phenotype using logistic mixed models, adjusting for age at enrollment, sex, and the top 10 principal components derived from the full genomic data, separately by study and ancestry. This mixed-model framework accounts for both relatedness and residual population structure within each ancestry group. Effect estimates were reported as log odds ratios with standard errors and were subsequently converted to odds ratios for presentation.

### Ancestry-specific and multi-ancestry meta-analysis of HFrEF, HFpEF, and all-cause HF

For European ancestry, we performed a meta-analysis of the HF subtypes, including 29,062 HFrEF cases, 29,281 HFpEF cases, and 380,050 controls from the MVP and the BioVU. The all-cause HF meta-analysis combined the MVP, BioVU, HERMES^10^, and FinnGen^57^, totaling 135,310 all-cause HF cases and 1,628,003 controls. For African ancestry, we analyzed 7637 HFrEF cases, 6779 HFpEF cases, 15,925 all-cause HF cases, and 96,026 controls from the MVP and the BioVU.

We additionally performed a multi-ancestry meta-analysis. For the HF subtypes, 38,781 HFrEF cases, 38,163 HFpEF cases, and 526,135 controls of European, African, Hispanic, and Asian ancestries from the MVP and the BioVU were analyzed. We additionally meta-analyzed 200,070 all-cause HF cases, and 2,076,466 controls form the MVP, the BioVU, and the recent multi-ancestry GWAS of all-cause HF by Levin et al.^12^. Supplementary Data 1A, B, and Supplementary Fig. 1 illustrate the numbers of cases and controls by study and ancestry.

The meta-analyses were conducted using the Genome-Wide Association Meta-Analysis (GWAMA) software^60^, which implements tests for heterogeneity across studies and ancestries, and applies random-effects meta-analysis to account for heterogeneity in genetic effect sizes. GWS independent loci were defined by *p* < 5 × 10^−8^, LD (*r*^2^) < 0.6, and a distance greater than 250 kb. Novel genomic loci, when compared with previous HF GWAS^9–13^, were defined as non-overlapping physical distances located greater than 500 kb from the genetic variant on both sides. GWS loci were mapped the nearest protein-coding gene which was located within ±10 kb region. If the significant SNP was >10 kb from any annotated protein-coding gene, the locus was labeled by its cytogenetic band.

### External validation in the Biobank Japan

External validation was performed using the independent Japanese GWAS of HF from BioBank Japan, which includes 16,251 all-cause HF cases and 197,577 controls (*N* = 213,828), 4254 HFrEF cases and 197,577 controls (*N* = 201,831), and 7154 HFpEF cases and 197,577 controls (*N* = 204,731), as reported by Enzan et al.^61^. A locus was considered replicated when the lead variant showed a concordant direction of effect and a two-sided *p* < 0.05.

### Phenome-wide association study (PheWAS) of African-specific loci

To explore pleiotropy and provide additional biological context for African-specific HF loci *CD36*, *SPI1*, *TRIM48*, and *SPNS3*, we conducted an exploratory, hypothesis-generating PheWAS of African-ancestry risk variants for HFrEF, HFpEF, and all-cause HF in the MVP African cohort. Phenotypes were derived from the VA electronic health record using the established MVP phenome framework, in which ICD-9 and ICD-10 diagnosis codes are aggregated into clinically meaningful phecodes by grouping related codes into disease categories^62^. Using this framework, we analyzed 1709 phecode-based phenotypes^62,63^. For each HF risk variant-phecode pair, we fit logistic regression models adjusting for age, sex, and the top 5 principal components, and controlled for multiple testing using the false discovery rate^64^ (FDR), with *q* < 0.05 considered statistically significant.

### Genomic inflation, LD score regression, heritability, and genetic correlation

For the meta-analysis results and each GWAS summary by study and ancestry, we calculated the inflation factors based on the median of the observed test statistics divided by the expected median under the null hypothesis. Using LDSC^65,66^ based on the GWAS summary statistics for European ancestry, we computed the LD score intercepts to examine the potential confounding effects of population stratification in the GWAS results. LD score regression analyses, including LDSC intercept estimation and genetic correlation analyses, were restricted to European-ancestry GWAS summary statistics and used the 1000 Genomes Phase 3 European LD score reference panel. We estimated SNP-based heritability (*h*^2^) for HF and subtypes separately in European and African participants using GREML-LDMS in GCTA among MVP participants with individual-level genotype data^67,68^. Analyses were performed separately within ancestry groups. Variants were stratified into minor allele frequency and linkage disequilibrium bins, and genetic relationship matrices were constructed for each bin. REML models included age, sex, and the top 10 genetic PCs as covariates. Observed-scale estimates were converted to the liability scale using assumed disease prevalences of 5% for HFrEF, 5% for HFpEF, and 10% for all-cause HF, which were broadly consistent with the observed prevalence of these phenotypes in the MVP analytic sample. Further, genetic correlations^69^ were estimated between HFrEF, HFpEF, and all-cause HF based on the meta-analysis summary statistics of individuals with genetic ancestry similar to reference populations of European ancestry. For each phenotype pair, variants were harmonized to the same effect allele and restricted to the common set of variants available in both GWAS.

### Gene-based analysis, tissue expression, enrichment analysis, and overlap with enhancers

Based on the multi-ancestry GWAS summaries of HFrEF, HFpEF, and all-cause HF, we used MAGMA (multivariate gene-based analysis of genome-wide association studies) for gene and gene-set analysis of aggregating genetic signals within individual genes to identify gene-based associations beyond the single-marker level^70^. We used the 1000 Genomes Phase 3 all-population reference panel, including European, African, Admixed American, East Asian, and South Asian populations, as the LD reference panel.

Tissue expression analysis of 54 specific tissue types was performed using the Genotype-Tissue Expression (GTEx) dataset, which provides comprehensive information on gene expression across human tissues of a wide range of organs and biological systems. Additionally, an analysis of 30 more generalized tissue types, without specifying subtypes or regions, was performed to understand gene expression across major tissues.

To further explore the functional relevance of genes associated with HFrEF, HFpEF, and all-cause HF among European ancestry, we conducted gene set analysis and tissue enrichment analysis using the DEPICT (Data-driven Expression-Prioritized Integration for Complex Traits) tool^71,72^. The gene set enrichment analysis was based on curated gene sets, including Gene Ontology (GO)^73^ and Kyoto Encyclopedia of Genes and Genomes (KEGG)^74^; the tissue type enrichment analysis was based on Medical Subject Heading (MeSH) tissue and cell type annotations^75^. FDR^64^ was applied, with significance defined by a corrected *q* < 0.05.

We obtained protein-quantitative trait loci (pQTLs) from the Fenland study, a genome-proteome-wide association study in 10,708 European-descent individuals. The genome-proteome-wide association study was performed using 10.2 million genetic variants, including plasma abundances of 4775 distinct protein targets measured using the SOMAscan V4 assay in plasma^17,76^. The SOMAscan assay employs single-stranded oligonucleotides (aptamers) with specific binding affinity to a single protein. We retrieved functional annotations from the Fenland proteo-genomic study^17^ for each SNP we identified for HFpEF, HFrEF, and all-cause HF, matched by chromosomal position and reference allele. Bonferroni correction was applied to account for multiple testing. All variants were aligned to the GRCh37/hg19 genome build, and effect estimates were harmonized to the HF risk allele. Variants with incompatible allele coding or unresolved strand orientation were excluded.

Further, to identify overlapping enhancer regions that may be involved in HF subtypes and all-cause HF, we used the EnhancerAtlas 2.0 database^18^, which included 295 human tissue and cell-specific enhancers. A locus was considered to overlap an enhancer when the genomic position of the lead or proxy variant fell within an annotated enhancer interval.

### Transcriptome-wide association study (TWAS), colocalization analysis, and gene priortization

TWAS analyses were restricted to European-ancestry GWAS summary statistics and used FUSION/GTEx expression weights with matched European LD reference panels. The TWAS analysis pipeline was implemented using the software FUSION^77^. We utilized the reference datasets obtained from GTEx (Genotype-Tissue Expression, V8)^78^, containing gene expression profiles across multiple tissues, including those relevant to HF, such as aorta, coronary artery, tibial artery, atrial appendage, left ventricle, and skeletal muscle. The tissue-specific gene expression weights were used to prioritize loci that contribute to HF and its subtypes while accounting for the LD structure. Bonferroni correction based on *p* < 1.16 × 10^−6^ (0.05/43114) was applied to control for multiple testing. In addition, colocalization analysis was conducted to identify colocalized regions between gene expression and HF subtypes and all-cause HF using the coloc package in R^79–81^. In colocalization, five hypotheses were evaluated: there is no association between the gene expression and HF; there is a significant association between the gene expression and HF, but this association is driven solely by the functional effects of the gene expression; there is a significant association between the gene expression and HF, but this association is driven solely by the genetic variants identified in GWAS; both the gene expression and HF have independent associations with different genetic variants; there is evidence for colocalization, indicating that the gene expression and HF signals share common causal variant. We defined colocalization when the maximum posterior probability of a sharing causal variant between the gene expression and HF association is >0.75^82^.

We used polygenic priority score (PoPS)^19^ to prioritize candidate genes for HFrEF, HFpEF, and all-cause HF. GWAS summary statistics from the multi-ancestry meta-analyses were used, matching to the 1000 Genomes Phase 3 multi-ancestry reference panel, including all available ancestry populations as the LD reference panel for gene-based analysis. Variants were harmonized to the reference panel by chromosome, genomic position, and alleles, and variants with unresolved allele mismatches, duplicate identifiers, or missing association statistics were excluded. MAGMA^70^ was used to generate gene-level association statistics using the SNP-wise mean gene model. PoPS was then run separately for each HF phenotype. A total of 18,383 genes were tested, and the results were ranked by PoPS score. For each sentinel GWS locus, we identified all genes located within ±500 kb of the sentinel variant and selected the genes with the highest PoPS score as the prioritized candidate gene for that locus.

### Functional annotation and chromatin state analysis

We additionally performed functional annotation of GWS loci using chromatin state data from the Roadmap Epigenomics^83^ and ENCODE^84^. Specifically, we overlaid GWAS sentinel SNPs with the 15-core ChromHMM model^85^ across cardiac tissues, including fetal heart, right atrium, left ventricle, right ventricle, and aorta. Each variant was annotated with regulatory states, enabling SNP-level classification into promoter, enhancer, transcribed, or repressed elements. Because a variant could map to different chromatin states across cardiac tissues, annotations were retained separately for each tissue rather than assigning a single consensus state. These analyses were descriptive and were used to provide functional context.

### Credible set analysis and fine-mapping of causal variants

To identify credible sets of causal variants associated with HF subtypes and all-cause HF, we utilized the R package LDlinkR^49,86^ to identify proxy and putative functional variants within a two-sided window of 500 kb of each GWS SNP with a pairwise *R*^2^ > 0.01 based on the 1000 Genomes reference panel^49^. Then, we employed the R package corrcoverage^87–89^ to determine the credible sets of causal variants from Bayesian genetic fine-mapping. To define the credible sets at a high confidence level, we set thresholds of 0.90 and 0.95 on the posterior probability of inclusion for each variant.

MESuSiE^90^ was used for fine-mapping among the European, African and Hispanic cohorts within the MVP. Ancestry-specific LD matrices were estimated from MVP individual-level genotype data for the corresponding European, African, and Hispanic ancestry groups. For the GWS loci identified in the multi-ancestry meta-analysis of GWAS on HFrEF and HFpEF, fine-mapping was performed based on a 1-Mb window centered on each GWS loci, extending 500 kb upstream and 500 kb downstream. Ancestry-specific and cross-ancestry signals with a posterior inclusion probability (PIP) threshold of 0.5 and 95% credible sets were identified.

### Therapeutic target profile

We queried TWAS-prioritized genes in Open Targets^91^ (release 17 September 2025) to evaluate their therapeutic tractability. Loci that reached genome-wide significance and had a Bonferroni-corrected *p* < 0.05 in TWAS were assessed for evidence of tractability, including targets of approved drugs, drugs in clinical trials, preclinical small molecules, or predicted druggability by small molecules or antibodies. The highest level of available evidence was recorded for each gene, together with the corresponding drug-development phase and modality. We additionally queried DrugBank 6.0^92^ using gene symbols and corresponding UniProt identifiers. For each gene, we extracted all drugs with a documented interaction with the encoded protein. Drug-target relationships were summarized descriptively and were not interpreted as evidence that modulation of the corresponding gene would prevent or treat HF. Open targets tractability classifications and DrugBank interactions were considered hypothesis-generating because they may reflect evidence obtained in diseases other than HF and do not establish the direction, tissue specificity, safety, or therapeutic effect of target modulation.

### Mendelian randomization (MR)

Based on the results from the European ancestry, MR was performed to explore the potential causal associations between HFrEF, HFpEF and several risk factors, including coronary artery disease, type 2 diabetes, body mass index, high-density lipoprotein cholesterol, low-density lipoprotein cholesterol, total cholesterol, triglycerides, systolic blood pressure, diastolic blood pressure, and pulse pressure. The genetic instruments for the risk factors were selected from recent large-scale GWAS of European or European-dominant populations^93–97^. LD clumping of genetic instruments was performed using the 1000 Genomes Phase 3 European reference panel, consistent with the ancestry of the exposure GWAS instruments. MR analyses were performed using the R package TwoSampleMR^98^. The primary inference was based on the fixed-effect inverse-variance weighted (IVW) method and its two-sided *P* value. MR-Egger regression, weighted median, and weighted mode methods were performed as sensitivity analyses to assess the robustness of the IVW estimates under different assumptions regarding horizontal pleiotropy and invalid instruments. Potential directional pleiotropy was evaluated using the MR-Egger intercept test. In addition, MR-PRESSO (Mendelian Randomization Pleiotropy RESidual Sum and Outlier)^99^ was used to detect and correct for pleiotropic outlier variants, and MR analyses were repeated after removing genetic outliers that could bias the causal estimates. MR findings were interpreted as more robust when the IVW association was statistically significant, the direction of effect was consistent across sensitivity methods, and the estimates were not materially changed after outlier removal.

### Reporting summary

Further information on research design is available in the Nature Portfolio Reporting Summary linked to this article.

## Supplementary information

Supplementary Information

Description of Additional Supplementary Files

Supplementary Data 1–25

Reporting Summary

Transparent Peer Review File

## Data Availability

Due to US Department of Veterans Affairs (VA) regulations and our ethics agreements, the analytic datasets used for this study are not permitted to leave the Million Veteran Program (MVP) research environment and VA firewall. This limitation is consistent with other MVP studies based on VA data. Access is limited to eligible VA-system researchers with an approved VA and MVP research protocol and must occur within an approved VA computing environment. Information regarding applications may be obtained from the MVP Program Office at MVPLOI@va.gov. The timing and outcome of formal access review are determined by the MVP Program Office. The full summary level genome-wide association analyses results will be available through dbGaP with accession number phs001672. Individual-level BioVU data are also subject to controlled access to protect participant privacy and to comply with Vanderbilt University Medical Center institutional and ethical requirements. Access requires an Institutional Review Board-approved protocol. Requests and questions regarding access should be directed to biovu@vumc.org. Formal review and data-provision timelines are determined by BioVU. Previously generated summary statistics used in this study were obtained from the following sources: the HERMES all-cause heart failure GWAS through the Cardiovascular Disease Knowledge Portal (http://www.broadcvdi.org/); FinnGen all-cause heart failure GWAS results through the FinnGen results portal (https://www.finngen.fi/en/access_results); the multi-ancestry all-cause heart failure GWAS of Levin et al. through the GWAS Catalog (GCST90162626) and Zenodo; and the BioBank Japan heart failure GWAS of Enzan et al. through the GWAS Catalog (GCST90668009 for all-cause heart failure, GCST90668010 for HFrEF, and GCST90668011 for HFpEF). These summary-level datasets are available under the access and use terms established by their respective data providers. Publicly accessible reference and functional datasets used in the analyses included the 1000 Genomes Project Phase 3 reference panels (https://www.internationalgenome.org/data-portal/data-collection/phase3/); GTEx version 8 gene-expression and expression quantitative trait locus data (https://gtexportal.org/home/); and Fenland proteogenomic protein quantitative trait locus summary data (https://www.omicscience.org/apps/pgwas). Functional annotation resources included the EnhancerAtlas 2.0 (http://www.singlecelldb.com/), ENCODE data (https://www.encodeproject.org/). Therapeutic-target and drug-target information was obtained from the Open Targets Platform (https://platform.opentargets.org/) and DrugBank 6.0 (https://go.drugbank.com/). Complete study-generated downstream results, including study and ancestry-specific quality-control information, genome-wide significant loci, heterogeneity analyses, genomic inflation, and LD-score regression results, PheWAS results, gene-based and enrichment analyses, pQTL and enhancer lookups, TWAS and colocalization results, PoPS gene-prioritization results, chromatin-state annotations, credible sets and fine-mapping results, therapeutic-target evaluations, and Mendelian randomization results, are provided in Supplementary Data 1–25. All other data supporting the findings of this study are available within the article and its Supplementary Information files.
