# Supplementary material for: Multi-ancestry genetic architecture of heart failure subtypes
Source: Nat Commun. 2026 Aug 13;17:9730. doi: 10.1038/s41467-026-76438-y (PMC13562571; doi:10.1038/s41467-026-76438-y)
Supplement: Supplementary file 1 [file 41467_2026_76438_MOESM1_ESM.pdf]

Supplementary Information

Multi-ancestry Genetic Architecture of Heart Failure Subtypes

Supplementary Figure 1. Genome-wide Association Study (GWAS) Sample and Workflow.

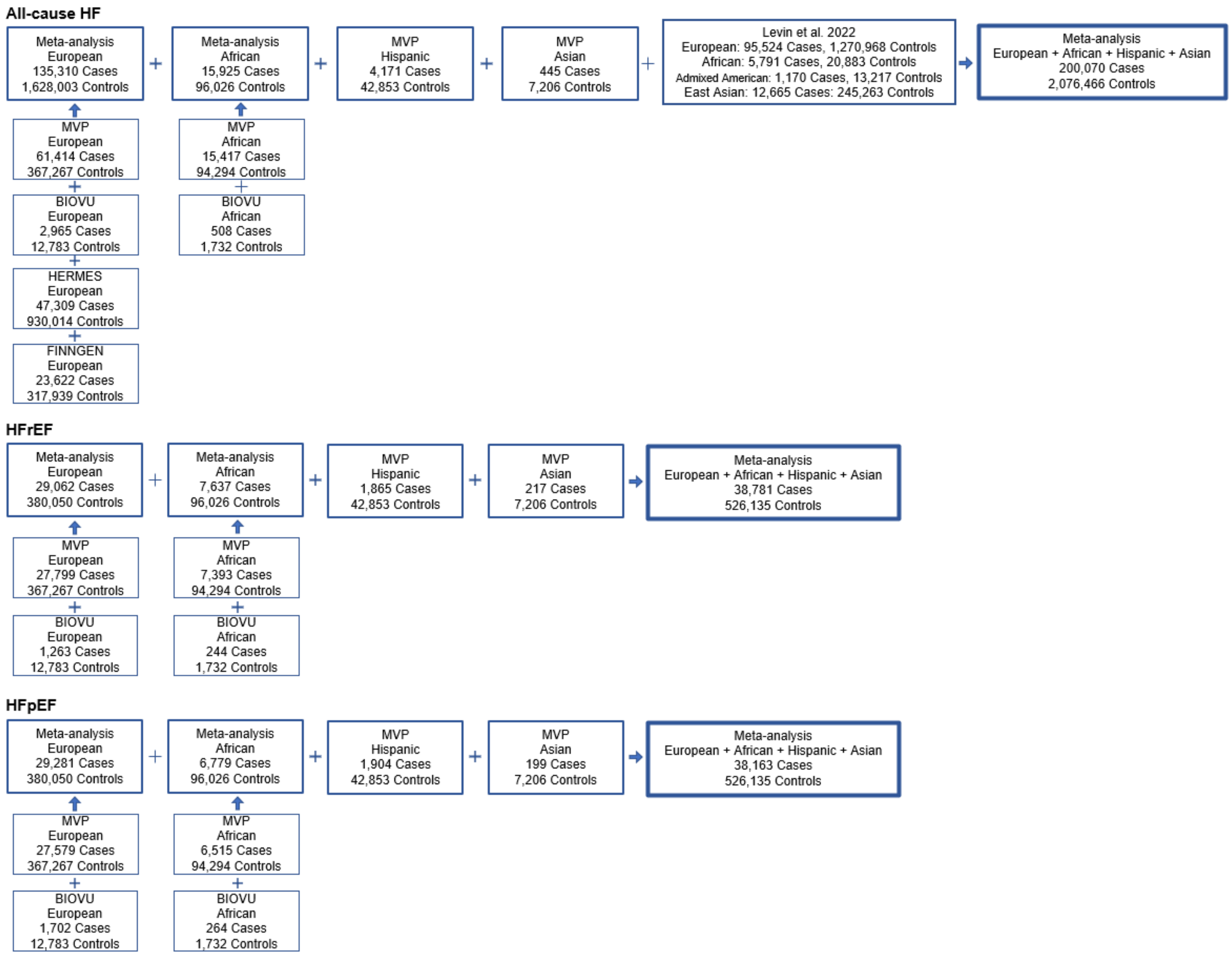

HF: Heart Failure; HFrEF: HF with reduced ejection fraction; HFpEF: HF with preserved ejection fraction.

Supplementary Figure 2. Manhattan Plots for the Meta-analysis of GWAS on HFpEF and TWAS.

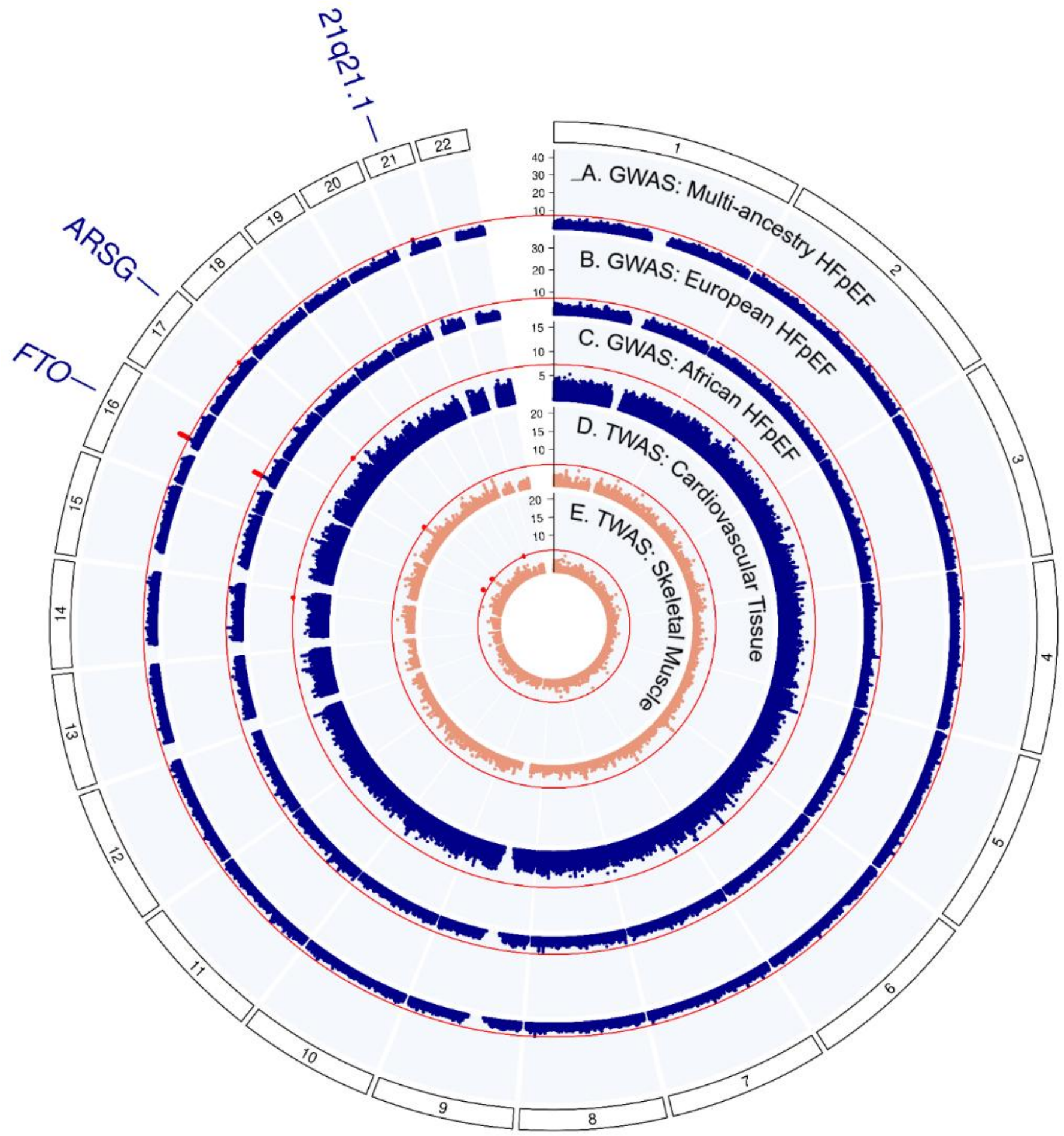

Track A: Manhattan plot of the multi-ancestry GWAS meta-analysis of HFpEF. Track B: Manhattan plot of the European-ancestry GWAS meta-analysis of HFpEF. Track C: Manhattan plot of the African-ancestry GWAS meta-analysis of HFpEF. Track D: Manhattan plot of the TWAS of HFpEF in cardiovascular tissues. Track E: Manhattan plot of the TWAS of HFpEF in skeletal muscle. Genome-wide significant HFpEF loci are labeled. Italic font indicates significant enrichment in skeletal muscle.

Supplementary Figure 3. Manhattan Plot for the Meta-analysis of GWAS on All-cause HF Among Multi-ancestry Cohort.

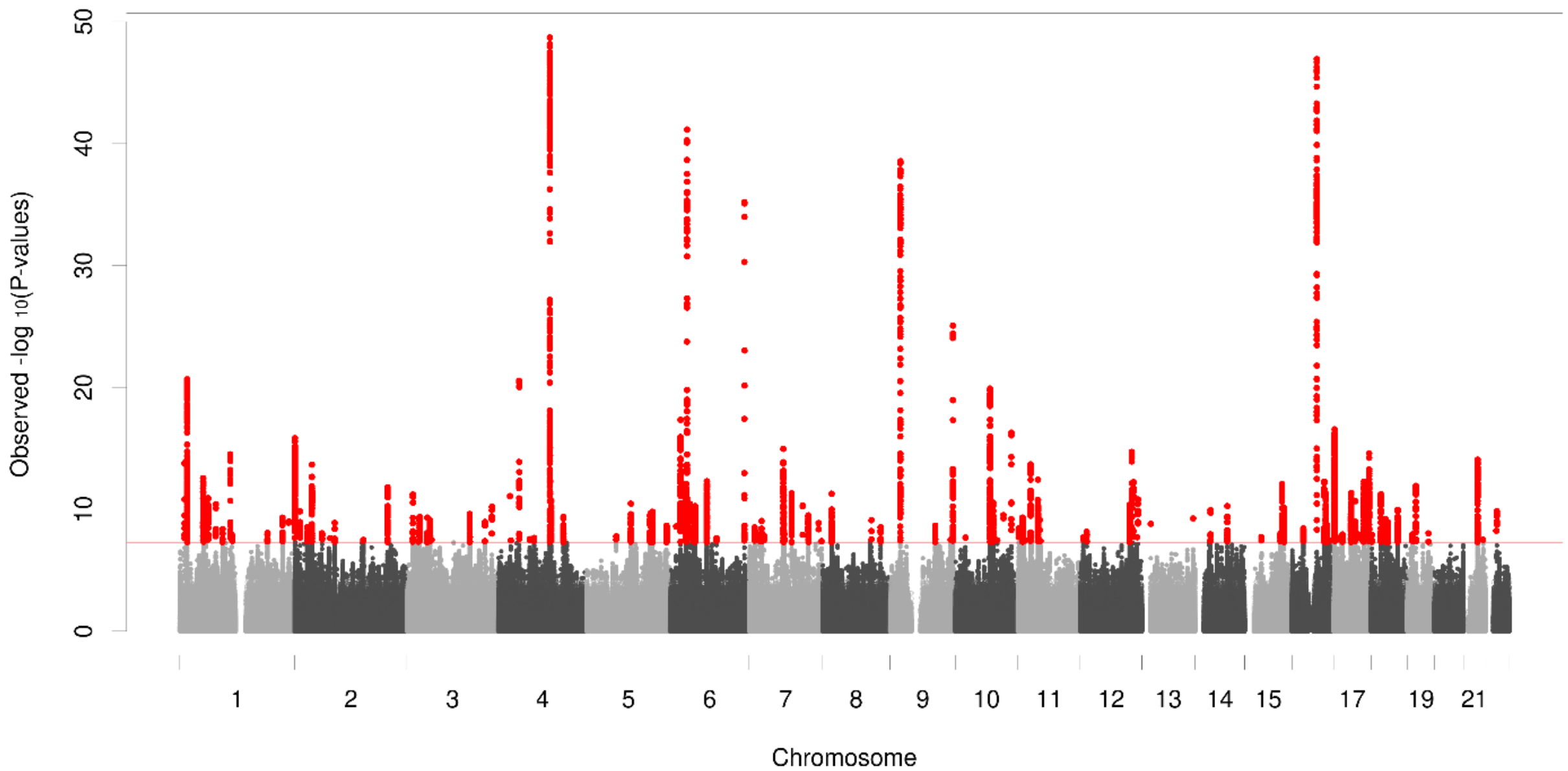

Supplementary Figure 4. Venn Diagram for Genome-wide Significant Loci Shared or Exclusive to All-cause HF, HFrEF, and HFpEF.

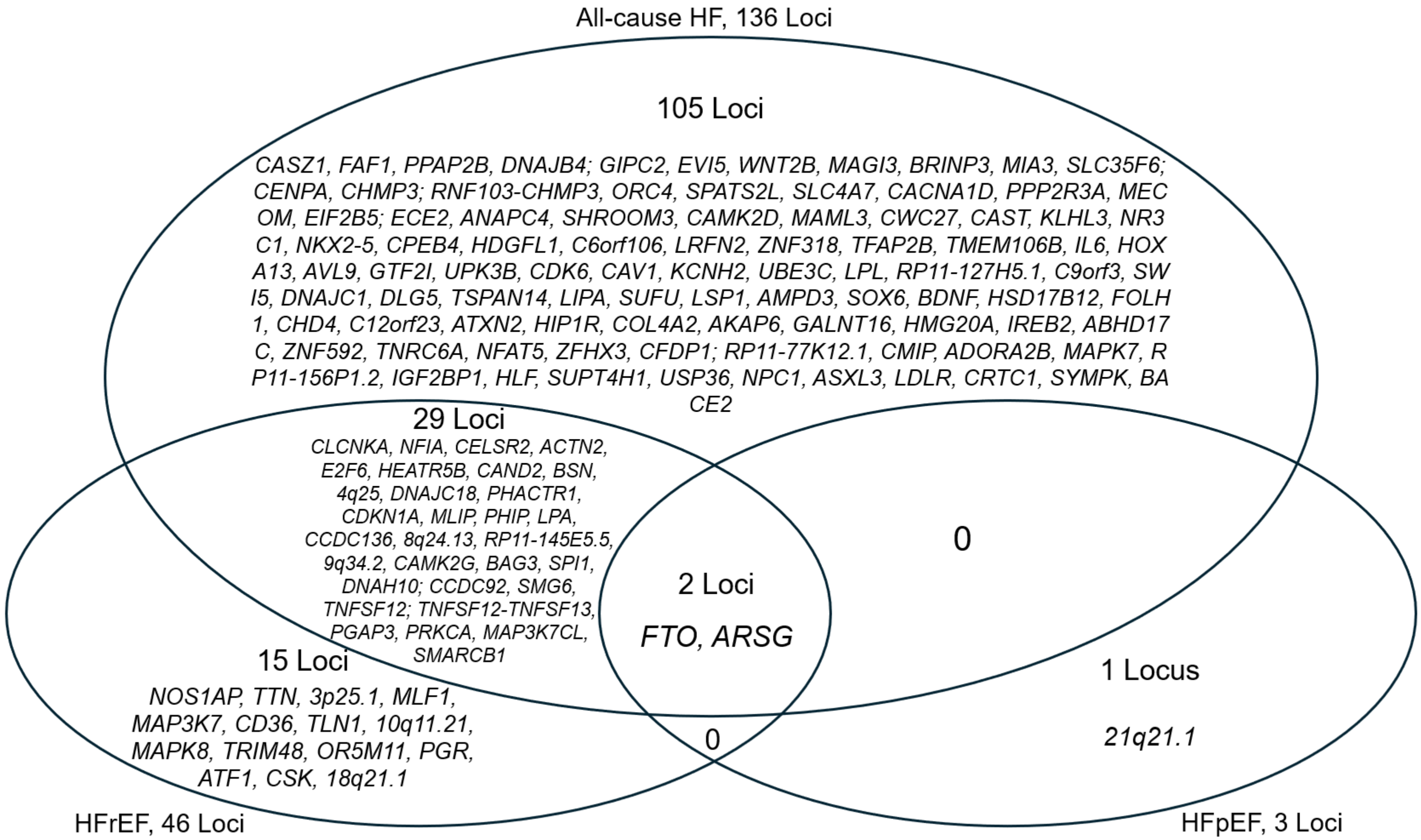

Supplementary Figure 5. Scatter Plot for Comparison of The Estimates for the Genome-wide Significant Loci for the Meta-analysis of GWAS on HFrEF and HFpEF Among European Ancestry.

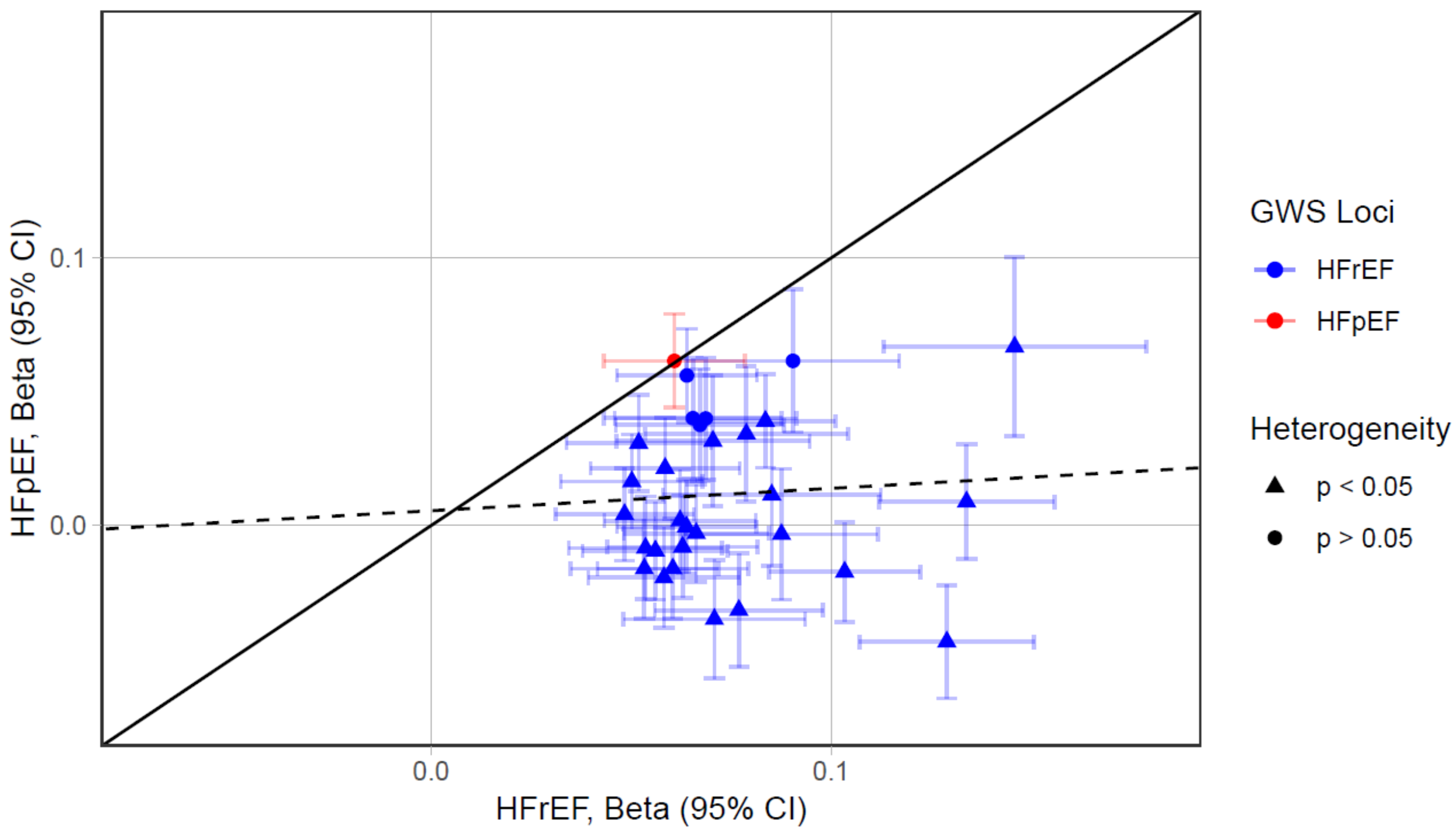

Horizontal and vertical error bars indicate 95% confidence intervals. Analyses included 29,062 HFrEF cases, 29,281 HFpEF cases, and 380,050 controls.

Supplementary Figure 6. Scatter Plot for Comparison of The Estimates for the Genome-wide Significant Loci for the Meta-analysis of GWAS on HFrEF and HFpEF Among African Ancestry.

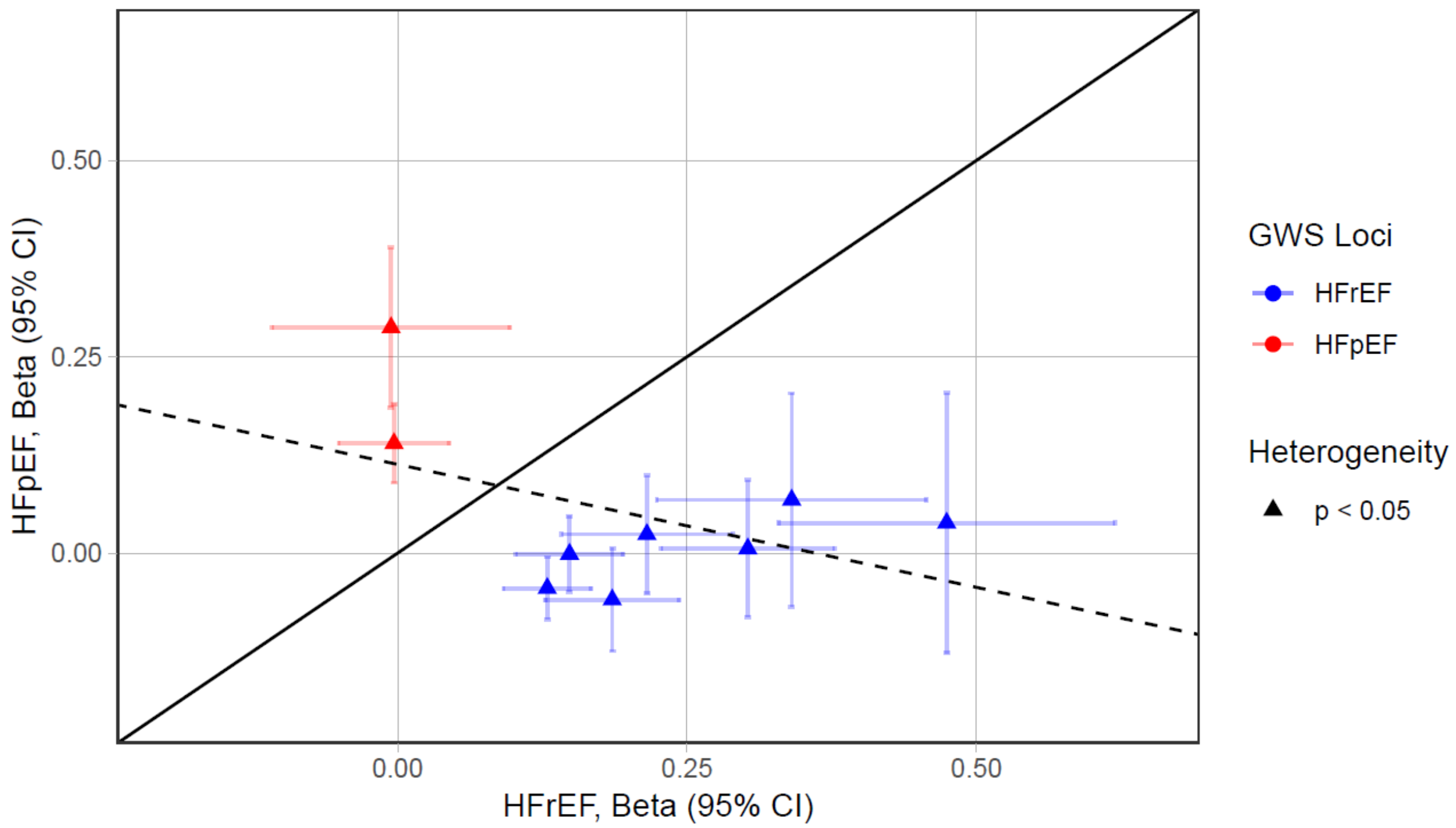

Horizontal and vertical error bars indicate 95% confidence intervals. Analyses included 7,637 HFrEF cases, 6,779 HFpEF cases, and 96,026 controls.

Supplementary Figure 7. Phenome-wide Associations of Heart Failure Loci in Participants of African Ancestry.

A.

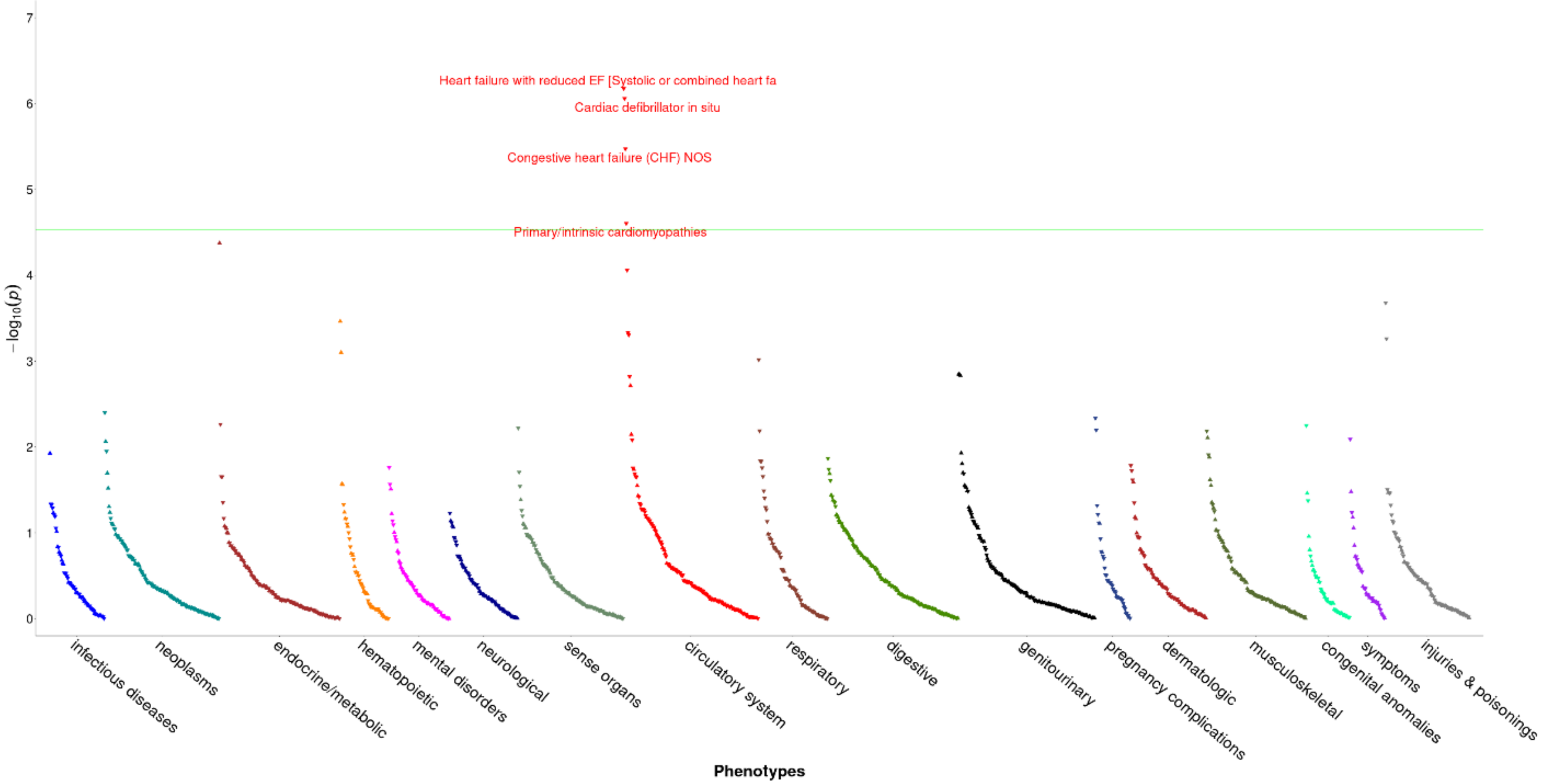

B.

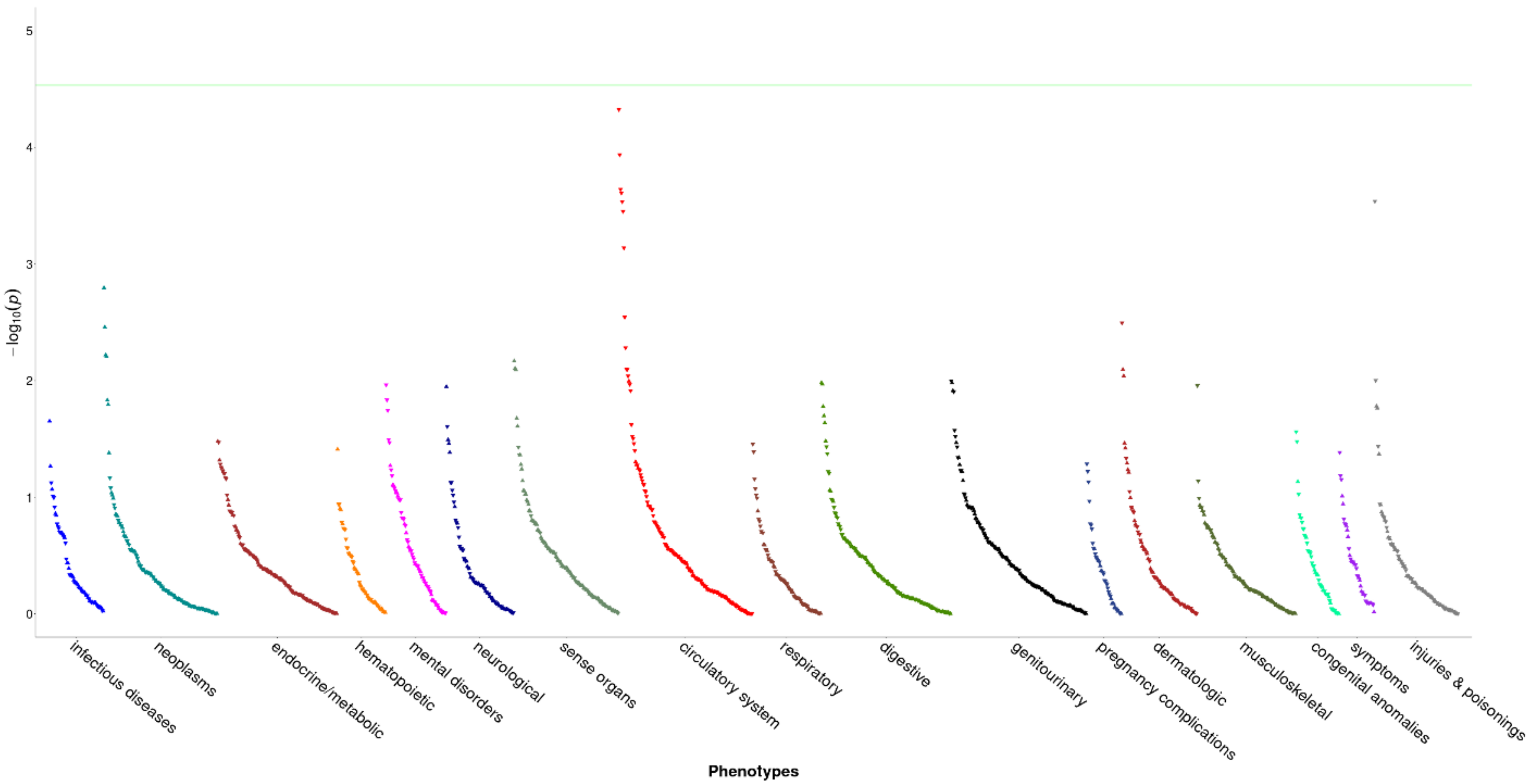

5
4
3
2
1
0
$-\log_{10}(p)$
infectious diseases
neoplasms
endocrine/metabolic
hematopoietic
mental disorders
neurological
sense organs
circulatory system
respiratory
digestive
genitourinary
pregnancy complications
dermatologic
musculoskeletal
congenital anomalies
symptoms
injuries & poisonings
Phenotypes

C.

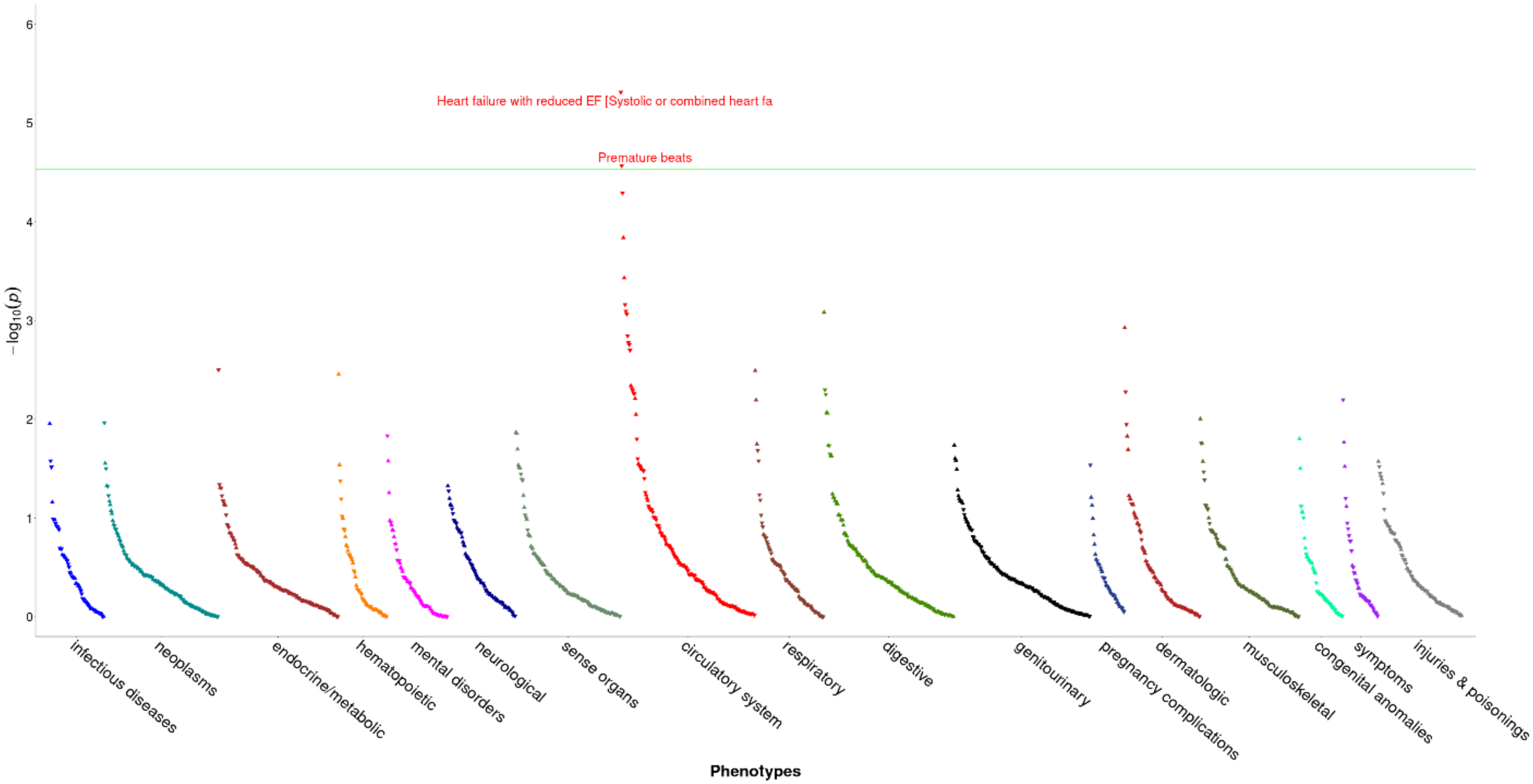

Heart failure with reduced EF [Systolic or combined heart fa
Premature beats
$-\log_{10}(p)$
0
1
2
3
4
5
6
infectious diseases
neoplasms
endocrine/metabolic
hematopoietic
mental disorders
neurological
sense organs
circulatory system
respiratory
digestive
genitourinary
pregnancy complications
dermatologic
musculoskeletal
congenital anomalies
symptoms
injuries & poisonings
Phenotypes

D.

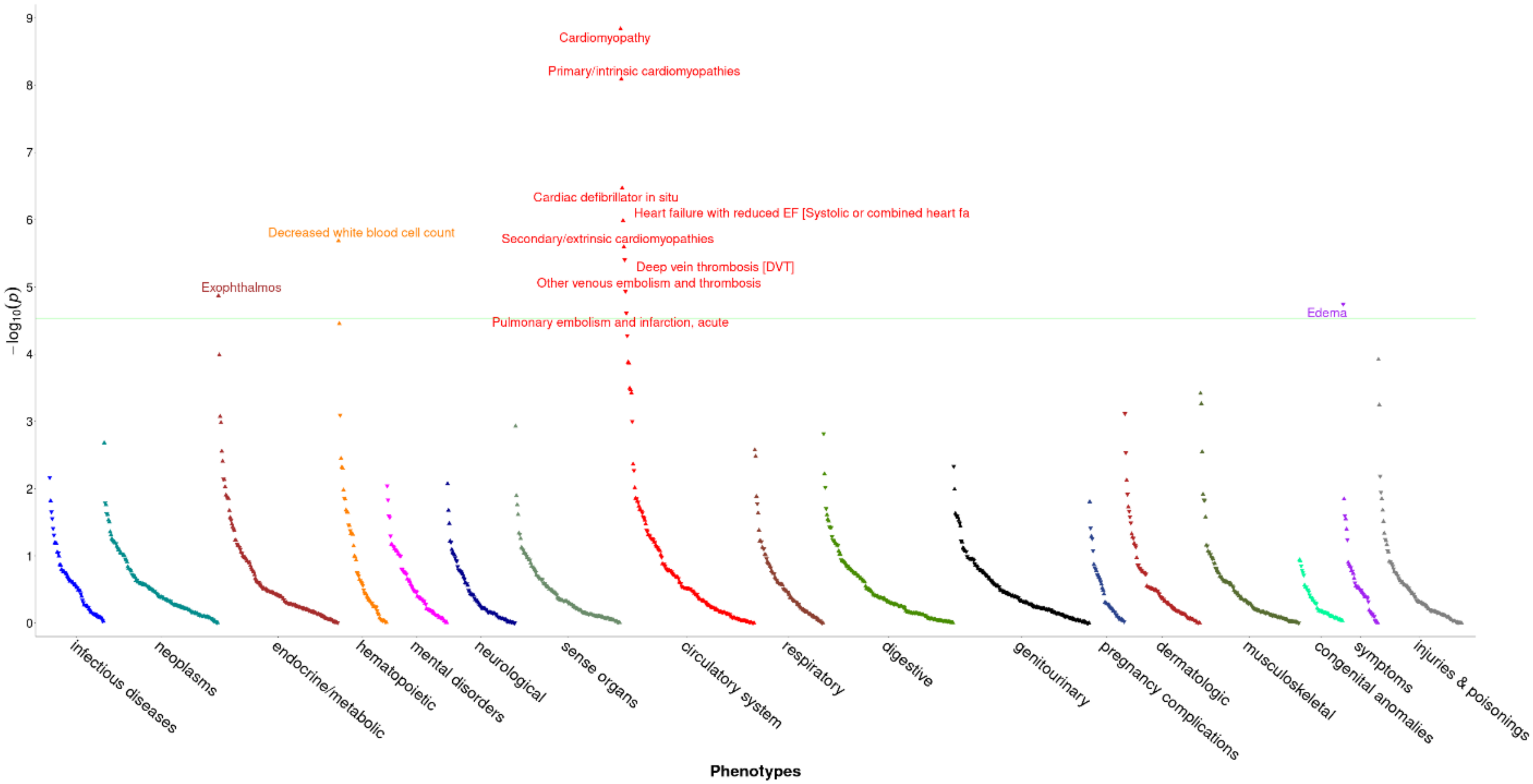
Cardiomyopathy
Primary/intrinsic cardiomyopathies
Cardiac defibrillator in situ
Heart failure with reduced EF [Systolic or combined heart fa
Decreased white blood cell count
Secondary/extrinsic cardiomyopathies
Deep vein thrombosis [DVT]
Exophthalmos
Other venous embolism and thrombosis
Edema
Pulmonary embolism and infarction, acute
$-\log_{10}(p)$
9
8
7
6
5
4
3
2
1
0
infectious diseases
neoplasms
endocrine/metabolic
hematopoietic
mental disorders
neurological
sense organs
circulatory system
respiratory
digestive
genitourinary
pregnancy complications
dermatologic
musculoskeletal
congenital anomalies
symptoms
injuries & poisonings
Phenotypes

E.

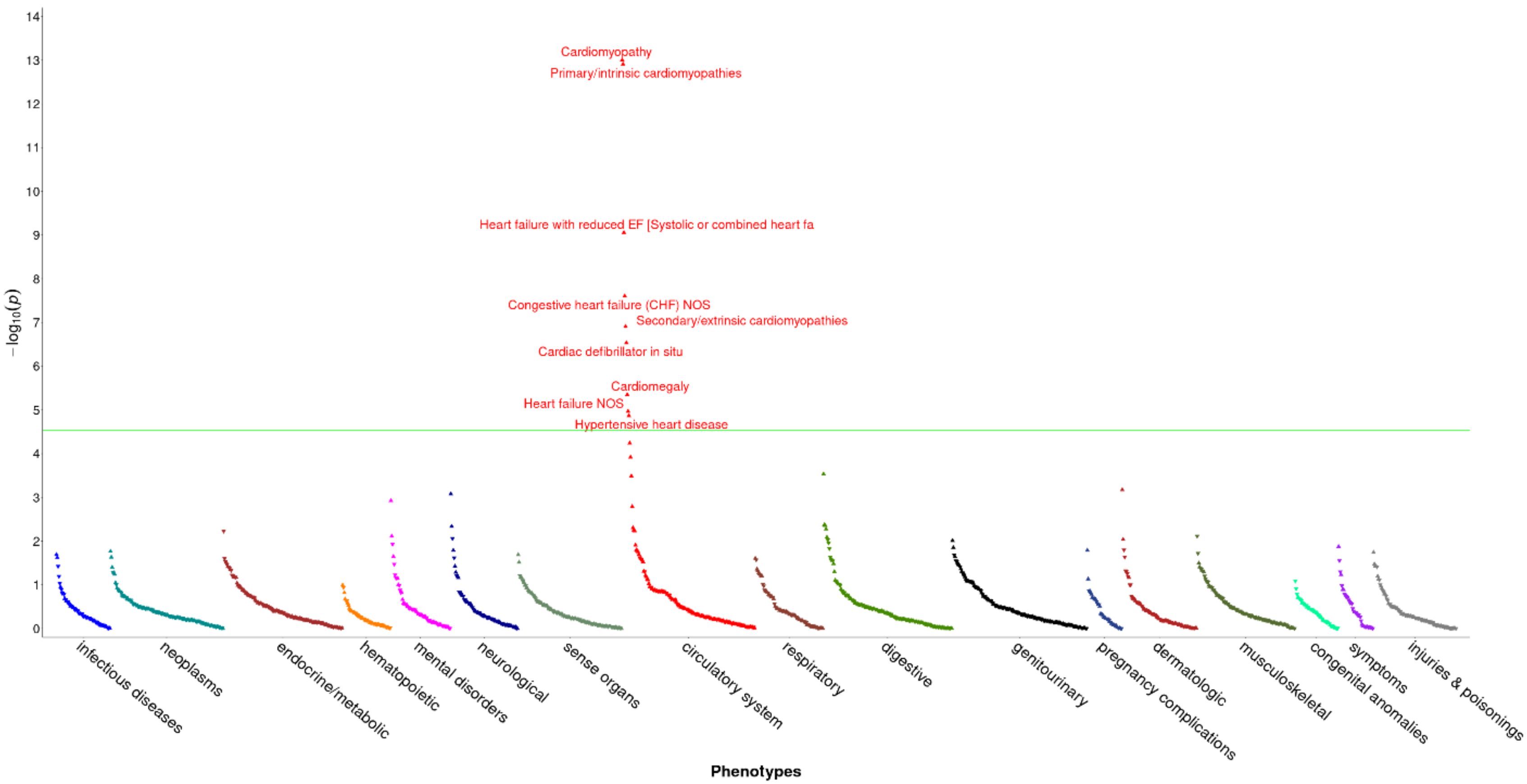

Cardiomyopathy
Primary/intrinsic cardiomyopathies
Heart failure with reduced EF [Systolic or combined heart fa
Congestive heart failure (CHF) NOS
Secondary/extrinsic cardiomyopathies
Cardiac defibrillator in situ
Cardiomegaly
Heart failure NOS
Hypertensive heart disease
−log10(p)
0
1
2
3
4
5
6
7
8
9
10
11
12
13
14
infectious diseases
neoplasms
endocrine/metabolic
hematopoietic
mental disorders
neurological
sense organs
circulatory system
respiratory
digestive
genitourinary
pregnancy complications
dermatologic
musculoskeletal
congenital anomalies
symptoms
injuries & poisonings
Phenotypes

F.

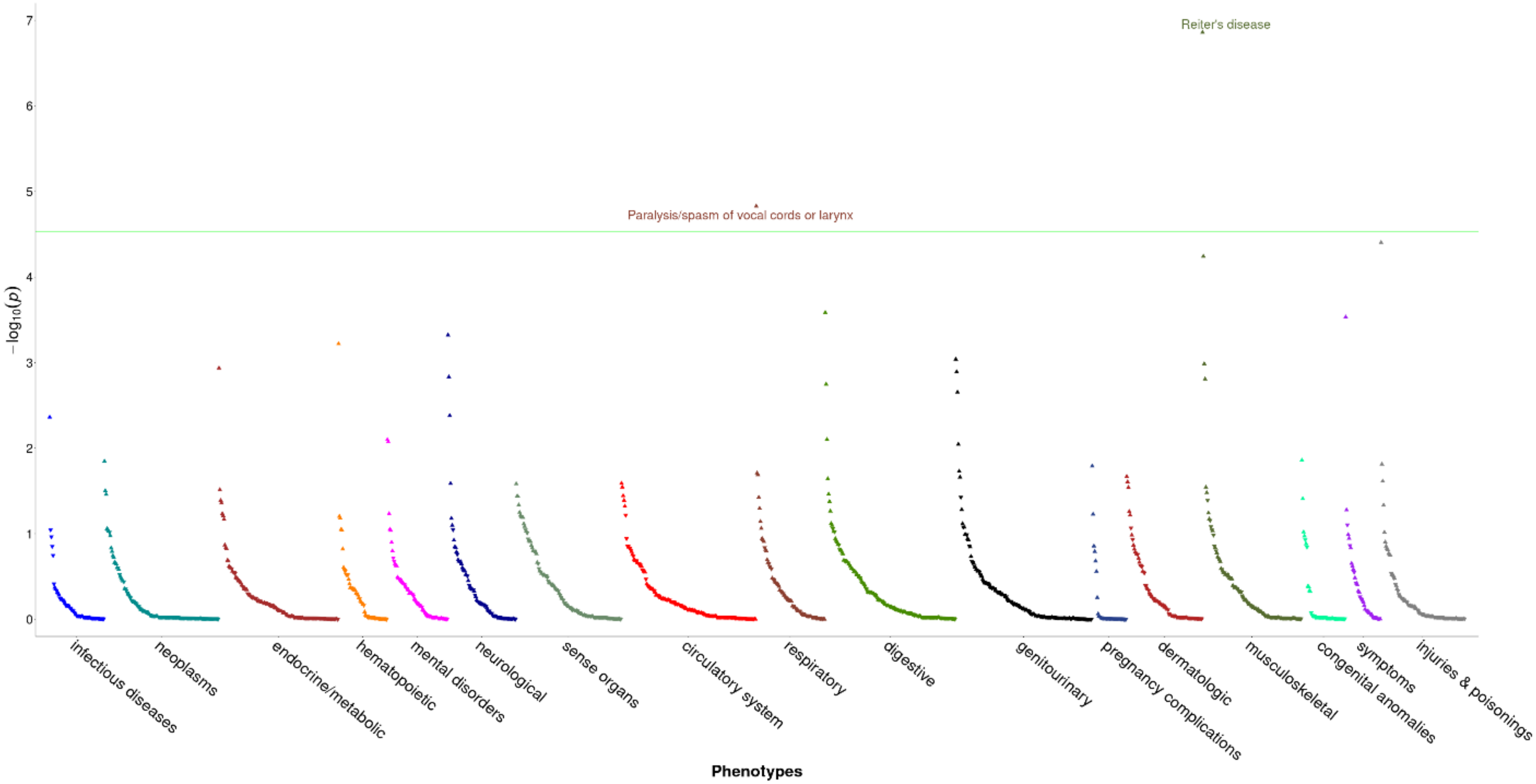

Reiter's disease
Paralysis/spasm of vocal cords or larynx
$-\log_{10}(p)$
0
1
2
3
4
5
6
7
infectious diseases
neoplasms
endocrine/metabolic
hematopoietic
mental disorders
neurological
sense organs
circulatory system
respiratory
digestive
genitourinary
pregnancy complications
dermatologic
musculoskeletal
congenital anomalies
symptoms
injuries & poisonings
Phenotypes

G.

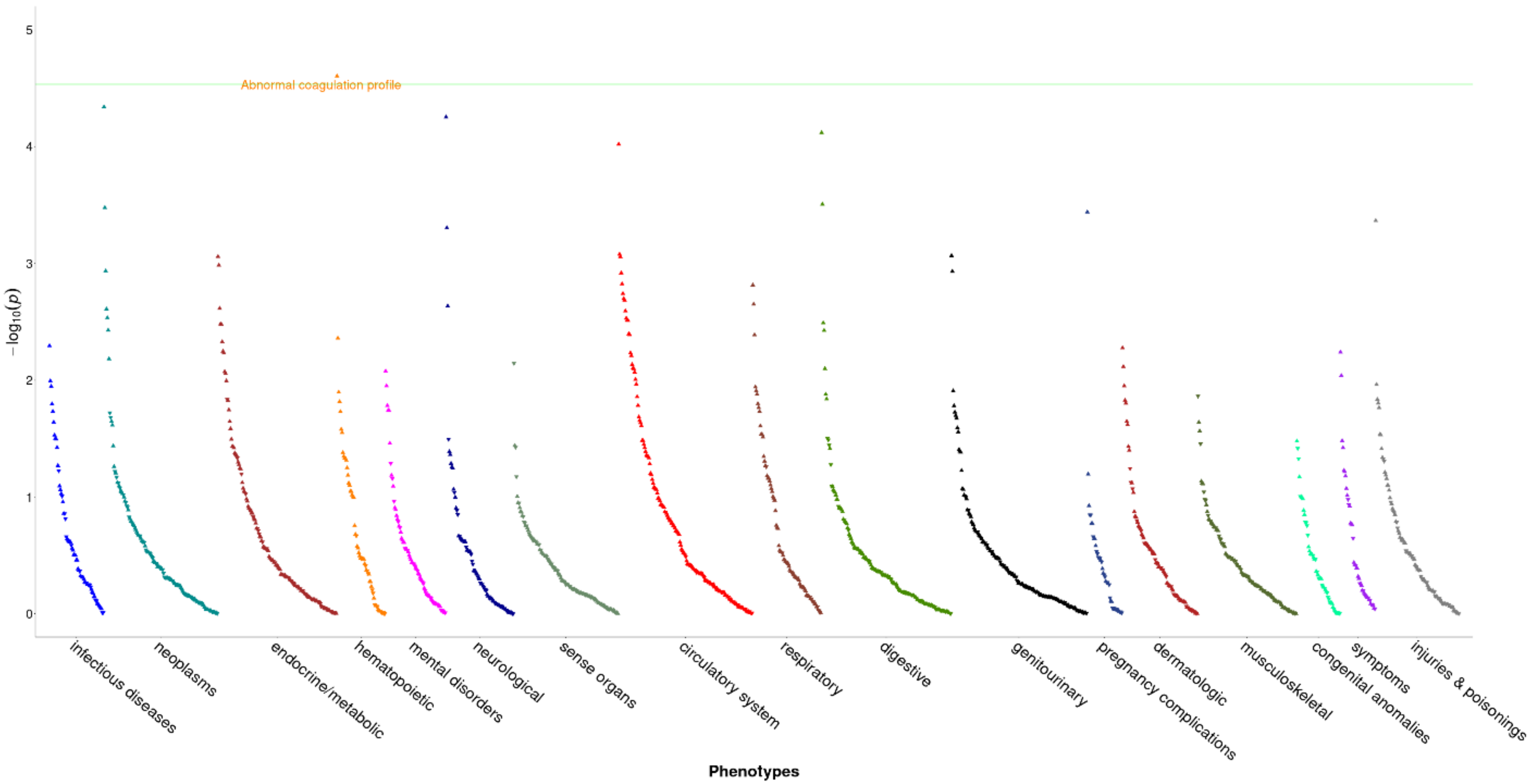

Abnormal coagulation profile
5
4
3
2
1
0
$-\log_{10}(p)$
infectious diseases
neoplasms
endocrine/metabolic
hematopoietic
mental disorders
neurological
sense organs
circulatory system
respiratory
digestive
genitourinary
pregnancy complications
dermatologic
musculoskeletal
congenital anomalies
symptoms
injuries & poisonings
Phenotypes

H.

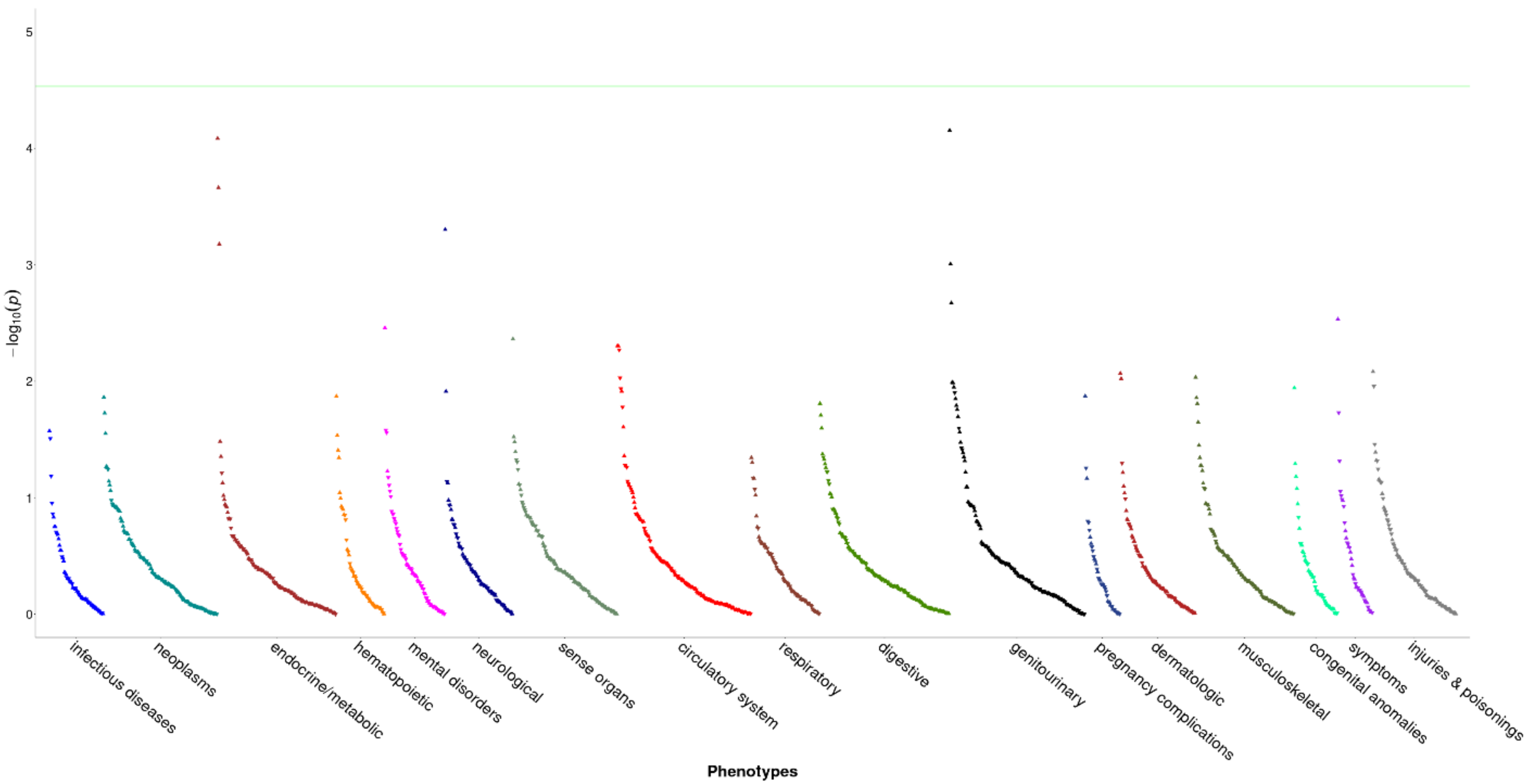

5
4
3
2
1
0
−log10(p)
infectious diseases
neoplasms
endocrine/metabolic
hematopoietic
mental disorders
neurological
sense organs
circulatory system
respiratory
digestive
genitourinary
pregnancy complications
dermatologic
musculoskeletal
congenital anomalies
symptoms
injuries & poisonings
Phenotypes

I.

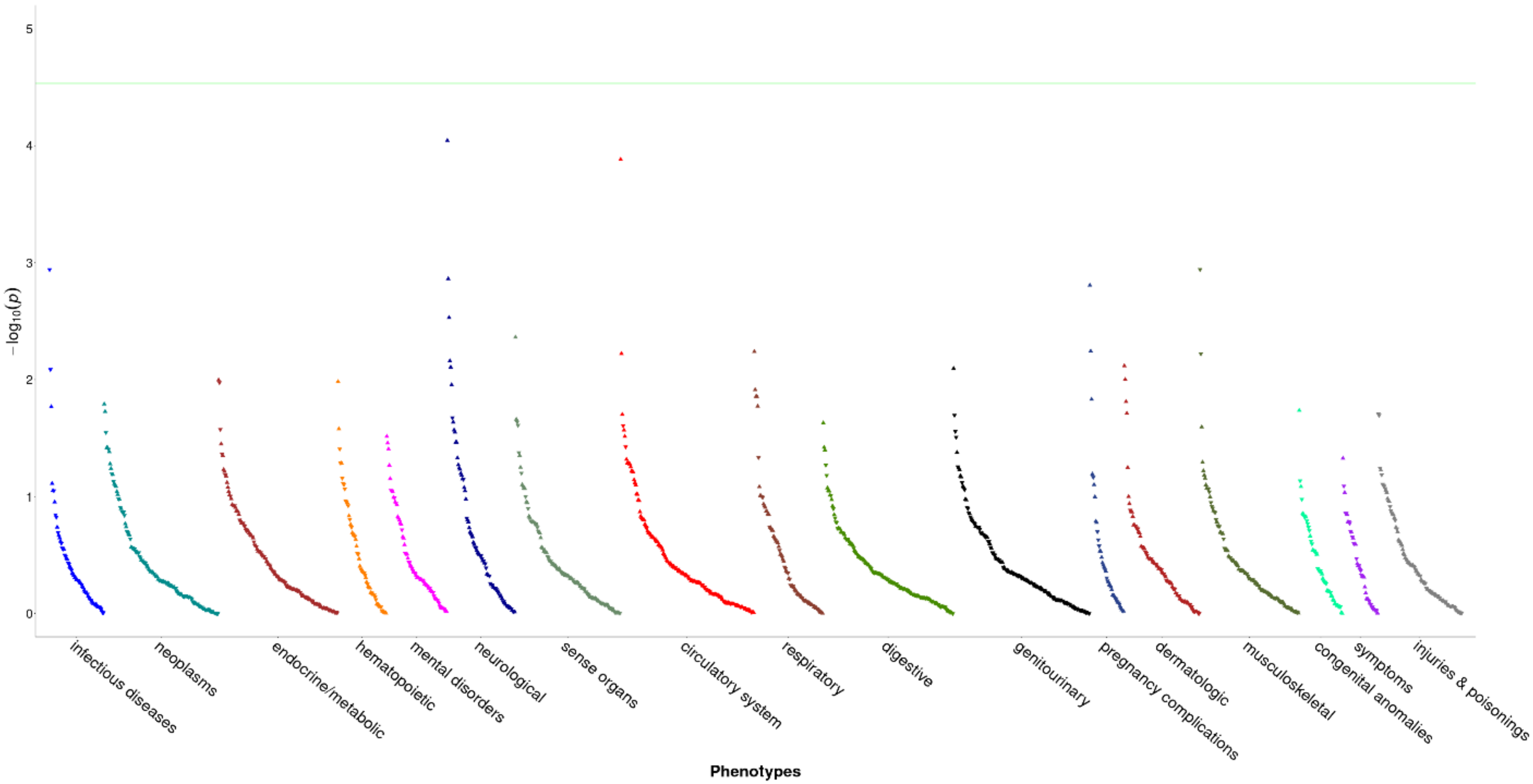

5
4
3
2
1
0
$-\log_{10}(p)$
infectious diseases
neoplasms
endocrine/metabolic
hematopoietic
mental disorders
neurological
sense organs
circulatory system
respiratory
digestive
genitourinary
pregnancy complications
dermatologic
musculoskeletal
congenital anomalies
symptoms
injuries & poisonings
Phenotypes

J.

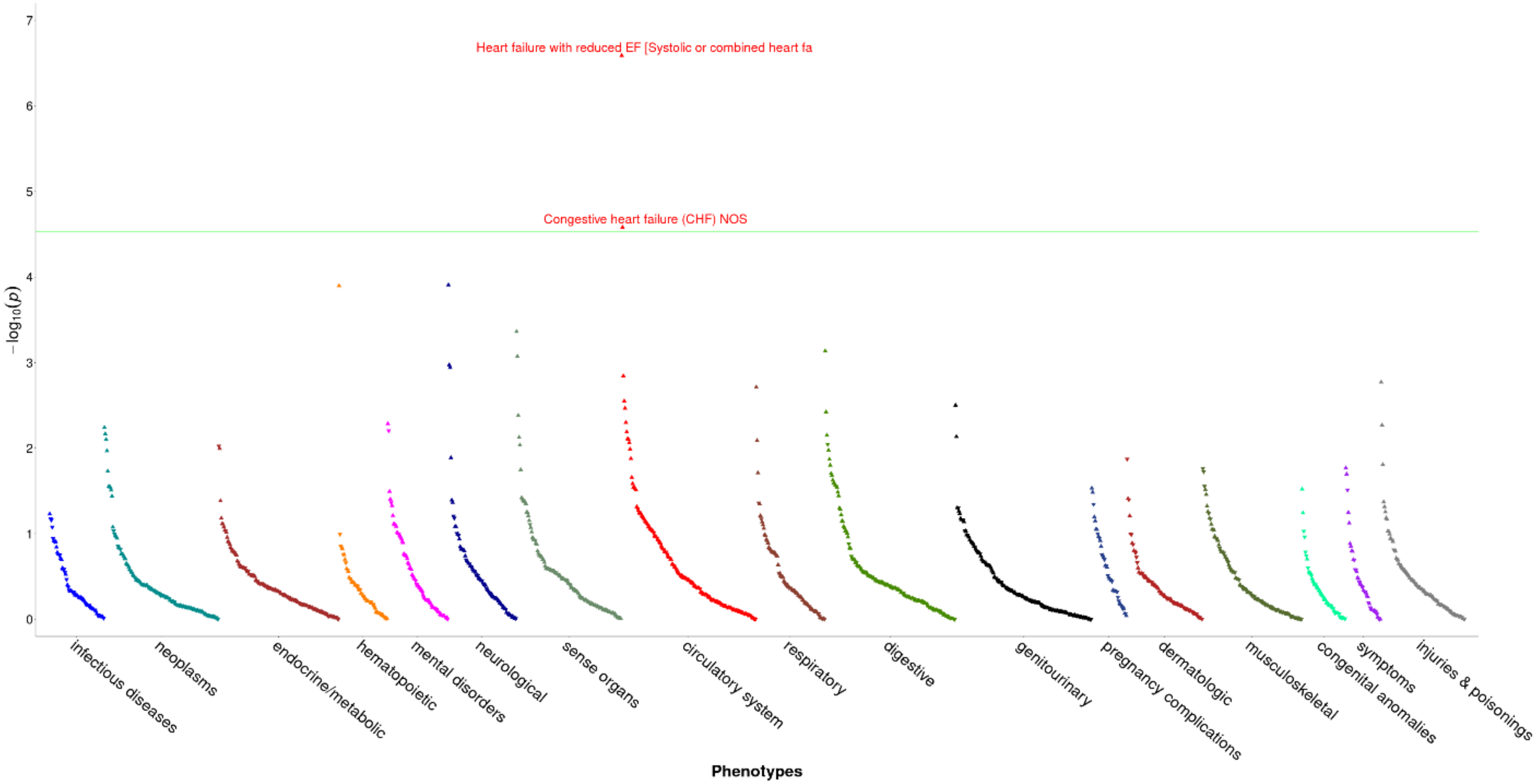

Heart failure with reduced EF [Systolic or combined heart fa
Congestive heart failure (CHF) NOS
7
6
5
4
3
2
1
0
−log10(p)
infectious diseases
neoplasms
endocrine/metabolic
hematopoietic
mental disorders
neurological
sense organs
circulatory system
respiratory
digestive
genitourinary
pregnancy complications
dermatologic
musculoskeletal
congenital anomalies
symptoms
injuries & poisonings
Phenotypes

K.

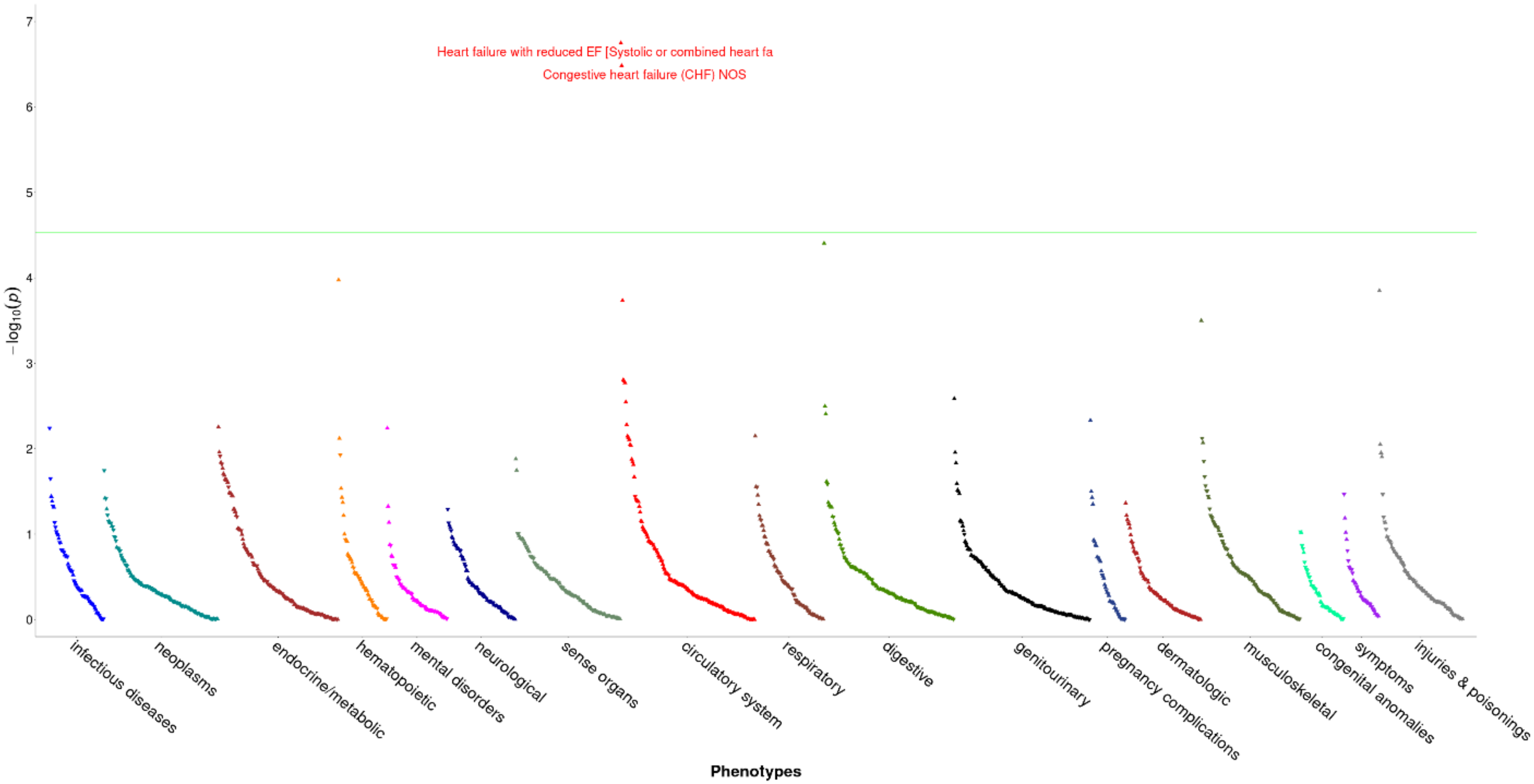

Heart failure with reduced EF [Systolic or combined heart fa
Congestive heart failure (CHF) NOS
$-\log_{10}(p)$
7
6
5
4
3
2
1
0
infectious diseases
neoplasms
endocrine/metabolic
hematopoietic
mental disorders
neurological
sense organs
circulatory system
respiratory
digestive
genitourinary
pregnancy complications
dermatologic
musculoskeletal
congenital anomalies
symptoms
injuries & poisonings
Phenotypes

L.

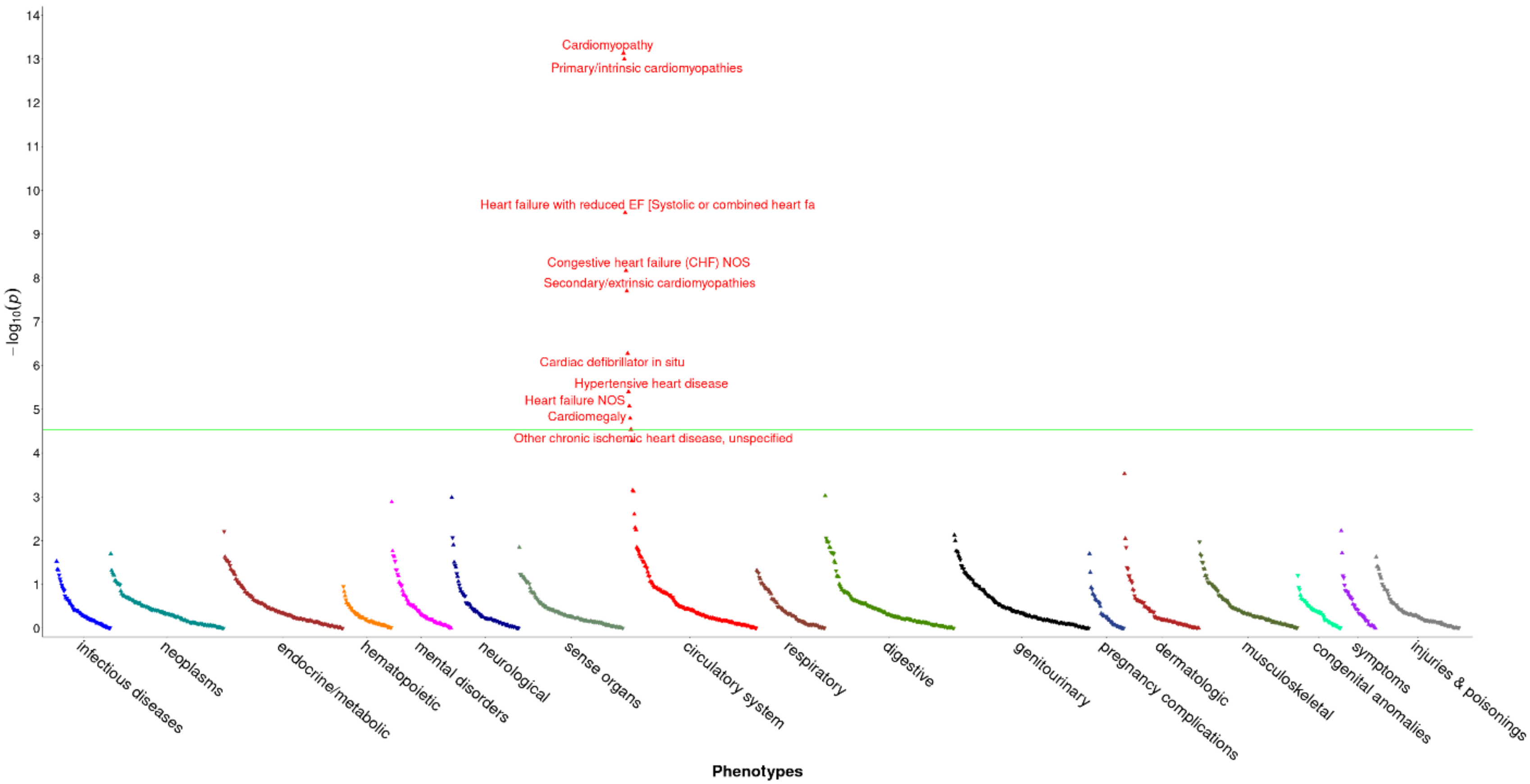

Panels show associations for HFrEF loci *HSPB7* rs1572381 (A), *CCDC141* rs7564036 (B), *CDKN1A* rs3176326 (C), *CD36* rs3211938 (D), *SPI1* rs113123267 (E), *TRIM48* rs111786504 (F), and *SPNS3* rs75531264 (G); HFpEF loci *14q32.13* rs12586269 (H) and *ARSG* rs75716431 (I); and all-cause HF loci *GUCA1C* rs2593932 (J), *AVL9* rs115698376 (K), and *SPI1* rs73453764 (L). The y axis shows -log10 (two-sided P value), and phenotypes are grouped by clinical category along the x axis. The horizontal green line indicates the false discovery rate significance threshold of q < 0.05.

VA Million Veteran Program
Core Acknowledgements for Publications
October 2025

MVP Program Office

- Sumitra Muralidhar, Ph.D., Program Director
US Department of Veterans Affairs, 810 Vermont Avenue NW, Washington, DC 20420
- Jennifer Moser, Ph.D., Associate Director, Scientific Programs
US Department of Veterans Affairs, 810 Vermont Avenue NW, Washington, DC 20420
- Jennifer E. Deen, B.S., Associate Director, Cohort & Public Relations
US Department of Veterans Affairs, 810 Vermont Avenue NW, Washington, DC 20420

MVP Steering Committee

- Co-Chair: Philip S. Tsao, Ph.D.
VA Palo Alto Health Care System, 3801 Miranda Avenue, Palo Alto, CA 94304
- Co-Chair: Sumitra Muralidhar, Ph.D.
US Department of Veterans Affairs, 810 Vermont Avenue NW, Washington, DC 20420
- J. Michael Gaziano, M.D., M.P.H.
VA Boston Healthcare System, 150 S. Huntington Avenue, Boston, MA 02130
- Adriana Hung, M.D., M.P.H.,
VA Tennessee Valley Healthcare System, 1310 24th Avenue, South Nashville, TN 37212
- Dave Oslin, M.D.
Philadelphia VA Medical Center, 3900 Woodland Avenue, Philadelphia, PA 19104
- Deepak Voora, M.D.
Durham VA Medical Center, 508 Fulton Street, Durham, NC 27705

MVP Co-Principal Investigators

- J. Michael Gaziano, M.D., M.P.H.
VA Boston Healthcare System, 150 S. Huntington Avenue, Boston, MA 02130
- Philip S. Tsao, Ph.D.
VA Palo Alto Health Care System, 3801 Miranda Avenue, Palo Alto, CA 94304

MVP Core Operations

- Jessica V. Brewer, M.P.H., Director, MVP Cohort Operations
VA Boston Healthcare System, 150 S. Huntington Avenue, Boston, MA 02130
- Mary T. Brophy M.D., M.P.H., Director, VA Central Biorepository
VA Boston Healthcare System, 150 S. Huntington Avenue, Boston, MA 02130
- Kelly Cho, M.P.H, Ph.D., Director, MVP Phenomics
VA Boston Healthcare System, 150 S. Huntington Avenue, Boston, MA 02130
- Lori Churby, B.S., Director, MVP Regulatory Affairs
VA Palo Alto Health Care System, 3801 Miranda Avenue, Palo Alto, CA 94304
- Jacob T. Kean, Ph.D., Acting Director, VA Informatics and Computing Infrastructure (VINCI)
VA Salt Lake City Health Care System, 500 Foothill Drive, Salt Lake City, UT 84148
- Saiju Pyarajan Ph.D., Director, Data and Computational Sciences
VA Boston Healthcare System, 150 S. Huntington Avenue, Boston, MA 02130
- Robert Ringer, Pharm.D., Director, VA Albuquerque Central Biorepository
New Mexico VA Health Care System, 1501 San Pedro Drive SE, Albuquerque, NM 87108
- Luis E. Selva, Ph.D., Director, MVP Biorepository Coordination

VA Boston Healthcare System, 150 S. Huntington Avenue, Boston, MA 02130
- Shahpoor (Alex) Shayan, M.S., Director, MVP PRE Informatics
VA Boston Healthcare System, 150 S. Huntington Avenue, Boston, MA 02130
- Brady Stephens, M.S., Principal Investigator, MVP Information Center
Canandaigua VA Medical Center, 400 Fort Hill Avenue, Canandaigua, NY 14424
- Stacey B. Whitbourne, Ph.D., Director, MVP Cohort Development and Management
VA Boston Healthcare System, 150 S. Huntington Avenue, Boston, MA 02130
