# Supplementary material for: Multi-ancestry genetic architecture of heart failure subtypes
Source: Nat Commun. 2026 Aug 13;17:9730. doi: 10.1038/s41467-026-76438-y (PMC13562571; doi:10.1038/s41467-026-76438-y)
Supplement: Supplementary file 2 [file 41467_2026_76438_MOESM2_ESM.pdf]

Description of Additional Supplementary Files

Supplementary Data 1A: Sample sizes and quality-control parameters for the study cohorts.

Supplementary Data 1B: Sample sizes for ancestry-specific and multi-ancestry meta-analyses of all-cause heart failure (HF), HF with reduced ejection fraction (HFrEF), and HF with preserved ejection fraction (HFpEF).

Supplementary Data 2: Genome-wide significant loci from the multi-ancestry genome-wide association study (GWAS) meta-analyses of HFrEF and HFpEF, including population-specific results and Biobank Japan replication.

Supplementary Data 3: The 136 genome-wide significant loci from the multi-ancestry GWAS meta-analysis of all-cause HF, including population-specific results and Biobank Japan replication.

Supplementary Data 4: Genome-wide significant loci from the European-ancestry GWAS meta-analyses of HFrEF and HFpEF.

Supplementary Data 5: Genome-wide significant loci from the African-ancestry GWAS meta-analyses of HFrEF and HFpEF.

Supplementary Data 6: The 102 genome-wide significant loci from the European-ancestry GWAS meta-analysis of all-cause HF.

Supplementary Data 7: The three genome-wide significant loci from the African-ancestry GWAS meta-analysis of all-cause HF.

Supplementary Data 8: Comparison of effect estimates for ancestry-specific genome-wide significant loci across HFrEF and HFpEF.

Supplementary Data 9: Genomic inflation factors and linkage disequilibrium score regression results by ancestry and study.

Supplementary Data 10: Genomic inflation factors and linkage disequilibrium score regression results for the GWAS meta-analyses.

Supplementary Data 11: Phenome-wide association study (PheWAS) associations meeting false discovery rate < 0.05 for African-ancestry loci; no statistically significant associations were identified for HFpEF loci.

Supplementary Data 12A: MAGMA gene-based test results for multi-ancestry HFrEF loci.

Supplementary Data 12B: MAGMA gene-based test results for multi-ancestry HFpEF loci.

Supplementary Data 12C: MAGMA gene-based test results for multi-ancestry all-cause HF loci.

Supplementary Data 13: Tissue-expression analysis across 54 specific tissue types using Genotype-Tissue Expression (GTEx) data for multi-ancestry loci of HFrEF, HFpEF, and all-cause HF.

Supplementary Data 14: Tissue-expression analysis across 30 general tissue types using GTEx data for multi-ancestry loci of HFrEF, HFpEF, and all-cause HF.

Supplementary Data 15A: DEPICT gene-set analysis of genome-wide significant HFrEF loci among participants of European ancestry.

Supplementary Data 15B: DEPICT gene-set analysis of suggestive HFpEF loci among participants of European ancestry.

Supplementary Data 15C: DEPICT gene-set analysis of genome-wide significant all-cause HF loci among participants of European ancestry.

Supplementary Data 16A: DEPICT pathway-enrichment analysis of genome-wide significant HFrEF loci among participants of European ancestry; results with nominal $P < 0.05$ are shown.

Supplementary Data 16B: DEPICT pathway-enrichment analysis of suggestive HFpEF loci among participants of European ancestry.

Supplementary Data 16C: DEPICT pathway-enrichment analysis of genome-wide significant all-cause HF loci among participants of European ancestry; results with nominal P < 0.05 are shown.

Supplementary Data 17A: Protein quantitative trait locus (pQTL) analysis for the 46 genome-wide significant HFrEF loci from the multi-ancestry GWAS meta-analysis; associations meeting Bonferroni-corrected P < 0.05 are shown.

Supplementary Data 17B: pQTL analysis for the three genome-wide significant HFpEF loci from the multi-ancestry GWAS meta-analysis; the top 10 associations are shown.

Supplementary Data 17C: pQTL analysis for the 136 genome-wide significant all-cause HF loci from the multi-ancestry GWAS meta-analysis; associations meeting Bonferroni-corrected P < 0.05 are shown.

Supplementary Data 18A: Overlap of the 46 genome-wide significant HFrEF loci from the multi-ancestry GWAS meta-analysis with enhancer regions.

Supplementary Data 18B: Overlap of the three genome-wide significant HFpEF loci from the multi-ancestry GWAS meta-analysis with enhancer regions.

Supplementary Data 18C: Overlap of the 136 genome-wide significant all-cause HF loci from the multi-ancestry GWAS meta-analysis with enhancer regions.

Supplementary Data 19A: Transcriptome-wide association study (TWAS) and colocalization analysis of HFrEF among participants of European ancestry.

Supplementary Data 19B: TWAS and colocalization analysis of HFpEF among participants of European ancestry.

Supplementary Data 19C: TWAS and colocalization analysis of all-cause HF among participants of European ancestry.

Supplementary Data 20: Top PoPS-prioritized candidate genes for the multi-ancestry GWAS meta-analyses of HFrEF, HFpEF, and all-cause HF; up to 200 genes are shown for each phenotype.

Supplementary Data 21A: Chromatin-state annotations for the 46 genome-wide significant HFrEF loci in the multi-ancestry cohort.

Supplementary Data 21B: Chromatin-state annotations for the three genome-wide significant HFpEF loci in the multi-ancestry cohort.

Supplementary Data 21C: Chromatin-state annotations for the 136 genome-wide significant all-cause HF loci in the multi-ancestry cohort.

Supplementary Data 22A: Candidate causal variants from credible-set analysis of the 46 genome-wide significant HFrEF loci from the multi-ancestry GWAS meta-analysis.

Supplementary Data 22B: Candidate causal variants from credible-set analysis of the three genome-wide significant HFpEF loci from the multi-ancestry GWAS meta-analysis.

Supplementary Data 22C: Candidate causal variants from credible-set analysis of the 136 genome-wide significant all-cause HF loci from the multi-ancestry GWAS meta-analysis.

Supplementary Data 23: Fine-mapping results for genome-wide significant loci from the multi-ancestry GWAS meta-analyses of HFrEF and HFpEF, using a 500-kb window on each side of the lead variant.

Supplementary Data 24A: Target-tractability assessment of TWAS-prioritized genes using Open Targets.

Supplementary Data 24B: Mapping of genome-wide significant HF loci to DrugBank-annotated pharmacologic targets.

Supplementary Data 25A: Mendelian randomization analyses of potential causal effects of risk factors on HFrEF.

Supplementary Data 25B: Mendelian randomization analyses of potential causal effects of risk factors on HFpEF.
