# Supplementary material for: Multi-ancestry genetic architecture of heart failure subtypes
Source: Nat Commun. 2026 Aug 13;17:9730. doi: 10.1038/s41467-026-76438-y (PMC13562571; doi:10.1038/s41467-026-76438-y)
Supplement: Supplementary file 3 [file 41467_2026_76438_MOESM4_ESM.pdf]

Corresponding author(s): Yan V. Sun

Last updated by author(s): 06/23/2026

Reporting Summary

Nature Portfolio wishes to improve the reproducibility of the work that we publish. This form provides structure for consistency and transparency in reporting. For further information on Nature Portfolio policies, see our Editorial Policies and the Editorial Policy Checklist.

Statistics

For all statistical analyses, confirm that the following items are present in the figure legend, table legend, main text, or Methods section.

n/a	Confirmed	
☐	☒	The exact sample size (n) for each experimental group/condition, given as a discrete number and unit of measurement
☐	☒	A statement on whether measurements were taken from distinct samples or whether the same sample was measured repeatedly
☐	☒	The statistical test(s) used AND whether they are one- or two-sided<br>*Only common tests should be described solely by name; describe more complex techniques in the Methods section.*
☐	☒	A description of all covariates tested
☐	☒	A description of any assumptions or corrections, such as tests of normality and adjustment for multiple comparisons
☐	☒	A full description of the statistical parameters including central tendency (e.g. means) or other basic estimates (e.g. regression coefficient) AND variation (e.g. standard deviation) or associated estimates of uncertainty (e.g. confidence intervals)
☐	☒	For null hypothesis testing, the test statistic (e.g. F, t, r) with confidence intervals, effect sizes, degrees of freedom and P value noted<br>*Give P values as exact values whenever suitable.*
☒	☐	For Bayesian analysis, information on the choice of priors and Markov chain Monte Carlo settings
☒	☐	For hierarchical and complex designs, identification of the appropriate level for tests and full reporting of outcomes
☐	☒	Estimates of effect sizes (e.g. Cohen's d, Pearson's r), indicating how they were calculated

Our web collection on statistics for biologists contains articles on many of the points above.

Software and code

Policy information about availability of computer code

Data collection	No software was used for data collection.
Data analysis	Analyses used PLINK, REGENIE, GWAMA, LDSC, GCTA/GREML-LDMS, MAGMA, DEPICT, FUSION, coloc, corrcoverage, LDlinkR, MESuSiE, PoPS, TwoSampleMR, MR-PRESSO, LiftOver, and R. External annotation and target resources included GTEx v8, Fenland pQTL data, EnhancerAtlas 2.0, Roadmap Epigenomics/ENCODE ChromHMM annotations, Open Targets, and DrugBank 6.0. Further details are provided in the Methods section.

For manuscripts utilizing custom algorithms or software that are central to the research but not yet described in published literature, software must be made available to editors and reviewers. We strongly encourage code deposition in a community repository (e.g. GitHub). See the Nature Portfolio guidelines for submitting code & software for further information.

Data

Policy information about availability of data

All manuscripts must include a data availability statement. This statement should provide the following information, where applicable:

- Accession codes, unique identifiers, or web links for publicly available datasets
- A description of any restrictions on data availability
- For clinical datasets or third party data, please ensure that the statement adheres to our policy

Due to US Department of Veterans Affairs (VA) regulations and our ethics agreements, the analytic datasets used for this study are not permitted to leave the

Million Veteran Program (MVP) research environment and VA firewall. This limitation is consistent with other MVP studies based on VA data. However, the MVP data are made available to researchers with an approved VA and MVP study protocol. The full summary level genome-wide association analyses results will be available through dbGaP with accession number phs001672.

Research involving human participants, their data, or biological material

Policy information about studies with human participants or human data. See also policy information about sex, gender (identity/presentation), and sexual orientation and race, ethnicity and racism.

Reporting on sex and gender	Our research focused on the heart failure in both sexes, so we did not perform sex- or gender-based analysis.
Reporting on race, ethnicity, or other socially relevant groupings	The GWAS was stratified by ancestry groups, including European, African, Hispanic and Asian.
Population characteristics	The population characteristics are reported in the Methods - Study Cohorts, Genotyping and Phenotyping section.
Recruitment	The Million Veteran Program (MVP) cohort: The MVP is a large, ongoing, multi-ancestry mega-biobank prospective cohort study that began enrollment in 2011, with participants recruited from over 60 sites across the US. The source population of the MVP is all patients of the Veterans Health Administration who were able to provide informed consent.<br>The Vanderbilt University DNA Databank (BioVU): Enrollment in the BioVU started in 2004, and this large, multi-ancestry biorepository was constructed through the donation of excess blood samples from clinical testing conducted on Vanderbilt clinic patients, with informed consent obtained.<br>The HEart failure Molecular Epidemiology for Therapeutic targetS (HERMES) consortium: The HERMES is a large consortium which includes 51 studies from 11 countries.<br>FinnGen: FinnGen is a research initiative that collaborates with both public and private entities. It utilizes imputed genotype data from Finnish biobanks, along with digital health records from Finnish health registries.
Ethics oversight	The MVP study was approved by the U.S. Department of Veterans Affairs Central Institutional Review Board. All MVP participants provided written informed consent.<br>The BioVU study was approved by the Vanderbilt University Medical Center Institutional Review Board. All BioVU participants provided written informed consent.

Note that full information on the approval of the study protocol must also be provided in the manuscript.

Field-specific reporting

Please select the one below that is the best fit for your research. If you are not sure, read the appropriate sections before making your selection.

☒ Life sciences ☐ Behavioural & social sciences ☐ Ecological, evolutionary & environmental sciences

For a reference copy of the document with all sections, see nature.com/documents/nr-reporting-summary-flat.pdf

Life sciences study design

All studies must disclose on these points even when the disclosure is negative.

Sample size	We performed genome-wide association studies (GWAS) with 38,781 HFrEF, 38,163 HFpEF participants, and 526,135 controls from European, African, Hispanic, and Asian ancestries, using data from the Million Veteran Program (MVP) and the Vanderbilt University DNA Databank (BioVU). Additionally, we performed a meta-analysis for all-cause HF, including 200,070 cases and 2,076,466 controls.
Data exclusions	We excluded individuals according to the standard quality control procedure of GWAS. Further details are described in the Method section.
Replication	Our GWAS of heart failure subtypes was conducted within the the Million Veteran Program (MVP), and was replicated/meta-analyzed using the Vanderbilt University DNA Databank (BioVU).
Randomization	Randomization is not applicable, because this is a population based case-control analysis.
Blinding	Blinding is not applicable, because this is a population based case-control analysis.

Reporting for specific materials, systems and methods

We require information from authors about some types of materials, experimental systems and methods used in many studies. Here, indicate whether each material, system or method listed is relevant to your study. If you are not sure if a list item applies to your research, read the appropriate section before selecting a response.

Materials & experimental systems

n/a	Involved in the study	
☒	☐	Antibodies
☒	☐	Eukaryotic cell lines
☒	☐	Palaeontology and archaeology
☒	☐	Animals and other organisms
☐	☒	Clinical data
☒	☐	Dual use research of concern
☒	☐	Plants

Methods

n/a	Involved in the study	
☒	☐	ChIP-seq
☒	☐	Flow cytometry
☒	☐	MRI-based neuroimaging

Clinical data

Policy information about clinical studies

All manuscripts should comply with the ICMJE guidelines for publication of clinical research and a completed CONSORT checklist must be included with all submissions.

Clinical trial registration: Not applicable.

Study protocol: Not applicable because our GWAS was a case-control study based on the retrospective observational data without any intervention.

Data collection: Clinical diagnosis of heart failure was obtained from electronic health records.

Outcomes: Heart failure and subtypes.

Plants

Seed stocks: *Report on the source of all seed stocks or other plant material used. If applicable, state the seed stock centre and catalogue number. If plant specimens were collected from the field, describe the collection location, date and sampling procedures.*

Novel plant genotypes: *Describe the methods by which all novel plant genotypes were produced. This includes those generated by transgenic approaches, gene editing, chemical/radiation-based mutagenesis and hybridization. For transgenic lines, describe the transformation method, the number of independent lines analyzed and the generation upon which experiments were performed. For gene-edited lines, describe the editor used, the endogenous sequence targeted for editing, the targeting guide RNA sequence (if applicable) and how the editor was applied.*

Authentication: *Describe any authentication procedures for each seed stock used or novel genotype generated. Describe any experiments used to assess the effect of a mutation and, where applicable, how potential secondary effects (e.g. second site T-DNA insertions, mosiacism, off-target gene editing) were examined.*
