# Supplementary material for: Multi-ancestry genetic architecture of heart failure subtypes
Source: Nat Commun. 2026 Aug 13;17:9730. doi: 10.1038/s41467-026-76438-y (PMC13562571; doi:10.1038/s41467-026-76438-y)
Supplement: Supplementary file 4 [file 41467_2026_76438_MOESM5_ESM.pdf]

nature portfolio

Peer Review File

Multi-ancestry Genetic Architecture of Heart Failure Subtypes

Corresponding Author: Professor Yan Sun

This file contains all reviewer reports in order by version, followed by all author rebuttals in order by version.

Version 0:

Reviewer comments:

Reviewer #1

(Remarks to the Author)
The authors explored the genetic underpinnings of heart failure with reduced ejection fraction (HFrEF) and heart failure with preserved ejection fraction (HFpEF) using a large-scale, multi-ancestry genome-wide association study (GWAS). The study combined data from over 600,000 individuals across the Million Veteran Program (MVP) and Vanderbilt University's BioVU, capturing participants of European, African, Hispanic, and Asian ancestries. The authors identified distinct genetic architectures not only between HF subtypes but also across populations. Downstream analyses—including PheWAS, tissue enrichment analysis, fine-mapping, and TWAS—revealed associated diseases, tissues, pathways, and genes. While the study highlights the heterogeneity of HF across subtypes and ancestries, I have several concerns regarding the manuscript:

1. Novel loci
While the Miami plots for HFpEF and HFrEF demonstrate their heterogeneity, the relationship with all-cause HF remains unclear. A Venn diagram showing loci shared or exclusive to all-cause HF, HFpEF, and HFrEF at genome-wide significance would clarify this visually.

2. TTN, BAG3
The identification of loci associated with representative cardiomyopathies underscores shared mechanisms between general HF and inherited cardiomyopathies. However, as many pathogenic variants in inherited cardiomyopathies are located in protein-coding regions, can coding variants be investigated using the identified tag SNPs by referencing population-specific linkage disequilibrium (LD) patterns?

3. PheWAS
For associations between HF-related loci and clinical risk factors, the tables currently lack intuitive visualization. Could a PheWAS Manhattan plot be added?
Furthermore, for notable risk factors—such as obesity, diabetes, hypertension, and lipid traits—could Mendelian Randomization (MR) be employed to estimate their causal effects on HFrEF and HFpEF, moving beyond simple genetic association?

4. European vs. African ancestry differences
For the β-plots in Figures 3 and 5, adding allele frequency comparisons between European and African ancestries alongside the effect sizes would allow readers to interpret both dimensions simultaneously.
Additionally, please include relevant literature supporting how observed allele frequency differences—combined with environmental and epidemiological context—strengthen the study's conclusions.

5. Variant-to-Gene mapping
While the manuscript employs TWAS for gene prioritization, were complementary integrative platforms such as Open Targets considered? Since different approaches prioritize genes differently, presenting independent results from multiple methods prior to integration would improve the robustness of gene-level inference.

6. Fine-mapping with Functional Prioritization
Beyond DEPICT's tissue prioritization (based on gene expression), could epigenomic data—such as ATAC-seq, ChIP-seq,

or single-nucleus RNA-seq (snRNA-seq)—be integrated to further prioritize causal genes or cell types at novel loci?

7. Subtype Polygenic Risk Score (PRS) Construction and Comparison
Could PRSs derived from GWASs of all-cause HF, HFrEF, and HFpEF be validated for subtype discrimination in external datasets (e.g., UK Biobank), or used to predict related cardiovascular conditions (e.g., ischemic heart disease, atrial fibrillation, or valvular disease)? Conversely, could PRSs for these conditions stratify HF subtypes? Additionally, could PRS-PheWAS be used to systematically explore traits linked to HF?

8. Drug Target Prioritization
Given the large-scale elucidation of the genetic architecture of heart failure, it would be valuable to examine the relationship between currently approved HF medications and GWAS findings, as well as explore potential drug repurposing opportunities based on newly identified loci. Specifically, could GWAS loci be mapped to known or putative drug targets (e.g., using DrugBank), and their clinical relevance discussed?

9. Internal and External Replication
For novel loci, replication would significantly strengthen the findings. I recommend demonstrating consistency via internal replication within the study cohort, and validating robustness through external replication in independent datasets.

10. Study Limitations
As the MVP cohort primarily consists of male participants, sex-stratified analyses may be underpowered. Please include in the main text any limitations arising from the demographic composition of the study population.

Reviewer #2

(Remarks to the Author)
The authors present genome wide association studies of heart failure with reduce and preserved ejection fraction, combining data from the Million Veteran Program and BioVU, for European, African, Hispanic, and Asian ancestries. This is an important increment in sample size for GWAS these disorders and addresses non-European ancestry groups. 9 novel genomic loci for HFrEF, 1 for HFpEF in a pan- ancestry analysis, and 12 for all-cause HF are reported . The lack of loci for HFpEF, despite an increase in sample size, suggest very low heritability and it would be interesting to report this.

A series of analyses are performed to explore the GWAS findings which are presented in a somewhat counter-intuitive sequence. Some of the claims made in the declarative subtitles are not fully supported by the results presented, and in some cases the results presented to not relate to the subtitle. Generally, the reporting could be clearer by including sufficient information, for example 'gene-based analysis' is report in the results section which appears to represent findings from MAGMA, but this only clear when reading with the methods. The approaches to prioritise genes at the identified loci could be updated to use modern approaches. The downstream analyses could be improved with more precise reporting and a logical structure and flow.

While the authors report a two-fold increase in sample size for HF subtypes, there is a modest yield in genetic discovery. Importantly they do address previously under-represented ancestry groups. The approach to case identification and GWAS appear robust, however, the manuscript would benefit from revision to improve the precision and completeness of reporting, to explain to the reader the significance (or lack thereof) of the findings, and to ensure that sufficient information is provided to interpret the results. The tables and figures could also be improved.

Specific comments
• The type of analysis for which results reported are not always clear in abstract
• Listing genes by nearest versus cytogenetic location not consistent
• "Genetic factors contribute to HF risk, supported by an estimated heritability of ~ 25% for both all-cause HF and its subtypes.8" is not correct, the cited study did not include HF subtypes
• The h2SNP of the traits should be reported.
• Report heterogeneity by ancestry in the pan-ancestry analysis
• No use of power adjusted replication framework when evaluating non-Eur ancestries
• Section on genetic architecture describes locus discovery
• The rational for pleiotropy scan of loci identified in individuals of African ancestry is not well outlined, and the analysis is limited.
• The approach to fine mapping and gene prioritisation could be improved to provide a more cohesive approach and to make use of modern tools, such as PoPs.
• The main table would be better located in the supplement.
• The multi-lane circus plots contain different analysis findings and are difficult to follow. These figures could be labelled, improved for clarity.
• Other figures are not referenced fully in the text – ie the ancestry heterogeneity plot (no figure number provide).
• Generally, the clarity and flow of the reporting could be improved, to explain rationale for the various analyses and the significance of the findings.

Reviewer #3

(Remarks to the Author)
Liu et al are reporting a GWAS for heart failure (HF) subtypes and all-cause HF in populations similar to genetic ancestries of multiple reference populations. Using standard downstream analyses for GWAS, they characterize HFrEF, HFpEF, and all-cause HF findings in populations with genetic ancestry similar to that of European and African reference populations. They report some novel findings which provide insights into disease etiology.

Major comments:

1) The use of "ancestry" and "ancestries" throughout the manuscript should conform to the NASEM report guidelines for referring to populations. Chapter 5, best Practice 6, page 125 of the report might be informative. https://www.nationalacademies.org/our-work/use-of-race-ethnicity-and-ancestry-as-population-descriptors-in-genomics-research

Furthermore, the use of ancestry and population should be used more consistently and accurately. For example, "Population-specific" on line 90 would be better as ancestry-specific. On line 118, the genetic correlations weren't done among European ancestry, they were among the GWAS estimates from individuals with genetic ancestry similar to reference populations of European ancestry. On line 172, the findings are generalizable for populations or individuals of a given ancestry. In Table 2 these are loci from a sample of individuals with genetic ancestry matching African reference populations, not "African HFrEF loci"

I understand that for MVP, the population labels also include self-identified race/ethnicity which limits the use of solely "genetic ancestry" in describing the population labels. A succinct definition of the ancestry labels early on in the manuscript would aid the reader in understanding the definition of "African ancestry" or "European ancestry" in this study.

For BioVU additional details are needed to understand how ancestry was inferred with STRUCTURE 2.2.

Technically, GWAS from the Finnish population are described as Finnish rather than European due to population structure that is unique compared to non-Finnish Europeans (https://www.nature.com/articles/nature19057). So the meta-analysis including FinnGen results could be described with more nuance, including in the methods (line 279).

2) Line 132-134: It sounds like you needed to relax your significant threshold to find any results from DEPICT for HFpEF. If so, this needs to be made clear "For HFpEF, no gene-sets were significant using only GWS loci, but using nominally significant genomic loci with p-value < 1E-4." Is there a justification for why this particular significance threshold was used?

3) Please be clear about what constitutes novel. In the discussion, line 164, it would be better to summarize the total loci found and then the number of "potentially novel loci. " In the methods, the literature used to define novel should be cited lines 311-313.

4) Line 166-168: Are the heterogeneous effects in HFpEF simply due to issues of power given that HFrEF has 9 GWS loci and HFpEF only has 1? For the 91% of HFrEF loci with heterogeneous effects in HFpEF—how are you counting loci here? Is it instead genome-wide significant variants in the 9 loci? This may be a flawed estimate. Perhaps it is better to report the number of lead SNPs at each of the 9 loci that has a heterogeneous effect in HFrEF. Finally, the conclusion "this suggests HFrEF and HFpEF have distinct underlying mechanisms..." should be softened as this has been generally known from clinical findings and previous research. These findings add to the evidence that HFrEF and HFpEF have distinct underlying mechanisms.

5) Lines 171-172: The sentence should be revised for clarity, but it also should be more specific — this shows there could be a shared genetic etiology. There are better references instead of reference 17. I would suggest the GWAS studies that first linked FTO to BMI or more recent GWAS by large consortia like GIANT, as well as work by Melina Claussnitzer and colleagues to disentangle this locus.

6) Table 1 should be condensed for readability in the manuscript with the current version as a supplementary table. I would suggest including only the meta-analysis summary statistics in the manuscript. The novel columns should be collapsed into one.

7) The abstract mentions Hispanic and Asian ancestries and the summary statistics are listed in Table 1, but the manuscript does not delve into any ancestry-specific findings from these populations. If there were not any significant loci this should be explained in the discussion.

8) The discussion lacks any elaboration on the limitations of the study.

9) Lines 288-291: How are related individuals considered in MVP and BioVU? Despite stratification by ancestry, I worry about additional population structure due to admixture in the samples. Population stratification and relatedness can be addressed by using logistic mixed models like SAIGE or REGENIE. Given some of the larger genomic control values, I would like to see a sensitivity analysis at least with an LMM in a non-European ancestry sample.

10) Figure 2 could be moved to supplement.

Minor comments:

Abstract

1) This sentence needs revision for clarity and accuracy:
"Four HFrEF loci in African ancestry, including CD36, SPI1, TRIM48, and SPNS3, which were rare in European ancestry." First, as an incomplete sentence it should be revised for clarity. Second, the loci themselves cannot be rare, but the alleles in these loci can be. Third, the way ancestry is represented here could be more accurate.
For example… "the allele frequency of the risk allele is considered rare in study participants with a genetic ancestry similar to that of European reference populations."

Introduction

2) Specify US dollars on line 54 if that is the currency from the reference

3) Period after LVEF on line 56 to make the subtype description more clear

4) Case/control sample sizes for the GWASs cited on line 62/63 and 67 will give the reader a sense of how these compares to the current study

Results

5) Are the loci mentioned in lines 84-87 also genome wide significant? If so specify as you have in the previous paragraph or drop "GWS" in the text since the sub-section title specifies these are GWS.

6) Line 94-95: the effect sizes significantly higher in one ancestry versus another? If so, include a p-value and the type of statistical test used.

7) Line 100-101: ARSG showed a null effect but does that mean the other two were significant in the same direction of effect? If so, please describe more clearly.

8) Line 105-109: Pleas remind the reader how many African-ancestry specific HFrEF and HFpEF and all-cause HF loci were tested in the PheWAS e.g. "HFrEF loci (n=7)"

9) Line 125-130: It is not adequately clear to the reader that this is based on eQTLs from GTEx. Despite being described in the methods, a more clear phrasing in the results is needed to explain the connection between SNPs/loci and tissue types (e.g. tissue-speciifc eQTL?, significant eQTL in a given tissue?)

10) Lines 146-150: Please remind the reader where the pQTL results are from. It would also be advisable to soften the conclusions—the analysis revealed potential molecular mechanisms but not "the" molecular mechanisms.

11) Lines 146-150: Why were pQTL and enhancers grouped together in the results? Enhancers would likely fit better with more the regulatory related findings such as the tissue expression and enrichment section.

12) Lines 146-150: Please also remind the reader how these regions of enhancers are related to the GWAS findings. For example, do the significant loci fall in enhancer regions? If so, how are these enhancer regions described. Could you name which loci seem to be enhancer-related?

13) Lines 146-150: Why was this analysis only done for all-cause HF? Were there no findings for the other subtypes? If so, please state this to avoid "cherry picking" results.

14) Line 152: Use GWS as the acronym for genome-wide significant and please check the rest of the article for consistency.

15) The interpretation of credible set analyses on line 154 is overstated. The credible set analysis simply suggests the causal variant is included in the given set of SNPs.

16) Line 160: Please state the PIP of FTO instead of PIP > 0.5

Discussion

17) Line 178: Genes CCDC92 and DNAH10 are included without context as to what subtype they are associated with. Please clarify this for the reader.

18) Line 181: Some loci associated with HFrEF are connected to Smads, not all loci. Please define which or write how many. Remind the reader what evidence connects them to Smads? Please also expand Smads to its full name before using the acronym.

19) Several of the claims in the discussion should be better cited. Is reference 25 sufficient for lines 189-190?

20) Line 211: You mention CD36 but you also described it in lines 188-192. Please combine the discussion on CD36 for clarity.

Tables

21) Gene names should be italicized

22) The test used for the heterogeneity p-value should be stated in the table or legend

Methods

23) Lines 251-256: If this physician-reviewed algorithm or NLP method have been previously published please cite or more fully describe (perhaps in supplement) for reproducibility

24) Line 291: If the age used was age at diagnosis, please specify.

25) Why were genetic correlations not estimated between the subtypes and all-cause HF for African ancestry meta-analysis?

26) Line 345: Why was significance defined as a corrected q-value < 0.2 for the gene set enrichment analysis? This is inconsistent with previous thresholds used and should be justified.

27) Line 368: Please cite the Fenland study

Figures

28) Figure 3: Need a third color for loci that are genome wide significant in both studies? Please use a correlation coefficient estimate with 95% confidence intervals instead of a fitted line. Loci that are off the diagonal could be labelled.

29) Figure 5: Please use colors different from Figure 3 for clarity. Loci that are off the diagonal could be labelled.

Version 1:

Reviewer comments:

Reviewer #2

(Remarks to the Author)
Addressing the points of my previous review -

* The h2SNP of the traits should be reported.
The authors have presented h2snp estimated using GREML-LDMS. This approach requires individual participant data, so I expect these results are from the MVP sample, but this is not specified. I suspect that the estimates are on the observed rather than the liability scale. If so, please convert to the liability scale so that they are comparable to other studies. If not, then the estimates are usually high compared with the h2snp reported in most published GWAS. Unusually, the heritability estimate for HFpEF is higher than for HFrEF, and this should be checked.

• Section on genetic architecture describes locus discovery

The h2 SNP should be given with 95% CI in the results - I suspect these are wide.

• The approach to fine mapping and gene prioritisation could be improved to provide a
more cohesive approach and to make use of modern tools, such as PoPs.

The authors highlight the challenge of implementing PoPs as a method for locus to gene prioritisation due to the difficulty of developing a well-specified LD reference, but then a number of the methods used also rely on an LD SNP correlation matrix (MAGMA, TWAS, colocalisation, Mendelian randomisation). It is not clear what references were used for these analyses - perhaps a within-sample LD matrix was computed from the MVP cohort, and the mapping was done in this GWAS subset?

There are similar issues in other sections of the manuscript.

Reviewer #3

(Remarks to the Author)
Reviewer #1

2. Thank you to the authors for looking into these pathogenic variants in clinker. It may be useful to point out in the discussion of TTN or in the limitations section the scenario in which rare pathogenic variants for cardiomyopathy are driving synthetic associations in these loci.

3. Thank you for adding a Mendelian Randomization analysis. Typically, you use a primary method for inference and other methods are used as sensitivity analysis. Are you using the standard IVW p-value for determining significance? The methods and results should be more clear what is used for inference and if you're then requiring directional consistency and consistent effects after outlier removal in the sensitivity analyses. Weighted median estimation and weighted mode estimation would be additional sensitivity analysis methods to include. Please also make clear if fixed-effects IVW was used and where the pleiotropy test p-value in the table is coming from (e.g. MR-Egger regression intercept test)

4. Thank you for the additional plot to compare allele frequency. Is it possible to include some additional gene labels in panel B, to make more direct comparisons between the points across the two panels?

In the text you've added, can you please make it clear that you're talking about the allele frequency of the leadSNP at each locus, as loci themselves do not have allele frequencies.

Otherwise, the authors have addressed the concerns very well.

9. Thank for your pursuing external validation. In the text, could you also include the proportion of loci you're classifying as novel which replicate in Biobank Japan?

10. The addition to the text could include the proportion of male veterans. Please be consistent with either men/women (gender) or male/female (sex) in these sentences.

Reviewer #3

Major comments

8. Suggest a re-write for consistent use of sex/gender terminology:

…predominantly composed of male veterans which limits our power for sex-stratified analyses, particularly among female veterans, and may constrain the generalizability of our findings to females. Larger and more ….including those with a higher proportion of female participants,….

In this section, it would be good to be specific that the external replication in BiobankJapan was just for all-cause HF

Minor comments

1. I would further clarify it is the SNP you're defining as the lead SNP which has the low risk-allele frequency.

5. The last sentence should be 102 GWAS loci and 3 GWS loci for consistency with the previous and readability.

10. When you list the rsIDs, perhaps add the gene name you're using for that locus

Version 2:

Reviewer comments:

Reviewer #2

(Remarks to the Author)
Thank you for addressing my comments.

Reviewer #3

(Remarks to the Author)
For the MR results with HFrEF, Type 2 Diabetes and pulse pressure have persistently significant pleiotropy test and MR Egger association is non-significant after outlier removal. Therefore, I think the results sentence "Type 2 diabetes, triglycerides, and pulse pressure demonstrated potential causal effects for HFrEF. " is overstating the evidence.

Finally, language in the results section around causality should use more careful wording such as "Genetically predicted X was associated with Y" or "MR provides evidence supporting a causal role of X on Y."

Table S25A and B should make clear that the pleiotropy test p-value is from MR Egger intercept test. Is an MR-PRESSO global test p-value available for the analyses with MR-PRESSO?

In copyediting, fixed-effects should be changed to fixed-effect (I made this mistake in my review, sorry!)

Otherwise, the authors have addressed my previous comments in a satisfactory manner. Thank you!

Open Access This Peer Review File is licensed under a Creative Commons Attribution 4.0 International License, which permits use, sharing, adaptation, distribution and reproduction in any medium or format, as long as you give appropriate credit to the original author(s) and the source, provide a link to the Creative Commons license, and indicate if changes were made.
In cases where reviewers are anonymous, credit should be given to 'Anonymous Referee' and the source.
The images or other third party material in this Peer Review File are included in the article's Creative Commons license, unless indicated otherwise in a credit line to the material. If material is not included in the article's Creative Commons license and your intended use is not permitted by statutory regulation or exceeds the permitted use, you will need to obtain permission directly from the copyright holder.
To view a copy of this license, visit https://creativecommons.org/licenses/by/4.0/

Reviewer 1:

The authors explored the genetic underpinnings of heart failure with reduced ejection fraction (HFrEF) and heart failure with preserved ejection fraction (HFpEF) using a large-scale, multi-ancestry genome-wide association study (GWAS). The study combined data from over 600,000 individuals across the Million Veteran Program (MVP) and Vanderbilt University's BioVU, capturing participants of European, African, Hispanic, and Asian ancestries. The authors identified distinct genetic architectures not only between HF subtypes but also across populations. Downstream analyses—including PheWAS, tissue enrichment analysis, fine-mapping, and TWAS—revealed associated diseases, tissues, pathways, and genes. While the study highlights the heterogeneity of HF across subtypes and ancestries, I have several concerns regarding the manuscript:

1. Novel loci

While the Miami plots for HFpEF and HFrEF demonstrate their heterogeneity, the relationship with all-cause HF remains unclear. A Venn diagram showing loci shared or exclusive to all-cause HF, HFpEF, and HFrEF at genome-wide significance would clarify this visually.

We have added a Venn diagram as Figure S4, as shown below:

Figure S4. Venn Diagram for Genome-wide Significant Loci Shared or Exclusive to All-cause HF, HFrEF, and HFpEF.

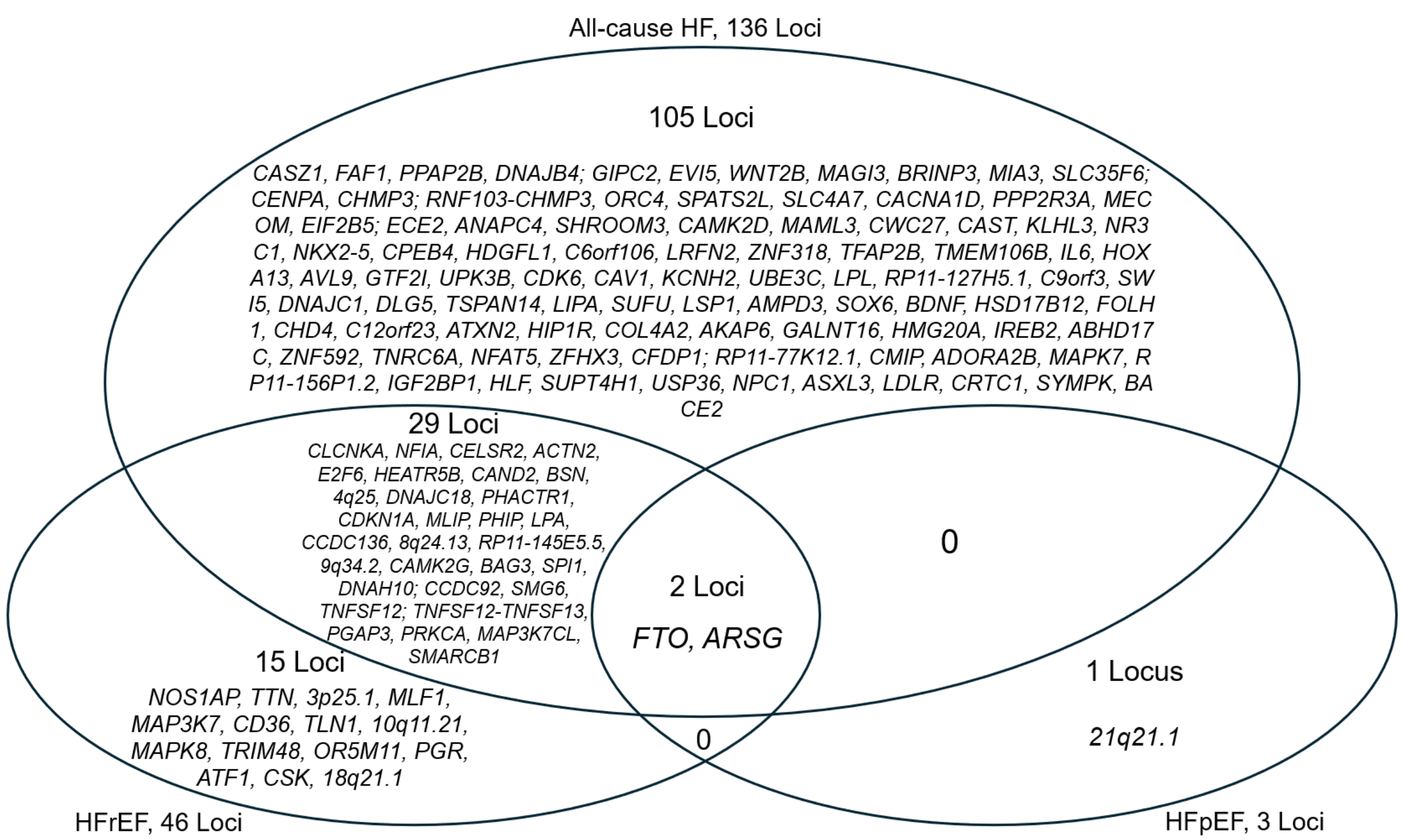

2. TTN, BAG3: The identification of loci associated with representative cardiomyopathies underscores shared mechanisms between general HF and inherited cardiomyopathies. However, as many pathogenic variants in inherited cardiomyopathies are located in protein-coding regions, can coding variants be investigated using the identified tag SNPs by referencing population-specific linkage disequilibrium (LD) patterns?

We appreciate the reviewer's insightful comment regarding the relationship between the *TTN* and *BAG3* loci and known pathogenic coding variants for inherited cardiomyopathies. To address this, we queried ClinVar for pathogenic missense variants reported in association with cardiomyopathy at the *TTN* and *BAG3* loci (Response Table 1). All established pathogenic variants identified in ClinVar were extremely rare, with minor allele frequencies ranging from 0 to 7×10^{-5}. Given this extremely low frequency, these unmeasured pathogenic coding variants are not well tagged by the common and low-frequency sentinel variants driving our GWAS signals. Therefore, we couldn't evaluate their associations with HF using LD-based approaches in this dataset.

Response Table 1. Pathogenic Missense Variants in *TTN* and *BAG3* Associated with Cardiomyopathy.

Gene	rsID	Chr.	Position (hg19)	Minor Allele Frequency
TTN	rs281864928	2	179391875	0
TTN	rs375159973	2	179399128	0.00005
TTN	rs753334568	2	179410768	0.00002
TTN	rs869320742	2	179410776	0.0000064
TTN	rs869320741	2	179410778	0
TTN	rs869320740	2	179410829	0.0000029
TTN	rs1190242728	2	179597966	0.00007
TTN	rs28933405	2	179650726	0.00006
BAG3	rs1589630141	10	121431884	0
BAG3	rs121918312	10	121431885	0
BAG3	rs397516881	10	121436429	0.00004
BAG3	rs756020699	10	121436474	0

3. PheWAS

For associations between HF-related loci and clinical risk factors, the tables currently lack intuitive visualization. Could a PheWAS Manhattan plot be added?

We have added PheWAS Manhattan plots for the genome-wide significant loci identified in the African ancestry meta-analyses: 7 for HFrEF (Table 2), 2 for HFpEF (Table 2), and 3 for all-cause HF (Table S7), as shown below:

Figure S7A. Manhattan Plot of the PheWAS for the HFrEF locus *HSPB7* (rs1572381) among African Ancestry.

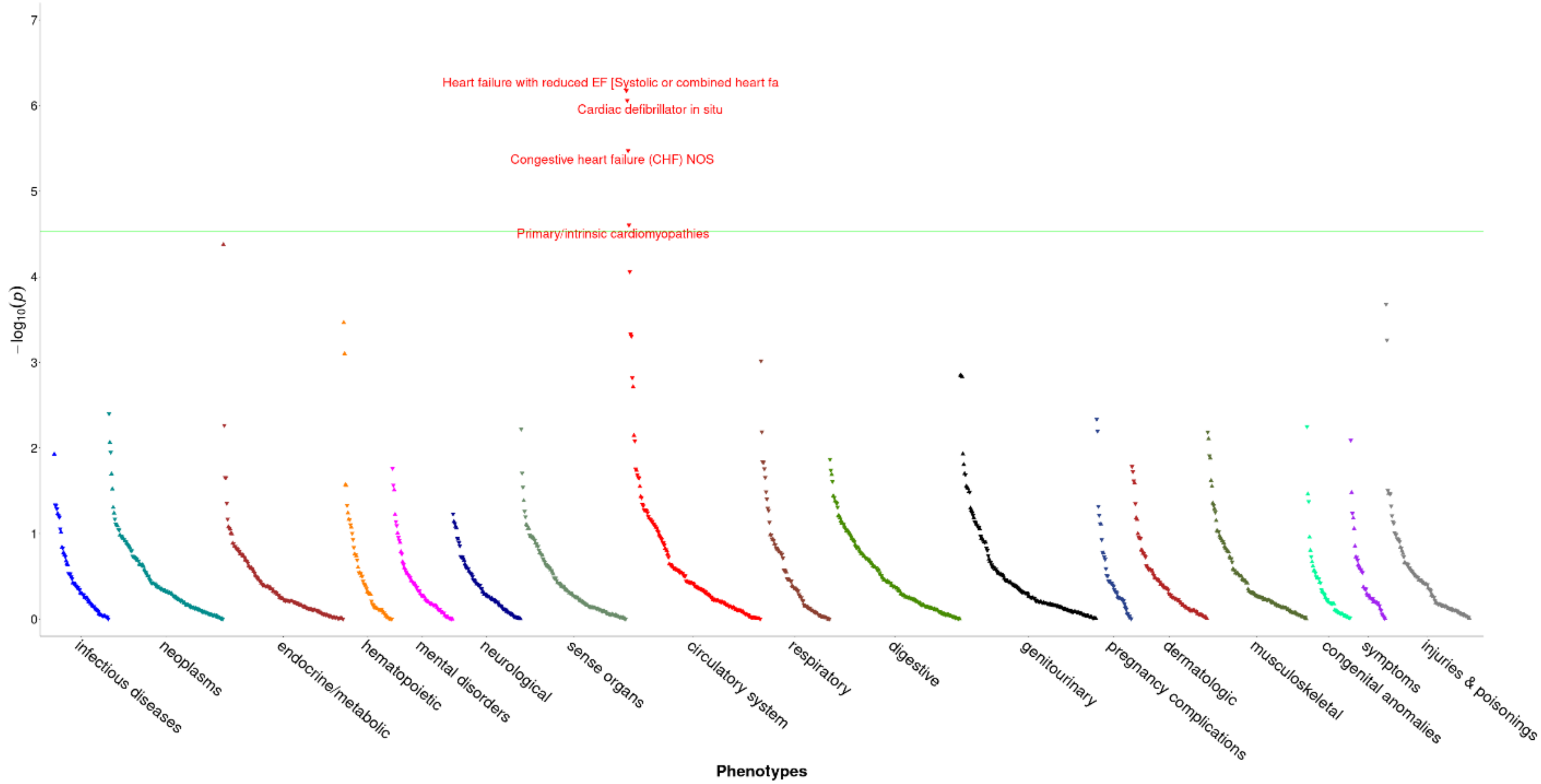

Figure S7B. Manhattan Plot of the PheWAS for the HFrEF locus *CCDC141* (rs7564036) among African Ancestry.

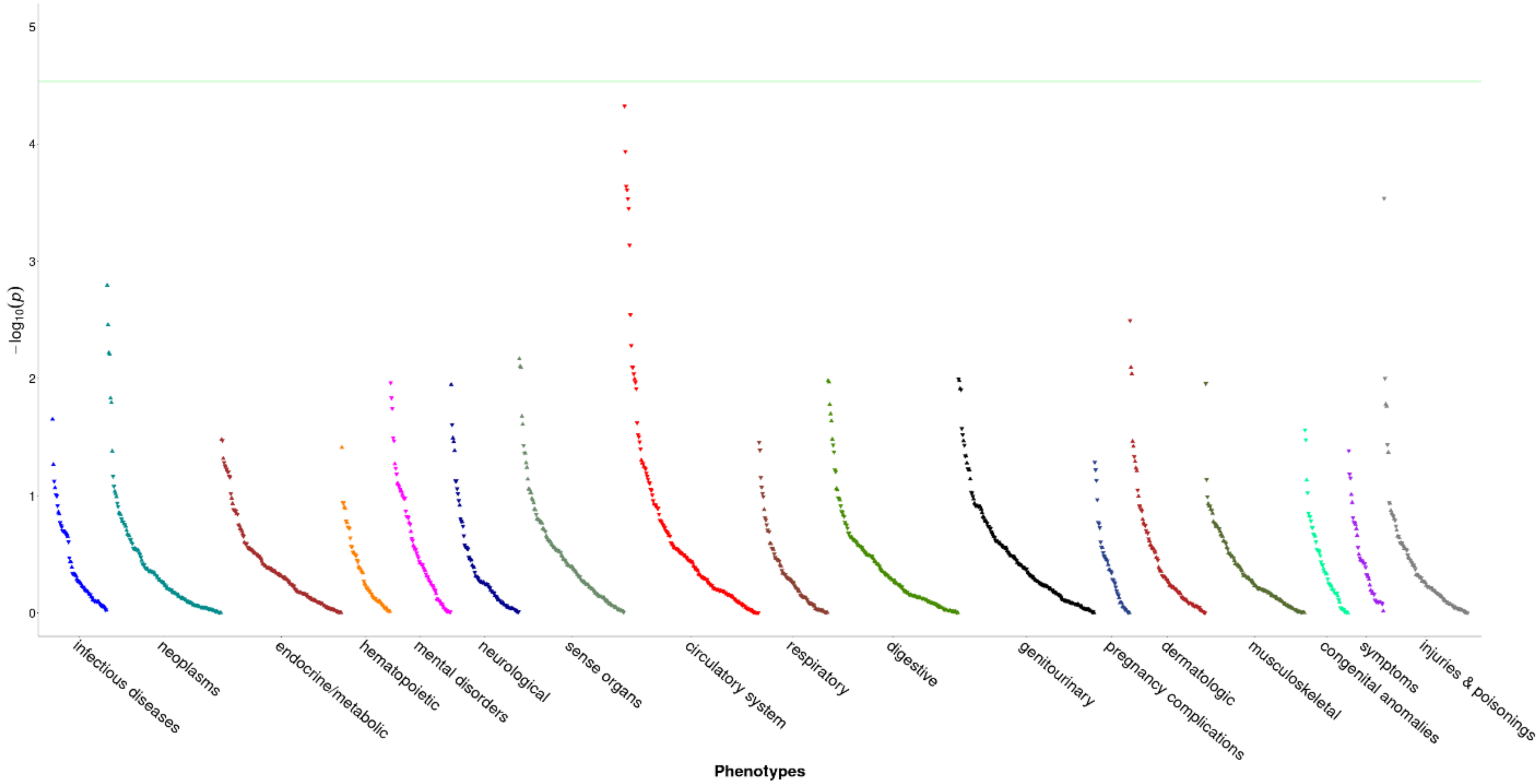

Figure S7C. Manhattan Plot of the PheWAS for the HFrEF locus *CDKN1A* (rs3176326) among African Ancestry.

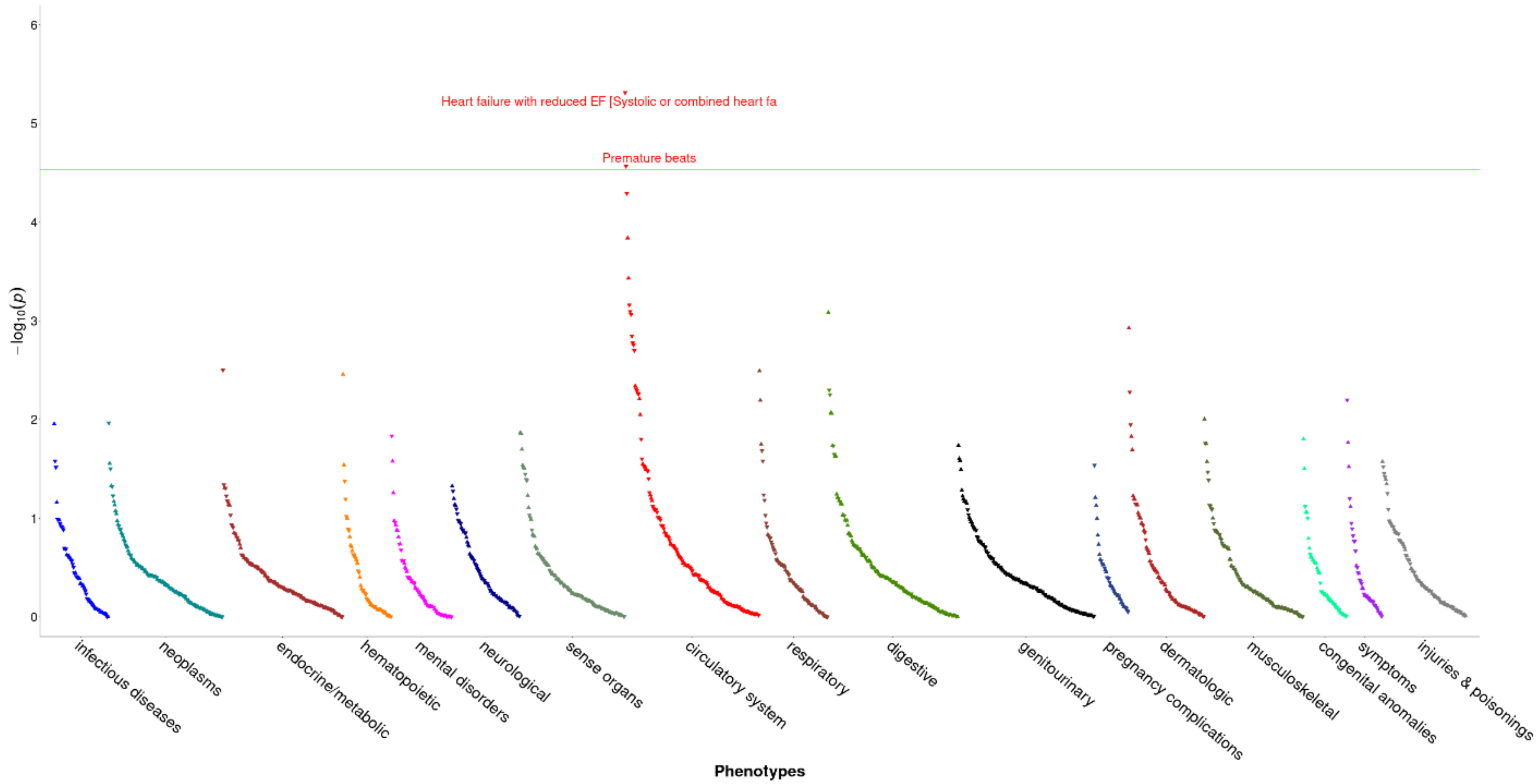

Figure S7D. Manhattan Plot of the PheWAS for the HFrEF locus *CD36* (rs3211938) among African Ancestry.

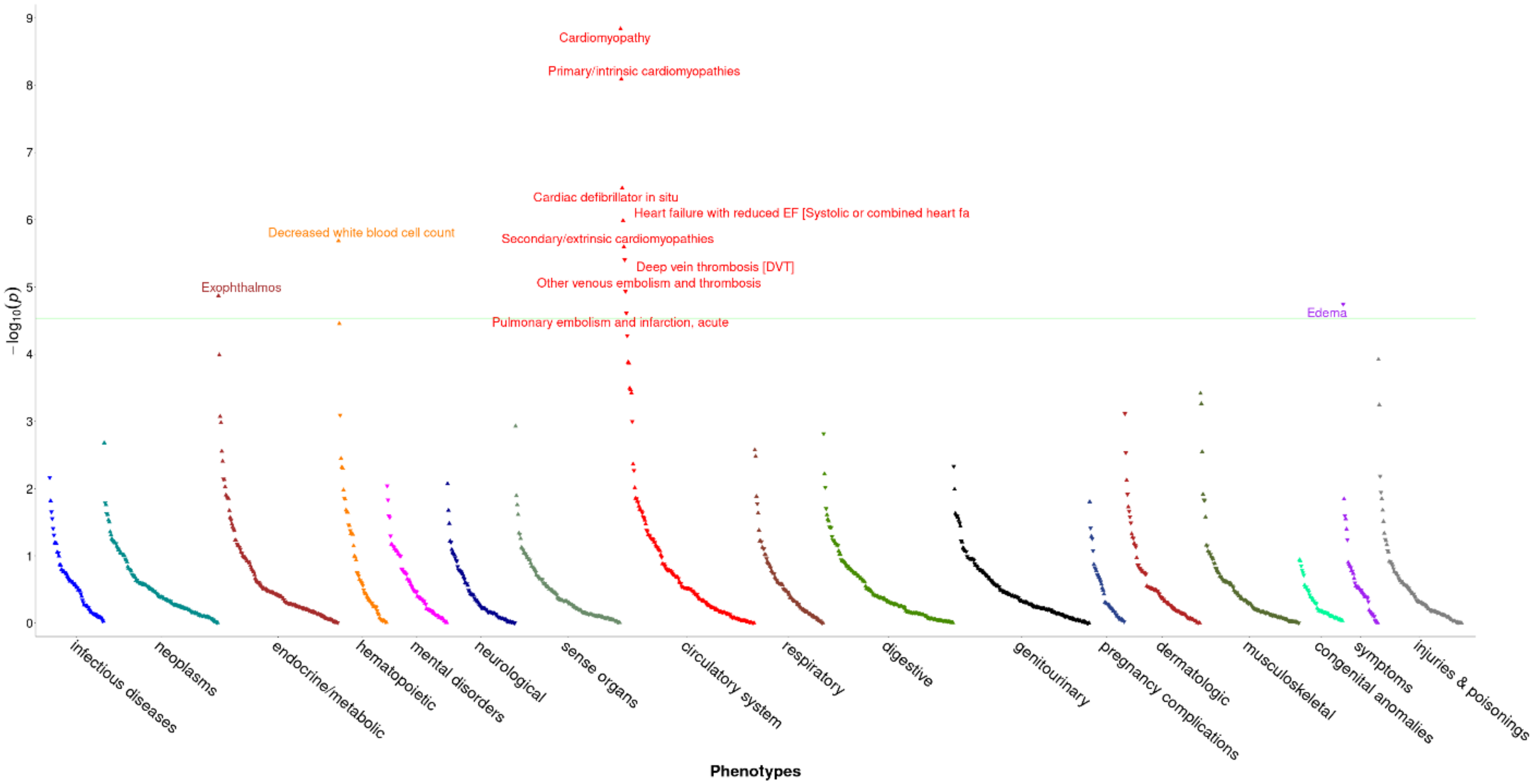

Figure S7E. Manhattan Plot of the PheWAS for the HFrEF locus *SPI1* (rs113123267) among African Ancestry.

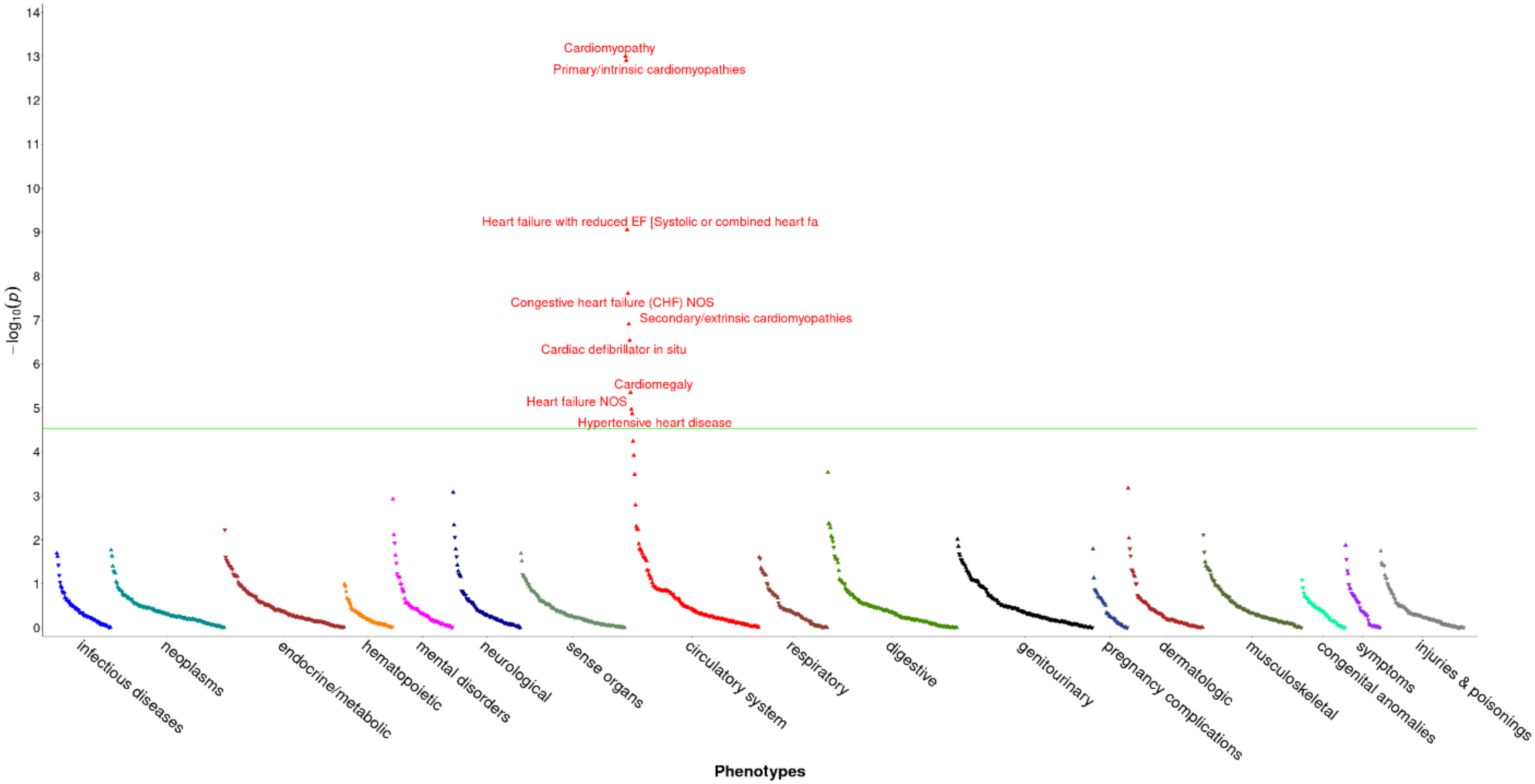

Figure S7F. Manhattan Plot of the PheWAS for the HFrEF locus *TRIM48* (rs111786504) among African Ancestry.

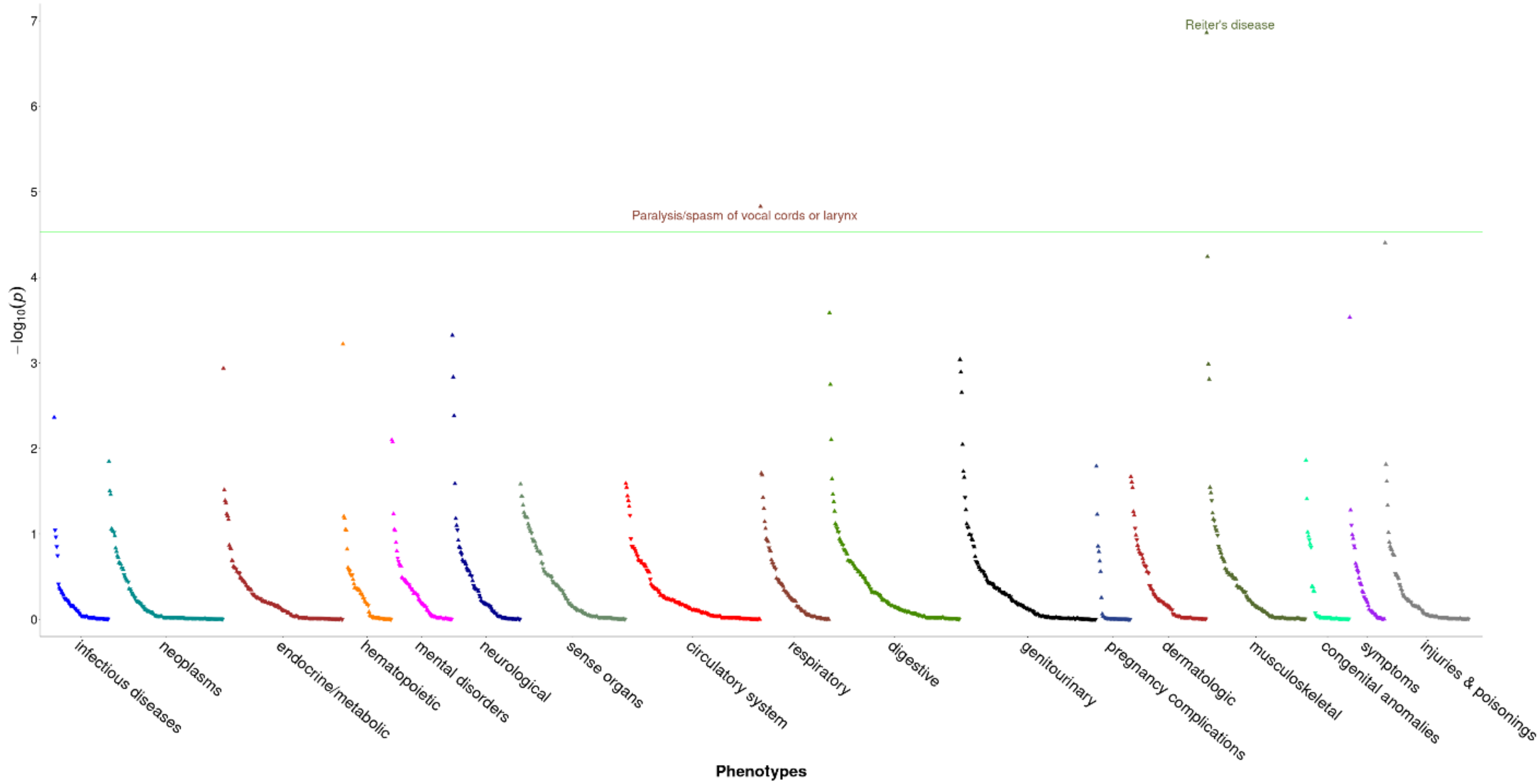

Figure S7G. Manhattan Plot of the PheWAS for the HFrEF locus *SPNS3* (rs75531264) among African Ancestry.

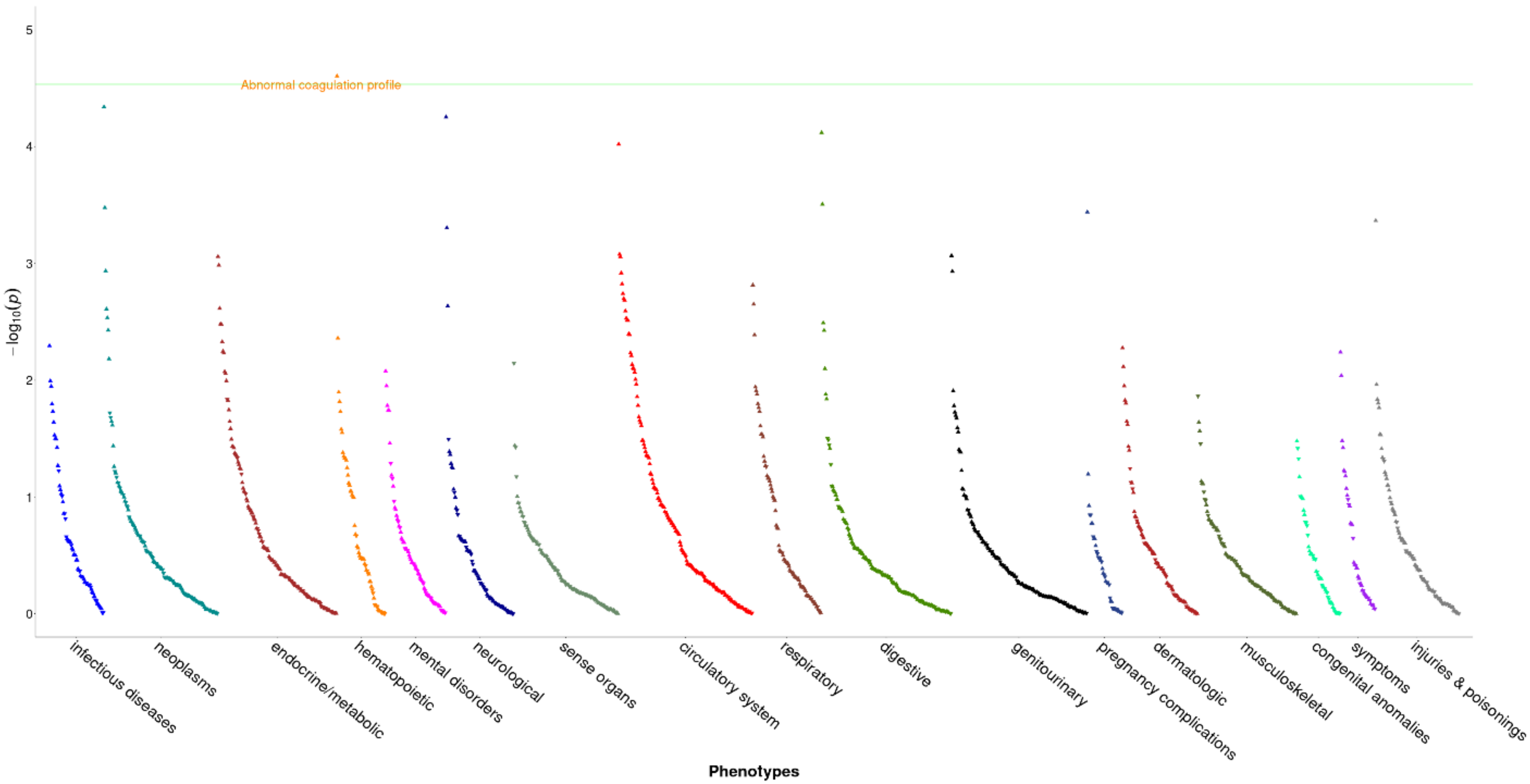

Figure S7H. Manhattan Plot of the PheWAS for the HFpEF locus 14q32.13 (rs12586269) among African Ancestry.

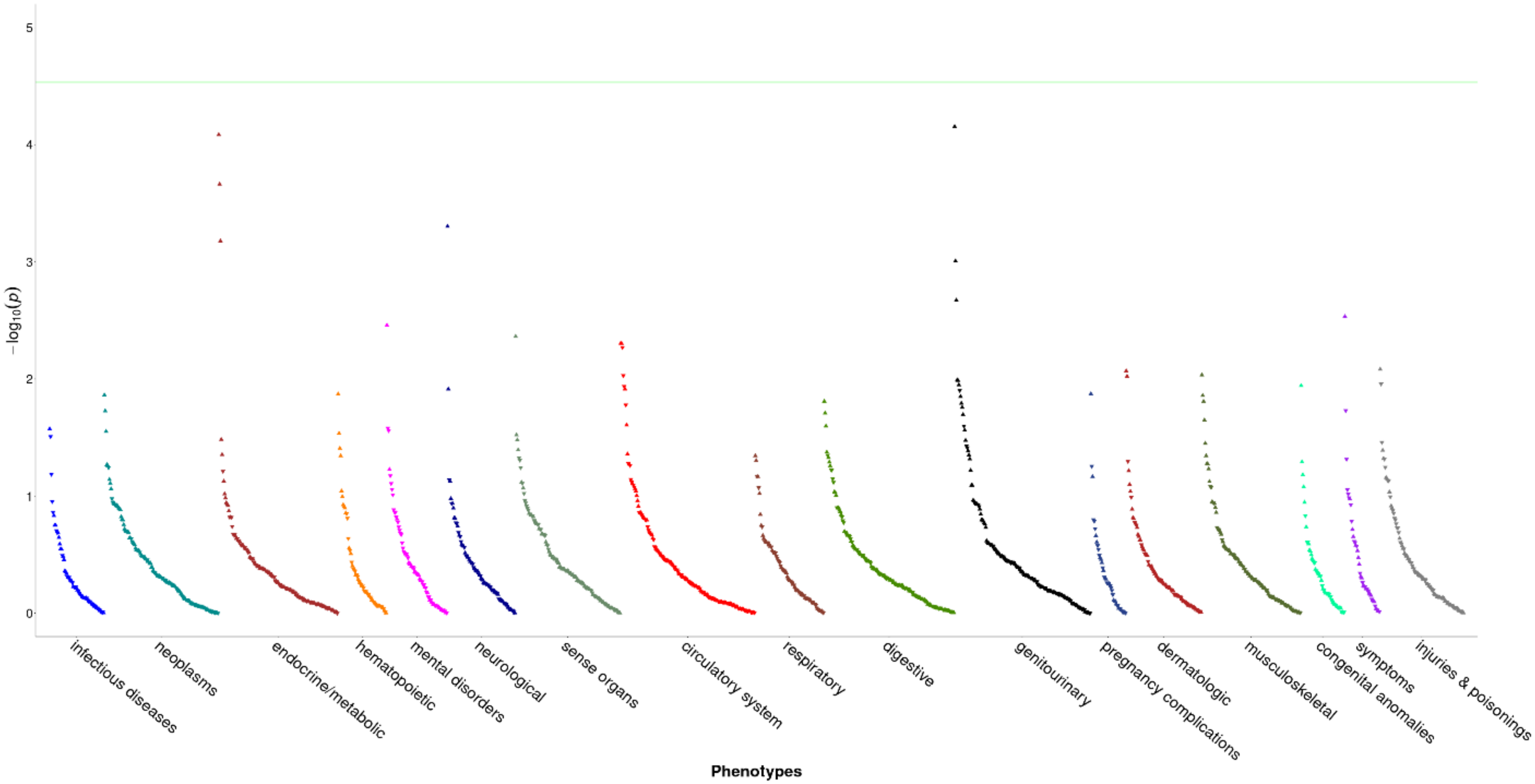

Figure S7I. Manhattan Plot of the PheWAS for the HFpEF locus *ARSG* (rs75716431) among African Ancestry.

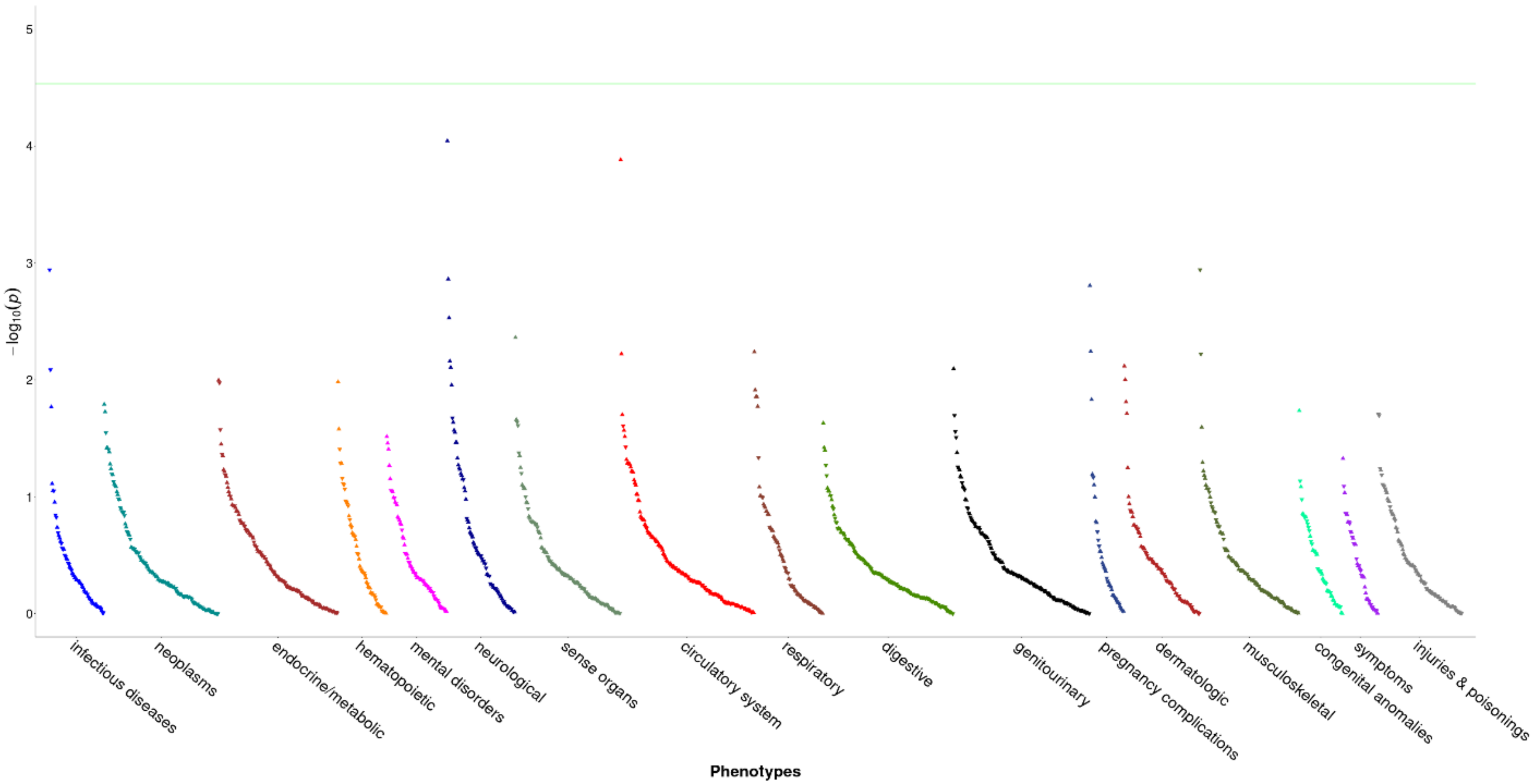

Figure S7J. Manhattan Plot of the PheWAS for the All-cause HF locus *GUCA1C* (rs2593932) among African Ancestry.

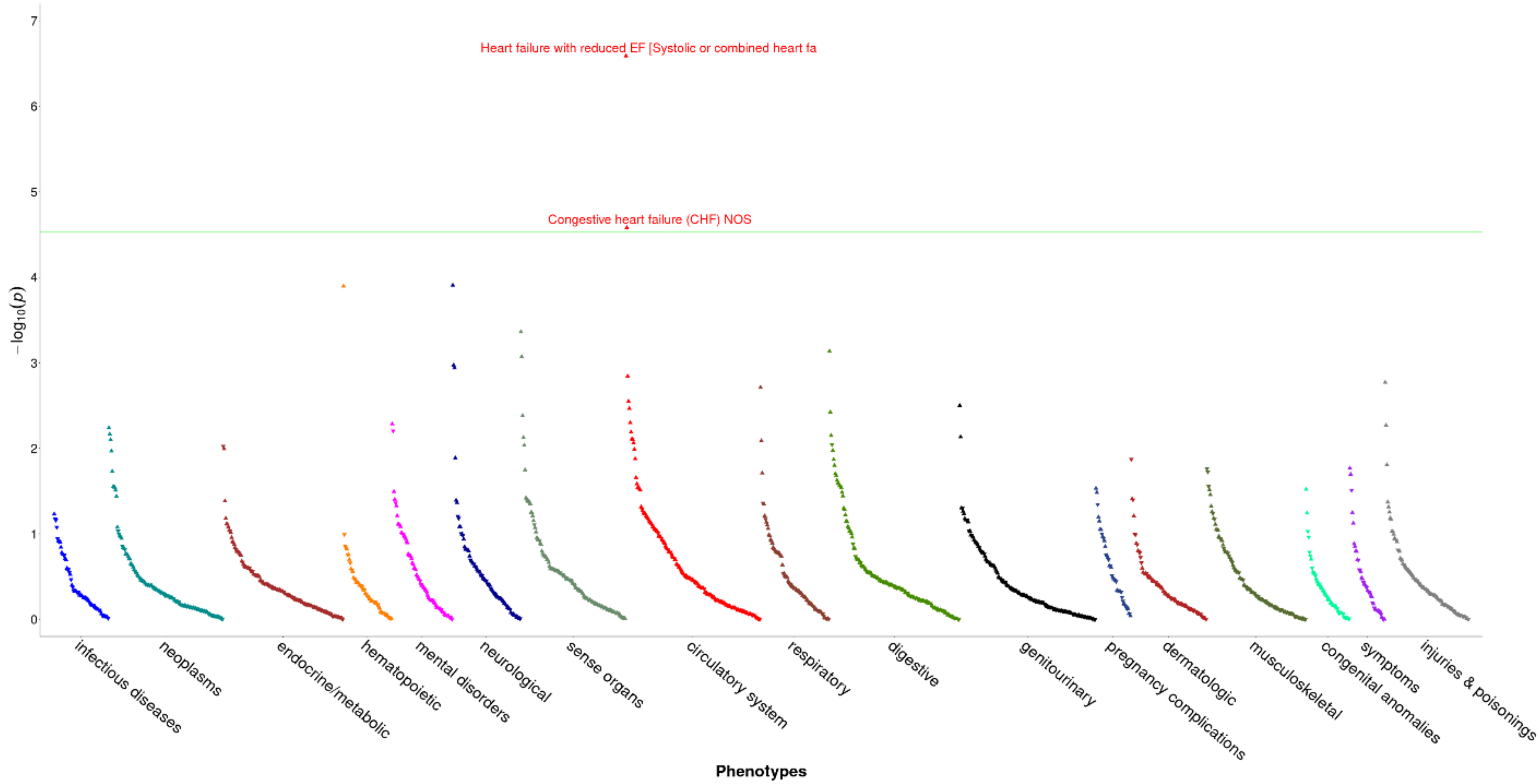

Figure S7K. Manhattan Plot of the PheWAS for the All-cause HF locus *AVL9* (rs115698376) among African Ancestry.

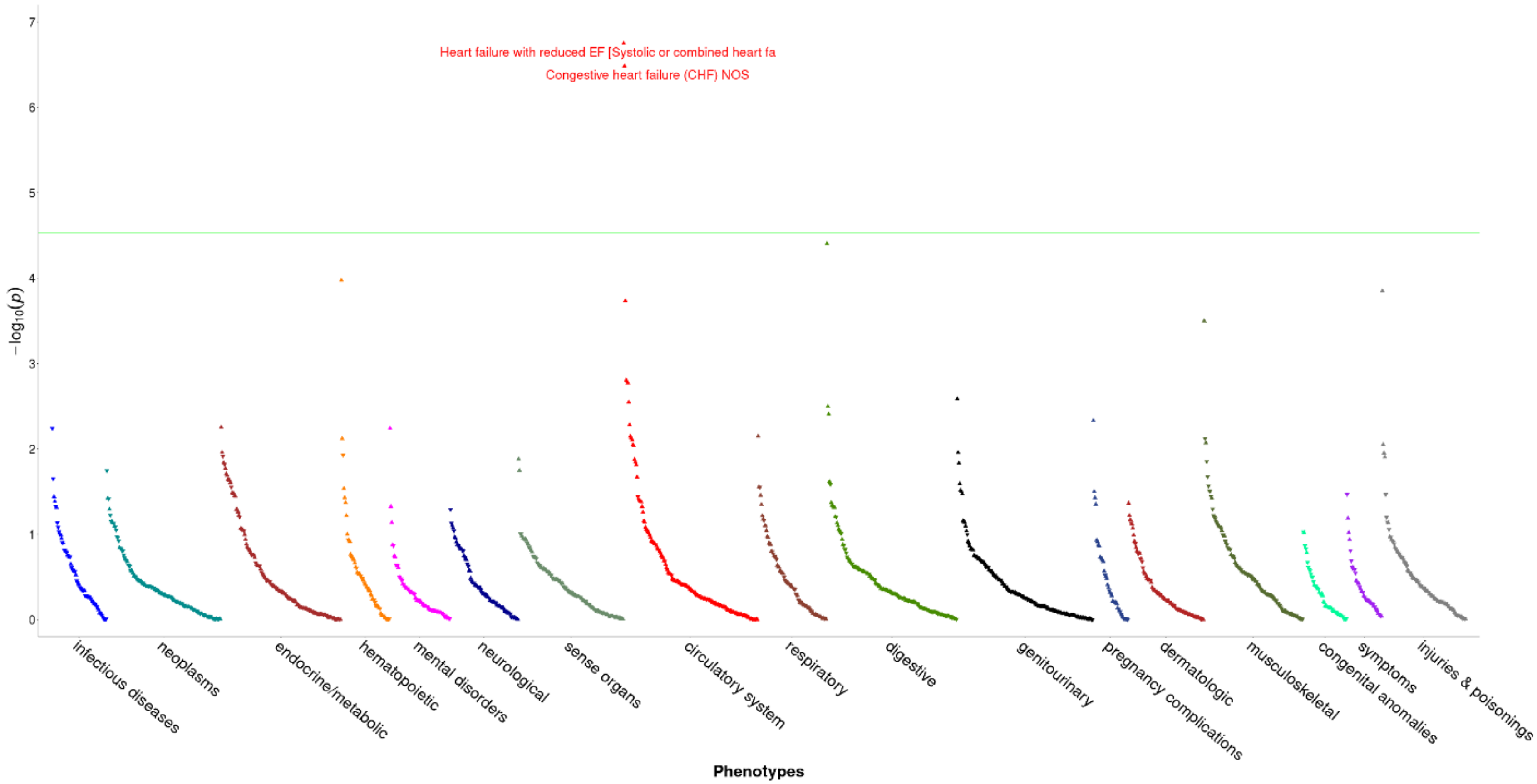

Figure S7L. Manhattan Plot of the PheWAS for the All-cause HF locus *SPI1* (rs73453764) among African Ancestry.

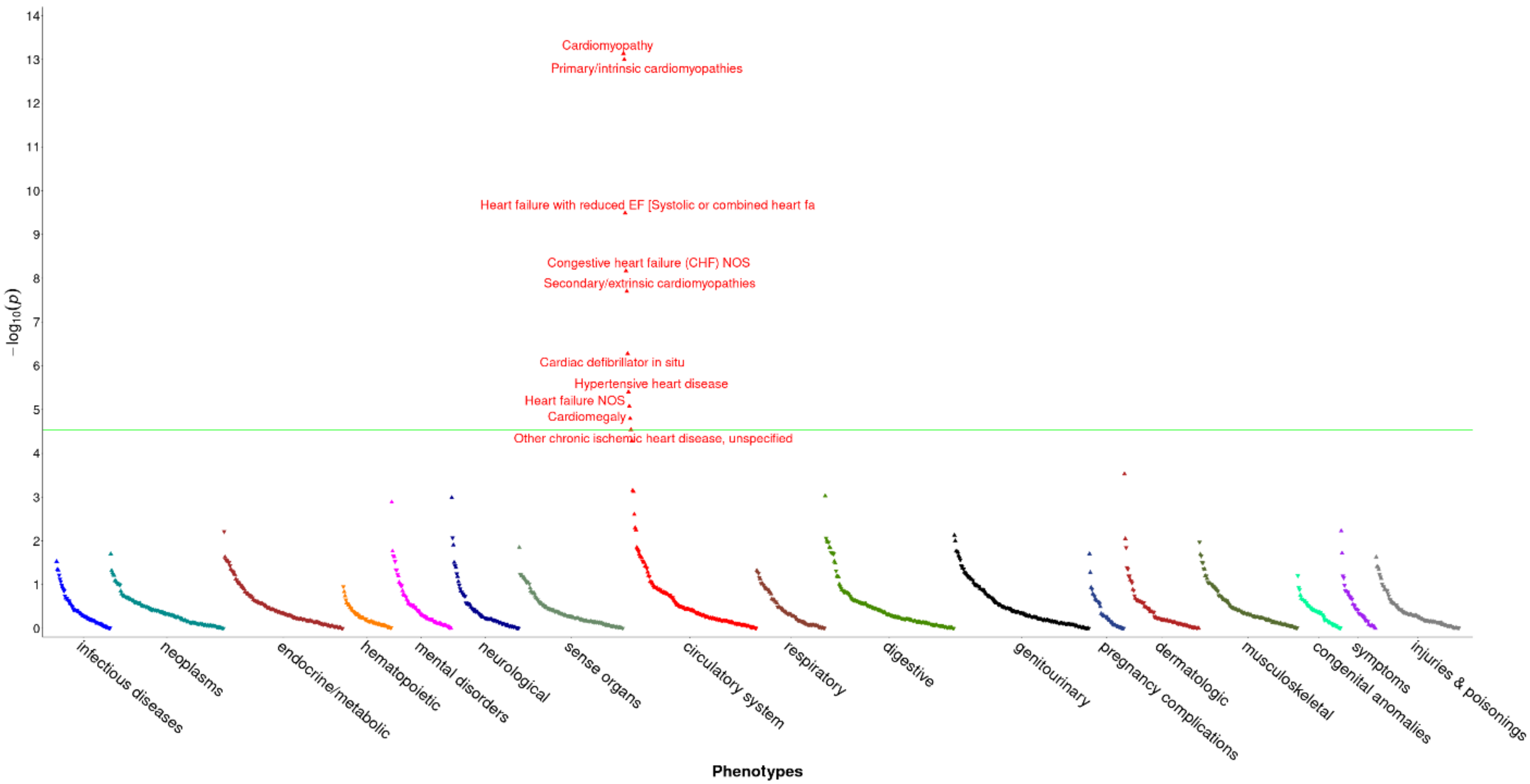

Furthermore, for notable risk factors—such as obesity, diabetes, hypertension, and lipid traits—could Mendelian Randomization (MR) be employed to estimate their causal effects on HFrEF and HFpEF, moving beyond simple genetic association?

Thank you for this suggestion. We have added the MR results, as shown below:

Methods:

Mendelian randomization (MR)

Based on the results from the European ancestry, MR was performed to explore the potential bi-directional causal associations between HFrEF, HFpEF and several risk factors, including coronary artery disease, type 2 diabetes, body mass index, high-density lipoprotein cholesterol, low-density lipoprotein cholesterol, total cholesterol, triglycerides, systolic blood pressure, diastolic blood pressure, and pulse pressure. The genetic instruments for the risk factors were selected from recent large-scale GWAS of European or European-dominant populations (PMID: 26343387; 30297969; 25673413; 24097068; 30224653).

The MR analysis was performed using the R package TwoSampleMR (PMID: 29846171), which incorporates MR-Egger model that accounts for potential pleiotropy, and inverse-variance weighted model. In addition, the MR-PRESSO (PMID: 29686387) (Mendelian Randomization Pleiotropy RESidual Sum and Outlier) method was used to detect and correct for pleiotropy, and MR analyses were conducted after removing genetic outliers that may introduce bias to the causal estimates.

Results:

Coronary artery disease, body mass index, low-density lipoprotein cholesterol, systolic blood pressure, and diastolic blood pressure showed strong causal effects on HFrEF, whereas high-density lipoprotein cholesterol and total cholesterol did not. Type 2 diabetes, triglycerides, and pulse pressure demonstrated potential causal effects for HFrEF. For HFpEF, coronary artery disease, body mass index, systolic blood pressure, diastolic blood pressure, and pulse pressure showed strong causal effects. Lipids did not demonstrate evidence of a causal effect. Type 2 diabetes showed a modest potential causal association, Tables S24A-S24B.

Table S24A. Mendelian Randomization for the Causal Effect of Risk Factors on HFrEF.

**Exposure**	**Model**	**N GIVs**	**OR (95%) for Binary Outcome**<br>**Beta (95% CI) for Continuous Outcome**	**P**	**Pleiotropy Test P**
**CAD**	MR Egger	52	1.49 (1.38, 1.62)	5.61e-13	0.8708
	IVW		1.5 (1.44, 1.57)	1.68e-78	
	MR Egger (MR-PRESSO)	52	1.49 (1.38, 1.62)	5.61e-13	0.8708
	IVW (MR-PRESSO)		1.5 (1.44, 1.57)	1.68e-78	
**T2D**	MR Egger	313	1.04 (0.98, 1.1)	0.220	0.0005
	IVW		1.14 (1.11, 1.17)	5.98e-19	
	MR Egger (MR-PRESSO)	308	1.04 (0.97, 1.11)	0.240	0.0051

	IVW (MR-PRESSO)		1.14 (1.11, 1.17)	7.05e-19	
BMI (per SD)	MR Egger	67	1.93 (1.41, 2.64)	1.07e-04	0.5403
	IVW		1.77 (1.55, 2.01)	1.01e-17	
	MR Egger (MR-PRESSO)	65	2.04 (1.56, 2.67)	2.44e-06	0.4038
	IVW (MR-PRESSO)		1.84 (1.64, 2.06)	5.77e-26	
HDL (per SD)	MR Egger	56	1.11 (0.96, 1.28)	0.177	0.0127
	IVW		0.96 (0.87, 1.06)	0.436	
	MR Egger (MR-PRESSO)	52	1.06 (0.95, 1.19)	0.271	0.1499
	IVW (MR-PRESSO)		1 (0.93, 1.08)	0.962	
LDL (per SD)	MR Egger	25	1.16 (1.03, 1.31)	0.0194	0.5975
	IVW		1.19 (1.1, 1.29)	6.81e-06	
	MR Egger (MR-PRESSO)	23	1.19 (1.02, 1.37)	0.0033	0.7801
	IVW (MR-PRESSO)		1.21 (1.11, 1.32)	2.40e-05	
TC (per SD)	MR Egger	35	1.06 (0.75, 1.5)	0.754	0.9783
	IVW		1.06 (0.93, 1.22)	0.391	
	MR Egger (MR-PRESSO)	34	1.08 (0.8, 1.46)	0.606	0.7032
	IVW (MR-PRESSO)		1.03 (0.91, 1.16)	0.672	
TG (per SD)	MR Egger	26	1.15 (1.01, 1.31)	0.0528	0.9180
	IVW		1.15 (1.06, 1.26)	0.0016	
	MR Egger (MR-PRESSO)	25	1.21 (1.07, 1.38)	6.98e-03	0.8836
	IVW (MR-PRESSO)		1.21 (1.1, 1.32)	3.05e-05	
SBP (per 10 mmHg)	MR Egger	244	1.27 (1.08, 1.49)	3.70e-03	0.8421
	IVW		1.29 (1.21, 1.37)	3.45e-15	
	MR Egger (MR-PRESSO)	241	1.42 (1.22, 1.66)	1.36e-05	0.3000
	IVW (MR-PRESSO)		1.32 (1.24, 1.4)	1.69e-19	
DBP (per 10 mmHg)	MR Egger	284	1.46 (1.14, 1.89)	3.56e-03	0.9444
	IVW		1.45 (1.3, 1.62)	2.10e-11	
	MR Egger (MR-PRESSO)	276	1.6 (1.28, 2.01)	5.34e-05	0.4414
	IVW (MR-PRESSO)		1.48 (1.35, 1.62)	4.62e-16	
PP (per 10 mmHg)	MR Egger	251	0.89 (0.68, 1.16)	0.378	0.0369
	IVW		1.15 (1.03, 1.29)	0.0127	
	MR Egger (MR-PRESSO)	244	0.94 (0.74, 1.19)	0.614	0.0637
	IVW (MR-PRESSO)		1.15 (1.05, 1.27)	4.19e-03	

CAD, coronary artery disease; T2D, type 2 diabetes; BMI, body mass index; HDL, high-density lipoprotein cholesterol; LDL, low-density lipoprotein cholesterol; TC, total cholesterol; TG, triglycerides; SBP, systolic blood pressure; DBP, diastolic blood pressure; PP, pulse pressure; MR, Mendelian randomization; IVW, inverse-variance weighted; PRESSO, pleiotropy residual sum and outlier; OR, odds ratio; CI, confidence interval; SD, standard deviation; GIVs, genetic instrumental variables.

Table S24B. Mendelian Randomization for the Causal Effect of Risk Factors on HFpEF.

**Exposure**	**Model**	**N GIVs**	**OR (95%)**	**P**	**Pleiotropy Test P**
CAD	MR Egger	52	1.16 (1.06, 1.27)	2.97e-03	0.4468
	IVW		1.19 (1.14, 1.25)	4.19e-13	
	MR Egger (MR-PRESSO)	52	1.16 (1.06, 1.27)	2.97e-03	0.4468
	IVW (MR-PRESSO)		1.19 (1.14, 1.25)	4.19e-13	
T2D	MR Egger	313	0.97 (0.92, 1.03)	0.338	1.96e-05
	IVW		1.08 (1.05, 1.11)	6.14e-09	
	MR Egger (MR-PRESSO)	309	0.95 (0.89, 1.02)	0.144	2.25e-05
	IVW (MR-PRESSO)		1.08 (1.05, 1.11)	1.11e-08	
BMI (per SD)	MR Egger	67	1.73 (1.36, 2.19)	2.41e-05	0.4714

	IVW		1.87 (1.69, 2.06)	7.40e-36	
	MR Egger (MR-PRESSO)	67	1.73 (1.36, 2.19)	2.41e-05	0.4714
	IVW (MR-PRESSO)		1.87 (1.69, 2.06)	7.40e-36	
**HDL (per SD)**	MR Egger	56	1.11 (1, 1.23)	0.0617	0.0012
	IVW		0.97 (0.9, 1.04)	0.379	
	MR Egger (MR-PRESSO)	54	1.08 (1, 1.17)	0.0543	0.0025
	IVW (MR-PRESSO)		0.98 (0.93, 1.04)	0.594	
**LDL (per SD)**	MR Egger	25	1.09 (0.96, 1.23)	0.211	0.7033
	IVW		1.07 (0.98, 1.16)	0.123	
	MR Egger (MR-PRESSO)	25	1.09 (0.96, 1.23)	0.211	0.7033
	IVW (MR-PRESSO)		1.07 (0.98, 1.16)	0.123	
**TC (per SD)**	MR Egger	35	1.18 (0.85, 1.62)	0.328	0.1536
	IVW		0.95 (0.83, 1.08)	0.401	
	MR Egger (MR-PRESSO)	35	1.18 (0.85, 1.62)	0.328	0.1536
	IVW (MR-PRESSO)		0.95 (0.83, 1.08)	0.401	
**TG (per SD)**	MR Egger	26	1.12 (0.96, 1.32)	0.175	0.4405
	IVW		1.07 (0.96, 1.19)	0.223	
	MR Egger (MR-PRESSO)	22	1.06 (0.94, 1.2)	0.362	0.7936
	IVW (MR-PRESSO)		1.05 (0.97, 1.14)	0.271	
**SBP (per 10 mmHg)**	MR Egger	244	1.17 (1.01, 1.36)	0.0429	0.5269
	IVW		1.22 (1.15, 1.3)	3.95e-11	
	MR Egger (MR-PRESSO)	244	1.17 (1.01, 1.36)	0.0429	0.5269
	IVW (MR-PRESSO)		1.22 (1.15, 1.3)	3.95e-11	
**DBP (per 10 mmHg)**	MR Egger	284	1.31 (1.07, 1.61)	9.54e-03	0.1579
	IVW		1.15 (1.05, 1.25)	1.97e-03	
	MR Egger (MR-PRESSO)	281	1.37 (1.13, 1.66)	1.69e-03	0.0732
	IVW (MR-PRESSO)		1.17 (1.07, 1.27)	3.40e-04	
**PP (per 10 mmHg)**	MR Egger	251	1.27 (1.05, 1.53)	0.0141	0.7629
	IVW		1.3 (1.21, 1.41)	2.07e-11	
	MR Egger (MR-PRESSO)	251	1.27 (1.05, 1.53)	0.0141	0.7629
	IVW (MR-PRESSO)		1.3 (1.21, 1.41)	2.07e-11	

CAD, coronary artery disease; T2D, type 2 diabetes; BMI, body mass index; HDL, high-density lipoprotein cholesterol; LDL, low-density lipoprotein cholesterol; TC, total cholesterol; TG, triglycerides; SBP, systolic blood pressure; DBP, diastolic blood pressure; PP, pulse pressure; MR, Mendelian randomization; IVW, inverse-variance weighted; PRESSO, pleiotropy residual sum and outlier; OR, odds ratio; CI, confidence interval; SD, standard deviation; GIVs, genetic instrumental variables.

4. European vs. African ancestry differences

For the β-plots in Figures 3 and 5, adding allele frequency comparisons between European and African ancestries alongside the effect sizes would allow readers to interpret both dimensions simultaneously.

We have revised this figure to present the original scatter plot comparing effect sizes as Panel A, and we have added a new Panel B comparing allele frequencies. This is the updated figure in the revised manuscript, as shown below:

Revised Figure 2. Scatter Plot for Comparison of the Estimates for the Genome-wide Significant Loci for the Meta-analysis of GWAS on HFrEF Among European Ancestry and African Ancestry. A: Comparison of Effect Sizes. Pearson Correlation Coefficient = 0.27 (95% CI -0.09,

0.57). Loci with Heterogeneity are Labeled. B: Comparison of Risk Allele Frequency. African-specific Loci are Labeled.

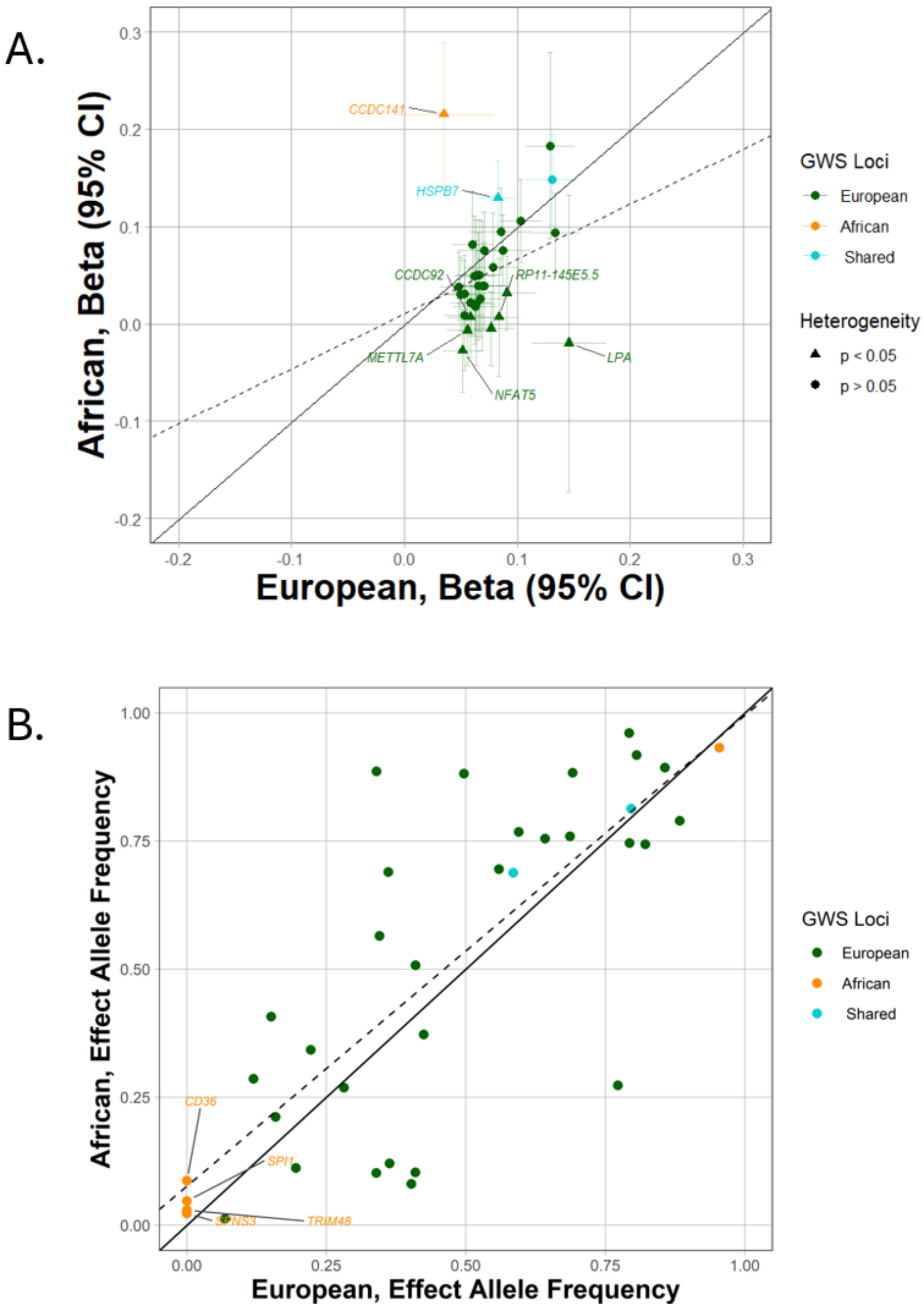

Additionally, please include relevant literature supporting how observed allele frequency differences—combined with environmental and epidemiological context—strengthen the study's conclusions.

We have added discussion of ancestry-related differences in allele frequencies and their relevance to cardiovascular diseases in the broader epidemiological context, as shown below:

Results:

"Of the 7 HFrEF GWS loci in the African ancestry, 2 loci showed small or null effects in the European ancestry, and 4 loci (*CD36*, *SPI1*, *TRIM48*, and *SPNS3)* were rare in the European ancestry, with MAF ≤ 0.05%, Table 2 & Figures 2-3. Two other loci, *CSK* (effect allele frequency 0.34 in Europeans vs. 0.89 in Africans) and *PSRC1* (0.77 in Europeans vs. 0.27 in Africans), demonstrated allele frequency differences of ⩾50% between European and African populations."

Discussion:

"The African-specific HFrEF locus *CD36* has an established role in mediating a range of critical cellular processes. It acts as a multifunctional receptor, plays a role in lipid handling, platelet function, inflammation, angiogenesis and fatty acid transport within the cardiovascular system (PMID: 11560944). *CD36* acts as a long-chain fatty acid transporter in cardiac myocytes and has function as a receptor for various ligands in endothelial cells, with a specific emphasis on its role in the context of diabetic cardiomyopathy (PMID: 19471024; 41174181). *SPI1* is implicated in cardiovascular processes due to its role in gene expression regulation and impacts pathways related to inflammation and cardiac remodeling (PMID: 35559389). The observation that the 4 HFrEF loci, *CD36*, *SPI1*, *TRIM48*, and *SPNS3* were rare within the European population provides valuable insights into the genetic diversity of HFrEF. Loci such as *PSRC1* and *CSK* highlight alleles with cross-ancestry relevance but marked differences in population frequencies. Variants at *PSRC1* have been consistently associated with lipid metabolism and coronary artery disease across multi-ethnic cohorts (PMID: 17634449). Similarly, *CSK* variants influence blood pressure regulation and hypertension risk, with stronger effects in non-European populations where the risk allele is more common (PMID: 34594039)."

5. Variant-to-Gene mapping

While the manuscript employs TWAS for gene prioritization, were complementary integrative platforms such as Open Targets considered? Since different approaches prioritize genes differently, presenting independent results from multiple methods prior to integration would improve the robustness of gene-level inference.

In response to the reviewer's suggestion, we evaluated the tractability of our TWAS-prioritized genes using Open Targets. Several genes show strong support for therapeutic targeting. *FTO* has evidence from advanced clinical studies. Additional genes, including *CLCNKA, PHACTR1, ATF1, MAP3K7, BPTF, NPC1, CAMK2D*, and *BDNF*, have preclinical or experimental support for druggability. These findings, summarized as a new table in the revised manuscript (shown below), provide evidence supporting the relevance of these loci and highlight their potential as therapeutic targets in HF. We have added the following statements in the revised manuscript:

Methods:

Therapeutic target profile

We queried TWAS-prioritized genes in Open Targets (release 17 September 2025, PMID: 33196847) to evaluate their therapeutic tractability. Loci that reached genome-wide significance and had a Bonferroni-corrected p value < 0.05 in TWAS were assessed for evidence of tractability, including targets of approved drugs, drugs in clinical trials, preclinical small molecules, or predicted druggability by small molecules or antibodies.

Results:

TWAS-prioritized genes showed varying levels of tractability in Open Targets. *FTO* exhibited evidence from advanced clinical studies, while *CLCNKA, PHACTR1, ATF1, MAP3K7, BPTF, NPC1, CAMK2D,* and *BDNF* had preclinical or experimental support for druggability, Table S23A.

Table S23A. Target Tractability Assessment for the TWAS-prioritized Genes Using Open Targets.

HF Type	Gene	Small Molecule	Antibody	PROTAC
HFrEF	*CLCNKA*	High-Quality Pocket; Druggable Family	GO CC high conf; UniProt loc med conf; UniProt SigP or TMHMM	-
	MLIP	-	UniProt loc med conf; GO CC med conf; Human Protein Atlas loc	Half-life Data
	PHACTR1	Structure with Ligand	-	Half-life Data
	ATF1	Druggable Family	-	UniProt Ubiquitination; Database Ubiquitination; Half-life Data; Small Molecule Binder
	MAP3K7	Structure with Ligand; High-Quality Ligand; Druggable Family	UniProt loc high conf; GO CC high conf; Human Protein Atlas loc	Literature; UniProt Ubiquitination; Database Ubiquitination; Half-life Data; Small Molecule Binder
	CCDC136	-	UniProt SigP or TMHMM; Human Protein Atlas loc	Database Ubiquitination; Half-life Data
	BPTF	Structure with Ligand; High-Quality Ligand; High-Quality Pocket	-	UniProt Ubiquitination; Database Ubiquitination; Half-life Data; Small Molecule Binder
	PGAP3	-	UniProt SigP or TMHMM; Human Protein Atlas loc	-
	CELSR2	-	UniProt loc med conf; UniProt SigP or TMHMM; GO CC med conf	Database Ubiquitination; Half-life Data
HFpEF	*FTO*	Advanced Clinical; Structure with Ligand; High-Quality Ligand	-	Database Ubiquitination; Half-life Data; Small Molecule Binder
All-cause HF	*CLCNKA*	High-Quality Pocket; Druggable Family	GO CC high conf; UniProt loc med conf; UniProt SigP or TMHMM	-
	MIA3	-	UniProt SigP or TMHMM	Database Ubiquitination; Half-life Data
	MLIP	-	UniProt loc med conf; GO CC med conf; Human Protein Atlas loc	Half-life Data
	GTF2I	-	-	UniProt Ubiquitination;

				Database Ubiquitination; Half-life Data
	HSD17B12	-	UniProt SigP or TMHMM; GO CC med conf	Database Ubiquitination; Half-life Data
	ABHD17C	-	GO CC high conf	Database Ubiquitination; Half-life Data
	NPC1	Structure with Ligand; High-Quality Ligand; Med-Quality Pocket; Druggable Family	GO CC high conf; UniProt SigP or TMHMM	Database Ubiquitination; Half-life Data; Small Molecule Binder
	PHACTR1	Structure with Ligand		Half-life Data
	CCDC136	-	UniProt SigP or TMHMM; Human Protein Atlas loc	Database Ubiquitination; Half-life Data
	CRTC1	-	GO CC high conf	Database Ubiquitination; Half-life Data
	SPATS2L	-	-	Database Ubiquitination; Half-life Data
	STRN	-	UniProt loc med conf	Database Ubiquitination; Half-life Data
	CAMK2D	Structure with Ligand; High-Quality Ligand; High-Quality Pocket; Druggable Family	UniProt loc high conf; GO CC med conf; Human Protein Atlas loc	Database Ubiquitination; Half-life Data; Small Molecule Binder
	SYNPO2L	-	-	Database Ubiquitination
	BPTF	Structure with Ligand; High-Quality Ligand; High-Quality Pocket	-	UniProt Ubiquitination; Database Ubiquitination; Half-life Data; Small Molecule Binder
	BDNF	-	UniProt loc high conf; GO CC high conf; UniProt SigP or TMHMM	Database Ubiquitination; Small Molecule Binder
	FTO	Advanced Clinical; Structure with Ligand; High-Quality Ligand	-	Database Ubiquitination; Half-life Data; Small Molecule Binder
	HLF	-	-	Database Ubiquitination
	PSRC1	-	-	-

6. Fine-mapping with Functional Prioritization

Beyond DEPICT's tissue prioritization (based on gene expression), could epigenomic data—such as ATAC-seq, ChIP-seq, or single-nucleus RNA-seq (snRNA-seq)—be integrated to further prioritize causal genes or cell types at novel loci?

We appreciate the reviewer's suggestion to integrate epigenomic data such as ATAC-seq, ChIP-seq, or single-nucleus RNA-seq to refine causal gene and cell-type prioritization. While ATAC-seq and snRNA-seq data are not yet broadly available across large panels of heart tissues, we incorporated orthogonal epigenomic annotations from the Roadmap Epigenomics and ENCODE projects, which include genome-wide ChIP-seq profiles of histone modifications. We integrated epigenomic annotations from Roadmap and ENCODE functional annotation to prioritize genes and cell types at the genome-significant loci. Specifically, we overlaid GWAS lead variants with ChromHMM chromatin states (15-core model) across cardiac tissues, including fetal heart, right atrium, left ventricle, right ventricle, and aorta, enabling SNP-level classification as promoter/TSS, enhancer, transcribed, repressed, or quiescent. This provides orthogonal

functional evidence that many HF signals reside in active regulatory elements in heart tissues. We have added the following statements and supplemental tables to the revised manuscript:

Methods:

We additionally performed functional annotation of genome-wide significant loci using chromatin state data from the Roadmap Epigenomics (PMID: 25693563) and ENCODE (PMID: 22955616). Specifically, we overlaid GWAS sentinel SNPs with the 15-core ChromHMM model (PMID: 22373907) across cardiac tissues, including fetal heart, right atrium, left ventricle, right ventricle, and aorta. Each variant was annotated with regulatory states, enabling SNP-level classification into promoter, enhancer, transcribed, or repressed elements.

Results:

Integration of epigenomic annotations revealed that multiple HF loci mapped to active regulatory regions within cardiac tissues, providing orthogonal support for their functional relevance. For HFrEF, several loci localized to enhancer or promoter states, including *CDKN1A* in Active TSS across atrial and ventricular tissues, *ACTN2* overlapping bivalent and promoter states, and *TTN* within genic enhancers, Table S20A. For HFpEF, *ARSG* was consistently annotated as enhancers across cardiac tissues, Table S20B. For all-cause HF, regulatory enrichment was observed at *NKX2-5*, *SYNPO2L* and *BAG*3 in enhancer regions, Table S20C.

Table S20A. Chromatin State Annotations for the 46 Genome-wide Significant Loci of HFrEF Among Multi-ancestry Cohort.

**Chr.**	**Position (hg19)**	**Gene**	**rsID**	**Fetal Heart**	**Right Atrium**	**Left Ventricle**	**Right Ventricle**
1	16348412	*CLCNKA*	rs945425	Enhancers	Weak transcription	Enhancers	Enhancers
1	61883027	*NFIA*	rs1997998	Weak transcription	Quiescent/Low	Weak transcription	Weak transcription
1	109817590	*CELSR2*	rs12740374	Weak transcription	Weak transcription	Weak transcription	Weak transcription
1	162024987	*NOS1AP*	rs10429888	Quiescent/Low	Quiescent/Low	Quiescent/Low	Quiescent/Low
1	236839990	*ACTN2*	rs524517	Weak transcription	Weak transcription	Weak Repressed PolyComb	Quiescent/Low
2	11571966	*E2F6*	rs34514099	Weak transcription	Quiescent/Low	Weak transcription	Quiescent/Low
2	37233937	*HEATR5B*	rs62133082	Weak transcription	Quiescent/Low	Strong transcription	Weak transcription
2	179595117	*TTN*	rs17076	Genic enhancers	Weak transcription	Genic enhancers	Weak transcription
3	12842223	*CAND2*	rs4642101	Weak transcription	Quiescent/Low	Enhancers	Weak transcription
3	14274451	-	rs6807275	Enhancers	Weak transcription	Enhancers	Weak transcription
3	49622632	*BSN*	rs34989545	Quiescent/Low	Quiescent/Low	Quiescent/Low	Quiescent/Low
3	158305894	*MLF1*	rs2082838	Weak transcription	Quiescent/Low	Weak transcription	Weak transcription

4	111664158	-	rs17042098	Quiescent/Low	Quiescent/Low	Quiescent/Low	Quiescent/Low
5	138746334	*DNAJC18*	rs11242464	Enhancers	Quiescent/Low	Enhancers	Weak transcription
6	12903957	*PHACTR1*	rs9349379	Weak Repressed PolyComb	Quiescent/Low	Quiescent/Low	Quiescent/Low
6	36646768	*CDKN1A*	rs146170154	Flanking Active TSS	Active TSS	Active TSS	Active TSS
6	53971921	*MLIP*	rs1407229	Enhancers	Weak transcription	Weak transcription	Weak transcription
6	79749166	*PHIP*	rs66577603	Weak transcription	Quiescent/Low	Weak transcription	Quiescent/Low
6	91303512	*MAP3K7*	rs282064	Weak Repressed PolyComb	Weak Repressed PolyComb	Weak Repressed PolyComb	Weak Repressed PolyComb
6	161010118	*LPA*	rs10455872	Quiescent/Low	Quiescent/Low	Quiescent/Low	Quiescent/Low
7	80300449	*CD36*	rs3211938	Weak transcription	Quiescent/Low	Strong transcription	Weak transcription
7	128455916	*CCDC136*	rs2270590	Weak transcription	Weak transcription	Weak transcription	Quiescent/Low
8	125861374	-	rs7461129	Enhancers	Enhancers	Flanking Active TSS	Weak transcription
9	22124744	*RP11-145E5.5*	rs4977575	Quiescent/Low	Quiescent/Low	Quiescent/Low	Quiescent/Low
9	35723234	*TLN1*	rs10814273	Strong transcription	Weak transcription	Strong transcription	Strong transcription
9	136151806	-	rs600038	Repressed PolyComb	Quiescent/Low	Weak Repressed PolyComb	Weak Repressed PolyComb
10	44750414	-	rs485838	Weak Repressed PolyComb	Quiescent/Low	Quiescent/Low	Quiescent/Low
10	49528021	*MAPK8*	rs148307915	Quiescent/Low	Weak transcription	Weak transcription	Weak transcription
10	75585307	*CAMK2G*	rs2242254	Quiescent/Low	Quiescent/Low	Weak transcription	Strong transcription
10	121424815	*BAG3*	rs375034445	Enhancers	Weak transcription	Weak transcription	Weak transcription
11	47379410	*SPI1*	rs113123267	Weak transcription	Weak transcription	Quiescent/Low	Genic enhancers
11	55047396	*TRIM48*	rs111786504	Quiescent/Low	Quiescent/Low	Quiescent/Low	Quiescent/Low
11	56329980	*OR5M11*	rs60504091	Quiescent/Low	Quiescent/Low	Quiescent/Low	Quiescent/Low
11	100942792	*PGR*	rs658286	Quiescent/Low	Quiescent/Low	Weak transcription	Quiescent/Low
12	51187290	*ATF1*	rs11169562	Weak transcription	Quiescent/Low	Weak transcription	Weak transcription
12	124410529	*DNAH10; CCDC92*	rs4575361	Quiescent/Low	Weak transcription	Quiescent/Low	Weak transcription
15	75086534	*CSK*	rs62006566	Weak transcription	Weak transcription	Weak transcription	Weak transcription
16	53834607	*FTO*	rs7188250	Quiescent/Low	Weak transcription	Quiescent/Low	Quiescent/Low
17	2205923	*SMG6*	rs2224770	Enhancers	Active TSS	Weak transcription	Weak transcription
17	7460957	*TNFSF12; TNFSF12-TNFSF13*	rs9907657	Quiescent/Low	Enhancers	Enhancers	Enhancers

17	37841211	*PGAP3*	rs2517953	Weak transcription	Quiescent/Low	Weak transcription	Weak transcription
17	64318357	*PRKCA*	rs9912468	Quiescent/Low	Quiescent/Low	Weak transcription	Quiescent/Low
17	65893289	*BPTF*	rs145606454	Weak transcription	Quiescent/Low	Strong transcription	Weak transcription
18	46516424	-	rs7229491	Enhancers	Enhancers	Weak transcription	Weak transcription
21	30540165	*MAP3K7CL*	rs56968346	Quiescent/Low	Weak transcription	Quiescent/Low	Quiescent/Low
22	24166788	*SMARCB1*	rs9608201	Enhancers	Weak transcription	Genic enhancers	Genic enhancers

Table S20B. Chromatin State Annotations for the 3 Genome-wide Significant Loci of HFpEF Among Multi-ancestry Cohort.

**Chr.**	**Position (hg19)**	**Gene**	**rsID**	**Fetal Heart**	**Right Atrium**	**Left Ventricle**	**Right Ventricle**
16	53803223	*FTO*	rs62048402	Enhancers	Weak transcription	Weak transcription	Enhancers
17	66382647	*ARSG*	rs75716431	Enhancers	Enhancers	Enhancers	Enhancers
21	18617840	-	rs113752382	Quiescent/Low	Quiescent/Low	Quiescent/Low	Quiescent/Low

Table S20C. Chromatin State Annotations for the 136 Genome-wide Significant Loci of All-cause HF Among Multi-ancestry Cohort.

**Chr.**	**Position (hg19)**	**Gene**	**rsID**	**Fetal Heart**	**Right Atrium**	**Left Ventricle**	**Right Ventricle**	**Aorta**
1	10796547	*CASZ1*	rs17035646	Enhancers	Weak transcription	Enhancers	Enhancers	Quiescent/Low
1	16348083	*CLCNKA*	rs2009371	Enhancers	Weak transcription	Weak transcription	Enhancers	Quiescent/Low
1	50975227	*FAF1*	rs116626164	Quiescent/Low	Quiescent/Low	Weak transcription	Quiescent/Low	Weak transcription
1	56990753	*PPAP2B*	rs72664341	Weak transcription	Enhancers	Genic enhancers	Weak transcription	Strong transcription
1	61886046	*NFIA*	rs1997997	Enhancers	Weak transcription	Weak transcription	Weak transcription	Weak transcription
1	78450517	*DNAJB4; GIPC2*	rs34517439	Enhancers	Weak transcription	Weak transcription	Weak transcription	Weak transcription
1	93237726	*EVI5*	rs35054144	Quiescent/Low	Quiescent/Low	Weak transcription	Weak transcription	Quiescent/Low
1	109822166	*PSRC1*	rs599839	Weak transcription	Weak transcription	Weak transcription	Weak transcription	Quiescent/Low
1	113046879	*WNT2B*	rs3790604	Repressed PolyComb	Quiescent/Low	Weak Repressed PolyComb	Weak Repressed PolyComb	Quiescent/Low
1	114173410	*MAGI3*	rs1230666	Quiescent/Low	Quiescent/Low	Weak transcription	Weak transcription	Weak transcription
1	190319316	*BRINP3*	rs61818779	Quiescent/Low	Quiescent/Low	Quiescent/Low	Quiescent/Low	Quiescent/Low
1	222806218	*MIA3*	rs17163345	Weak transcription	Quiescent/Low	Strong transcription	Strong transcription	Strong transcription

1	236852282	*ACTN2*	rs12724121	Weak transcription	Active TSS	Bivalent/Poised TSS	Active TSS	Enhancers
2	630070	-	rs66906321	Quiescent/Low	Quiescent/Low	Weak Repressed PolyComb	Weak Repressed PolyComb	Quiescent/Low
2	6147956	-	rs1358134	Quiescent/Low	Quiescent/Low	Weak Repressed PolyComb	Quiescent/Low	Quiescent/Low
2	11563960	*E2F6*	rs12995996	Weak transcription	Quiescent/Low	Weak transcription	Quiescent/Low	Weak transcription
2	26990273	*SLC35F6; CENPA*	-	Weak transcription	Enhancers	Enhancers	Enhancers	Weak transcription
2	36655721	*CRIM1*	rs28705335	Weak transcription	Weak transcription	Weak transcription	Weak transcription	Weak transcription
2	37192866	*STRN*	rs3770770	Active TSS	Active TSS	Active TSS	Active TSS	Active TSS
2	59302877	-	rs887912	Quiescent/Low	Weak Repressed PolyComb	Quiescent/Low	Quiescent/Low	Quiescent/Low
2	76914832	-	rs13389483	Quiescent/Low	Quiescent/Low	Quiescent/Low	Quiescent/Low	Quiescent/Low
2	86764004	*CHMP3; RNF103-CHMP3*	rs4832298	Weak transcription	Quiescent/Low	Weak transcription	Weak transcription	Weak transcription
2	148770274	*ORC4*	rs13033664	Quiescent/Low	Weak transcription	Weak transcription	Quiescent/Low	Quiescent/Low
2	201171651	*SPATS2L*	rs74853338	Weak transcription	Active TSS	Active TSS	Active TSS	Active TSS
3	12841804	*CAND2*	rs7650482	Enhancers	Enhancers	Enhancers	Weak transcription	Weak transcription
3	25121444	-	rs12629972	Quiescent/Low	Quiescent/Low	Quiescent/Low	Quiescent/Low	Quiescent/Low
3	27548900	*SLC4A7*	rs820430	Quiescent/Low	Quiescent/Low	Quiescent/Low	Quiescent/Low	Quiescent/Low
3	43930155	-	rs11710802	Weak Repressed PolyComb	Weak Repressed PolyComb	Weak Repressed PolyComb	Weak Repressed PolyComb	Quiescent/Low
3	49845006	*UBA7*	rs58339610	Enhancers	Enhancers	Enhancers	Enhancers	Weak transcription
3	53553923	*CACNA1D*	rs35593046	Quiescent/Low	Weak Repressed PolyComb	Quiescent/Low	Weak Repressed PolyComb	Quiescent/Low
3	135800409	*PPP2R3A*	rs34330586	Weak transcription	Quiescent/Low	Strong transcription	Weak transcription	Weak transcription
3	168853335	*MECOM*	rs77463690	Quiescent/Low	Weak transcription	Quiescent/Low	Quiescent/Low	Weak transcription
3	184000619	*EIF2B5; ECE2*	rs4912532	Weak Repressed PolyComb	Weak transcription	Weak Repressed PolyComb	Weak Repressed PolyComb	Weak Repressed PolyComb
4	25408838	*ANAPC4*	rs34811474	Weak transcription	Quiescent/Low	Strong transcription	Weak transcription	Weak transcription
4	26805393	-	rs7662342	Enhancers	Quiescent/Low	Enhancers	Quiescent/Low	Quiescent/Low
4	45181334	-	rs12507026	Weak transcription	Quiescent/Low	Quiescent/Low	Quiescent/Low	Quiescent/Low
4	67879681	-	rs1383722	Quiescent/Low	Quiescent/Low	Quiescent/Low	Quiescent/Low	Quiescent/Low
4	77372923	*SHROOM3*	rs1398018	Quiescent/Low	Quiescent/Low	Weak Repressed PolyComb	Quiescent/Low	Quiescent/Low

4	111701433	*PITX2*	rs59788391	Weak Repressed PolyComb	Quiescent/Low	Quiescent/Low	Quiescent/Low	Quiescent/Low
4	114384328	*CAMK2D*	rs17620390	Weak transcription	Quiescent/Low	Weak transcription	Weak transcription	Weak transcription
4	140884723	*MAML3*	rs3106209	Quiescent/Low	Quiescent/Low	Weak transcription	Quiescent/Low	Weak transcription
5	64293570	*CWC27*	rs1309540	Weak transcription	Quiescent/Low	Weak transcription	Weak transcription	Weak transcription
5	95857763	*CAST*	rs12514158	Quiescent/Low	Quiescent/Low	Quiescent/Low	Quiescent/Low	Quiescent/Low
5	137012171	*KLHL3*	rs11745324	Weak transcription	Weak transcription	Weak transcription	Weak transcription	Strong transcription
5	138773874	*DNAJC18*	rs11956224	Weak transcription	Weak transcription	Weak transcription	Weak transcription	Weak transcription
5	142752480	*NR3C1*	rs61752263	Weak transcription	Weak transcription	Weak transcription	Weak transcription	Weak transcription
5	172670745	*NKX2-5*	rs6891790	Active TSS	Active TSS	Bivalent/Poised TSS	Active TSS	Heterochromatin
5	173307137	*CPEB4*	rs6885658	Weak transcription	Weak transcription	Weak transcription	Weak transcription	Quiescent/Low
6	12903957	*PHACTR1*	rs9349379	Weak Repressed PolyComb	Quiescent/Low	Quiescent/Low	Quiescent/Low	Weak transcription
6	22598259	*HDGFL1*	rs7766436	Quiescent/Low	Quiescent/Low	Quiescent/Low	Quiescent/Low	Quiescent/Low
6	34683635	*C6orf106*	rs6938239	Quiescent/Low	Quiescent/Low	Quiescent/Low	Quiescent/Low	Quiescent/Low
6	36647289	*CDKN1A*	rs3176326	Active TSS	Transcr. at gene 5' and 3'	Active TSS	Active TSS	Active TSS
6	40383533	*LRFN2*	rs62395884	Weak Repressed PolyComb	Weak transcription	Weak transcription	Weak transcription	Quiescent/Low
6	43345803	*ZNF318*	rs7742789	Quiescent/Low	Quiescent/Low	Quiescent/Low	Quiescent/Low	Quiescent/Low
6	50788778	*TFAP2B*	rs3798519	Repressed PolyComb	Weak Repressed PolyComb	Repressed PolyComb	Weak Repressed PolyComb	Weak Repressed PolyComb
6	54005043	*MLIP*	rs816373	Weak transcription	Quiescent/Low	Weak transcription	Weak transcription	Quiescent/Low
6	79789473	*PHIP*	rs9343870	Flanking Active TSS	Active TSS	Quiescent/Low	Active TSS	Quiescent/Low
6	100600097	-	rs17789218	Quiescent/Low	Quiescent/Low	Quiescent/Low	Quiescent/Low	Quiescent/Low
6	161010118	*LPA*	rs10455872	Quiescent/Low	Quiescent/Low	Quiescent/Low	Quiescent/Low	Heterochromatin
7	12283787	*TMEM106B*	rs1990622	Quiescent/Low	Quiescent/Low	Quiescent/Low	Quiescent/Low	Quiescent/Low
7	22768124	*IL6*	rs1474347	Quiescent/Low	Active TSS	Quiescent/Low	Active TSS	Weak Repressed PolyComb
7	27243221	*HOXA13*	rs2023843	Repressed PolyComb	Weak transcription	Repressed PolyComb	Weak transcription	Weak Repressed PolyComb
7	32615191	*AVL9*	rs77413112	Weak transcription	Weak transcription	Strong transcription	Weak transcription	Weak transcription
7	74134911	*GTF2I*	rs35005436	Weak transcription	Quiescent/Low	Strong transcription	Weak transcription	Weak transcription
7	76654895	*UPK3B*	rs200944075	Quiescent/Low	Quiescent/Low	Quiescent/Low	Quiescent/Low	Quiescent/Low
7	92265798	*CDK6*	rs66939011	Weak transcription	Weak transcription	Enhancers	Weak transcription	Weak transcription

7	116191301	*CAV1*	rs11773845	Enhancers	Enhancers	Strong transcription	Enhancers	Weak transcription
7	128465450	*CCDC136*	rs60324735	Weak transcription	Weak transcription	Enhancers	Quiescent/Low	Quiescent/Low
7	150661409	*KCNH2*	rs7789146	Enhancers	Genic enhancers	Enhancers	Enhancers	Weak Repressed PolyComb
7	157040642	*UBE3C*	rs1182384	Weak transcription	Weak transcription	Weak transcription	Strong transcription	Weak transcription
8	19830921	*LPL*	rs10096633	Quiescent/Low	Quiescent/Low	Quiescent/Low	Quiescent/Low	Quiescent/Low
8	105978368	*RP11-127H5.1*	rs11774829	Quiescent/Low	Quiescent/Low	Quiescent/Low	Quiescent/Low	Quiescent/Low
8	125859850	-	rs34866937	Enhancers	Enhancers	Enhancers	Enhancers	Quiescent/Low
9	22124504	*RP11-145E5.5*	rs1333047	Quiescent/Low	Quiescent/Low	Quiescent/Low	Heterochromatin	Weak Repressed PolyComb
9	97619090	*C9orf3*	rs10993372	Quiescent/Low	Weak transcription	Quiescent/Low	Weak transcription	Weak transcription
9	131042734	*SWI5*	rs2270204	Weak transcription	Weak transcription	Weak transcription	Weak transcription	Weak transcription
9	136151806	*SURF6*	rs600038	Repressed PolyComb	Quiescent/Low	Weak Repressed PolyComb	Weak Repressed PolyComb	Weak Repressed PolyComb
10	22298641	*DNAJC1*	rs4328127	Quiescent/Low	Quiescent/Low	Quiescent/Low	Quiescent/Low	Quiescent/Low
10	75406912	*SYNPO2L*	rs34163229	Flanking Active TSS	Enhancers	Enhancers	Flanking Active TSS	Quiescent/Low
10	79636506	*DLG5*	rs67382505	Weak transcription	Weak transcription	Weak transcription	Weak transcription	Weak transcription
10	82203663	*TSPAN14*	rs4934169	Enhancers	Weak transcription	Weak transcription	Weak transcription	Quiescent/Low
10	91003419	*LIPA*	rs1332329	Quiescent/Low	Quiescent/Low	Weak transcription	Quiescent/Low	Strong transcription
10	104296418	*SUFU*	rs79159857	Quiescent/Low	Weak transcription	Weak transcription	Weak transcription	Weak transcription
10	121426884	*BAG3*	rs17617337	Enhancers	Enhancers	Enhancers	Enhancers	Weak transcription
11	1887068	*LSP1*	rs569550	Weak Repressed PolyComb	Weak transcription	Weak transcription	Enhancers	Quiescent/Low
11	10335700	*AMPD3*	rs1450265	Quiescent/Low	Quiescent/Low	Weak transcription	Quiescent/Low	Quiescent/Low
11	16286183	*SOX6*	rs10741694	Quiescent/Low	Quiescent/Low	Quiescent/Low	Quiescent/Low	Quiescent/Low
11	27724745	*BDNF*	rs988748	Repressed PolyComb	Weak Repressed PolyComb	Weak Repressed PolyComb	Weak Repressed PolyComb	Quiescent/Low
11	43633645	*HSD17B12*	rs7936836	Quiescent/Low	Weak Repressed PolyComb	Weak Repressed PolyComb	Quiescent/Low	Weak Repressed PolyComb
11	47715574	*AGBL2*	rs2904129	Quiescent/Low	Quiescent/Low	Quiescent/Low	Quiescent/Low	Quiescent/Low
11	49185414	*FOLH1*	rs58786959	Quiescent/Low	Quiescent/Low	Quiescent/Low	Quiescent/Low	Heterochromatin
12	6704169	*CHD4*	rs73047807	Strong transcription	Quiescent/Low	Strong transcription	Strong transcription	Strong transcription
12	14410485	-	rs17221259	Enhancers	Active TSS	Weak transcription	Quiescent/Low	Active TSS
12	107349294	*C12orf23*	rs2287163	Weak transcription	Active TSS	Active TSS	Active TSS	Active TSS

12	112007756	*ATXN2*	rs653178	Weak transcription	Enhancers	Enhancers	Enhancers	Enhancers
12	115552437	-	rs35444	Weak Repressed PolyComb	Quiescent/Low	Quiescent/Low	Quiescent/Low	Quiescent/Low
12	123339712	*HIP1R*	rs2292138	Quiescent/Low	Weak transcription	Weak transcription	Strong transcription	Weak transcription
12	125312425	*SCARB1*	rs10846744	Quiescent/Low	Enhancers	Quiescent/Low	Enhancers	Quiescent/Low
13	111049623	*COL4A2*	rs9515203	Enhancers	Enhancers	Enhancers	Active TSS	Weak transcription
14	33308791	*AKAP6*	rs35336096	Quiescent/Low	Quiescent/Low	Quiescent/Low	Quiescent/Low	Weak transcription
14	69765644	*GALNT16*	rs1890942	Quiescent/Low	Enhancers	Enhancers	Enhancers	Quiescent/Low
15	36381553	-	rs11636009	Quiescent/Low	Quiescent/Low	Quiescent/Low	Quiescent/Low	Quiescent/Low
15	77713586	*HMG20A*	rs34591043	Active TSS	Active TSS	Active TSS	Active TSS	Active TSS
15	78761414	*IREB2*	rs17484235	Weak transcription	Quiescent/Low	Weak transcription	Weak transcription	Weak transcription
15	81018587	*ABHD17C*	rs7174250	Weak transcription	Weak transcription	Weak transcription	Quiescent/Low	Weak transcription
15	85329421	*ZNF592*	rs12906348	Weak transcription	Enhancers	Genic enhancers	Enhancers	Genic enhancers
16	24793741	*TNRC6A*	rs2343606	Weak transcription	Quiescent/Low	Strong transcription	Strong transcription	Genic enhancers
16	53803223	*FTO*	rs62048402	Enhancers	Weak transcription	Weak transcription	Enhancers	Weak transcription
16	69730426	*NFAT5*	rs1437134	Weak transcription	Weak transcription	Weak transcription	Weak transcription	Weak transcription
16	73048367	*ZFHX3*	rs67329386	Weak transcription	Weak transcription	Weak transcription	Weak transcription	Weak transcription
16	75456423	*CFDP1; RP11-77K12.1*	rs11648176	Weak transcription	Weak transcription	Weak transcription	Weak transcription	Weak transcription
16	81525204	*CMIP*	rs62043959	Weak transcription	Enhancers	Enhancers	Weak transcription	Quiescent/Low
17	1256074	*YWHAE*	rs75543711	Weak transcription	Quiescent/Low	Strong transcription	Strong transcription	Strong transcription
17	2203453	*SMG6*	rs216193	Weak transcription	Weak transcription	Strong transcription	Weak transcription	Strong transcription
17	7498271	*FXR2*	rs72827590	Weak transcription	Weak transcription	Strong transcription	Strong transcription	Strong transcription
17	15828161	*ADORA2B*	rs2535599	Quiescent/Low	Quiescent/Low	Quiescent/Low	Quiescent/Low	Weak Repressed PolyComb
17	19281828	*MAPK7*	rs2233072	Active TSS	Active TSS	Active TSS	Active TSS	Active TSS
17	37846521	*PGAP3; ERBB2*	rs2517960	Weak transcription	Quiescent/Low	Weak transcription	Weak transcription	Weak transcription
17	45146717	*RP11-156P1.2*	rs34710835	Quiescent/Low	Quiescent/Low	Weak transcription	Weak transcription	Weak transcription
17	47090785	*IGF2BP1*	rs11079849	Flanking Active TSS	Weak transcription	Repressed PolyComb	Weak Repressed PolyComb	Repressed PolyComb
17	53400110	*HLF*	rs12940636	Quiescent/Low	Quiescent/Low	Weak transcription	Weak transcription	Quiescent/Low

17	56429673	*SUPT4H1*	rs2680703	Active TSS	Active TSS	Active TSS	Active TSS	Active TSS
17	64318357	*PRKCA*	rs9912468	Quiescent/Low	Quiescent/Low	Weak transcription	Quiescent/Low	Heterochromatin
17	65893289	*BPTF*	rs145606454	Weak transcription	Quiescent/Low	Strong transcription	Weak transcription	Weak transcription
17	76798155	*USP36*	rs2306527	Weak transcription	Weak transcription	Strong transcription	Strong transcription	Strong transcription
18	1837007	-	rs1371274	Quiescent/Low	Quiescent/Low	Quiescent/Low	Quiescent/Low	Quiescent/Low
18	21138865	*NPC1*	rs2472610	Weak transcription	Quiescent/Low	Weak transcription	Weak transcription	Weak transcription
18	31259579	*ASXL3*	rs12607512	Weak transcription	Quiescent/Low	Weak transcription	Quiescent/Low	Weak transcription
18	36553476	-	rs60951582	Quiescent/Low	Quiescent/Low	Heterochromatin	Quiescent/Low	Heterochromatin
18	57739072	-	rs11664369	Quiescent/Low	Quiescent/Low	Quiescent/Low	Quiescent/Low	Quiescent/Low
19	11188247	*LDLR*	rs56289821	Quiescent/Low	Quiescent/Low	Quiescent/Low	Quiescent/Low	Quiescent/Low
19	18826052	*CRTC1*	rs4808152	Weak transcription	Weak transcription	Weak transcription	Weak transcription	Weak transcription
19	46327831	*SYMPK*	rs10520390	Weak transcription	Weak transcription	Strong transcription	Strong transcription	Strong transcription
21	30540165	*MAP3K7CL*	rs56968346	Quiescent/Low	Weak transcription	Quiescent/Low	Quiescent/Low	Quiescent/Low
21	42626851	*BACE2*	rs12482462	Weak transcription	Quiescent/Low	Strong transcription	Strong transcription	ZNF genes & repeats
22	24178279	*DERL3*	rs5760061	Enhancers	Enhancers	Enhancers	Enhancers	Quiescent/Low

7. Subtype Polygenic Risk Score (PRS) Construction and Comparison

Could PRSs derived from GWASs of all-cause HF, HFrEF, and HFpEF be validated for subtype discrimination in external datasets (e.g., UK Biobank), or used to predict related cardiovascular conditions (e.g., ischemic heart disease, atrial fibrillation, or valvular disease)? Conversely, could PRSs for these conditions stratify HF subtypes? Additionally, could PRS-PheWAS be used to systematically explore traits linked to HF?

UK Biobank currently lacks the routine echocardiographic information required to reliably sub-phenotype prevalent HF cases as HFrEF vs HFpEF. Therefore, validation of subtype discrimination within UKB is not feasible. Instead, we constructed genetic risk scores (GRS) for all-cause HF and HFrEF from our discovery GWAS and evaluated their phenome-wide associations across the UKB EHR phenome. HFpEF GRS was not pursued due to limited number of significant loci. To construct a PRS distinguishing HF subtypes, we would need to identify differential genetic associations between HF subtypes in a separate GWAS. Given the scope of the present manuscript, we decided to address this unique PRS utility in a separate study.

Using the 136 sentinel loci for all-cause HF and 46 sentinel loci for HFrEF, we created weighted genetic risk scores using GWAS-based β coefficients for each participant. Among 361,048 unrelated UKB participants with genetic data and linked hospital inpatient records, we mapped ICD-10 diagnoses to phecodes and tested 1,238 phecodes with ⩾20 cases. We performed a PheWAS using the PheWAS R package (https://github.com/PheWAS/PheWAS), fitting logistic regression models for each phecode with adjustment for sex and the top ten genetic principal components. Multiple testing was controlled using Bonferroni correction.

Both the all-cause HF GRS and the HFrEF GRS showed strongest associations with cardiovascular phenotypes, notably atrial fibrillation/flutter and cardiac dysrhythmias, as well as hypertension. Results are summarized below.

Response Table 2A. Phenome-wide Association Study of the All-cause HF Genetic Risk Score. All Associations with Bonferroni corrected P < 0.05 are listed.

Phenotype	OR	P	Bonferroni	N Cases	N Controls
Hypertension	1.08 (1.07, 1.09)	2.89E-110	6.23E-108	129956	231092
Essential hypertension	1.08 (1.07, 1.09)	2.89E-110	6.23E-108	129956	231092
Cardiac dysrhythmias	1.13 (1.11, 1.15)	2.63E-54	5.68E-52	17402	343646
Atrial fibrillation and flutter	1.13 (1.11, 1.15)	2.63E-54	5.68E-52	17402	343646
Pleurisy; pleural effusion	1.06 (1.04, 1.07)	8.10E-11	1.75E-08	15063	344571
Chronic ulcer of skin	1.09 (1.06, 1.13)	2.44E-07	5.28E-05	3470	357578
Other diseases of respiratory system, not elsewhere classified	1.04 (1.03, 1.06)	8.29E-07	0.0002	15205	345843
Other diseases of respiratory system, NEC	1.04 (1.03, 1.06)	8.29E-07	0.0002	15205	345843
Other symptoms/disorders or the urinary system	1.04 (1.02, 1.06)	1.89E-05	0.0041	11889	333684
Cerebrovascular disease	1.11 (1.06, 1.17)	2.68E-05	0.0058	1569	359479
Occlusion of cerebral arteries	1.11 (1.06, 1.17)	2.68E-05	0.0058	1569	359479
Cerebral artery occlusion, with cerebral infarction	1.11 (1.06, 1.17)	2.68E-05	0.0058	1569	359479
Urinary incontinence	1.05 (1.02, 1.08)	9.65E-05	0.0208	6611	333684

Gangrene	1.1 (1.04, 1.15)	0.0002	0.0468	1618	359430

Response Table 2B. Phenome-wide Association Study of the HFrEF Genetic Risk Score. All Associations with Bonferroni Corrected P < 0.05 Are Shown.

Phenotype	OR	P	Bonferroni	N Cases	N Controls
Cardiac dysrhythmias	1.09 (1.08, 1.11)	2.00E-30	4.33E-28	17402	343646
Atrial fibrillation and flutter	1.09 (1.08, 1.11)	2.00E-30	4.33E-28	17402	343646
Hypertension	1.03 (1.02, 1.03)	2.09E-13	4.51E-11	129956	231092
Essential hypertension	1.03 (1.02, 1.03)	2.09E-13	4.51E-11	129956	231092

8. Drug Target Prioritization

Given the large-scale elucidation of the genetic architecture of heart failure, it would be valuable to examine the relationship between currently approved HF medications and GWAS findings, as well as explore potential drug repurposing opportunities based on newly identified loci. Specifically, could GWAS loci be mapped to known or putative drug targets (e.g., using DrugBank), and their clinical relevance discussed?

We appreciate this insightful suggestion. In response, we systematically mapped our genome-wide significant loci to known or putative drug targets using DrugBank 6.0 (PubMed: 37953279). For each locus, we annotated the nearest protein-coding gene and queried DrugBank for drugs targeting the corresponding protein. We then examined whether any of these agents corresponded to currently approved guideline-directed HF therapies (e.g., renin-angiotensin system inhibitors, β-blockers, mineralocorticoid receptor antagonists, SGLT2 inhibitors, ARNI, hydralazine-nitrate, ivabradine, soluble guanylate cyclase stimulators). Although none of the mapped drugs in our GWAS-based analysis are approved specifically for HF treatment, these new targets may offer hypothesis-generating opportunities for repurposing non-HF drugs and for exploring novel therapeutic pathways. We added these results to the manuscript:

Methods:

Therapeutic target profile
"We additionally queried DrugBank 6.0 (PubMed: 37953279) using gene symbols and corresponding UniProt identifiers. For each gene, we extracted all drugs with a documented interaction with the encoded protein."

Results:

"Overall, the queries from the DrugBank indicate that the genetic architecture of HF identified in our GWAS is not simply a reflection of existing pharmacologic pathways targeted by current HF guideline directed medical therapy. Instead, the gene-drug links we identified are primarily hypothesis-generating signals that highlight potential avenues for drug repurposing and motivate further experimental work, Table S23B."

Table S23B. Mapping of Genome-wide Significant Heart Failure Loci to DrugBank-annotated Pharmacologic Targets.

rsID	Chr.	Position (hg19)	Gene	DrugBank ID	UniProt ID	Drug Name	Drug Group	Action
Genome-wide Significant Loci for HFrEF								
rs945425	1	16348412	*CLCNKA*	BE0002376	P51800	Niflumic acid	experimental	inducer
						Talniflumate	investigational	antagonist
rs7188250	16	53834607	*FTO*	BE0005795	Q9C0B1	Artenimol	approved, investigational	ligand
Genome-wide Significant Loci for HFpEF								
rs62048402	16	53803223	*FTO*	BE0005795	Q9C0B1	Artenimol	approved, investigational	ligand
Genome-wide Significant Loci for All-cause HF								
rs2009371	1	16348083	*CLCNKA*	BE0002376	P51800	Niflumic acid	experimental	inducer
						Talniflumate	investigational	antagonist
rs62048402	16	53803223	*FTO*	BE0005795	Q9C0B1	Artenimol	approved, investigational	ligand

9. Internal and External Replication

For novel loci, replication would significantly strengthen the findings. I recommend demonstrating consistency via internal replication within the study cohort, and validating robustness through external replication in independent datasets.

We thank the reviewer for this important suggestion. As part of the original analysis, we performed ancestry-specific and study-specific GWAS and provided full summary statistics for each locus across contributing cohorts and ancestry groups. These results demonstrate consistent effect directions and broadly similar effect sizes across ancestries and studies, thereby serving as internal validation of the multi-ancestry results from the MVP and BioVU cohorts. In addition, we have performed external replication using an independent Japanese GWAS of HF and subtypes from BioBank Japan (BBJ), which includes 16,251 all-cause HF cases and 197,577 controls (N = 213,828), 4,254 HFrEF cases and 197,577 controls (N = 201,831), and 7,154 HFpEF cases and 197,577 controls (N = 204,731), as reported by Enzan et al. (PMID: 41184235). For each lead variant from our multi-ancestry meta-analyses, we extracted the corresponding association statistics in BBJ for the matched HF phenotype (all-cause HF, HFrEF, or HFpEF). We have added a new subsection describing these analyses in the Methods and Results and the updated corresponding tables in the Results (below).

Methods:
External validation in the Biobank Japan

"External validation was performed using the independent Japanese GWAS of HF from BioBank Japan, which includes 16,251 all-cause HF cases and 197,577 controls (N = 213,828), 4,254 HFrEF cases and 197,577 controls (N = 201,831), and 7,154 HFpEF cases and 197,577 controls (N = 204,731), as reported by Enzan et al. (PMID: 41184235).
Results
"Out of the 46 GWS loci identified in the multi-ancestry meta-analysis of HFrEF, 34 loci were available in Biobank Japan; among these, 21 loci replicated at $P < 0.05$ with the same direction of genetic effect. For

HFpEF, 1 of the 3 GWS loci was available in Biobank Japan and replicated at P < 0.05 with concordant effect direction. For all-cause HF, out of the 136 GWS loci, 106 loci were available in Biobank Japan, of which 46 replicated at P < 0.05 with concordant effect direction, Tables 1 and S2-S3. Collectively, the ancestry- and study-specific internal results, together with the substantial proportion of loci that replicate in an independent East Asian population with consistent effect directions, support the robustness and generalizability of our findings."

Revised Table 1. Genome-wide Significant Loci for the Multi-ancestry Meta-analysis of GWAS on HFrEF and HFpEF.

rsID	Chr.	Position (hg19)	Gene	Novel *	EA	NEA	Meta-analysis		Biobank Japan Validation		
							OR (95% CI)	P	EAF	OR (95% CI)	P
The 46 Genome-wide Significant Loci for HFrEF											
rs945425	1	16348412	*CLCNKA*	No	C	T	1.11 (1.1, 1.13)	1.37E-37	-	-	-
rs1997998	1	61883027	*NFIA*	No	T	G	1.06 (1.05, 1.08)	3.12E-13	0.03	1.06 (0.91, 1.23)	4.71E-01
rs12740374	1	109817590	*CELSR2*	No	G	T	1.06 (1.04, 1.08)	6.41E-10	-	-	-
rs10429888	1	162024987	*NOS1AP*	Yes	G	A	1.08 (1.05, 1.11)	4.57E-09	0.62	1.05 (1, 1.1)	4.74E-02
rs524517	1	236839990	*ACTN2*	No	T	A	1.07 (1.05, 1.1)	1.83E-08	0.81	1.1 (1.04, 1.17)	8.55E-04
rs34514099	2	11571966	*E2F6*	No	T	C	1.06 (1.04, 1.08)	2.74E-11	0.59	1.11 (1.06, 1.16)	1.70E-05
rs62133082	2	37233937	*HEATR5B*	No	T	C	1.06 (1.04, 1.09)	1.30E-09	0.43	1.08 (1.03, 1.13)	1.56E-03
rs17076	2	179595117	*TTN*	Yes	C	G	1.08 (1.06, 1.1)	4.94E-14	0.34	1.17 (1.12, 1.23)	3.41E-11
rs4642101	3	12842223	*CAND2*	No	G	T	1.05 (1.03, 1.07)	4.18E-09	0.3	1.15 (1.1, 1.21)	5.41E-09
rs6807275	3	14274451	*3p25.1*	No	G	A	1.07 (1.05, 1.09)	4.72E-13	0.3	1.14 (1.09, 1.2)	5.98E-08
rs34989545	3	49622632	*BSN*	No	G	GT	1.06 (1.04, 1.08)	1.33E-09	-	-	-
rs2082838	3	158305894	*MLF1*	No	A	G	1.06 (1.04, 1.08)	4.00E-09	0.06	1.01 (0.92, 1.11)	8.30E-01
rs17042098	4	111664158	*4q25*	No	A	G	1.1 (1.07, 1.12)	4.34E-15	0.31	1.09 (1.04, 1.14)	7.96E-04
rs11242464	5	138746334	*DNAJC18*	No	C	G	1.05 (1.03, 1.07)	2.32E-08	0.41	1.13 (1.08, 1.18)	3.27E-07
rs9349379	6	12903957	*PHACTR1*	No	G	A	1.06 (1.05, 1.08)	4.63E-14	0.66	1.01 (0.97, 1.07)	5.68E-01
rs146170154	6	36646768	*CDKN1A*	No	C	CTA	1.14 (1.12, 1.16)	5.14E-41	0.89	1.24 (1.15, 1.35)	4.05E-08
rs1407229	6	53971921	*MLIP*	No	T	C	1.06 (1.04, 1.07)	2.97E-11	0.34	1.08 (1.03, 1.13)	2.54E-03
rs66577603	6	79749166	*PHIP*	No	C	CTT	1.04 (1.03, 1.06)	4.77E-08	0.63	1.03 (0.98, 1.08)	2.60E-01
rs282064	6	91303512	*MAP3K7*	Yes	T	G	1.05 (1.04, 1.07)	2.44E-10	0.33	1.06 (1.01, 1.11)	1.34E-02
rs10455872	6	161010118	*LPA*	No	G	A	1.15 (1.12, 1.19)	2.29E-18	-	-	-
rs3211938	7	80300449	*CD36*	Yes	G	T	1.2 (1.14, 1.28)	3.92E-10	-	-	-
rs2270590	7	128455916	*CCDC136*	No	G	A	1.08 (1.06, 1.1)	5.28E-15	0.44	1.06 (1.02, 1.11)	8.85E-03
rs7461129	8	125861374	*8q24.13*	No	C	T	1.07 (1.05, 1.09)	2.03E-14	0.5	1.14 (1.09, 1.2)	7.86E-09
rs4977575	9	22124744	*RP11-145E5.5*	No	G	C	1.08 (1.07, 1.1)	5.37E-22	0.66	1.07 (1.02, 1.12)	6.56E-03
rs10814273	9	35723234	*TLN1*	Yes	T	G	1.05 (1.03, 1.07)	3.12E-08	0.05	1.12 (1.01, 1.23)	2.95E-02
rs600038	9	136151806	*9q34.2*	No	C	T	1.06 (1.04, 1.08)	2.06E-09	0.28	1.05 (1, 1.11)	4.44E-02
rs485838	10	44750414	*10q11.21*	Yes	G	T	1.06 (1.04, 1.08)	1.94E-08	0.67	1.02 (0.97, 1.07)	3.59E-01
rs148307915	10	49528021	*MAPK8*	Yes	A	G	1.08 (1.05, 1.1)	2.29E-09	-	-	-
rs2242254	10	75585307	*CAMK2G*	No	G	A	1.07 (1.05, 1.1)	3.48E-10	0.79	1.04 (0.98, 1.1)	2.01E-01
rs375034445	10	121424815	*BAG3*	No	A	AT	1.14 (1.12, 1.16)	5.09E-34	-	-	-
rs113123267	11	47379410	*SPI1*	No	T	C	1.35 (1.26, 1.46)	2.74E-15	-	-	-
rs111786504	11	55047396	*TRIM48*	No	G	A	1.61 (1.39, 1.86)	1.57E-10	-	-	-
rs60504091	11	56329980	*OR5M11*	No	A	G	1.26 (1.18, 1.36)	3.56E-10	-	-	-
rs658286	11	100942792	*PGR*	No	T	G	1.05 (1.03, 1.07)	1.01E-08	-	-	-
rs11169562	12	51187290	*ATF1*	No	T	C	1.05 (1.03, 1.07)	1.80E-09	0.36	1.02 (0.98, 1.07)	3.11E-01
rs4575361	12	124410529	*DNAH10; CCDC92*	No	A	T	1.05 (1.03, 1.07)	9.36E-09	0.84	1.03 (0.97, 1.1)	3.35E-01
rs62006566	15	75086534	*CSK*	Yes	C	T	1.05 (1.03, 1.07)	2.69E-08	-	-	-
rs7188250	16	53834607	*FTO*	No	C	T	1.06 (1.05, 1.08)	1.55E-13	0.21	1.05 (0.99, 1.11)	1.10E-01
rs2224770	17	2205923	*SMG6*	No	T	C	1.05 (1.03, 1.07)	1.76E-09	0.24	1.08 (1.03, 1.14)	2.92E-03
rs9907657	17	7460957	*TNFSF12; TNFSF12-TNFSF13*	No	T	G	1.06 (1.04, 1.09)	6.09E-09	0.21	1 (0.95, 1.06)	9.36E-01
rs2517953	17	37841211	*PGAP3*	No	G	C	1.06 (1.05, 1.08)	5.84E-14	0.5	1.02 (0.98, 1.07)	3.49E-01
rs9912468	17	64318357	*PRKCA*	No	G	C	1.05 (1.03, 1.06)	2.49E-09	0.41	1.06 (1.01, 1.11)	1.03E-02
rs145606454	17	65893289	*BPTF*	No	A	AC	1.06 (1.04, 1.08)	3.97E-09	0.68	1.02 (0.97, 1.07)	5.58E-01
rs7229491	18	46516424	*18q21.1*	Yes	C	G	1.05 (1.03, 1.07)	4.82E-09	0.46	1.01 (0.97, 1.06)	6.38E-01
rs56968346	21	30540165	*MAP3K7CL*	No	T	C	1.09 (1.07, 1.11)	4.17E-17	0.1	1.16 (1.08, 1.25)	5.28E-05
rs9608201	22	24166788	*SMARCB1*	No	A	G	1.07 (1.05, 1.09)	3.24E-14	0.61	1.17 (1.11, 1.22)	1.19E-10
**The 3 Genome-wide Significant Loci for HFpEF**											
rs62048402	16	53803223	*FTO*	No	A	G	1.06 (1.05, 1.08)	1.10E-13	0.2	1.06 (1.02, 1.11)	6.34E-03
rs75716431	17	66382647	*ARSG*	No	T	C	1.12 (1.08, 1.17)	4.27E-08	0.12	1.02 (0.96, 1.07)	5.39E-01
rs113752382	21	18617840	*21q21.1*	Yes	T	C	1.12 (1.08, 1.17)	3.39E-08	-	-	-

Chr.: Chromosome; EA: Effect Allele; NEA: Non-effect Allele; EAF: Effect Allele Frequency; OR: Odds Ratio; CI: Confidence Interval.

*A total of 9 novel HFrEF loci and 1 novel HFpEF locus as compared to previous all-cause HF GWAS (PMID: 31919418, 36376295, 36517512, 37429843, 37503172, 40038546), defined as non-overlapping physical distance of > 500kb on both sides of the variant.

Revised Table S2. Genome-wide Significant Loci for the Multi-ancestry Meta-analysis of GWAS on HFrEF and HFpEF, Including Population-specific Results.

rsID	Chr.	Position (hg19)	Gene	Novel *	EA	NEA	Meta-analysis		MVP European			BIOVU European			MVP African			BIOVU African			MVP Hispanic			MVP Asian			Mulit-ancestry Heterogeneity P**	Biobank Japan Validation		
							OR (95% CI)	P	EAF	OR (95% CI)	P	EAF	OR (95% CI)	P	EAF	OR (95% CI)	P	EAF	OR (95% CI)	P	EAF	OR (95% CI)	P	EAF	OR (95% CI)	P		EAF	OR (95% CI)	P
**The 46 Genome-wide Significant Loci for HFrEF**																														
rs945425	1	16348412	*CLCNKA*	No	C	T	1.11 (1.1, 1.13)	1.37E-37	0.67	1.11 (1.08, 1.13)	1.82E-24	0.68	1.17 (1.07, 1.28)	8.97E-04	0.74	1.12 (1.08, 1.17)	1.14E-08	0.74	1.57 (1.23, 2.01)	2.47E-04	0.63	1.14 (1.06, 1.23)	3.29E-04	0.93	1.15 (0.74, 1.79)	5.33E-01	0.0705	-	-	-
rs1997998	1	61883027	*NFIA*	No	T	G	1.06 (1.05, 1.08)	3.12E-13	0.36	1.07 (1.09, 1.05)	7.13E-12	0.35	1.1 (1.2, 1.01)	2.79E-02	0.12	1.06 (1.12, 1)	6.82E-02	0.11	0.92 (1.27, 0.67)	6.11E-01	0.24	1.02 (1.11, 0.94)	6.11E-01	0.06	1.01 (1.73, 0.59)	9.72E-01	0.7830	0.03	1.06 (0.91, 1.23)	4.71E-01
rs12740374	1	109817590	*CELSR2*	No	G	T	1.06 (1.04, 1.08)	6.41E-10	0.78	1.06 (1.09, 1.04)	9.76E-09	0.78	1.15 (1.27, 1.04)	8.31E-03	0.74	1.03 (1.07, 0.99)	1.13E-01	0.76	0.88 (1.09, 0.7)	2.42E-01	0.79	1.04 (1.13, 0.96)	3.41E-01	0.93	1.37 (2.03, 0.93)	1.13E-01	0.1128	-	-	-
rs10429888	1	162024987	*NOS1AP*	Yes	G	A	1.08 (1.05, 1.11)	4.57E-09	0.86	1.07 (1.1, 1.04)	5.11E-07	-	-	-	0.97	1.08 (1.21, 0.97)	1.58E-01	-	-	-	0.86	1.19 (1.33, 1.07)	1.28E-03	0.68	1.08 (1.37, 0.86)	4.93E-01	0.3034	0.62	1.05 (1, 1.1)	4.74E-02
rs524517	1	236839990	*ACTN2*	No	T	A	1.07 (1.05, 1.1)	1.83E-08	0.89	1.05 (1.09, 1.02)	3.67E-04	-	-	-	0.81	1.1 (1.16, 1.05)	4.74E-05	-	-	-	0.82	1.14 (1.25, 1.04)	5.45E-03	0.84	0.89 (1.18, 0.67)	4.26E-01	0.1177	0.81	1.1 (1.04, 1.17)	8.55E-04
rs34514099	2	11571966	*E2F6*	No	T	C	1.06 (1.04, 1.08)	2.74E-11	0.34	1.06 (1.04, 1.08)	1.18E-10	-	-	-	0.1	1.05 (0.99, 1.12)	1.22E-01	-	-	-	0.47	1.03 (0.96, 1.11)	3.46E-01	0.59	1.07 (0.85, 1.33)	5.72E-01	0.8795	0.59	1.11 (1.06, 1.16)	1.70E-05
rs62133082	2	37233937	*HEATR5B*	No	T	C	1.06 (1.04, 1.09)	1.30E-09	0.81	1.07 (1.09, 1.04)	8.81E-09	0.81	1.05 (1.16, 0.94)	4.02E-01	0.92	1.05 (1.12, 0.98)	1.47E-01	0.92	1.4 (2.11, 0.93)	1.11E-01	0.75	1.05 (1.14, 0.97)	2.10E-01	0.55	0.95 (1.16, 0.77)	5.90E-01	0.6349	0.43	1.08 (1.03, 1.13)	1.56E-03
rs17076	2	179595117	*TTN*	Yes	C	G	1.08 (1.06, 1.1)	4.94E-14	0.82	1.07 (1.1, 1.05)	1.03E-08	0.81	1.15 (1.28, 1.03)	1.37E-02	0.74	1.08 (1.12, 1.04)	2.24E-04	-	-	-	0.67	1.1 (1.18, 1.02)	1.06E-02	0.45	1.02 (1.25, 0.83)	8.63E-01	0.7007	0.34	1.17 (1.12, 1.23)	3.41E-11
rs4642101	3	12842223	*CAND2*	No	G	T	1.05 (1.03, 1.07)	4.18E-09	0.64	1.05 (1.03, 1.07)	1.75E-06	0.64	1.06 (0.97, 1.16)	1.72E-01	0.71	1.05 (1.01, 1.09)	2.25E-02	0.72	1.09 (0.88, 1.36)	4.24E-01	0.47	1.08 (1.01, 1.16)	3.49E-02	0.3	1.09 (0.88, 1.37)	4.28E-01	0.9587	0.3	1.15 (1.1, 1.21)	5.41E-09
rs6807275	3	14274451	*3p25.1*	No	G	A	1.07 (1.05, 1.09)	4.72E-13	0.68	1.07 (1.09, 1.05)	2.16E-10	0.66	1.1 (1.2, 1)	4.09E-02	0.87	1.11 (1.17, 1.05)	3.42E-04	0.86	1.29 (1.75, 0.95)	9.80E-02	0.79	0.99 (1.08, 0.91)	8.44E-01	0.35	0.86 (1.1, 0.68)	2.25E-01	0.1024	0.3	1.14 (1.09, 1.2)	5.98E-08
rs34989545	3	49622632	*BSN*	No	G	GT	1.06 (1.04, 1.08)	1.33E-09	0.18	1.05 (1.08, 1.03)	1.14E-05	-	-	-	0.28	1.09 (1.13, 1.05)	2.57E-05	-	-	-	0.14	1.07 (1.2, 0.96)	1.92E-01	0.05	0.95 (1.94, 0.47)	8.89E-01	0.5999	-	-	-
rs2082838	3	158305894	*MLF1*	No	A	G	1.06 (1.04, 1.08)	4.00E-09	0.15	1.05 (1.02, 1.07)	4.04E-04	0.15	1.08 (0.96, 1.21)	1.95E-01	0.44	1.08 (1.04, 1.12)	2.79E-05	0.44	1.31 (1.08, 1.6)	7.09E-03	0.18	1.08 (0.99, 1.18)	9.30E-02	0.08	0.9 (0.63, 1.3)	5.76E-01	0.1886	0.06	1.01 (0.92, 1.11)	8.30E-01
rs17042098	4	111664158	*4q25*	No	A	G	1.1 (1.07, 1.12)	4.34E-15	0.12	1.09 (1.06, 1.12)	8.26E-10	0.11	1.19 (1.06, 1.35)	4.83E-03	0.14	1.07 (1.02, 1.13)	6.64E-03	0.14	0.99 (0.75, 1.3)	9.34E-01	0.19	1.16 (1.06, 1.27)	1.01E-03	0.33	1.26 (1.01, 1.57)	4.43E-02	0.2844	0.31	1.09 (1.04, 1.14)	7.96E-04
rs11242464	5	138746334	*DNAJC18*	No	C	G	1.05 (1.03, 1.07)	2.32E-08	0.68	1.04 (1.02, 1.06)	9.34E-05	0.68	1.13 (1.04, 1.24)	6.45E-03	0.29	1.06 (1.02, 1.11)	3.67E-03	-	-	-	0.62	1.1 (1.02, 1.18)	1.22E-02	0.33	0.93 (0.74, 1.17)	5.42E-01	0.1636	0.41	1.13 (1.08, 1.18)	3.27E-07
rs9349379	6	12903957	*PHACTR1*	No	G	A	1.06 (1.05, 1.08)	4.63E-14	0.4	1.06 (1.04, 1.08)	1.37E-11	0.41	1.13 (1.04, 1.22)	5.51E-03	0.08	1 (0.94, 1.07)	9.76E-01	0.09	1.61 (1.18, 2.2)	2.80E-03	0.35	1.1 (1.02, 1.18)	1.27E-02	0.59	1.11 (0.91, 1.36)	3.06E-01	0.0269	0.66	1.01 (0.97, 1.07)	5.68E-01
rs146170154	6	36646768	*CDKN1A*	No	C	CTA	1.14 (1.12, 1.16)	5.14E-41	0.79	1.14 (1.16, 1.11)	7.84E-30	0.8	1.22 (1.36, 1.1)	2.82E-04	0.8	1.14 (1.2, 1.09)	5.16E-09	-	-	-	0.83	1.12 (1.22, 1.02)	1.57E-02	0.79	1.07 (1.39, 0.83)	6.02E-01	0.7246	0.89	1.24 (1.15, 1.35)	4.05E-08
rs1407229	6	53971921	*MLIP*	No	T	C	1.06 (1.04, 1.07)	2.97E-11	0.36	1.06 (1.09, 1.05)	5.29E-11	-	-	-	0.69	1.02 (1.06, 0.98)	4.12E-01	0.68	1.06 (1.3, 0.86)	6.09E-01	0.48	1.09 (1.17, 1.01)	2.01E-02	0.31	1.01 (1.26, 0.8)	9.46E-01	0.2730	0.34	1.08 (1.03, 1.13)	2.54E-03
rs66577603	6	79749166	*PHIP*	No	C	CTT	1.04 (1.03, 1.06)	4.77E-08	0.52	1.05 (1.03, 1.06)	1.84E-06	0.54	1.05 (0.97, 1.14)	2.58E-01	0.43	1.04 (1, 1.08)	5.20E-02	0.44	1.12 (0.92, 1.35)	2.73E-01	0.48	1.04 (0.96, 1.11)	3.40E-01	0.67	1.12 (0.89, 1.41)	3.19E-01	0.9574	0.63	1.03 (0.98, 1.08)	2.60E-01
rs282064	6	91303512	*MAP3K7*	Yes	T	G	1.05 (1.04, 1.07)	2.44E-10	0.31	1.05 (1.03, 1.07)	2.06E-06	0.32	1.13 (1.04, 1.23)	5.26E-03	0.4	1.05 (1.02, 1.09)	5.36E-03	0.41	1.2 (0.98, 1.46)	7.48E-02	0.31	1.07 (0.99, 1.15)	1.01E-01	0.36	1.12 (0.89, 1.41)	3.25E-01	0.4426	0.33	1.06 (1.01, 1.11)	1.34E-02
rs10455872	6	161010118	*LPA*	No	G	A	1.15 (1.12, 1.19)	2.29E-18	0.07	1.15 (1.11, 1.19)	3.65E-16	0.07	1.29 (1.12, 1.5)	5.95E-04	0.01	0.95 (0.81, 1.11)	4.82E-01	0.01	2.08 (1.02, 4.24)	4.36E-02	0.04	1.24 (1.04, 1.47)	1.82E-02	-	-	-	0.0187	-	-	-
rs3211938	7	80300449	*CD36*	Yes	G	T	1.2 (1.14, 1.28)	3.92E-10	-	-	-	-	-	-	0.09	1.19 (1.12, 1.26)	1.48E-08	0.09	1.7 (1.27, 2.27)	3.60E-04	-	-	-	-	-	-	0.0181	-	-	-
rs2270590	7	128455916	*CCDC136*	No	G	A	1.08 (1.06, 1.1)	5.28E-15	0.84	1.07 (1.09, 1.04)	2.29E-07	0.85	1.17 (1.31, 1.04)	1.15E-02	0.39	1.1 (1.14, 1.06)	9.90E-08	0.39	1.03 (1.25, 0.84)	8.03E-01	0.74	1.11 (1.21, 1.03)	8.04E-03	0.58	0.84 (1.04, 0.68)	1.04E-01	0.0744	0.44	1.06 (1.02, 1.11)	8.85E-03
rs7461129	8	125861374	*8q24.13*	No	C	T	1.07 (1.05, 1.09)	2.03E-14	0.69	1.06 (1.08, 1.04)	4.00E-09	0.68	1.11 (1.22, 1.02)	1.89E-02	0.88	1.11 (1.17, 1.05)	3.96E-04	0.87	1.35 (1.86, 0.98)	6.77E-02	0.72	1.09 (1.18, 1.01)	2.44E-02	0.59	1.32 (1.63, 1.06)	1.14E-02	0.1131	0.5	1.14 (1.09, 1.2)	7.86E-09
rs4977575	9	22124744	*RP11-145E5.5*	No	G	C	1.08 (1.07, 1.1)	5.37E-22	0.5	1.09 (1.07, 1.11)	1.13E-21	0.5	1.01 (0.93, 1.09)	9.03E-01	0.88	1.01 (0.95, 1.08)	7.44E-01	0.89	0.91 (0.66, 1.26)	5.73E-01	0.57	1.13 (1.05, 1.22)	6.15E-04	0.65	1.09 (0.88, 1.35)	4.39E-01	0.0481	0.66	1.07 (1.02, 1.12)	6.56E-03
rs10814273	9	35723234	*TLN1*	Yes	T	G	1.05 (1.03, 1.07)	3.12E-08	0.28	1.05 (1.03, 1.07)	3.05E-07	0.29	1.07 (0.98, 1.17)	1.28E-01	0.22	1.04 (1, 1.09)	6.26E-02	-	-	-	0.31	1 (0.93, 1.08)	9.61E-01	0.13	1.26 (0.93, 1.7)	1.37E-01	0.4719	0.05	1.12 (1.01, 1.23)	2.95E-02
rs600038	9	136151806	*9q34.2*	No	C	T	1.06 (1.04, 1.08)	2.06E-09	0.22	1.05 (1.03, 1.08)	9.20E-07	0.21	1.02 (0.92, 1.13)	7.18E-01	0.14	1.06 (1.01, 1.12)	1.73E-02	0.13	1.32 (1.01, 1.72)	4.36E-02	0.17	1.14 (1.04, 1.24)	4.26E-03	0.18	0.99 (0.76, 1.27)	9.21E-01	0.2899	0.28	1.05 (1, 1.11)	4.44E-02
rs485838	10	44750414	*10q11.21*	Yes	G	T	1.06 (1.04, 1.08)	1.94E-08	0.86	1.06 (1.09, 1.04)	5.36E-06	0.86	1.18 (1.34, 1.04)	9.01E-03	0.64	1.04 (1.08, 1)	5.43E-02	0.64	1.18 (1.44, 0.96)	1.20E-01	0.78	1.08 (1.17, 0.99)	8.30E-02	0.76	1.1 (1.4, 0.86)	4.66E-01	0.3502	0.67	1.02 (0.97, 1.07)	3.59E-01
rs148307915	10	49528021	*MAPK8*	Yes	A	G	1.08 (1.05, 1.1)	2.29E-09	0.13	1.07 (1.04, 1.1)	7.68E-07	0.13	1 (0.88, 1.13)	9.87E-01	0.07	1.09 (1.01, 1.17)	1.86E-02	-	-	-	0.12	1.2 (1.08, 1.33)	7.56E-04	0.02	1.39 (0.58, 3.33)	4.59E-01	0.1914	-	-	-
rs2242254	10	75585307	*CAMK2G*	No	G	A	1.07 (1.05, 1.1)	3.48E-10	0.86	1.08 (1.11, 1.05)	1.15E-09	-	-	-	0.89	1.06 (1.12, 1)	3.99E-02	-	-	-	0.89	0.98 (1.1, 0.88)	7.87E-01	0.81	1.17 (1.51, 0.9)	2.31E-01	0.3416	0.79	1.04 (0.98, 1.1)	2.01E-01
rs375034445	10	121424815	*BAG3*	No	A	AT	1.14 (1.12, 1.16)	5.09E-34	0.79	1.13 (1.15, 1.1)	2.18E-26	0.8	1.4 (1.57, 1.25)	4.93E-09	0.96	1.2 (1.32, 1.09)	1.77E-04	-	-	-	0.89	1.07 (1.2, 0.96)	2.06E-01	0.97	1.64 (3.2, 0.84)	1.43E-01	0.0020	-	-	-
rs113123267	11	47379410	*SPI1*	No	T	C	1.35 (1.26, 1.46)	2.74E-15	-	-	-	-	-	-	0.05	1.33 (1.23, 1.44)	2.12E-13	0.05	2 (1.37, 2.92)	3.22E-04	-	-	-	-	-	-	0.0383	-	-	-
rs111786504	11	55047396	*TRIM48*	No	G	A	1.61 (1.39, 1.86)	1.57E-10	-	-	-	-	-	-	0.03	1.55 (1.33, 1.82)	3.04E-08	0.04	1.98 (1.34, 2.94)	6.65E-04	-	-	-	-	-	-	0.2602	-	-	-
rs60504091	11	56329980	*OR5M11*	No	A	G	1.26 (1.18, 1.36)	3.56E-10	-	-	-	-	-	-	0.05	1.25 (1.35, 1.16)	2.93E-09	0.04	1.65 (2.47, 1.1)	1.54E-02	-	-	-	-	-	-	0.1919	-	-	-
rs658286	11	100942792	*PGR*	No	T	G	1.05 (1.03, 1.07)	1.01E-08	0.5	1.05 (1.07, 1.03)	4.62E-07	-	-	-	0.79	1.04 (1.09, 0.99)	8.31E-02	0.78	1 (1.26, 0.79)	9.73E-01	0.61	1.1 (1.19, 1.03)	7.34E-03	0.92	0.84 (1.25, 0.57)	3.97E-01	0.4842	-	-	-
rs11169562	12	51187290	*ATF1*	No	T	C	1.05 (1.03, 1.07)	1.80E-09	0.39	1.05 (1.03, 1.07)	1.02E-07	0.4	1.05 (0.96, 1.14)	2.72E-01	0.33	1.03 (0.99, 1.07)	1.17E-01	0.33	1.15 (0.94, 1.41)	1.74E-01	0.29	1.1 (1.02, 1.18)	1.63E-02	0.24	0.98 (0.77, 1.25)	8.75E-01	0.6363	0.36	1.02 (0.98, 1.07)	3.11E-01
rs4575361	12	124410529	*DNAH10; CCDC92*	No	A	T	1.05 (1.03, 1.07)	9.36E-09	0.68	1.06 (1.08, 1.04)	9.83E-09	0.69	1.08 (1.19, 0.99)	8.26E-02	0.75	1.02 (1.06, 0.98)	4.38E-01	-	-	-	0.72	1.01 (1.09, 0.94)	7.70E-01	0.85	1.24 (1.68, 0.92)	1.62E-01	0.2388	0.84	1.03 (0.97, 1.1)	3.35E-01
rs62006566	15	75086534	*CSK*	Yes	C	T	1.05 (1.03, 1.07)	2.69E-08	0.36	1.05 (1.07, 1.04)	1.92E-08	-	-	-	0.8	1.03 (1.08, 0.99)	1.46E-01	-	-	-	0.62	1 (1.08, 0.93)	9.37E-01	0.76	0.94 (1.19, 0.73)	5.89E-01	0.3886	-	-	-
rs7188250	16	53834607	*FTO*	No	C	T	1.06 (1.05, 1.08)	1.55E-13	0.41	1.06 (1.04, 1.08)	2.62E-11	0.42	1.14 (1.05, 1.24)	2.24E-03	0.1	1.06 (1, 1.12)	4.60E-02	0.12	0.8 (0.58, 1.1)	1.70E-01	0.26	1.05 (0.97, 1.13)	2.68E-01	0.19	0.96 (0.74, 1.24)	7.62E-01	0.2719	0.21	1.05 (0.99, 1.11)	1.10E-01
rs2224770	17	2205923	*SMG6*	No	T	C	1.05 (1.03, 1.07)	1.76E-09	0.38	1.05 (1.06, 1.03)	1.47E-06	0.38	1.06 (1.15, 0.97)	1.76E-01	0.63	1.08 (1.12, 1.04)	4.71E-05	-	-	-	0.39	1 (1.07, 0.93)	9.14E-01	0.38	0.92 (1.14, 0.75)	4.49E-01	0.2085	0.24	1.08 (1.03, 1.14)	2.92E-03
rs9907657	17	7460957	*TNFSF12; TNFSF12-TNFSF13*	No	T	G	1.06 (1.04, 1.09)	6.09E-09	0.16	1.07 (1.04, 1.1)	6.78E-08	0.16	1.12 (1, 1.25)	4.50E-02	0.21	1.04 (0.99, 1.09)	9.07E-02	-	-	-	0.12	1.03 (0.92, 1.16)	5.97E-01	0.19	1.03 (0.78, 1.36)	8.38E-01	0.6499	0.21	1 (0.95, 1.06)	9.36E-01
rs2517953	17	37841211	*PGAP3*	No	G	C	1.06 (1.05, 1.08)	5.84E-14	0.36	1.06 (1.08, 1.04)	6.17E-10	0.36	1 (1.09, 0.92)	9.71E-01	0.77	1.11 (1.16, 1.06)	2.23E-06	0.77	1.03 (1.31, 0.82)	7.80E-01	0.46	1.06 (1.13, 0.98)	1.32E-01	0.59	0.95 (1.18, 0.77)	6.62E-01	0.2453	0.5	1.02 (0.98, 1.07)	3.49E-01
rs9912468	17	64318357	*PRKCA*	No	G	C	1.05 (1.03, 1.06)	2.49E-09	0.42	1.04 (1.06, 1.02)	4.35E-06	0.43	1.22 (1.33, 1.12)	2.50E-06	0.37	1.04 (1.08, 1.01)	1.53E-02	0.37	0.88 (1.07, 0.72)	2.03E-01	0.44	1.06 (1.14, 0.99)	8.61E-02	0.48	0.85 (1.04, 0.7)	1.21E-01	0.0010	0.41	1.06 (1.01, 1.11)	1.03E-02
rs145606454	17	65893289	*BPTF*	No	A	AC	1.06 (1.04, 1.08)	3.97E-09	0.21	1.06 (1.04, 1.09)	1.90E-08	0.21	0.99 (0.9, 1.1)	9.05E-01	0.22	1.07 (1.02, 1.12)	8.28E-03	-	-	-	0.27	1.02 (0.94, 1.1)	6.47E-01	0.62	0.88 (0.7, 1.1)	2.49E-01	0.2459	0.68	1.02 (0.97, 1.07)	5.58E-01
rs7229491	18	46516424	*18q21.1*	Yes	C	G	1.05 (1.03, 1.07)	4.82E-09	0.66	1.05 (1.03, 1.07)	1.93E-06	0.67	1.04 (0.95, 1.14)	3.73E-01	0.81	1.09 (1.04, 1.14)	3.84E-04	-	-	-	0.65	1.03 (0.95, 1.1)	4.96E-01	0.44	1.09 (0.88, 1.34)	4.52E-01	0.5947	0.46	1.01 (0.97, 1.06)	6.38E-01
rs56968346	21	30540165	*MAP3K7CL*	No	T	C	1.09 (1.07, 1.11)	4.17E-17	0.15	1.09 (1.06, 1.12)	3.62E-12	0.16	1.07 (0.96, 1.2)	2.03E-01	0.41	1.08 (1.04, 1.12)	1.43E-05	-	-	-	0.2	1.06 (0.97, 1.15)	2.17E-01	0.23	1.16 (0.9, 1.49)	2.52E-01	0.9368	0.1	1.16 (1.08, 1.25)	5.28E-05
rs9608201	22	24166788	*SMARCB1*	No	A	G	1.07 (1.05, 1.09)	3.24E-14	0.79	1.06 (1.04, 1.08)	6.93E-07	0.81	1.11 (1, 1.23)	5.41E-02	0.46	1.1 (1.06, 1.14)	4.40E-07	0.49	1.1 (0.9, 1.33)	3.49E-01	0.72	1.09 (1.01, 1.18)	2.39E-02	0.59	1.13 (0.91, 1.4)	2.69E-01	0.6404	0.61	1.17 (1.11, 1.22)	1.19E-10
**The 3 Genome-wide Significant Loci for HFpEF**																														
rs62048402	16	53803223	*FTO*	No	A	G	1.06 (1.05, 1.08)	1.10E-13	0.4	1.06 (1.05, 1.08)	1.29E-11	0.41	1.05 (0.98, 1.13)	1.80E-01	0.1	1.07 (1.01, 1.14)	2.46E-02	0.12	1.17 (0.89, 1.55)	2.67E-01	0.26	1.04 (0.96, 1.12)	3.83E-01	0.18	1.36 (1.03, 1.81)	2.96E-02	0.5290	0.2	1.06 (1.02, 1.11)	6.34E-03
rs75716431	17	66382647	*ARSG*	No	T	C	1.12 (1.08, 1.17)	4.27E-08	0.02	1.09 (1.01, 1.18)	3.11E-02	0.02	0.89 (0.66, 1.2)	4.42E-01	0.18	1.15 (1.09, 1.21)	4.07E-08	-	-	-	0.04	0.9 (0.74, 1.09)	2.86E-01	0.13	1.44 (1.02, 2.03)	3.91E-02	0.0303	0.12	1.02 (0.96, 1.07)	5.39E-01
rs113752382	21	18617840	*21q21.1*	Yes	T	C	1.12 (1.08, 1.17)	3.39E-08	0.07	1.11 (1.16, 1.07)	1.08E-06	-	-	-	0.03	1.29 (1.52, 1.09)	2.73E-03	-	-	-	0.06	1.09 (1.32, 0.89)	4.15E-01	-	-	-	0.2329	-	-	-

Chr.: Chromosome; EA: Effect Allele; NEA: Non-effect Allele; EAF: Effect Allele Frequency; OR: Odds Ratio; CI: Confidence Interval.

*A total of 9 novel HFrEF loci and 1 novel HFpEF locus as compared to previous all-cause HF GWAS (PMID: 31919418, 36376295, 36517512, 37429843, 37503172, 40038546), defined as non-overlapping physical distance of > 500kb on both sides of the variant.

**Heterogeneity P-values from Cochran's Q test.

Revised Table S3. The 136 Genome-wide Significant Loci for the Meta-analysis of GWAS on All-cause HF Among Multi-ancestry Cohort.

rsID	Chr.	Position (hg19)	Gene	Novel*	EA	NEA	Meta-analysis		MVP European			BIOVU European			MVP African			BIOVU African			MVP Hispanic			MVP Asian			Levin et al. 2022			Mulit-ancestry Heterogeneity p**	Biobank Japan Validation		
							OR (95% CI)	P	EAF	OR (95% CI)	P	EAF	OR (95% CI)	P	EAF	OR (95% CI)	P	EAF	OR (95% CI)	P	EAF	OR (95% CI)	P	EAF	OR (95% CI)	P	EAF	OR (95% CI)	P		EAF	OR (95% CI)	P
rs17035646	1	10796547	*CASZ1*	No	A	G	1.03 (1.02, 1.04)	1.58E-14	0.34	1.03 (1.02, 1.04)	3.02E-05	0.34	1.06 (1, 1.12)	6.33E-02	0.18	1.07 (1.03, 1.1)	9.85E-05	0.19	1.06 (0.89, 1.27)	5.16E-01	0.45	1.03 (0.98, 1.08)	3.15E-01	0.54	0.94 (0.81, 1.11)	4.75E-01	0.4	1.03 (1.02, 1.04)	1.65E-07	0.3361	0.67	1.06 (1.03, 1.09)	4.56E-06
rs2009371	1	16348083	*CLCNKA*	No	T	A	1.04 (1.03, 1.05)	2.22E-21	0.67	1.04 (1.03, 1.06)	6.80E-10	0.67	1.07 (1, 1.13)	4.12E-02	0.74	1.05 (1.02, 1.08)	7.61E-04	0.74	1.12 (0.95, 1.32)	1.67E-01	0.63	1.05 (1, 1.11)	4.10E-02	0.93	1.07 (0.76, 1.5)	6.97E-01	0.69	1.04 (1.02, 1.05)	1.02E-08	0.8275	0.98	0.98 (0.86, 1.11)	7.54E-01
rs116626164	1	50975227	*FAF1*	No	A	T	1.12 (1.08, 1.15)	2.76E-13	0.02	1.12 (1.07, 1.18)	3.04E-06	0.02	1.09 (0.87, 1.36)	4.37E-01	-	-	-	-	-	-	-	-	-	-	-	-	0.02	1.11 (1.07, 1.16)	2.51E-08	0.9427	-	-	-
rs72664341	1	56990753	*PPAP2B*	No	A	C	1.05 (1.03, 1.06)	7.09E-11	0.91	1.06 (1.08, 1.03)	2.19E-06	0.91	1.12 (1.24, 1.01)	3.16E-02	0.93	1.03 (1.08, 0.97)	3.48E-01	-	-	-	0.93	0.96 (1.06, 0.87)	4.50E-01	0.94	1.22 (1.71, 0.87)	2.40E-01	0.91	1.04 (1.06, 1.02)	1.18E-05	0.2447	0.98	1 (0.91, 1.1)	9.67E-01
rs1997997	1	61886046	*NFIA*	No	A	G	1.03 (1.02, 1.04)	1.23E-11	0.53	1.02 (1.04, 1.01)	1.80E-04	-	-	-	0.28	1.03 (1.06, 1)	9.03E-02	-	-	-	0.52	1 (1.05, 0.95)	8.94E-01	0.48	1.22 (1.42, 1.04)	1.54E-02	0.51	1.04 (1.05, 1.02)	4.56E-08	0.1428	0.49	1.03 (1, 1.05)	3.69E-02
rs34517439	1	78450517	*DNAJB4; GIPC2*	No	A	C	1.04 (1.03, 1.06)	3.89E-11	0.11	1.07 (1.04, 1.09)	1.57E-09	0.12	1.08 (0.99, 1.17)	7.63E-02	0.02	1.11 (1.01, 1.22)	3.44E-02	0.02	0.81 (0.47, 1.4)	4.51E-01	0.05	1.05 (0.93, 1.19)	4.15E-01	-	-	-	0.12	1.03 (1.01, 1.04)	2.61E-03	0.0625	-	-	-
rs35054144	1	93237726	*EVI5*	No	C	T	1.04 (1.03, 1.05)	4.24E-09	0.1	1.05 (1.03, 1.07)	1.23E-05	0.1	0.98 (0.89, 1.08)	6.92E-01	0.02	1.15 (1.04, 1.26)	4.88E-03	0.02	1.4 (0.86, 2.27)	1.77E-01	0.06	1.15 (1.04, 1.28)	7.17E-03	0.05	1.55 (1.12, 2.15)	9.04E-03	0.1	1.03 (1.01, 1.05)	1.70E-03	0.0053	0.02	1.03 (0.94, 1.12)	5.45E-01
rs599839	1	109822166	*PSRC1*	No	A	G	1.04 (1.03, 1.05)	3.13E-15	0.77	1.05 (1.03, 1.06)	4.36E-09	0.77	1.08 (1.01, 1.16)	2.17E-02	0.27	1.04 (1, 1.07)	2.26E-02	0.29	0.94 (0.8, 1.1)	4.42E-01	0.74	1.05 (0.99, 1.11)	9.31E-02	0.91	1.29 (0.96, 1.73)	8.96E-02	0.76	1.03 (1.02, 1.04)	8.92E-06	0.2001	0.92	1.04 (0.99, 1.09)	8.33E-02
rs3790604	1	113046879	*WNT2B*	No	A	C	1.04 (1.02, 1.05)	1.24E-08	0.07	1.04 (1.01, 1.06)	3.31E-03	-	-	-	0.02	1.05 (0.95, 1.16)	3.25E-01	-	-	-	0.13	1.04 (0.96, 1.12)	3.16E-01	0.25	0.98 (0.82, 1.17)	8.40E-01	0.17	1.04 (1.02, 1.05)	2.21E-06	0.9793	0.29	1.01 (0.99, 1.04)	2.94E-01
rs1230666	1	114173410	*MAGI3*	No	A	G	1.03 (1.02, 1.04)	3.97E-08	0.14	1.04 (1.06, 1.03)	1.90E-06	0.14	1.02 (1.1, 0.94)	6.99E-01	0.02	1.06 (1.16, 0.98)	1.42E-01	0.03	1.1 (1.75, 0.69)	6.89E-01	0.07	0.99 (1.09, 0.91)	8.79E-01	-	-	-	0.15	1.02 (1.04, 1.01)	2.31E-03	0.5193	-	-	-
rs61818779	1	190319316	*BRINP3*	No	A	G	1.03 (1.02, 1.04)	8.69E-09	0.8	1.03 (1.05, 1.02)	5.41E-05	-	-	-	0.81	1.06 (1.09, 1.03)	5.38E-04	-	-	-	0.73	1.03 (1.09, 0.97)	3.03E-01	0.8	0.92 (1.11, 0.76)	3.80E-01	0.79	1.02 (1.03, 1.01)	1.69E-03	0.1438	0.8	1 (0.97, 1.03)	8.86E-01
rs17163345	1	222806218	*MIA3*	No	G	A	1.03 (1.02, 1.03)	4.95E-10	0.71	1.03 (1.02, 1.05)	2.58E-06	0.71	1.02 (0.96, 1.09)	5.45E-01	0.3	1.03 (1.01, 1.06)	1.75E-02	0.31	1.09 (0.93, 1.27)	2.78E-01	0.53	1.02 (0.97, 1.07)	5.43E-01	0.6	1.03 (0.89, 1.2)	6.71E-01	0.67	1.02 (1.01, 1.03)	3.95E-04	0.7400	0.54	1.02 (0.99, 1.04)	1.54E-01
rs12724121	1	236852282	*ACTN2*	No	T	A	1.02 (1.02, 1.03)	1.01E-09	0.61	1.02 (1.01, 1.03)	1.53E-03	-	-	-	0.18	1.05 (1.01, 1.08)	8.15E-03	-	-	-	0.45	1.03 (0.98, 1.08)	3.06E-01	0.48	0.98 (0.85, 1.14)	8.17E-01	0.58	1.02 (1.01, 1.03)	3.06E-06	0.7201	0.42	1.04 (1.01, 1.06)	1.76E-03
rs66906321	2	630070	-	No	C	T	1.04 (1.03, 1.05)	1.48E-16	0.8	1.05 (1.03, 1.07)	1.45E-08	0.83	1.04 (0.97, 1.13)	2.60E-01	0.84	1.05 (1.01, 1.09)	1.92E-02	-	-	-	0.84	1.03 (0.96, 1.1)	4.13E-01	0.88	1.04 (0.79, 1.36)	7.78E-01	0.83	1.04 (1.02, 1.05)	5.47E-08	0.9463	0.9	1.05 (1.01, 1.09)	2.32E-02
rs1358134	2	6147956	-	No	A	G	1.03 (1.02, 1.03)	1.51E-09	0.75	1.04 (1.05, 1.02)	3.37E-06	-	-	-	0.72	1.05 (1.09, 1.02)	4.69E-04	-	-	-	0.72	1.01 (1.06, 0.95)	8.50E-01	0.59	1.09 (1.28, 0.93)	2.74E-01	0.69	1.02 (1.03, 1.01)	1.62E-03	0.0785	0.58	1.05 (1.02, 1.07)	1.81E-04
rs12995996	2	11563960	*E2F6*	No	C	T	1.03 (1.02, 1.04)	1.48E-10	0.34	1.03 (1.02, 1.05)	2.53E-06	-	-	-	0.1	1.04 (1, 1.09)	6.73E-02	-	-	-	0.47	1.01 (0.96, 1.06)	7.06E-01	0.6	1.03 (0.87, 1.21)	7.63E-01	0.4	1.03 (1.01, 1.04)	3.35E-05	0.8186	-	-	-
rs57372210	2	26990273	*SLC35F6; CENPA*	No	C	T	1.03 (1.02, 1.03)	3.02E-09	0.63	1.03 (1.04, 1.01)	1.50E-04	-	-	-	0.92	1.04 (1.09, 0.99)	1.46E-01	-	-	-	0.57	1.02 (1.07, 0.97)	4.15E-01	0.84	0.98 (1.2, 0.8)	8.51E-01	0.65	1.02 (1.04, 1.01)	1.75E-05	0.9802	0.83	1.01 (0.98, 1.04)	6.39E-01
rs28705335	2	36655721	*CRIM1*	No	C	T	1.02 (1.02, 1.03)	5.00E-09	0.3	1.01 (1, 1.03)	1.05E-01	-	-	-	0.28	1.05 (1.02, 1.08)	1.83E-03	-	-	-	0.28	1.06 (1.01, 1.12)	2.40E-02	0.19	1.07 (0.88, 1.3)	4.87E-01	0.31	1.03 (1.02, 1.04)	7.35E-07	0.1001	0.18	1.02 (0.99, 1.05)	1.98E-01
rs3770770	2	37192866	*STRN*	No	C	T	1.04 (1.03, 1.05)	2.18E-14	0.81	1.05 (1.07, 1.04)	2.98E-10	-	-	-	0.92	1.02 (1.07, 0.97)	3.59E-01	-	-	-	0.75	1.02 (1.08, 0.96)	5.22E-01	0.58	1 (1.17, 0.85)	9.79E-01	0.73	1.03 (1.04, 1.02)	1.17E-06	0.2216	0.44	1.02 (1, 1.04)	1.02E-01
rs887912	2	59302877	-	No	T	C	1.03 (1.02, 1.03)	9.26E-09	0.29	1.03 (1.05, 1.02)	4.23E-06	-	-	-	0.09	1.04 (1.09, 1)	5.44E-02	-	-	-	0.17	1.06 (1.13, 0.99)	8.04E-02	0.03	0.84 (1.33, 0.54)	4.61E-01	0.27	1.02 (1.03, 1.01)	2.61E-03	0.3405	-	-	-
rs13389483	2	76914832	-	Yes	G	A	1.09 (1.06, 1.12)	2.38E-08	-	-	-	-	-	-	0.16	1.07 (1.03, 1.11)	3.15E-04	0.16	1.16 (0.96, 1.39)	1.29E-01	0.02	1.01 (0.81, 1.26)	9.12E-01	0.01	1.2 (0.33, 4.28)	7.83E-01	0.12	1.13 (1.07, 1.2)	1.20E-05	0.4969	-	-	-
rs4832298	2	86764004	*CHMP3; RNF103-CHMP3*	No	C	T	1.03 (1.02, 1.04)	1.32E-09	0.32	1.03 (1.04, 1.01)	3.05E-04	0.32	0.95 (1.01, 0.89)	9.40E-02	0.24	1 (1.04, 0.97)	7.68E-01	-	-	-	0.29	1.01 (1.07, 0.96)	7.54E-01	0.12	0.85 (1.12, 0.65)	2.44E-01	0.3	1.04 (1.05, 1.02)	1.56E-08	0.0296	0.09	0.96 (0.92, 1)	6.78E-02
rs13033664	2	148770274	*ORC4*	Yes	T	G	1.02 (1.01, 1.03)	3.31E-08	0.3	1.02 (1.01, 1.04)	9.51E-04	0.3	1.04 (0.98, 1.1)	2.29E-01	0.18	1.03 (1, 1.07)	4.66E-02	-	-	-	0.35	1 (0.96, 1.06)	8.58E-01	0.44	0.94 (0.8, 1.09)	3.92E-01	0.33	1.02 (1.01, 1.03)	6.81E-05	0.7716	0.44	1.02 (1, 1.05)	8.03E-02
rs74853338	2	201171651	*SPATS2L*	No	T	C	1.03 (1.02, 1.04)	1.57E-12	0.42	1.03 (1.01, 1.04)	3.95E-05	0.44	1.03 (0.97, 1.09)	3.20E-01	0.21	1.02 (0.98, 1.05)	3.42E-01	-	-	-	0.54	1.06 (1.01, 1.11)	2.21E-02	0.68	0.99 (0.84, 1.17)	9.10E-01	0.46	1.03 (1.02, 1.04)	1.13E-07	0.7852	0.69	1.03 (1, 1.05)	4.59E-02
rs7650482	3	12841804	*CAND2*	No	G	A	1.03 (1.02, 1.04)	6.23E-12	0.64	1.02 (1.01, 1.04)	3.90E-04	0.64	1.06 (1, 1.13)	4.88E-02	0.88	1.03 (0.99, 1.08)	1.08E-01	-	-	-	0.49	1.04 (0.99, 1.1)	8.27E-02	0.31	1 (0.85, 1.18)	9.90E-01	0.59	1.03 (1.02, 1.04)	1.61E-07	0.8422	0.3	1.06 (1.04, 1.09)	2.99E-06
rs12629972	3	25121444	-	No	C	T	1.02 (1.01, 1.03)	4.56E-08	0.59	1.02 (1.01, 1.03)	2.13E-03	-	-	-	0.38	1.03 (1, 1.05)	5.05E-02	-	-	-	0.66	0.98 (0.93, 1.03)	3.86E-01	0.74	1.05 (0.87, 1.27)	6.21E-01	0.61	1.02 (1.01, 1.03)	1.33E-05	0.5515	0.76	1.03 (1.01, 1.06)	1.80E-02
rs820430	3	27548900	*SLC4A7*	No	A	G	1.03 (1.02, 1.03)	4.28E-10	0.61	1.03 (1.04, 1.01)	1.36E-04	0.62	1.06 (1.13, 1)	3.71E-02	0.92	1.05 (1.11, 1)	5.11E-02	0.92	1.03 (1.36, 0.77)	8.59E-01	0.66	1.02 (1.08, 0.97)	4.16E-01	0.38	1.02 (1.2, 0.87)	8.03E-01	0.58	1.02 (1.03, 1.01)	2.01E-05	0.8321	0.37	1.02 (0.99, 1.04)	1.50E-01
rs11710802	3	43930155	-	No	T	G	1.03 (1.02, 1.04)	4.83E-10	0.75	1.03 (1.04, 1.01)	9.87E-04	0.75	1 (1.07, 0.94)	9.04E-01	0.94	1.06 (1.12, 1)	4.98E-02	0.94	1.14 (1.56, 0.83)	4.11E-01	0.76	1.05 (1.12, 0.99)	8.16E-02	0.54	1.12 (1.32, 0.95)	1.78E-01	0.67	1.03 (1.04, 1.02)	2.44E-06	0.7031	0.43	1.03 (1.01, 1.06)	6.36E-03
rs58339610	3	49845006	*UBA7*	No	A	T	1.03 (1.02, 1.04)	7.93E-10	0.18	1.03 (1.01, 1.05)	2.75E-04	-	-	-	0.26	1.04 (1.01, 1.07)	8.81E-03	-	-	-	0.14	1.05 (0.98, 1.13)	1.85E-01	0.02	0.71 (0.41, 1.23)	2.22E-01	0.17	1.03 (1.02, 1.05)	4.53E-05	0.6919	-	-	-
rs35593046	3	53553923	*CACNA1D*	No	G	T	1.02 (1.02, 1.03)	3.22E-08	0.75	1.03 (1.04, 1.01)	3.15E-04	-	-	-	0.85	0.98 (1.02, 0.94)	2.59E-01	-	-	-	0.73	0.99 (1.05, 0.93)	7.94E-01	0.53	1.14 (1.35, 0.97)	1.09E-01	0.69	1.03 (1.04, 1.02)	1.57E-06	0.0599	0.44	1.07 (1.05, 1.1)	1.20E-08
rs34330586	3	135800409	*PPP2R3A*	No	A	T	1.03 (1.02, 1.04)	2.43E-10	0.77	1.04 (1.06, 1.02)	3.01E-06	-	-	-	-	-	-	-	-	-	0.76	1.08 (1.15, 1.02)	1.31E-02	0.86	1.09 (1.41, 0.85)	4.79E-01	0.77	1.02 (1.04, 1.01)	7.21E-05	0.2290	0.82	1.04 (1.01, 1.07)	1.25E-02
rs77463690	3	168853335	*MECOM*	No	A	G	1.05 (1.03, 1.06)	1.11E-09	0.92	1.04 (1.07, 1.02)	1.73E-03	0.92	1.14 (1.27, 1.02)	2.32E-02	0.96	1.05 (1.13, 0.97)	2.00E-01	-	-	-	0.92	1.1 (1.21, 1)	3.95E-02	0.85	1.22 (1.57, 0.95)	1.16E-01	0.92	1.04 (1.06, 1.02)	1.23E-05	0.3909	0.9	1.02 (0.98, 1.06)	3.35E-01
rs4912532	3	184000619	*EIF2B5; ECE2*	No	G	T	1.02 (1.02, 1.03)	6.21E-11	0.5	1.03 (1.04, 1.01)	7.21E-05	-	-	-	0.45	1.02 (1.05, 1)	6.47E-02	-	-	-	0.4	1.03 (1.08, 0.98)	2.81E-01	0.57	0.88 (1.03, 0.76)	1.16E-01	0.5	1.02 (1.03, 1.01)	1.00E-06	0.4630	0.52	1.01 (0.99, 1.03)	4.62E-01
rs34811474	4	25408838	*ANAPC4*	No	G	A	1.03 (1.02, 1.05)	8.42E-12	0.78	1.04 (1.05, 1.02)	1.79E-06	0.79	1.04 (1.11, 0.97)	3.32E-01	0.96	1.05 (1.12, 0.98)	1.39E-01	0.95	1.29 (1.88, 0.89)	1.76E-01	0.87	1.05 (1.13, 0.97)	2.05E-01	0.98	1.28 (2.45, 0.67)	4.46E-01	0.78	1.03 (1.05, 1.02)	1.04E-05	0.8726	-	-	-
rs7662342	4	26805393	-	No	A	G	1.02 (1.01, 1.03)	3.65E-08	0.4	1.01 (1, 1.02)	8.07E-02	0.4	1.03 (0.97, 1.09)	2.98E-01	0.18	1.04 (1.01, 1.08)	2.22E-02	0.19	1.07 (0.9, 1.28)	4.57E-01	0.45	1 (0.95, 1.05)	9.11E-01	0.51	1.05 (0.9, 1.23)	5.28E-01	0.43	1.03 (1.02, 1.04)	4.96E-07	0.4755	0.51	1 (0.98, 1.03)	9.19E-01
rs12507026	4	45181334	-	No	T	A	1.04 (1.03, 1.04)	2.97E-21	0.43	1.03 (1.02, 1.05)	5.25E-07	0.43	1.03 (0.97, 1.09)	3.61E-01	0.24	1.04 (1.01, 1.07)	1.76E-02	0.23	1.07 (0.9, 1.27)	4.23E-01	0.36	1.07 (1.02, 1.13)	6.55E-03	0.24	1.05 (0.88, 1.25)	5.92E-01	0.42	1.04 (1.03, 1.05)	6.09E-13	0.8858	0.3	1.03 (1.01, 1.06)	1.39E-02
rs1383722	4	67879681	-	No	A	G	1.03 (1.02, 1.03)	2.78E-08	0.25	1.02 (1.03, 1)	5.28E-02	0.23	1.01 (1.08, 0.94)	7.64E-01	0.26	1.02 (1.05, 0.99)	1.90E-01	-	-	-	0.23	1.02 (1.09, 0.96)	5.23E-01	0.09	1.23 (1.79, 0.85)	2.73E-01	0.22	1.03 (1.05, 1.02)	1.43E-07	0.4897	0.04	0.99 (0.93, 1.05)	7.55E-01
rs1398018	4	77372923	*SHROOM3*	Yes	T	C	1.02 (1.01, 1.03)	2.27E-08	0.57	1.03 (1.04, 1.01)	7.91E-05	0.57	1.06 (1.12, 1)	6.88E-02	0.87	0.99 (1.03, 0.95)	7.03E-01	0.86	0.93 (1.15, 0.75)	4.97E-01	0.64	1.05 (1.11, 1)	4.11E-02	0.91	0.7 (0.93, 0.53)	1.33E-02	0.6	1.02 (1.03, 1.01)	1.66E-04	0.0449	0.91	1.01 (0.97, 1.05)	5.73E-01
rs59788391	4	111701433	*PITX2*	No	G	A	1.08 (1.07, 1.1)	2.03E-49	0.11	1.08 (1.06, 1.1)	1.74E-13	0.12	1.13 (1.04, 1.24)	5.05E-03	0.04	1.02 (0.95, 1.1)	5.74E-01	0.03	1.13 (0.75, 1.72)	5.54E-01	0.19	1.09 (1.03, 1.16)	4.87E-03	0.39	1.07 (0.91, 1.26)	3.86E-01	0.22	1.09 (1.07, 1.1)	6.55E-35	0.5908	0.44	1.14 (1.11, 1.17)	1.44E-27
rs17620390	4	114384328	*CAMK2D*	No	C	A	1.03 (1.02, 1.04)	1.95E-11	0.26	1.03 (1.02, 1.05)	1.61E-05	0.27	1.04 (0.98, 1.11)	1.98E-01	0.11	1.04 (0.99, 1.08)	1.05E-01	0.12	1.4 (1.13, 1.73)	2.28E-03	0.19	1.03 (0.97, 1.09)	4.01E-01	0.09	0.95 (0.73, 1.24)	7.28E-01	0.25	1.03 (1.02, 1.04)	6.31E-06	0.2042	0.05	1 (0.95, 1.06)	9.05E-01
rs3106209	4	140884723	*MAML3*	No	C	A	1.03 (1.02, 1.03)	4.16E-10	0.66	1.02 (1.01, 1.03)	4.09E-03	0.68	1.05 (0.99, 1.11)	1.37E-01	-	-	-	-	-	-	0.78	1.06 (0.99, 1.13)	7.19E-02	0.67	1.16 (0.98, 1.36)	8.11E-02	0.67	1.03 (1.02, 1.04)	3.32E-07	0.3837	0.73	1.03 (1, 1.06)	3.63E-02
rs1309540	5	64293570	*CWC27*	No	A	G	1.03 (1.02, 1.04)	1.64E-08	0.15	1.03 (1.01, 1.04)	2.73E-03	0.16	1.06 (0.98, 1.14)	1.58E-01	0.06	1.09 (1.03, 1.15)	1.36E-03	-	-	-	0.27	1.02 (0.96, 1.08)	5.48E-01	0.13	0.97 (0.78, 1.21)	8.00E-01	0.14	1.03 (1.01, 1.04)	7.33E-05	0.3683	0.16	1.04 (1.01, 1.07)	1.49E-02
rs12514158	5	95857763	*CAST*	No	C	T	1.03 (1.02, 1.03)	3.43E-11	0.42	1.02 (1.04, 1.01)	5.26E-04	0.4	1.06 (1.13, 1)	3.61E-02	0.51	1.02 (1.05, 0.99)	1.14E-01	-	-	-	0.5	1.03 (1.09, 0.98)	1.85E-01	0.49	1.03 (1.21, 0.88)	6.91E-01	0.45	1.03 (1.04, 1.02)	6.17E-07	0.8701	0.4	1.03 (1.01, 1.06)	7.07E-03
rs11745324	5	137012171	*KLHL3*	No	G	A	1.03 (1.02, 1.04)	3.45E-10	0.77	1.03 (1.04, 1.01)	1.05E-03	-	-	-	0.88	1 (1.04, 0.96)	9.20E-01	-	-	-	0.83	1.03 (1.1, 0.97)	3.23E-01	0.93	0.88 (1.2, 0.65)	4.12E-01	0.78	1.04 (1.05, 1.02)	1.76E-08	0.3206	0.94	1.04 (0.99, 1.1)	1.12E-01
rs11956224	5	138773874	*DNAJC18*	No	G	A	1.03 (1.02, 1.04)	2.12E-10	0.68	1.03 (1.01, 1.04)	3.86E-04	-	-	-	0.32	1.04 (1.01, 1.07)	6.89E-03	-	-	-	0.62	1.02 (0.97, 1.08)	3.62E-01	0.33	0.95 (0.8, 1.13)	5.86E-01	0.6	1.03 (1.02, 1.05)	3.91E-06	0.7724	0.42	1.04 (1.02, 1.07)	3.10E-04
rs61752263	5	142752480	*NR3C1*	No	T	C	1.04 (1.03, 1.05)	1.63E-10	0.15	1.04 (1.02, 1.06)	1.17E-05	0.16	1.03 (0.95, 1.11)	5.10E-01	0.03	1.12 (1.04, 1.22)	3.20E-03	0.03	1.62 (1.08, 2.42)	1.90E-02	0.07	1.07 (0.98, 1.18)	1.50E-01	0.01	0.87 (0.37, 2.01)	7.40E-01	0.16	1.03 (1.01, 1.05)	7.76E-05	0.1026	-	-	-
rs6891790	5	172670745	*NKX2-5*	No	G	T	1.02 (1.02, 1.03)	2.70E-08	0.71	1.03 (1.04, 1.01)	2.00E-04	-	-	-	0.42	1.04 (1.07, 1.01)	6.90E-03	-	-	-	0.53	1 (1.06, 0.95)	8.65E-01	0.39	0.95 (1.12, 0.8)	5.27E-01	0.63	1.02 (1.03, 1.01)	3.76E-04	0.5296	0.35	1.03 (1, 1.05)	6.66E-02
rs6885658	5	173307137	*CPEB4*	No	C	T	1.03 (1.02, 1.04)	2.19E-09	0.71	1.03 (1.04, 1.01)	3.30E-04	-	-	-	0.91	1.03 (1.07, 0.98)	2.74E-01	-	-	-	0.83	1.02 (1.09, 0.95)	5.92E-01	0.92	0.86 (1.15, 0.65)	3.24E-01	0.69	1.03 (1.04, 1.02)	2.63E-06	0.8382	0.9	1.03 (0.99, 1.07)	1.62E-01
rs9349379	6	12903957	*PHACTR1*	No	G	A	1.02 (1.02, 1.03)	2.59E-09	0.4	1.03 (1.02, 1.05)	3.01E-07	0.41	1.04 (0.98, 1.1)	1.76E-01	0.08	0.99 (0.94, 1.04)	6.22E-01	0.09	1.14 (0.89, 1.46)	3.03E-01	0.35	1.07 (1.02, 1.12)	9.35E-03	0.59	1.06 (0.91, 1.23)	4.86E-01	0.44	1.02 (1.01, 1.03)	2.36E-03	0.1074	0.66	1 (0.98, 1.03)	7.96E-01
rs7766436	6	22598259	*HDGFL1*	No	T	C	1.04 (1.03, 1.04)	4.70E-18	0.3	1.04 (1.03, 1.06)	9.59E-10	0.3	1.02 (0.96, 1.08)	5.66E-01	0.32	1.05 (1.02, 1.07)	1.47E-03	0.31	0.97 (0.83, 1.14)	7.14E-01	0.3	1.02 (0.97, 1.07)	5.13E-01	0.17	0.86 (0.7, 1.05)	1.37E-01	0.27	1.03 (1.02, 1.04)	2.05E-08	0.3445	0.23	1.06 (1.03, 1.09)	6.12E-05
rs6938239	6	34683635	*C6orf106*	No	G	A	1.04 (1.03, 1.05)	8.46E-13	0.17	1.04 (1.06, 1.02)	3.71E-05	0.15	1.1 (1.19, 1.02)	1.82E-02	0.22	1.01 (1.05, 0.98)	3.70E-01	0.21	1.04 (1.23, 0.87)	6.92E-01	0.13	1.1 (1.19, 1.02)	1.69E-02	0.04	0.84 (1.42, 0.49)	5.13E-01	0.16	1.04 (1.05, 1.02)	1.22E-07	0.3462	-	-	-
rs3176326	6	36647289	*CDKN1A*	No	G	A	1.07 (1.06, 1.08)	7.26E-42	0.8	1.08 (1.09, 1.06)	2.27E-19	-	-	-	0.81	1.08 (1.11, 1.04)	1.23E-05	-	-	-	0.83	1.05 (1.12, 0.99)	1.24E-01	0.79	1.01 (1.22, 0.83)	9.59E-01	0.81	1.06 (1.08, 1.05)	3.96E-20	0.6886	0.9	1.11 (1.07, 1.16)	1.23E-07
rs62395884	6	40383533	*LRFN2*	No	T	C	1.02 (1.02, 1.03)	1.89E-08	0.25	1.03 (1.02, 1.05)	7.18E-05	0.26	1.03 (0.97, 1.1)	3.27E-01	0.11	1.02 (0.98, 1.07)	2.87E-01	0.1	0.85 (0.67, 1.08)	1.74E-01	0.16	1.05 (0.99, 1.13)	1.28E-01	0.2	1.06 (0.87, 1.28)	5.76E-01	0.26	1.02 (1.01, 1.03)	2.40E-04	0.6594	0.32	1.01 (0.99, 1.04)	3.55E-01
rs7742789	6	43345803	*ZNF318*	No	T	C	1.03 (1.02, 1.04)	3.63E-11	0.31	1.03 (1.02, 1.05)	9.06E-07	-	-	-	0.67	1.04 (1.01, 1.07)	4.21E-03	-	-	-	0.4	1.03 (0.98, 1.08)	2.30E-01	0.36	1.01 (0.86, 1.18)	9.34E-01	0.33	1.02 (1.01, 1.03)	1.84E-04	0.4428	0.35	1.02 (0.99, 1.04)	1.42E-01
rs3798519	6	50788778	*TFAP2B*	No	C	A	1.03 (1.02, 1.04)	2.61E-10	0.18	1.03 (1.01, 1.04)	1.29E-03	0.18	1.02 (0.94, 1.09)	6.65E-01	0.12	1.05 (1.01, 1.09)	2.00E-02	0.14	1.04 (0.85, 1.27)	6.98E-01	0.32	1.06 (1.01, 1.12)	2.71E-02	0.27	1.04 (0.88, 1.23)	6.39E-01	0.21	1.03 (1.02, 1.04)	4.52E-06	0.9017	0.29	1.05 (1.02, 1.07)	5.16E-04
rs816373	6	54005043	*MLIP*	No	G	A	1.03 (1.02, 1.03)	5.57E-11	0.36	1.03 (1.01, 1.04)	6.31E-05	0.36	1.05 (0.99, 1.12)	9.35E-02	0.67	1.04 (1.01, 1.06)	1.47E-02	-	-	-	0.48	1.07 (1.02, 1.12)	5.21E-03	0.3	1 (0.84, 1.18)	9.57E-01	0.36	1.02 (1.01, 1.03)	7.24E-05	0.3875	0.38	1.03 (1.01, 1.06)	1.07E-02
rs9343870	6	79789473	*PHIP*	No	G	T	1.03 (1.02, 1.04)	5.05E-13	0.38	1.03 (1.02, 1.04)	5.92E-06	0.39	1.03 (0.97, 1.09)	3.16E-01	0.13	1.01 (0.97, 1.05)	6.61E-01	0.13	1.37 (1.11, 1.69)	2.84E-03	0.35	1.05 (1, 1.11)	5.87E-02	0.64	1.08 (0.91, 1.29)	3.61E-01	0.42	1.03 (1.02, 1.04)	2.28E-07	0.1463	0.62	1.01 (0.99, 1.04)	4.38E-01
rs17789218	6	100600097	-	No	C	T	1.03 (1.02, 1.04)	2.36E-08	0.25	1.02 (1.01, 1.04)	3.68E-03	-	-	-	0.05	1.01 (0.95, 1.08)	6.84E-01	-	-	-	0.17	1.1 (1.03, 1.17)	4.24E-03	0.02	1.02 (0.58, 1.81)	9.41E-01	0.22	1.03 (1.02, 1.04)	1.11E-05	0.2868	-	-	-
rs10455872	6	161010118	*LPA*	No	G	A	1.11 (1.09, 1.12)	6.52E-36	0.07	1.12 (1.09, 1.14)	7.53E-19	0.07	1.22 (1.1, 1.36)	1.88E-04	0.01	1.01 (0.9, 1.13)	9.17E-01	0.01	0.92 (0.48, 1.75)	7.93E-01	0.04	1.15 (1.02, 1.3)	2.44E-02	-	-	-	0.06	1.1 (1.07, 1.12)	3.34E-16	0.1633	-	-	-
rs1990622	7	12283787	*TMEM106B*	No	G	A	1.02 (1.02, 1.03)	3.08E-09	0.41	1.03 (1.01, 1.04)	5.35E-05	-	-	-	0.68	1.03 (1, 1.06)	4.18E-02	-	-	-	0.53	1.04 (0.99, 1.09)	1.59E-01	0.56	1.01 (0.86, 1.19)	8.79E-01	0.44	1.02 (1.01, 1.03)	1.77E-04	0.8609	0.61	1.01 (0.99, 1.04)	3.87E-01
rs1474347	7	22768124	*IL6*	Yes	C	A	1.02 (1.01, 1.03)	3.83E-08	0.42	1.02 (1.03, 1.01)	1.76E-03	-	-	-	0.15	1.01 (1.05, 0.98)	4.89E-01	-	-	-	0.21	1.08 (1.15, 1.02)	1.23E-02	0.04	0.84 (1.31, 0.54)	4.35E-01	0.45	1.02 (1.03, 1.01)	3.00E-05	0.3624	-	-	-
rs2023843	7	27243221	*HOXA13*	No	T	C	1.04 (1.03, 1.05)	9.30E-10	0.91	1.04 (1.01, 1.06)	1.65E-03	0.93	0.96 (0.87, 1.07)	5.00E-01	0.88	1.07 (1.02, 1.11)	2.85E-03	-	-	-	0.85	1.11 (1.04, 1.2)	3.55E-03	0.69	1.2 (1.01, 1.42)	3.60E-02	0.8	1.03 (1.02, 1.05)	7.12E-05	0.0662	0.6	1.03 (1, 1.05)	1.86E-02
rs77413112	7	32615191	*AVL9*	Yes	C	T	1.32 (1.2, 1.45)	1.48E-08	-	-	-	-	-	-	0.01	1.36 (1.22, 1.52)	3.78E-08	-	-	-	-	-	-	-	-	-	0.01	1.19 (0.98, 1.45)	7.36E-02	0.2485	-	-	-
rs35005436	7	74134911	*GTF2I*	No	C	T	1.05 (1.04, 1.06)	1.13E-15	0.15	1.05 (1.03, 1.07)	1.83E-07	-	-	-	0.07	1.02 (0.96, 1.07)	5.42E-01	-	-	-	0.1	1.04 (0.95, 1.13)	3.88E-01	0.03	0.92 (0.58, 1.46)	7.09E-01	0.14	1.05 (1.03, 1.07)	8.01E-10	0.7918	0.03	1.06 (0.99, 1.14)	1.09E-01
rs200944075	7	76654895	*UPK3B*	No	C	A	1.07 (1.04, 1.09)	1.09E-08	0.11	1.06 (1.04, 1.09)	8.03E-07	-	-	-	0.04	1.13 (1.03, 1.24)	8.64E-03	-	-	-	0.16	1.08 (0.99, 1.19)	7.63E-02	0.23	1.05 (0.82, 1.35)	7.01E-01	-	-	-	0.6136	-	-	-
rs66939011	7	92265798	*CDK6*	No	T	C	1.03 (1.02, 1.04)	5.08E-12	0.72	1.03 (1.05, 1.02)	1.21E-05	0.72	1.04 (1.11, 0.98)	1.83E-01	0.85	1.02 (1.05, 0.98)	4.08E-01	0.84	0.95 (1.15, 0.78)	6.03E-01	0.66	0.99 (1.05, 0.95)	8.24E-01	0.88	0.82 (1.04, 0.65)	9.92E-02	0.74	1.03 (1.04, 1.02)	4.55E-08	0.3062	0.89	1.04 (1, 1.08)	5.52E-02
rs11773845	7	116191301	*CAV1*	No	A	C	1.03 (1.02, 1.03)	4.94E-11	0.58	1.02 (1.01, 1.04)	4.82E-04	0.59	1.09 (1.03, 1.15)	4.96E-03	0.34	1.05 (1.02, 1.08)	6.02E-04	0.36	1.18 (1.01, 1.37)	3.21E-02	0.6	1.02 (0.98, 1.08)	3.34E-01	0.73	1.13 (0.95, 1.34)	1.61E-01	0.59	1.02 (1.01, 1.03)	6.30E-05	0.0637	0.66	1.04 (1.02, 1.07)	9.30E-04
rs60324735	7	128465450	*CCDC136*	No	A	G	1.03 (1.02, 1.05)	3.17E-10	0.88	1.05 (1.07, 1.03)	1.25E-06	0.89	1.12 (1.23, 1.02)	1.45E-02	0.79	1.07 (1.11, 1.04)	1.36E-05	-	-	-	0.88	0.99 (1.07, 0.92)	7.81E-01	0.81	0.89 (1.08, 0.74)	2.32E-01	0.85	1.02 (1.03, 1.01)	5.89E-03	0.0033	0.74	1.03 (1, 1.06)	4.68E-02
rs7789146	7	150661409	*KCNH2*	No	G	A	1.03 (1.02, 1.04)	1.35E-09	0.82	1.03 (1.05, 1.01)	5.95E-04	0.82	1.08 (1.16, 1)	4.65E-02	0.64	1.01 (1.04, 0.98)	4.18E-01	0.66	0.96 (1.11, 0.83)	6.15E-01	0.87	1.01 (1.08, 0.94)	8.63E-01	0.68	1.08 (1.26, 0.92)	3.47E-01	0.78	1.03 (1.04, 1.02)	1.11E-06	0.5693	0.71	1.03 (1, 1.06)	2.11E-02
rs1182384	7	157040642	*UBE3C*	Yes	G	A	1.02 (1.01, 1.03)	4.22E-08	0.63	1.01 (1, 1.03)	2.54E-02	-	-	-	0.18	1.02 (0.99, 1.05)	2.64E-01	-	-	-	0.54	1.04 (0.99, 1.09)	9.90E-02	0.58	0.95 (0.82, 1.11)	5.48E-01	0.62	1.03 (1.02, 1.04)	7.12E-07	0.3975	0.47	0.99 (0.97, 1.01)	3.36E-01
rs10096633	8	19830921	*LPL*	No	C	T	1.04 (1.03, 1.05)	5.40E-12	0.87	1.04 (1.06, 1.02)	5.31E-05	0.87	1.07 (1.17, 0.99)	1.04E-01	0.57	1.02 (1.05, 1)	9.56E-02	0.59	1.12 (1.29, 0.97)	1.30E-01	0.86	1.12 (1.2, 1.04)	2.43E-03	0.91	0.98 (1.26, 0.76)	8.56E-01	0.85	1.04 (1.05, 1.02)	3.59E-06	0.2925	0.88	1.02 (0.99, 1.06)	1.84E-01

rs11774829	8	105978368	*RP11-127H5.1*	No	T	A	1.04 (1.03, 1.06)	7.89E-10	0.91	1.04 (1.06, 1.01)	1.35E-03	-	-	-	0.97	1.05 (1.14, 0.97)	2.46E-01	-	-	-	0.94	1.1 (1.23, 0.98)	9.23E-02	-	-	-	0.88	1.04 (1.06, 1.03)	8.91E-07	0.7966	-	-	-
rs34866937	8	125859850	-	No	G	A	1.02 (1.02, 1.03)	2.98E-09	0.69	1.03 (1.04, 1.01)	3.07E-04	0.68	1.06 (1.12, 0.99)	8.02E-02	0.79	1.03 (1.07, 1)	5.19E-02	-	-	-	0.71	0.99 (1.04, 0.94)	6.78E-01	0.58	1.08 (1.27, 0.93)	3.15E-01	0.66	1.02 (1.03, 1.01)	2.62E-05	0.6235	0.49	1.03 (1.01, 1.06)	6.16E-03
rs1333047	9	22124504	*RP11-145E5.5*	No	T	A	1.05 (1.04, 1.06)	2.80E-39	0.5	1.07 (1.06, 1.08)	8.91E-25	0.5	1.04 (0.98, 1.1)	2.12E-01	0.88	1.03 (0.98, 1.08)	2.04E-01	0.89	0.99 (0.78, 1.26)	9.38E-01	0.57	1.06 (1.01, 1.12)	1.58E-02	0.65	1.11 (0.95, 1.3)	1.95E-01	0.51	1.04 (1.03, 1.05)	3.03E-16	0.0871	0.66	1.03 (1.01, 1.06)	1.52E-02
rs10993372	9	97619090	*C9orf3*	No	T	A	1.03 (1.02, 1.04)	2.17E-09	-	-	-	0.41	1.02 (0.96, 1.08)	5.15E-01	0.17	1.03 (1, 1.07)	8.51E-02	0.17	1 (0.82, 1.21)	9.61E-01	0.3	1.08 (1.02, 1.14)	9.19E-03	0.22	0.88 (0.72, 1.08)	2.33E-01	0.37	1.03 (1.02, 1.04)	7.50E-08	0.4246	0.28	1.04 (1.01, 1.07)	2.38E-03
rs2270204	9	131042734	*SWI5*	No	G	T	1.02 (1.02, 1.03)	3.14E-08	0.25	1.03 (1.01, 1.04)	5.70E-04	-	-	-	0.7	1.02 (0.99, 1.05)	1.99E-01	-	-	-	0.37	1.01 (0.96, 1.07)	6.25E-01	-	-	-	0.33	1.02 (1.01, 1.04)	3.13E-05	0.9427	0.59	1.03 (1.01, 1.06)	7.80E-03
rs600038	9	136151806	*SURF6*	No	C	T	1.05 (1.04, 1.06)	8.75E-26	0.22	1.04 (1.03, 1.06)	5.00E-08	0.21	0.99 (0.92, 1.06)	7.76E-01	0.14	1.06 (1.03, 1.1)	9.17E-04	0.13	1.1 (0.89, 1.35)	3.84E-01	0.17	1.04 (0.98, 1.11)	2.30E-01	0.18	1.17 (0.97, 1.42)	1.07E-01	0.22	1.05 (1.04, 1.06)	5.92E-17	0.4825	0.28	1.04 (1.01, 1.07)	2.33E-03
rs4328127	10	22298641	*DNAJC1*	No	C	G	1.03 (1.02, 1.04)	2.17E-08	0.28	1.03 (1.04, 1.01)	4.50E-04	-	-	-	0.11	1.04 (1.09, 0.99)	1.58E-01	-	-	-	0.27	1.04 (1.1, 0.98)	2.26E-01	0.08	0.91 (1.35, 0.61)	6.42E-01	0.24	1.03 (1.04, 1.01)	6.94E-05	0.9625	0.04	1 (0.93, 1.07)	9.64E-01
rs34163229	10	75406912	*SYNPO2L*	No	G	T	1.05 (1.04, 1.06)	1.28E-20	0.85	1.06 (1.08, 1.04)	3.96E-10	0.86	1.08 (1.18, 0.99)	7.08E-02	0.8	1.03 (1.07, 1)	3.24E-02	0.8	1.19 (1.42, 0.99)	6.87E-02	0.87	1.02 (1.09, 0.95)	6.54E-01	0.82	1.18 (1.43, 0.97)	1.02E-01	0.84	1.04 (1.06, 1.03)	2.91E-10	0.3898	0.79	1.04 (1.01, 1.07)	1.21E-02
rs67382505	10	79636506	*DLG5*	Yes	G	GA	1.15 (1.1, 1.21)	1.63E-08	-	-	-	-	-	-	0.87	1.15 (1.09, 1.21)	4.60E-07	-	-	-	0.91	1.23 (1.06, 1.43)	7.04E-03	-	-	-	-	-	-	0.3868	-	-	-
rs4934169	10	82203663	*TSPAN14*	No	A	G	1.03 (1.02, 1.04)	2.87E-11	0.28	1.03 (1.05, 1.02)	4.80E-06	-	-	-	0.12	1.01 (1.05, 0.96)	7.61E-01	-	-	-	0.2	1.04 (1.11, 0.98)	1.96E-01	0.08	1.03 (1.46, 0.72)	8.89E-01	0.26	1.03 (1.04, 1.02)	1.54E-06	0.8387	0.02	1.06 (0.97, 1.15)	1.98E-01
rs1332329	10	91003419	*LIPA*	Yes	C	A	1.02 (1.01, 1.03)	3.52E-08	0.34	1.03 (1.01, 1.04)	1.37E-04	0.35	0.94 (0.88, 1)	4.25E-02	0.41	1.02 (0.99, 1.05)	1.18E-01	0.42	1.05 (0.9, 1.21)	5.51E-01	0.45	1.03 (0.98, 1.08)	2.23E-01	0.37	0.89 (0.76, 1.04)	1.52E-01	0.36	1.02 (1.01, 1.03)	4.73E-05	0.0878	0.24	1.02 (0.99, 1.05)	1.88E-01
rs79159857	10	104296418	*SUFU*	No	G	C	1.04 (1.03, 1.05)	3.02E-10	0.11	1.04 (1.02, 1.06)	2.25E-04	0.11	1.11 (1.01, 1.22)	2.35E-02	0.02	1.06 (0.97, 1.16)	2.06E-01	0.03	1.17 (0.75, 1.84)	4.86E-01	0.06	0.99 (0.9, 1.1)	9.02E-01	0.13	0.86 (0.69, 1.08)	2.09E-01	0.12	1.04 (1.02, 1.06)	1.44E-06	0.4484	0.06	1.06 (1.01, 1.11)	2.07E-02
rs17617337	10	121426884	*BAG3*	No	C	T	1.04 (1.03, 1.05)	5.34E-17	0.79	1.04 (1.06, 1.02)	3.81E-07	0.8	1.16 (1.25, 1.08)	7.45E-05	0.96	1.03 (1.11, 0.97)	3.26E-01	0.96	1.44 (2.14, 0.97)	6.91E-02	0.89	1 (1.08, 0.92)	9.42E-01	0.97	0.95 (1.57, 0.57)	8.32E-01	0.78	1.04 (1.06, 1.03)	1.07E-09	0.0574	-	-	-
rs569550	11	1887068	*LSP1*	No	G	T	1.02 (1.02, 1.03)	3.61E-09	0.39	1.03 (1.01, 1.04)	1.10E-04	-	-	-	0.17	1.01 (0.98, 1.05)	4.17E-01	-	-	-	0.54	0.99 (0.94, 1.04)	7.88E-01	0.59	1.09 (0.93, 1.28)	2.76E-01	0.44	1.02 (1.01, 1.03)	7.20E-06	0.6590	0.75	1.05 (1.02, 1.08)	1.52E-03
rs1450265	11	10335700	*AMPD3*	No	G	T	1.04 (1.02, 1.05)	4.70E-10	0.12	1.05 (1.03, 1.07)	2.58E-06	0.12	1.04 (0.95, 1.13)	4.39E-01	0.14	1.03 (0.99, 1.07)	1.48E-01	0.14	1 (0.81, 1.24)	9.71E-01	0.09	1.05 (0.97, 1.15)	2.40E-01	0.01	0.93 (0.46, 1.89)	8.36E-01	0.15	1.03 (1.01, 1.05)	1.06E-04	0.8940	-	-	-
rs10741694	11	16286183	*SOX6*	Yes	T	C	1.02 (1.01, 1.03)	3.71E-08	0.39	1.02 (1.03, 1)	8.60E-03	0.38	1.04 (1.1, 0.98)	2.01E-01	0.39	1.02 (1.05, 1)	9.75E-02	0.38	1.14 (1.31, 0.99)	7.21E-02	0.43	1.05 (1.1, 1)	6.26E-02	0.25	0.98 (1.18, 0.82)	8.56E-01	0.34	1.02 (1.03, 1.01)	4.15E-05	0.6595	-	-	-
rs988748	11	27724745	*BDNF*	No	G	C	1.04 (1.03, 1.05)	2.10E-14	0.78	1.05 (1.03, 1.07)	5.70E-10	0.79	1.03 (0.96, 1.1)	4.45E-01	0.92	1.02 (0.96, 1.07)	5.79E-01	0.93	1.18 (0.88, 1.57)	2.72E-01	0.81	1.04 (0.98, 1.11)	1.94E-01	0.53	1.07 (0.92, 1.25)	3.71E-01	0.75	1.03 (1.02, 1.04)	3.93E-06	0.4233	0.6	1.01 (0.98, 1.03)	5.66E-01
rs7936836	11	43633645	*HSD17B12*	No	C	A	1.03 (1.02, 1.04)	3.71E-13	0.6	1.03 (1.04, 1.01)	3.25E-05	-	-	-	0.9	1.03 (1.08, 0.98)	2.28E-01	-	-	-	0.49	1.03 (1.08, 0.98)	2.87E-01	0.4	1.14 (1.33, 0.97)	1.15E-01	0.59	1.03 (1.04, 1.02)	1.46E-08	0.8017	-	-	-
rs2904129	11	47715574	*AGBL2*	No	C	T	1.05 (1.03, 1.06)	7.49E-10	0.06	1.04 (1.02, 1.07)	2.04E-03	0.06	1 (0.88, 1.13)	9.93E-01	0.2	1.06 (1.03, 1.09)	4.22E-04	-	-	-	0.05	1 (0.89, 1.11)	9.58E-01	-	-	-	0.08	1.05 (1.03, 1.07)	2.48E-05	0.7627	-	-	-
rs58786959	11	49185414	*FOLH1*	Yes	A	G	1.04 (1.03, 1.05)	4.01E-08	0.07	1.04 (1.02, 1.07)	1.13E-03	-	-	-	0.22	1.06 (1.02, 1.09)	4.65E-04	-	-	-	0.06	1.02 (0.92, 1.13)	7.26E-01	0.11	0.78 (0.6, 1.01)	5.90E-02	0.09	1.03 (1.01, 1.05)	1.40E-03	0.1642	0.07	1 (0.95, 1.05)	9.19E-01
rs73047807	12	6704169	*CHD4*	Yes	G	C	1.03 (1.02, 1.04)	2.15E-08	0.15	1.03 (1.01, 1.05)	2.22E-03	-	-	-	0.05	1.05 (0.99, 1.11)	1.28E-01	-	-	-	0.07	1.03 (0.93, 1.14)	5.36E-01	0.02	0.51 (0.29, 0.9)	2.08E-02	0.15	1.03 (1.02, 1.05)	6.89E-06	0.1765	-	-	-
rs17221259	12	14410485	-	No	T	C	1.03 (1.02, 1.04)	7.18E-09	0.8	1.03 (1.05, 1.02)	2.48E-05	0.8	1.04 (1.11, 0.96)	3.35E-01	0.96	1.01 (1.08, 0.95)	7.33E-01	0.96	1.15 (1.71, 0.78)	4.74E-01	0.88	1.03 (1.11, 0.95)	4.81E-01	0.77	1 (1.18, 0.84)	9.56E-01	0.8	1.03 (1.04, 1.01)	9.09E-05	0.9626	0.79	1.02 (0.99, 1.05)	1.89E-01
rs2287163	12	107349294	*C12orf23*	No	C	T	1.03 (1.02, 1.03)	4.29E-11	0.62	1.03 (1.05, 1.02)	2.03E-06	0.63	1 (1.06, 0.94)	9.73E-01	0.68	1.02 (1.05, 1)	1.06E-01	-	-	-	0.51	1.01 (1.06, 0.97)	5.87E-01	0.65	1.07 (1.25, 0.92)	3.79E-01	0.63	1.02 (1.03, 1.01)	9.51E-06	0.8101	0.61	1.04 (1.01, 1.06)	3.96E-03
rs653178	12	112007756	*ATXN2*	No	C	T	1.03 (1.02, 1.04)	2.03E-15	0.49	1.03 (1.04, 1.01)	6.31E-05	-	-	-	0.08	1 (1.04, 0.95)	8.93E-01	-	-	-	0.29	0.98 (1.03, 0.93)	4.66E-01	0.03	1.43 (2.3, 0.88)	1.45E-01	0.45	1.04 (1.05, 1.03)	1.10E-13	0.0225	-	-	-
rs35444	12	115552437	-	No	A	G	1.03 (1.02, 1.04)	5.92E-13	0.61	1.02 (1.04, 1.01)	1.58E-04	0.62	1.02 (1.08, 0.96)	6.04E-01	0.59	1.03 (1.05, 1)	4.49E-02	0.61	0.98 (1.13, 0.84)	7.61E-01	0.66	1.03 (1.09, 0.98)	1.97E-01	0.7	1 (1.17, 0.85)	9.60E-01	0.63	1.03 (1.04, 1.02)	9.49E-09	0.9759	0.74	1.08 (1.05, 1.11)	9.05E-09
rs2292138	12	123339712	*HIP1R*	No	A	G	1.03 (1.02, 1.04)	1.97E-08	0.12	1.04 (1.06, 1.02)	3.43E-04	-	-	-	0.24	1.01 (1.04, 0.98)	6.64E-01	-	-	-	0.1	1.06 (1.15, 0.97)	2.13E-01	0.25	1.03 (1.23, 0.86)	7.34E-01	0.17	1.03 (1.05, 1.02)	6.95E-06	0.5191	0.22	1.01 (0.98, 1.04)	4.85E-01
rs10846744	12	125312425	*SCARB1*	No	C	G	1.03 (1.02, 1.04)	1.53E-11	0.15	1.03 (1.02, 1.05)	1.55E-04	0.15	1.11 (1.03, 1.2)	7.89E-03	0.63	1.04 (1.02, 1.07)	2.10E-03	-	-	-	0.3	0.98 (0.93, 1.03)	4.60E-01	0.5	1.05 (0.9, 1.23)	5.49E-01	0.29	1.03 (1.02, 1.04)	2.75E-06	0.1705	0.67	1 (0.98, 1.03)	7.69E-01
rs9515203	13	111049623	*COL4A2*	No	T	C	1.03 (1.02, 1.04)	5.66E-10	0.74	1.02 (1.04, 1.01)	1.97E-03	-	-	-	0.73	1.03 (1.06, 1)	4.64E-02	-	-	-	0.7	1.03 (1.09, 0.98)	2.56E-01	0.91	1.14 (1.5, 0.87)	3.32E-01	0.75	1.03 (1.04, 1.02)	9.19E-07	0.8931	0.85	1.05 (1.02, 1.09)	2.99E-03
rs35336096	14	33308791	*AKAP6*	No	T	G	1.03 (1.02, 1.03)	1.22E-10	0.55	1.03 (1.02, 1.05)	2.91E-07	-	-	-	0.24	1 (0.97, 1.04)	8.01E-01	-	-	-	0.48	1.01 (0.96, 1.07)	6.12E-01	0.55	0.99 (0.84, 1.17)	9.13E-01	0.54	1.02 (1.01, 1.03)	1.66E-05	0.4171	0.43	0.98 (0.95, 1)	6.89E-02
rs1890942	14	69765644	*GALNT16*	No	G	A	1.03 (1.02, 1.04)	5.38E-11	0.62	1.03 (1.02, 1.04)	2.91E-05	-	-	-	0.87	1.02 (0.98, 1.06)	3.15E-01	-	-	-	0.79	1.04 (0.98, 1.11)	1.77E-01	0.96	0.74 (0.45, 1.2)	2.17E-01	0.62	1.03 (1.02, 1.04)	1.39E-06	0.7070	-	-	-
rs11636009	15	36381553	-	No	C	T	1.02 (1.01, 1.03)	1.88E-08	0.44	1.02 (1.01, 1.03)	1.08E-03	0.46	1.02 (0.96, 1.08)	5.34E-01	0.21	1.01 (0.98, 1.04)	6.34E-01	0.21	0.88 (0.73, 1.05)	1.61E-01	0.54	1.07 (1.02, 1.12)	6.48E-03	0.71	1.11 (0.93, 1.31)	2.52E-01	0.48	1.02 (1.01, 1.03)	2.84E-05	0.2584	0.8	1.01 (0.98, 1.04)	4.51E-01
rs34591043	15	77713586	*HMG20A*	No	G	A	1.02 (1.02, 1.03)	9.59E-09	0.69	1.02 (1, 1.03)	9.72E-03	0.72	1.09 (1.02, 1.16)	8.55E-03	0.52	1.04 (1.01, 1.07)	2.65E-03	0.53	1.11 (0.96, 1.28)	1.43E-01	0.63	1.08 (1.02, 1.14)	4.69E-03	0.43	0.91 (0.77, 1.06)	2.16E-01	0.64	1.02 (1.01, 1.03)	3.90E-04	0.0329	0.4	1.01 (0.99, 1.04)	2.51E-01
rs17484235	15	78761414	*IREB2*	No	G	C	1.03 (1.02, 1.03)	1.13E-08	0.33	1.03 (1.01, 1.04)	1.28E-04	-	-	-	0.06	0.97 (0.91, 1.02)	2.39E-01	-	-	-	0.2	1.06 (1, 1.13)	6.22E-02	0.06	1.14 (0.8, 1.63)	4.54E-01	0.32	1.03 (1.01, 1.04)	2.08E-05	0.2187	0.03	1.02 (0.95, 1.09)	6.11E-01
rs7174250	15	81018587	*ABHD17C*	No	T	C	1.03 (1.02, 1.04)	8.36E-13	0.46	1.03 (1.01, 1.04)	3.14E-05	-	-	-	0.12	1.03 (0.99, 1.07)	1.21E-01	-	-	-	0.3	1.11 (1.05, 1.17)	1.32E-04	0.07	1.27 (0.94, 1.72)	1.20E-01	0.43	1.03 (1.02, 1.04)	9.03E-07	0.0447	0.07	1.06 (1.01, 1.11)	1.76E-02
rs12906348	15	85329421	*ZNF592*	No	C	A	1.03 (1.02, 1.04)	7.40E-11	0.27	1.02 (1.01, 1.04)	9.48E-04	-	-	-	0.06	1.06 (1, 1.11)	4.35E-02	-	-	-	0.18	1.05 (0.99, 1.12)	1.03E-01	0.1	1.01 (0.77, 1.31)	9.60E-01	0.25	1.03 (1.02, 1.04)	2.17E-07	0.7208	0.1	1.02 (0.98, 1.06)	2.96E-01
rs2343606	16	24793741	*TNRC6A*	No	C	T	1.03 (1.02, 1.03)	3.88E-09	0.73	1.03 (1.05, 1.02)	1.00E-05	0.74	1.01 (1.08, 0.95)	6.80E-01	0.72	1.02 (1.04, 0.99)	2.92E-01	-	-	-	0.77	1 (1.06, 0.95)	8.70E-01	0.86	1.05 (1.32, 0.83)	7.12E-01	0.75	1.02 (1.04, 1.01)	6.85E-05	0.8380	0.92	1.01 (0.97, 1.05)	5.97E-01
rs62048402	16	53803223	*FTO*	No	A	G	1.06 (1.05, 1.07)	1.20E-47	0.4	1.07 (1.05, 1.08)	8.48E-23	0.41	1.09 (1.03, 1.15)	4.05E-03	0.1	1.06 (1.02, 1.11)	3.93E-03	0.12	1.01 (0.8, 1.26)	9.54E-01	0.26	1.04 (0.98, 1.1)	1.96E-01	0.18	1.12 (0.92, 1.35)	2.49E-01	0.39	1.05 (1.04, 1.06)	2.15E-23	0.7007	0.2	1.05 (1.02, 1.08)	2.19E-03
rs1437134	16	69730426	*NFAT5*	No	G	A	1.03 (1.02, 1.04)	5.17E-13	0.58	1.04 (1.02, 1.05)	1.92E-08	0.59	1.02 (0.96, 1.08)	5.22E-01	0.77	1.01 (0.98, 1.04)	6.77E-01	0.78	1.06 (0.89, 1.26)	5.16E-01	0.65	1.05 (1, 1.11)	6.05E-02	0.82	1.18 (0.96, 1.44)	1.13E-01	0.6	1.02 (1.01, 1.03)	5.60E-06	0.3294	0.86	1 (0.97, 1.04)	8.61E-01
rs67329386	16	73048367	*ZFHX3*	No	T	C	1.03 (1.02, 1.04)	5.09E-12	0.16	1.04 (1.02, 1.06)	3.08E-06	0.17	1.01 (0.93, 1.09)	8.34E-01	0.26	0.98 (0.96, 1.01)	2.65E-01	0.27	0.99 (0.84, 1.16)	8.93E-01	0.24	1.07 (1.01, 1.14)	2.08E-02	0.24	1.04 (0.87, 1.25)	6.60E-01	0.22	1.04 (1.02, 1.05)	1.01E-08	0.0277	0.35	1.07 (1.04, 1.1)	4.93E-08
rs11648176	16	75456423	*CFDP1; RP11-77K12.1*	No	G	C	1.02 (1.02, 1.03)	1.85E-09	0.58	1.02 (1.01, 1.03)	5.38E-03	-	-	-	0.33	1.04 (1.01, 1.07)	2.56E-03	-	-	-	0.63	0.99 (0.94, 1.04)	7.88E-01	0.58	0.96 (0.82, 1.12)	5.98E-01	0.57	1.03 (1.01, 1.04)	1.13E-06	0.3455	0.53	1.02 (1, 1.05)	6.67E-02
rs62043959	16	81525204	*CMIP*	No	A	C	1.03 (1.02, 1.03)	3.96E-08	0.73	1.03 (1.05, 1.02)	4.26E-05	-	-	-	0.93	0.98 (1.03, 0.93)	4.07E-01	-	-	-	0.65	1 (1.06, 0.95)	9.01E-01	0.93	1.12 (1.57, 0.8)	5.03E-01	0.75	1.03 (1.04, 1.01)	3.10E-05	0.3132	0.91	0.98 (0.94, 1.03)	4.50E-01
rs75543711	17	1256074	*YWHAE*	No	G	A	1.04 (1.03, 1.05)	1.86E-09	0.92	1.03 (1.06, 1.01)	4.48E-03	-	-	-	0.97	1.05 (1.12, 0.97)	2.16E-01	-	-	-	0.77	1.01 (1.07, 0.95)	6.85E-01	0.83	1.02 (1.24, 0.84)	8.68E-01	0.86	1.04 (1.06, 1.03)	1.37E-07	0.8522	0.7	1.02 (1, 1.05)	7.60E-02
rs216193	17	2203453	*SMG6*	No	A	G	1.03 (1.02, 1.04)	2.82E-17	0.38	1.03 (1.05, 1.02)	1.90E-07	0.38	1.02 (1.08, 0.96)	5.33E-01	0.6	1.05 (1.07, 1.02)	6.20E-04	0.61	1.06 (1.22, 0.92)	4.38E-01	0.38	0.97 (1.02, 0.92)	1.94E-01	0.38	1.01 (1.17, 0.86)	9.42E-01	0.38	1.03 (1.04, 1.02)	6.73E-10	0.2335	0.24	1.04 (1.01, 1.07)	1.01E-02
rs72827590	17	7498271	*FXR2*	No	G	A	1.04 (1.02, 1.05)	3.08E-08	0.11	1.05 (1.03, 1.07)	1.61E-06	0.12	1.12 (1.03, 1.22)	7.67E-03	0.03	1.02 (0.94, 1.1)	6.84E-01	0.03	1.27 (0.87, 1.85)	2.23E-01	0.07	1.07 (0.97, 1.17)	1.77E-01	0.06	0.93 (0.67, 1.27)	6.36E-01	0.11	1.02 (1, 1.04)	1.70E-02	0.1872	0.11	1.01 (0.98, 1.05)	4.66E-01
rs2535599	17	15828161	*ADORA2B*	No	A	G	1.03 (1.02, 1.04)	1.76E-08	0.21	1.02 (1, 1.03)	2.07E-02	0.21	1.06 (0.99, 1.14)	7.97E-02	0.59	1.02 (1, 1.05)	7.11E-02	-	-	-	0.26	1.05 (0.99, 1.11)	8.36E-02	0.23	1.03 (0.86, 1.22)	7.59E-01	0.23	1.03 (1.02, 1.04)	7.73E-06	0.7169	0.16	0.98 (0.95, 1.01)	2.70E-01
rs2233072	17	19281828	*MAPK7*	No	T	G	1.03 (1.02, 1.04)	1.18E-08	0.23	1.02 (1.04, 1.01)	5.48E-03	-	-	-	0.84	1.04 (1.08, 1)	4.46E-02	-	-	-	0.49	1.07 (1.12, 1.01)	1.14E-02	0.93	1.08 (1.63, 0.71)	7.16E-01	0.25	1.03 (1.04, 1.01)	2.82E-05	0.5664	-	-	-
rs2517960	17	37846521	*PGAP3; ERBB2*	No	T	C	1.03 (1.02, 1.04)	4.57E-12	0.36	1.04 (1.05, 1.03)	1.53E-08	0.36	0.99 (1.05, 0.93)	7.87E-01	0.77	1.06 (1.09, 1.02)	5.36E-04	-	-	-	0.46	1.03 (1.08, 0.98)	3.04E-01	0.59	1.02 (1.19, 0.87)	7.96E-01	0.38	1.02 (1.03, 1.01)	4.48E-04	0.0817	0.5	1.03 (1, 1.05)	1.97E-02
rs34710835	17	45146717	*RP11-156P1.2*	No	C	T	1.02 (1.02, 1.03)	4.01E-09	0.39	1.03 (1.04, 1.01)	3.38E-05	0.38	1 (1.06, 0.94)	9.91E-01	0.6	1.04 (1.06, 1.01)	8.46E-03	-	-	-	0.48	1.03 (1.08, 0.98)	2.27E-01	0.67	1.05 (1.25, 0.89)	5.35E-01	0.44	1.02 (1.03, 1.01)	5.99E-04	0.6983	0.83	1 (0.96, 1.03)	7.68E-01
rs11079849	17	47090785	*IGF2BP1*	No	C	T	1.03 (1.02, 1.04)	2.13E-11	0.69	1.03 (1.05, 1.02)	1.08E-05	-	-	-	0.81	1.03 (1.06, 0.99)	1.15E-01	-	-	-	0.72	1.02 (1.08, 0.97)	3.94E-01	0.95	0.95 (1.41, 0.64)	8.02E-01	0.69	1.03 (1.04, 1.02)	2.00E-06	0.9838	0.98	1.05 (0.97, 1.14)	2.51E-01
rs12940636	17	53400110	*HLF*	No	T	C	1.02 (1.02, 1.03)	1.15E-08	0.67	1.02 (1.03, 1)	1.27E-02	0.66	1.06 (1.12, 0.99)	7.82E-02	0.81	1.01 (1.05, 0.98)	5.34E-01	0.82	1.1 (1.32, 0.91)	3.15E-01	0.76	1.02 (1.08, 0.96)	5.12E-01	0.79	1.08 (1.32, 0.89)	4.47E-01	0.65	1.03 (1.04, 1.02)	8.95E-07	0.7641	0.64	1.04 (1.01, 1.06)	3.14E-03
rs2680703	17	56429673	*SUPT4H1*	Yes	G	A	1.02 (1.02, 1.03)	2.41E-08	0.47	1.02 (1.01, 1.03)	1.64E-03	-	-	-	0.38	1.02 (1, 1.05)	7.23E-02	-	-	-	0.38	1.02 (0.97, 1.07)	4.63E-01	0.13	0.74 (0.57, 0.97)	2.62E-02	0.35	1.03 (1.02, 1.04)	1.25E-05	0.1621	-	-	-
rs9912468	17	64318357	*PRKCA*	No	G	C	1.02 (1.01, 1.03)	4.61E-08	0.42	1.03 (1.04, 1.02)	1.60E-05	0.43	1.05 (1.11, 0.99)	8.33E-02	0.37	1.02 (1.04, 0.99)	2.23E-01	0.37	0.87 (1.01, 0.75)	6.26E-02	0.44	1.01 (1.06, 0.96)	7.33E-01	0.48	0.97 (1.12, 0.83)	6.45E-01	0.42	1.02 (1.03, 1.01)	8.31E-04	0.2224	0.41	1 (0.98, 1.03)	8.42E-01
rs145606454	17	65893289	*BPTF*	No	A	AC	1.05 (1.04, 1.06)	5.28E-13	0.21	1.05 (1.04, 1.07)	5.72E-11	0.21	1.06 (0.99, 1.14)	8.74E-02	0.22	1.05 (1.01, 1.08)	1.17E-02	-	-	-	0.27	1.04 (0.98, 1.1)	1.70E-01	0.62	0.92 (0.78, 1.08)	3.04E-01	-	-	-	0.5663	0.68	1 (0.97, 1.02)	7.16E-01
rs2306527	17	76798155	*USP36*	No	C	T	1.03 (1.02, 1.04)	2.61E-15	0.48	1.02 (1.04, 1.01)	2.88E-04	0.48	0.99 (1.05, 0.93)	6.60E-01	0.7	1.05 (1.08, 1.02)	4.78E-04	-	-	-	0.56	1.04 (1.09, 0.99)	1.24E-01	0.6	0.98 (1.15, 0.84)	8.41E-01	0.46	1.04 (1.05, 1.02)	1.12E-10	0.2598	0.65	0.99 (0.97, 1.02)	4.41E-01
rs1371274	18	1837007	-	No	C	A	1.03 (1.02, 1.04)	1.32E-08	0.14	1.04 (1.02, 1.06)	1.22E-04	-	-	-	0.21	1.02 (0.99, 1.05)	2.25E-01	-	-	-	0.12	1.05 (0.97, 1.13)	2.62E-01	0.02	1.33 (0.73, 2.4)	3.48E-01	0.15	1.03 (1.02, 1.05)	7.09E-05	0.8065	-	-	-
rs2472610	18	21138865	*NPC1*	No	T	C	1.03 (1.02, 1.04)	5.93E-12	0.35	1.02 (1.04, 1.01)	2.86E-04	0.35	1.05 (1.12, 0.99)	1.04E-01	0.15	1.02 (1.06, 0.98)	4.09E-01	-	-	-	0.24	1.05 (1.11, 0.99)	8.71E-02	0.14	1.21 (1.54, 0.95)	1.29E-01	0.34	1.03 (1.04, 1.02)	4.92E-08	0.6132	0.05	1.07 (1.01, 1.13)	2.82E-02
rs12607512	18	31259579	*ASXL3*	No	G	A	1.02 (1.02, 1.03)	6.20E-10	0.44	1.02 (1, 1.03)	9.72E-03	0.44	1.03 (0.97, 1.09)	3.14E-01	0.35	1.02 (0.99, 1.05)	1.43E-01	-	-	-	0.55	1.03 (0.98, 1.08)	2.59E-01	0.75	1.08 (0.9, 1.29)	4.31E-01	0.49	1.03 (1.02, 1.04)	7.92E-08	0.8372	0.78	1.03 (1, 1.06)	4.09E-02
rs60951582	18	36553476	-	No	A	C	1.04 (1.03, 1.05)	1.14E-09	0.9	1.05 (1.07, 1.03)	2.93E-06	0.9	1.04 (1.15, 0.95)	3.98E-01	0.91	1.06 (1.11, 1.01)	2.17E-02	-	-	-	0.93	1.12 (1.23, 1.02)	2.10E-02	0.88	1.05 (1.32, 0.84)	6.58E-01	0.87	1.03 (1.04, 1.01)	1.24E-03	0.2389	0.75	1.02 (0.99, 1.05)	1.49E-01
rs11664369	18	57739072	-	No	T	C	1.03 (1.02, 1.04)	1.08E-10	0.26	1.02 (1.01, 1.04)	2.01E-03	0.27	0.96 (0.9, 1.03)	2.63E-01	0.25	1.03 (1, 1.07)	4.19E-02	0.26	0.97 (0.82, 1.14)	7.18E-01	0.16	1.04 (0.97, 1.11)	2.34E-01	0.17	1.2 (0.97, 1.48)	9.53E-02	0.24	1.03 (1.02, 1.04)	5.56E-08	0.2853	0.17	1.05 (1.01, 1.08)	4.34E-03
rs56289821	19	11188247	*LDLR*	No	G	A	1.04 (1.02, 1.05)	1.17E-08	0.88	1.04 (1.06, 1.02)	1.55E-04	0.88	1.06 (1.15, 0.97)	2.22E-01	0.92	1.05 (1.1, 1)	3.47E-02	0.92	1.18 (1.55, 0.9)	2.42E-01	0.91	1.01 (1.1, 0.93)	8.40E-01	0.98	1 (1.75, 0.58)	9.88E-01	0.89	1.03 (1.05, 1.01)	3.23E-04	0.9133	-	-	-
rs4808152	19	18826052	*CRTC1*	No	A	G	1.03 (1.02, 1.04)	1.60E-12	0.53	1.03 (1.05, 1.02)	2.40E-07	-	-	-	0.22	1.03 (1.06, 0.99)	1.12E-01	-	-	-	0.42	0.98 (1.03, 0.94)	4.73E-01	0.13	0.95 (1.21, 0.74)	6.72E-01	0.48	1.03 (1.04, 1.02)	6.82E-07	0.3393	0.08	1.04 (0.99, 1.09)	9.20E-02
rs10520390	19	46327831	*SYMPK*	No	C	G	1.06 (1.04, 1.08)	9.52E-09	0.95	1.05 (1.09, 1.02)	7.36E-04	-	-	-	-	-	-	-	-	-	0.97	1.1 (1.28, 0.94)	2.28E-01	-	-	-	0.94	1.06 (1.08, 1.03)	6.00E-06	0.8645	-	-	-
rs56968346	21	30540165	*MAP3K7CL*	No	T	C	1.04 (1.03, 1.05)	8.59E-15	0.15	1.05 (1.03, 1.07)	1.57E-07	0.16	1.03 (0.95, 1.11)	5.20E-01	0.41	1.04 (1.01, 1.07)	5.75E-03	-	-	-	0.2	1.05 (0.99, 1.12)	8.42E-02	0.23	1.09 (0.91, 1.32)	3.56E-01	0.17	1.03 (1.02, 1.05)	1.54E-06	0.8635	0.1	1.01 (0.97, 1.05)	6.18E-01
rs12482462	21	42626851	*BACE2*	No	T	C	1.02 (1.01, 1.03)	3.19E-08	0.41	1.03 (1.02, 1.05)	2.15E-06	-	-	-	0.15	1.01 (0.97, 1.05)	7.36E-01	-	-	-	0.52	1.02 (0.97, 1.07)	5.19E-01	0.64	0.92 (0.78, 1.09)	3.53E-01	0.44	1.02 (1.01, 1.03)	6.21E-04	0.3085	0.73	1.03 (1.01, 1.06)	1.41E-02
rs5760061	22	24178279	*DERL3*	No	A	G	1.03 (1.02, 1.04)	1.43E-10	0.79	1.02 (1.01, 1.04)	3.55E-03	0.81	1 (0.93, 1.08)	9.47E-01	0.46	1.03 (1.01, 1.06)	1.75E-02	0.49	1.05 (0.91, 1.21)	5.13E-01	0.72	1.06 (1, 1.12)	4.45E-02	0.6	1.12 (0.95, 1.31)	1.73E-01	0.74	1.03 (1.02, 1.04)	1.09E-06	0.8125	0.61	1.01 (0.99, 1.03)	4.13E-01

Chr.: Chromosome; EA: Effect Allele; NEA: Non-effect Allele; EAF: Effect Allele Frequency; OR: Odds Ratio; CI: Confidence Interval.

*A total of 12 novel loci as compared to previous all-cause HF GWAS (PMID: 31919418, 36376295, 36517512, 37429843, 37503172, 40038546), defined as non-overlapping physical distance of > 500kb on both sides of the variant.

**Heterogeneity P-values from Cochran's Q test.

10. Study Limitations

As the MVP cohort primarily consists of male participants, sex-stratified analyses may be underpowered. Please include in the main text any limitations arising from the demographic composition of the study population.

We thank the reviewer for highlighting this important point. We have now explicitly acknowledged this in the Discussion and clarified that our study is likely underpowered to detect female-specific loci or to comprehensively characterize sex differences in genetic risk for HF:

"The MVP cohort is predominantly composed of male veterans, which limits the statistical power for sex-stratified analyses, particularly among women, and may constrain the generalizability of our findings to female. Larger and more demographically diverse cohorts, including those with a higher proportion of women, will be needed to fully characterize sex-specific genetic architecture and to confirm the applicability of these findings across broader clinical populations."

Reviewer 2:

The authors present genome wide association studies of heart failure with reduce and preserved ejection fraction, combining data from the Million Veteran Program and BioVU, for European, African, Hispanic, and Asian ancestries. This is an important increment in sample size for GWAS these disorders and addresses non-European ancestry groups. 9 novel genomic loci for HFrEF, 1 for HFpEF in a pan- ancestry analysis, and 12 for all-cause HF are reported. The lack of loci for HFpEF, despite an increase in sample size, suggest very low heritability and it would be interesting to report this.

A series of analyses are performed to explore the GWAS findings which are presented in a somewhat counter-intuitive sequence. Some of the claims made in the declarative subtitles are not fully supported by the results presented, and in some cases the results presented to not relate to the subtitle. Generally, the reporting could be clearer by including sufficient information, for example 'gene-based analysis' is report in the results section which appears to represent findings from MAGMA, but this only clear when reading with the methods. The approaches to prioritise genes at the identified loci could be updated to use modern approaches. The downstream analyses could be improved with more precise reporting and a logical structure and flow.

While the authors report a two-fold increase in sample size for HF subtypes, there is a modest yield in genetic discovery. Importantly they do address previously under-represented ancestry groups. The approach to case identification and GWAS appear robust, however, the manuscript would benefit from revision to improve the precision and completeness of reporting, to explain to the reader the significance (or lack thereof) of the findings, and to ensure that sufficient information is provided to interpret the results. The tables and figures could also be improved.

Specific comments

• The type of analysis for which results reported are not always clear in abstract

We thank the reviewer for this helpful comment. We have revised the abstract to more clearly indicate the type of analysis underlying each set of results. Specifically, we now state that we performed random-effects meta-analyses of multi-ancestry GWAS for HFrEF and HFpEF in the Million Veteran Program and BioVU, and a separate meta-analysis for all-cause HF across MVP, BioVU, and published GWAS. We also briefly summarize the downstream gene-based tests, tissue-enrichment, transcriptome-wide association, and fine-mapping analyses that support the implicated pathways and candidate causal genes. This revised abstract (below) provides the maximum level of methodological detail possible while adhering to the journal's 150-word limit:

Revised Abstract

Heart failure (HF) affects 6.7 million people in the US and includes two major subtypes, HF with reduced ejection fraction (HFrEF) and HF with preserved ejection fraction (HFpEF), with distinct genetic architectures. We meta-analyzed genome-wide association studies (GWAS) of 38,781 HFrEF cases, 38,163 HFpEF cases, and 526,135 controls across European, African, Hispanic, and Asian ancestries using the Million Veteran Program and Vanderbilt University DNA Databank (BioVU). We identified 46 genome-wide significant loci for HFrEF (9 novel) and 3 loci for HFpEF (1 novel). Four HFrEF loci were detected in African ancestry participants (near CD36, SPI1, TRIM48, and SPNS3), with low risk-allele frequencies in European reference populations. In the all-cause HF meta-analysis (200,070 cases, 2,076,466 controls), we identified 136 loci (12 novel). Gene-based tests, tissue enrichment, transcriptome-wide association, and fine-mapping implicated vascular, metabolic, and TGF-β/Smad signaling pathways and nominated candidate causal genes, clarifying shared and subtype-specific risk across ancestries.

• Listing genes by nearest versus cytogenetic location not consistent

We thank the reviewer for pointing out this inconsistency. In the original manuscript, loci were labeled by nearest gene, whereas some were referred to by cytogenetic location. This arose because a SNP was mapped to the nearest gene only if it lies within a ±10 kb window around the gene body; loci for which the lead SNP fell outside this window were not assigned a gene name and were therefore labeled by cytogenetic band. We have now clarified this in the Methods:

"Genome-wide significant loci were mapped the nearest protein-coding gene which was located within ±10 kb region. If the significant SNP was >10 kb from any annotated protein-coding gene, the locus was labeled by its cytogenetic band."

• "Genetic factors contribute to HF risk, supported by an estimated heritability of ~ 25% for both all-cause HF and its subtypes.8" is not correct, the cited study did not include HF subtypes

We thank the reviewer for this careful clarification. We have revised the sentence in the Introduction to avoid overextending this result to HFrEF and HFpEF. We additionally added a citation of our previous estimation of HFrEF and HFpEF heritability (PMID: 36517512). The revised text now reads:

"Genetic factors contribute to HF risk, supported by an estimated heritability of ~ 26% for all-cause HF (PMID: 31919418), 25% for HFrEF and 22% for HFpEF (PMID: 36517512)."

• The h2SNP of the traits should be reported.

We thank the reviewer for this suggestion. We estimated SNP-based heritability using GREML-LDMS in the European and African groups and have added these results to the revised manuscript. We also attempted the same analysis in the Hispanic American group. However, the GREML procedure did not converge, likely reflecting genetic heterogeneity for stable variance-component estimation in this population group. We therefore did not report the heritability

estimates of HF and subtypes in Hispanic Americans. We have added the following statements to methods and results:

Methods:

"We estimated SNP-based heritability (h^2) for HF and subtypes separately in European and African participants using GREML-LDMS in GCTA (PMID: 21167468, 26323059). Variants were stratified into minor allele frequency (MAF) and linkage disequilibrium (LD) bins, and genetic relationship matrices (GRMs) were constructed for each bin. We then fit a multi-GRM REML model including age, sex, and top 10 PCs to estimate the h^2."

Results

"Among individuals of European ancestry, the estimated SNP-based heritability h^2 was 0.31 for HFrEF, 0.26 for HFpEF, and 0.31 for all-cause HF. Among individuals of African ancestry, the corresponding estimates were 0.29 for HFrEF, 0.36 for HFpEF, and 0.19 for all-cause HF."

• Report heterogeneity by ancestry in the pan-ancestry analysis

We have added the heterogeneity test p-values for the multi-ancestry meta-analyses to the revised Tables S2-S3. Specifically, for each lead SNP we now report the p-value from Cochran's Q test for heterogeneity across studies and ancestries, as implemented in the GWAMA random-effects meta-analysis framework. These heterogeneity statistics are derived from the same random-effects models described in the Methods. Please see our response to Reviewer #1, Point 9, for the updated tables.

• No use of power adjusted replication framework when evaluating non-Eur ancestries

We appreciate this important point. We agree that differences in sample size and allele frequency between European and non-European ancestry cohorts may affect statistical power. In the current analysis, we evaluated external replication in a non-European population (Biobank Japan; response to Reviewer #1, Point 9) and assessed heterogeneity of loci across ancestries using random-effects meta-analysis and Cochran's Q statistics (see response to the previous comment), to facilitate interpretation of generalizability to non-European populations. We further demonstrated consistency of effect sizes across ancestries (Figure 2), such that, despite differences in statistical power, the directions and magnitudes of effects were similar. We now explicitly acknowledge the absence of a formal power-adjusted replication framework as a limitation in the Discussion (below), noting that some loci with non-significant or attenuated effects in non-European ancestries may simply be underpowered rather than truly absent or ancestry-specific. Future work with larger non-European HF cohorts will be better positioned to more rigorously quantify cross-ancestry transferability of HF loci.

"Although we compared effect estimates across ancestries and reported heterogeneity statistics, we did not apply a formal power-adjusted framework for non-European populations. Therefore,

some loci that appear non-significant or attenuated in non-European ancestries may simply reflect limited statistical power rather than true ancestry-specific absence of effect."

• Section on genetic architecture describes locus discovery

We thank the reviewer for this comment. We understand that "genetic architecture" is a broader concept than simple locus discovery. To improve clarity, we have moved the two sections "Different genetic architecture across ancestries" and "Different genetic architecture across HFrEF and HFpEF" that mainly discussed the locus discovery and comparison to the section titled "Identification of genome-wide significant (GWS) loci". Further, we have added a separate section titled "Heritability and genetic correlations", as shown below:
Heritability and genetic correlations

"Among individuals of European ancestry, the estimated SNP-based heritability h^2 was 0.31 for HFrEF, 0.26 for HFpEF, and 0.31 for all-cause HF. Among individuals of African ancestry, the corresponding estimates were 0.29 for HFrEF, 0.36 for HFpEF, and 0.19 for all-cause HF. Genetic correlations were estimated between HF subtypes and all-cause HF, with 0.55 (standard error [SE] 0.05, $p < 0.0001$) between HFrEF and HFpEF, 0.92 (SE 0.02, $p < 0.0001$) and 0.83 (SE 0.02, $p < 0.0001$) between all-cause HF and HFrEF, and all-cause HF and HFpEF, respectively."

• The rational for pleiotropy scan of loci identified in individuals of African ancestry is not well outlined, and the analysis is limited.

We thank the reviewer for this helpful comment. The "pleiotropy scan" refers to our phenome-wide association study (PheWAS) of HF risk loci in individuals of African ancestry. Our primary rationale was to evaluate whether the African-enriched loci (*CD36*, *SPI1*, *TRIM48*, and *SPNS3*), which are rare in European populations, are linked to a broader spectrum of HF-related traits such as cardiomyopathy, hypertensive heart disease, and arrhythmias. We have now clarified this rationale in the Methods (below). We also added PheWAS Manhattan plots as requested by Reviewer #1, Comment #3 (above). We agree that this analysis is exploratory and limited by sample size, phenotype definitions, and restriction to the African-ancestry subset in MVP. We now explicitly describe the PheWAS as a hypothesis-generating pleiotropy scan.

Methods

"To explore pleiotropy and provide additional biological context for African-specific HF loci *CD36*, *SPI1*, *TRIM48*, and *SPNS3*, we conducted an exploratory, hypothesis-generating PheWAS of African-ancestry risk variants for HFrEF, HFpEF, and all-cause HF in the MVP African cohort. Phenotypes were derived from the VA electronic health record using the established MVP phenome framework, in which ICD-9 and ICD-10 diagnosis codes are aggregated into clinically meaningful phecodes by grouping related codes into disease categories (PMID: 33984066). Using this framework, we analyzed 1,709 phecode-based phenotypes (PMID: 33984066, 20335276). For each HF risk variant-phecode pair, we fit logistic regression models adjusting for

age, sex, and the top 5 principal components, and controlled for multiple testing using the false discovery rate (FDR, Benjamini and Hochberg, 1995), with $q < 0.05$ considered statistically significant."

• The approach to fine mapping and gene prioritisation could be improved to provide a more cohesive approach and to make use of modern tools, such as PoPs.

We thank the reviewer for this thoughtful suggestion and agree that modern integrative prioritization frameworks such as PoPS (PMID: 37443254) represent important advances in post-GWAS analysis. In the present study, our primary focus is on characterizing the genetic architecture of HF across multiple ancestries, with particular emphasis on non-European populations. Because PoPS currently rely heavily on LD and functional reference resources that are best characterized in European populations (PMID: 37443254), their direct application to our multi-ancestry and non-European analyses is not straightforward and may introduce ancestry-specific biases.

Instead, we implemented a multi-layered gene-prioritization strategy that combines Bayesian fine-mapping (credible sets and posterior inclusion probabilities), gene-based tests (MAGMA), TWAS with colocalization, pQTL annotation, tissue and pathway enrichment, and overlap with enhancer regions to highlight likely causal genes and pathways. Further, the fine mapping strategy MESuSiE (PMID: 38168930, published in 2024) is the most up to date fine mapping tool that is well suited in a multi-ancestry population, incorporating individual-level genomics data that accounts for the heterogeneous LD structure across ancestries. Therefore, we hope the reviewer agrees that this pipeline represents a modern and comprehensive gene-prioritization strategy that is well aligned with the goals and design of our multi-ancestry study. While additional tools such as PoPS may further complement these analyses in the future, particularly as non-European LD and functional reference resources continue to improve, we believe that our current pipeline provides a robust and cohesive framework for highlighting likely causal genes and pathways in HF.

• The main table would be better located in the supplement.

Thank you for this comment. We have moved the current Table 1 to Table S2 in the revised manuscript.

• The multi-lane circus plots contain different analysis findings and are difficult to follow. These figures could be labelled, improved for clarity.

Thank you for this comment. We have added labels to all tracks of the circular Manhattan plots as shown below:

Figure 1. Manhattan Plots for the Meta-analysis of GWAS on HFrEF and TWAS.

Track A: Manhattan Plot of the Multi-ancestry GWAS Meta-analysis on HFrEF. Track B: Manhattan Plot of the European-ancestry GWAS Meta-analysis on HFrEF. Track C: Manhattan

Plot of the African-ancestry GWAS Meta-analysis on HFrEF. Track D: Manhattan Plot of the TWAS on HFrEF in Cardiovascular Tissue Types. Track E: Manhattan Plot of the TWAS on HFrEF in Skeletal Muscle Tissue Type. The Genome-wide Significant Loci of HFrEF are Labeled, with Red Color Indicating African-Specific Genome-wide Significant Loci. Bold Font Indicates Significant Enrichment in Cardiovascular Tissue Types. Italic Font Indicates Significant Enrichment in Skeletal Muscle Tissue Type. Bold Italic Font Indicates Significant Enrichment in both Cardiovascular and Skeletal Muscle Tissue Types.

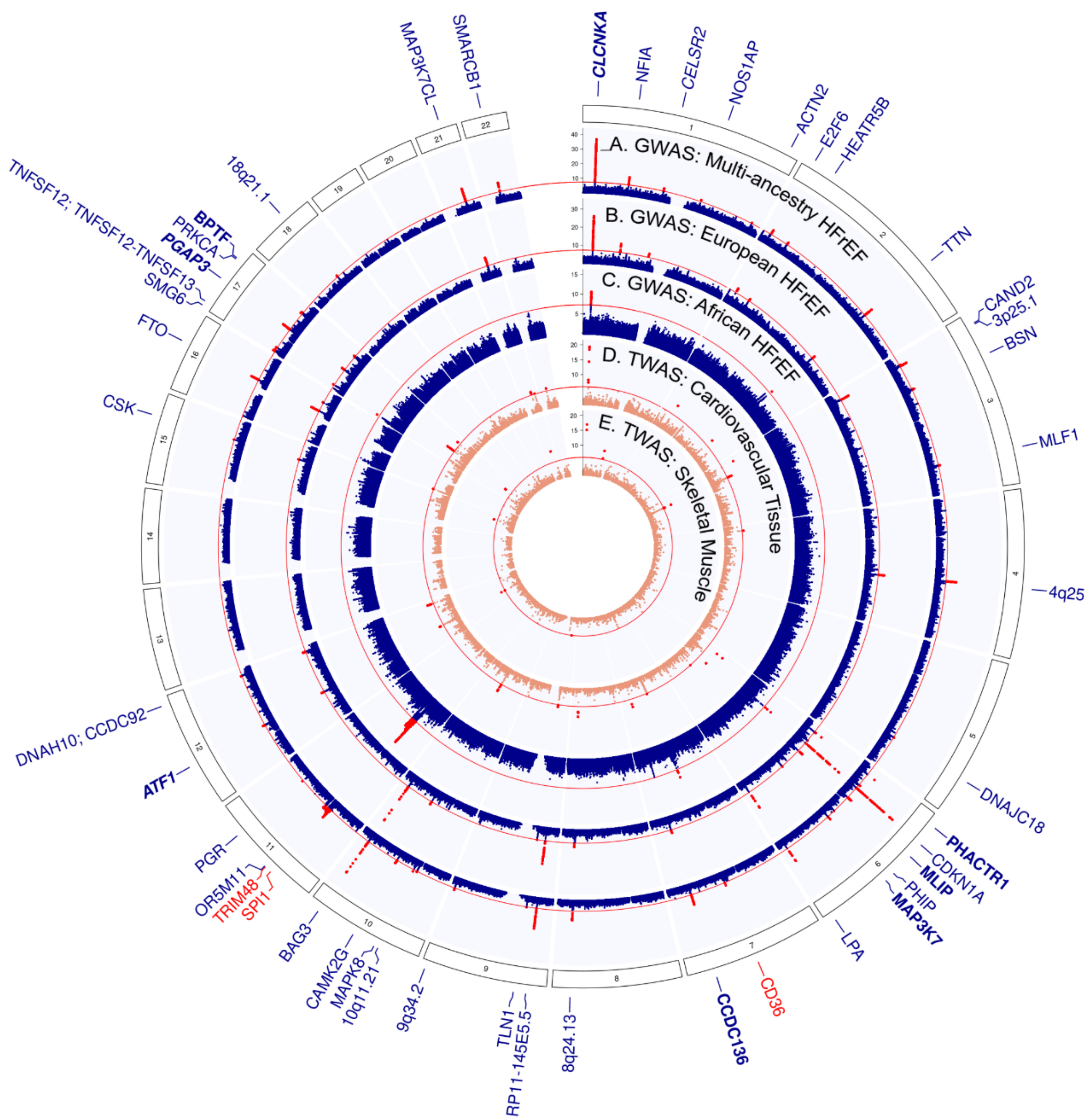

Figure S2. Manhattan Plots for the Meta-analysis of GWAS on HFpEF and TWAS.

Track A: Manhattan Plot of the Multi-ancestry GWAS Meta-analysis on HFpEF. Track B: Manhattan Plot of the European-ancestry GWAS Meta-analysis on HFrEF. Track C: Manhattan Plot of the African-ancestry GWAS Meta-analysis on HFpEF. Track D: Manhattan Plot of the

TWAS on HFpEF in Cardiovascular Tissue Types. Track E: Manhattan Plot of the TWAS on HFpEF in Skeletal Muscle Tissue Type. The Genome-wide Significant Loci of HFpEF are Labeled. Italic Font Indicates Significant Enrichment in Skeletal Muscle Tissue Type.

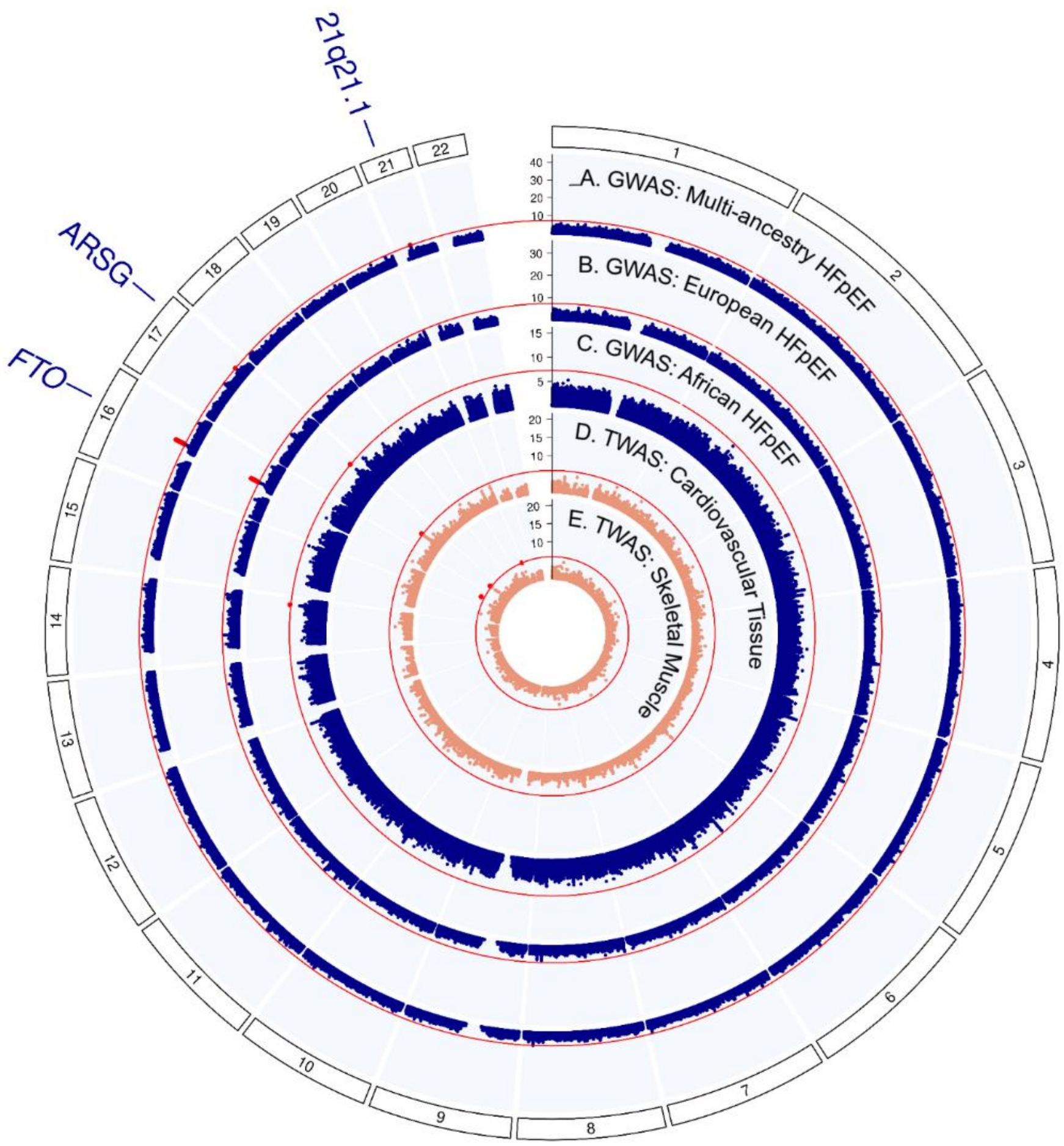

• Other figures are not referenced fully in the text – ie the ancestry heterogeneity plot (no figure number provide).

We included the figures referenced above. We confirmed that all other figures (Figures 2-4 in the revised manuscript) were referenced in the manuscript as follows:

"Comparing the estimates for the GWS loci across ancestral populations revealed mostly similar genetic effects, with some population-specific associations. Of the 29 HFrEF loci in the European ancestry, 7 loci showed small or null effects in African ancestry, **Table 2 & Figure 2**. Of the 7 HFrEF GWS loci in the African ancestry, 2 loci showed small or null effects in the European ancestry, and 4 loci (*CD36*, *SPI1*, *TRIM48*, and *SPNS3)* were rare in the European ancestry, with MAF ⩽ 0.05%, **Table 2 & Figures 2-3**…Genetic loci are mostly specific to HFrEF and HFpEF. Of the 46 multi-ancestry HFrEF loci, 42 showed heterogenous effects in HFpEF, **Table 3 & Figure 4**."

• Generally, the clarity and flow of the reporting could be improved, to explain rationale for the various analyses and the significance of the findings.

We thank the reviewer for this important comment. In revising the manuscript, we have made changes aimed specifically at improving clarity, flow, and explicit explanation of the rationale and significance of each analysis. First, we reorganized the Results to begin with "Identification of genome-wide significant (GWS) loci," external replication (Biobank Japan) and then move logically through cross-ancestry and subtype comparisons, heritability and genetic correlation ("Heritability and genetic correlations"), and functional follow-up. We also revised the abstract to more clearly specify the methodology. Second, we clarified the rationale in the Methods for key downstream analyses, including the African-ancestry PheWAS (explicitly described as an exploratory, hypothesis-generating pleiotropy scan), the Mendelian randomization analyses of HF risk factors, the Open Targets therapeutic tractability assessment for TWAS-prioritized genes, and the integration of Roadmap/ENCODE chromatin states for functional prioritization of loci. Third, we enhanced the visual presentation to make the findings easier to interpret, including adding a Venn diagram of loci shared across all-cause HF, HFrEF, and HFpEF; PheWAS Manhattan plots; a revised Figure 3 with separate panels for effect sizes and allele frequencies; and clearer labels on the circular Manhattan plots. Finally, we expanded the Discussion to better articulate the biological and clinical implications of key findings (e.g., MR results, ancestry-related differences, and functional/therapeutic interpretation). We hope the reviewer agrees with us that these changes have substantially improved the clarity and flow of the manuscript.

Reviewer #3 (Remarks to the Author):

Liu et al are reporting a GWAS for heart failure (HF) subtypes and all-cause HF in populations similar to genetic ancestries of multiple reference populations. Using standard downstream analyses for GWAS, they characterize HFrEF, HFpEF, and all-cause HF findings in populations with genetic ancestry similar to that of European and African reference populations. They report some novel findings which provide insights into disease etiology.

Major comments:

1) The use of "ancestry" and "ancestries" throughout the manuscript should conform to the NASEM report guidelines for referring to populations. Chapter 5, best Practice 6, page 125 of the report might be informative.

https://www.nationalacademies.org/our-work/use-of-race-ethnicity-and-ancestry-as-population-descriptors-in-genomics-research

Furthermore, the use of ancestry and population should be used more consistently and accurately. For example, "Population-specific" on line 90 would be better as ancestry-specific. On line 118, the genetic correlations weren't done among European ancestry, they were among the GWAS estimates from individuals with genetic ancestry similar to reference populations of European ancestry. On line 172, the findings are generalizable for populations or individuals of a given ancestry. In Table 2 these are loci from a sample of individuals with genetic ancestry matching African reference populations, not "African HFrEF loci"

We thank the reviewer for this important and constructive comment and for directing us to the NASEM report. In the revision, we have carefully reviewed the manuscript to align our language closely with these best-practice recommendations and to use "ancestry" and "population" more consistently and precisely. In the Million Veteran Program (MVP), participants are classified into groups such as EUR, AFR, HIS, and EAS using algorithms that integrate genetic data and self-identified race/ethnicity, and then relate individuals to 1000 Genomes reference superpopulations, as previously described (PMID: 31564439). We hope these changes address the reviewer's concerns and bring our use of ancestry/population terminology into closer alignment with the NASEM guidelines. Specifically:

- In line 90 and related context: we replaced "population-specific" with "ancestry-specific" where the context refers to genetic ancestry.
- In line 118 and related context: we clarified that genetic correlations were estimated using GWAS summary statistics from individuals with genetic ancestry similar to reference populations of European ancestry, rather than "among European ancestry" in a broad sense:
  "Further, genetic correlations were estimated between HFrEF, HFpEF and all-cause HF based on the meta-analysis summary statistics of from individuals with genetic ancestry similar to reference populations of European ancestry."
- In line 172 and related context, we revised statements about generalizability to explicitly refer to "individuals or populations with similar genetic ancestry" rather than implying generalizability to all "European" or "African" populations.
- In Table 2 and related text, we changed phrasing such as "African HFrEF loci" to "loci identified in individuals with African genetic ancestry" to emphasize that these loci were discovered in a sample whose inferred genetic ancestry matches African reference populations.
- In the Methods, we clarified our use of ancestry terminology by noting that "European," "African," "Hispanic," and "Asian" refer to genetically inferred ancestry groups defined using the Harmonizing Genetic Ancestry and Self-identified Race/Ethnicity (HARE) approach, rather than social or geographic populations:
  "Individual ancestries for European, African, Hispanic and Asian were estimated and defined using the algorithm of Harmonizing Genetic Ancestry and Self-identified Race/Ethnicity (HARE), which incorporated both self-reported race/ethnicity and genetic profile and successfully assigned ancestries for > 98% participants to the following four non-overlapping ancestry groups: non-Hispanic white (European), non-Hispanic Black (African), Hispanic, and non-Hispanic Asian participants. We therefore use the terms "European ancestry," "African ancestry," "Hispanic ancestry," and "Asian ancestry" to refer specifically to these HARE-defined groups within MVP, which combine self-identified race/ethnicity with genetic information."

I understand that for MVP, the population labels also include self-identified race/ethnicity which limits the use of solely "genetic ancestry" in describing the population labels. A succinct definition of the ancestry labels early on in the manuscript would aid the reader in understanding the definition of "African ancestry" or "European ancestry" in this study.

We thank the reviewer for this important clarification. We agree that, in MVP, the ancestry labels reflect a combination of self-identified race/ethnicity and genetic information, and thus are not "pure" genetic ancestry labels. As described in the MVP design and HARE methodology papers (PMID: 31564439, 37268996), the Harmonizing Genetic Ancestry and Self-identified Race/Ethnicity (HARE) algorithm uses self-identified race/ethnicity together with genetic principal components to assign participants to analytically useful groups (e.g., "European", "African", "Hispanic", "Asian") that approximate reference genetic ancestries while respecting self-identification. In the revised manuscript, we now define these labels explicitly early in the Methods and clarify that our use of terms such as "European ancestry" and "African ancestry" refers to these HARE-derived groups within MVP, rather than to global populations or social constructs of race. This added definition will help readers correctly interpret the population labels used in our analyses:

"Individual ancestries for European, African, Hispanic and Asian were estimated and defined using the algorithm of Harmonizing Genetic Ancestry and Self-identified Race/Ethnicity (HARE), which incorporated both self-reported race/ethnicity and genetic profile and successfully assigned ancestries for > 98% participants to the following four non-overlapping ancestry groups: non-Hispanic white (European), non-Hispanic Black (African), Hispanic, and non-Hispanic Asian participants. We therefore use the terms "European ancestry," "African ancestry," "Hispanic ancestry," and "Asian ancestry" to refer specifically to these HARE-defined groups within MVP, which combine self-identified race/ethnicity with genetic information."

For BioVU additional details are needed to understand how ancestry was inferred with STRUCTURE 2.2.

We thank the reviewer for this helpful suggestion. We have expanded the BioVU Methods section to provide additional details on ancestry inference. Briefly, in BioVU, global genetic ancestry was inferred using STRUCTURE 2.2 applied to a panel of ancestry-informative markers, as previously described (PMID: 20733501). In this work, 360 ancestry-informative SNPs genotyped in BioVU participants were used to estimate individual membership probabilities in ancestry clusters corresponding to reference European and African populations, and the resulting genetically inferred ancestry showed high concordance with administratively assigned race/ethnicity. In the Methods section, we now explicitly reference this approach and its validation in BioVU (below), and we cite both Dumitrescu et al. (PMID: 20733501) and Hall et al. (PMID: 24896101), which further demonstrate concordance between administratively assigned race and genetic ancestry in this biobank.

"In BioVU, genetic ancestry was inferred using STRUCTURE 2.2 applied to a panel of 360 ancestry-informative markers genotyped on the Illumina DNA Test Panel, as previously described. Briefly, for each individual, probabilities in ancestry clusters were estimated corresponding to reference European and African populations; participants were assigned to European- or African-ancestry groups based on their highest posterior membership probability (PMID: 20733501, 24896101)."

Technically, GWAS from the Finnish population are described as Finnish rather than European due to population structure that is unique compared to non-Finnish Europeans (https://www.nature.com/articles/nature19057). So the meta-analysis including FinnGen results could be described with more nuance, including in the methods (line 279).

We thank the reviewer for highlighting this important nuance. We agree that Finnish individuals represent a genetically distinct population within Europe and that FinnGen GWAS are more accurately described as being conducted in a Finnish population rather than in a generic "European ancestry" group. In the revised manuscript, we have updated the Methods to refer to FinnGen as a Finnish population-based cohort:
"FinnGen is a research initiative that collaborates with both public and private entities and uses imputed genotype data from Finnish biobanks linked to nationwide digital health records (PMID: 36653562). This study population represents a genetically distinct population within Europe."

2) Line 132-134: It sounds like you needed to relax your significant threshold to find any results from DEPICT for HFpEF. If so, this needs to be made clear "For HFpEF, no gene-sets were significant using only GWS loci, but using nominally significant genomic loci with p-value < 1E-4." Is there a justification for why this particular significance threshold was used?

We thank the reviewer for this helpful comment. The understanding is correct that, the SNP p value threshold was relaxed for HFpEF. Because DEPICT relies on having a sufficient number of independent associated loci to detect gene-set and tissue enrichment, the very small number of HFpEF GWS loci provided inadequate power for enrichment analysis. To increase power in an exploratory manner, we therefore performed an additional DEPICT analysis including

independent loci with $p < 1\times10^{-4}$, a relaxed threshold that is used to balance inclusion of more loci with retention of enrichment for true associations. We now explicitly state in the Results:

"For HFpEF, no gene sets reached significance when restricting the analysis to the genome-wide significant loci. Therefore, in an exploratory analysis, we included nominally significant loci with $p < 1\times10^{-4}$, which resulted in gene sets in relation to abnormal muscle regeneration and abnormal vascular smooth muscle morphology, Tables S15A-S15C."

3) Please be clear about what constitutes novel. In the discussion, line 164, it would be better to summarize the total loci found and then the number of "potentially novel loci. " In the methods, the literature used to define novel should be cited lines 311-313.

We thank the reviewer for this helpful suggestion. We have clarified how novel loci were defined summarized in the Methods and Discussion, respectively. We also use the term "potentially novel" to emphasize that novelty is defined relative to currently published GWAS using a distance-based criterion.

Methods

"Novel genomic loci, when compared with previous HF GWAS (PMID: 31919418, 36376295, 36517512, 37429843, 37503172, 40038546), were defined as non-overlapping physical distance located greater than 500kb from the genetic variant on both sides."

Discussion:

"In this large multi-ancestry study, we identified 46 genome-wide significant loci for HFrEF, 3 loci for HFpEF, and 136 loci for all-cause HF. Of these, 9 HFrEF loci, 1 HFpEF locus, and 12 all-cause HF loci were potentially novel compared with previously reported HF GWAS (PMID: 31919418, 36376295, 36517512, 37429843, 37503172, 40038546)."

4) Line 166-168: Are the heterogeneous effects in HFpEF simply due to issues of power given that HFrEF has 9 GWS loci and HFpEF only has 1? For the 91% of HFrEF loci with heterogeneous effects in HFpEF—how are you counting loci here? Is it instead genome-wide significant variants in the 9 loci? This may be a flawed estimate. Perhaps it is better to report the number of lead SNPs at each of the 9 loci that has a heterogeneous effect in HFrEF. Finally, the conclusion "this suggests HFrEF and HFpEF have distinct underlying mechanisms..." should be softened as this has been generally known from clinical findings and previous research. These findings add to the evidence that HFrEF and HFpEF have distinct underlying mechanisms.

We appreciate the reviewer's thoughtful comments on the interpretation of heterogeneity between HFrEF and HFpEF. In our analysis, heterogeneity was assessed at the lead SNP for each of the 46 multi-ancestry HFrEF loci using Cochran's Q test comparing the effect estimates for HFrEF versus HFpEF (Table 3). The 91% refers to 42 of the 46 HFrEF lead SNPs showing evidence of heterogeneity (Q test $p < 0.05$), not to the number of HFpEF loci. We have clarified in the text that the heterogeneity is evaluated at the level of HFrEF lead SNPs. Additionally, we have revised the Discussion to report the absolute counts (42 of 46 loci) rather than only a percentage. Rather than implying that our results newly demonstrate distinct mechanisms, we now state that our findings add to existing clinical and genetic evidence that HFrEF and HFpEF only partially share genetic determinants:

"When we compared effect estimates between subtypes at the lead SNPs for the 46 multi-ancestry HFrEF loci, 42 showed evidence of heterogeneous effects between HFrEF and HFpEF (Cochran's Q $p < 0.05$, Table 3). Taken together with the limited overlap in genome-wide significant loci for HFpEF, these findings add to prior clinical and genetic evidence that HFrEF and HFpEF have only partially shared genetic determinants and likely involve partly distinct underlying biological mechanisms."

5) Lines 171-172: The sentence should be revised for clarity, but it also should be more specific — this shows there could be a shared genetic etiology. There are better references instead of reference 17. I would suggest the GWAS studies that first linked FTO to BMI or more recent GWAS by large consortia like GIANT, as well as work by Melina Claussnitzer and colleagues to disentangle this locus.

We thank the reviewer for this helpful suggestion. We have revised the text to emphasize shared genetic rather than disease etiology between obesity and HF subtypes, and cite foundational GWAS and mechanistic work at the *FTO* locus, including the first BMI GWAS identifying *FTO* as an obesity susceptibility locus and subsequent large consortia and functional studies.

“The *FTO* locus, originally identified as the first common obesity-susceptibility locus in BMI GWAS and repeatedly implicated in large consortia and mechanistic studies, supports a shared genetic etiology between obesity and both HFrEF and HFpEF (PMID: 17434869, 20935630, 26287746, 24247219).”

6) Table 1 should be condensed for readability in the manuscript with the current version as a supplementary table. I would suggest including only the meta-analysis summary statistics in the manuscript. The novel columns should be collapsed into one.

Thank you for this comment. In the revised manuscript, we have reduced the original Table 1 to only meta-analysis and the external replication in Biobank Japan, and moved the remaining ancestry-specific summaries to Table S2. We have also collapsed the two columns for novel loci into one. Please see our response to Reviewer #1, Point 9, for the updated table.

7) The abstract mentions Hispanic and Asian ancestries and the summary statistics are listed in Table 1, but the manuscript does not delve into any ancestry-specific findings from these populations. If there were not any significant loci this should be explained in the discussion.

We thank the reviewer for this comment. In our ancestry-specific meta-analyses, no loci reached genome-wide significance in the Hispanic- or Asian-ancestry groups, primarily due to the smaller number of HF cases and corresponding limited statistical power. For this reason, we focused our narrative on ancestry-specific findings in European- and African-ancestry groups, while still providing ancestry-stratified effect estimates for all four ancestry groups (including Hispanic and Asian) in the original Table 1 (now Table S2 in the revised manuscript) and the corresponding supplemental tables. We have now explicitly stated in the Discussion:

“Although our study included participants of European, African, Hispanic, and Asian ancestry, the numbers of HFrEF and HFpEF cases of Hispanic and Asian ancestry were modest. As a result, no loci reached genome-wide significance in ancestry-specific analyses for these groups, and Hispanic and Asian participants contributed primarily through the multi-ancestry meta-analyses. Larger HF cohorts in Hispanic- and Asian-ancestry populations will be needed to achieve adequate power for ancestry-specific discovery and to more fully characterize genetic risk in these groups.”

8) The discussion lacks any elaboration on the limitations of the study.

We thank the reviewer for this important suggestion. We have now added a dedicated paragraph toward the end of the Discussion section, which reads as follows:

“Our study has several limitations. The MVP cohort is predominantly composed of male veterans, which limits our power for sex-stratified analyses, particularly among women, and may constrain the generalizability of our findings to female. Larger and more demographically diverse cohorts, including those with a higher proportion of women, will be needed to fully characterize sex-specific genetic architecture and to confirm the applicability of these findings across broader clinical populations. Although our study included participants of European, African, Hispanic, and Asian ancestry, the numbers of HFrEF and HFpEF cases of Hispanic and Asian ancestry were modest. As a result, no loci reached genome-wide significance in ancestry-specific analyses for these groups, and Hispanic and Asian participants contributed primarily through the multi-ancestry meta-analyses. Larger HF cohorts in Hispanic- and Asian-ancestry populations will be needed to achieve adequate power for ancestry-specific discovery and to more fully characterize genetic risk in these groups. In addition, although we compared effect estimates across ancestries and reported heterogeneity statistics, we did not apply a formal power-adjusted framework for non-European populations. Therefore, some loci that appear non-significant or attenuated in non-European ancestries may simply reflect limited statistical power rather than true ancestry-specific absence of effect. Finally, although we demonstrate internal consistency across ancestries and cohorts and external replication in BioBank Japan, further validation in independent, deeply phenotyped non-European populations with detailed HF subtyping will be important to confirm and extend our findings.”

9) Lines 288-291: How are related individuals considered in MVP and BioVU? Despite stratification by ancestry, I worry about additional population structure due to admixture in the samples. Population stratification and relatedness can be addressed by using logistic mixed models like SAIGE or REGENIE. Given some of the larger genomic control values, I would like to see a sensitivity analysis at least with an LMM in a non-European ancestry sample.

We thank the reviewer for this important comment. We apologize for the lack of clarity in our original description. In both MVP and BioVU, we used REGENIE (PMID: 34017140), which implements a two-step logistic mixed model framework that accounts for sample relatedness and population structure, rather than standard logistic regression in

PLINK2 as previously stated. We have revised the Methods section accordingly to clarify the use of REGENIE and its handling of relatedness and population structure (below). To further assess residual confounding, we examined genomic control inflation factors and LD Score Regression (LDSC) intercepts, which are summarized in Table S9-S10. The LDSC intercepts were close to 1 (1.0272 for HFrEF, 1.0158 for HFpEF, and 1.0649 for all-cause HF), indicating no concern of residual population stratification or relatedness.

"For the MVP and BioVU, autosomal SNPs were analyzed using logistic mixed models implemented in REGENIE (PMID: 34017140) for HFrEF, HFpEF, and all-cause HF, separately by study and ancestry. Under an additive genetic model, REGENIE was run in its standard two-step framework. We first fitted a whole-genome ridge regression model using high-quality genotyped autosomal variants after linkage disequilibrium (LD) pruning (window size 50 SNPs, shift window size 5 SNPs, LD threshold 0.5). Then, we tested imputed SNP dosages for association with each HF phenotype using logistic mixed models, adjusting for age at enrollment, sex, and the top 10 principal components derived from the full genomic data, separately by study and ancestry. This mixed-model framework accounts for both relatedness and residual population structure within each ancestry group."

10) Figure 2 could be moved to supplement.

We have moved the original Figure 2 to Figure S3 as suggested.

Minor comments:

Abstract

1) This sentence needs revision for clarity and accuracy:

"Four HFrEF loci in African ancestry, including CD36, SPI1, TRIM48, and SPNS3, which were rare in European ancestry."

First, as an incomplete sentence it should be revised for clarity. Second, the loci themselves cannot be rare, but the alleles in these loci can be. Third, the way ancestry is represented here could be more accurate.

For example… "the allele frequency of the risk allele is considered rare in study participants with a genetic ancestry similar to that of European reference populations."

We thank the reviewer for this helpful suggestion. We have revised the sentence for clarity and accuracy, while following the 150 word limit in the abstact:

"Four HFrEF loci were detected in African ancestry participants (near CD36, SPI1, TRIM48, and SPNS3), with low risk-allele frequencies in European reference populations."

Introduction

2) Specify US dollars on line 54 if that is the currency from the reference

Thank you for pointing this out. We have revised the text to specify the currency:
"…an estimated annual cost of 53 billion US dollars (USD)…"

3) Period after LVEF on line 56 to make the subtype description more clear

Thank you for this helpful suggestion. We have revised the sentence to improve clarity:
"The clinical subtypes of HF are defined by left ventricular ejection fraction (LVEF). HF with reduced ejection fraction (HFrEF) is defined as LVEF $\leqslant$ 40%, and HF with preserved ejection fraction (HFpEF) is defined as LVEF $\geqslant$ 50%."

4) Case/control sample sizes for the GWASs cited on line 62/63 and 67 will give the reader a sense of how these compares to the current study

We thank the reviewer for this helpful suggestion. We have revised the Introduction to explicitly report the case/control sample sizes of the key prior HF GWAS (Shah et al., Rasooly et al., Levin et al., and Joseph et al.), so that readers can directly compare those studies with our current analysis:

"Recent large genome-wide association study (GWAS) of all-cause HF among > 90,000 cases and > 1 million controls of European ancestry identified 39 genomic loci associated with all-cause HF (PMID: 31919418, 37429843), and a multi-ancestry GWAS including in > 200,000 cases and > 2 million controls identified 176 loci (PMID: 36376295, 40038546, 40195560)."

"The recent GWAS of HF subtypes among Europeans, which included 19,495 HFrEF cases, 19,589 HFpEF cases, and 258,943 controls, identified 13 loci associated with HFrEF but only one associated with HFpEF, despite a comparable sample size between the two subtypes (PMID: 36517512)."

Results

5) Are the loci mentioned in lines 84-87 also genome wide significant? If so specify as you have in the previous paragraph or drop "GWS" in the text since the sub-section title specifies these are GWS.

We appreciate this comment. Yes, these loci reached genome-wide significance. We have revised the text to explicitly state that these are genome-wide significant (GWS) loci, consistent with the description in the previous paragraph:

"In the ancestry-specific meta-analyses, for European ancestry, we identified 29 GWS HFrEF loci and 1 GWS HFpEF locus *FTO*, Table S4. For African ancestry, we identified 7 GWS HFrEF loci and 2 GWS HFpEF loci, Table S5. Additionally, the all-cause HF GWAS meta-analyses revealed GWS 102 loci for European ancestry and 3 loci for African ancestry, Tables S6-S7."

6) Line 94-95: the effect sizes significantly higher in one ancestry versus another? If so, include a p-value and the type of statistical test used.

We appreciate this comment. Yes, for HFpEF the ancestry-specific effect sizes at the *FTO* and *14q32.13* loci are significantly different between European and African ancestry. We now report the heterogeneity P-values from Cochran's Q test to quantify these differences and have clarified this in the Results:

"For HFpEF, the effect for the risk alleles at the *FTO* and *14q32.13* loci were heterogeneous across European and African ancestries, with statistical significance from the Cochran's Q test p value of 0.015 and 3.2×10^{-8}, respectively, Table 2."

7) Line 100-101: ARSG showed a null effect but does that mean the other two were significant in the same direction of effect? If so, please describe more clearly.

We thank the reviewer for this suggestion. Of the three HFpEF loci, *ARSG* locus showed a null effect in HFrEF (Table 3). The other two loci showed the same direction of effect in HFrEF. We have revised the text to clarify this point and refer the reader to Table 3 for the detailed ancestry-specific estimates:

"Of the three multi-ancestry HFpEF loci, *FTO* and *21q21.1* exhibited directionally concordant and risk-increasing effects on HFrEF, whereas ARSG showed no evidence of association with HFrEF, Table 3."

8) Line 105-109: Pleas remind the reader how many African-ancestry specific HFrEF and HFpEF and all-cause HF loci were tested in the PheWAS e.g. "HFrEF loci (n=7)"

We thank the reviewer for this helpful comment. Our primary rationale was to evaluate whether four African-enriched loci (*CD36, SPI1, TRIM48,* and *SPNS3*), which are rare in European populations, are linked to a broader spectrum of HF-related traits such as cardiomyopathy, hypertensive heart disease, and arrhythmias. We have now clarified this rationale in the Methods, and specified the four loci that were tested:

"To explore pleiotropy and provide additional biological context for African-specific HF loci *CD36, SPI1, TRIM48,* and *SPNS3*, we conducted an exploratory, hypothesis-generating PheWAS of African-ancestry risk variants for HFrEF, HFpEF, and all-cause HF in the MVP African cohort. Phenotypes were derived from the VA electronic health record using the established MVP phenome framework, in which ICD-9 and ICD-10 diagnosis codes are aggregated into clinically meaningful phecodes by grouping related codes into disease categories (PMID: 33984066). Using this framework, we analyzed 1,709 phecode-based phenotypes (PMID: 33984066, 20335276). For each HF risk variant-phecode pair, we fit logistic regression models adjusting for age, sex, and the top five principal components."

9) Line 125-130: It is not adequately clear to the reader that this is based on eQTLs from GTEx. Despite being described in the methods, a more clear phrasing in the results is needed to explain the connection between SNPs/loci and tissue types (e.g. tissue-speciifc eQTL?, significant eQTL in a given tissue?)

We thank the reviewer for this helpful comment. We have revised the Results to explicitly describe this connection between SNPs/loci, eQTLs, and tissue types:

"Using tissue-specific expression quantitative trait loci (eQTL) data from the GTEx database, we found that multi-ancestry HFrEF loci harbored significant *cis*-eQTLs in tibial artery, coronary artery and aorta, indicating the potential mechanism of narrowing or blockage of the arteries, coronary artery disease, and arterial stiffness of the aorta. In

contrast, multi-ancestry HFpEF loci showed significant eQTLs in pituitary gland, adrenal gland, and thyroid, suggesting mechanisms in fluid balance, blood pressure regulation, and cardiac function, Tables S13-S14."

10) Lines 146-150: Please remind the reader where the pQTL results are from. It would also be advisable to soften the conclusions—the analysis revealed potential molecular mechanisms but not "the" molecular mechanisms.

We appreciate this comment. We have revised the text to explicitly describe the source of pQTL results and softened the language referring to "potential molecular mechanisms":

"Using protein-quantitative trait loci from the Fenland proteogenomic study of 4,775 protein targets (PMID: 34819519), we examined the risk loci for all-cause HF and its subtypes to explore potential molecular mechanisms. These analyses indicated that genetic variants at HF risk loci may potentially influence circulating protein expression levels. For example, rs600038 and rs597808 were each associated with the levels of more than 180 protein targets, Tables S17A-S17C."

11) Lines 146-150: Why were pQTL and enhancers grouped together in the results? Enhancers would likely fit better with more the regulatory related findings such as the tissue expression and enrichment section.

We appreciate this helpful suggestion. Accordingly, we have moved the results of overlap with enhancers to the section "Gene-based analysis, tissue expression, enrichment analysis and overlap with enhancers".

12) Lines 146-150: Please also remind the reader how these regions of enhancers are related to the GWAS findings. For example, do the significant loci fall in enhancer regions? If so, how are these enhancer regions described. Could you name which loci seem to be enhancer-related?

We have revised the Results text to state that we used EnhancerAtlas 2.0 database (PMID: 27515742), which included 295 human tissue and cell specific enhancers, and that many of the lead HF loci fall within enhancer regions with examples:

"To further investigate potential regulatory mechanisms, we overlapped the lead GWAS variants with tissue-specific enhancer annotations derived from EnhancerAtlas 2.0 database (PMID: 27515742), which included 295 human tissue and cell specific enhancers. A substantial fraction of the HF loci fell within predicted enhancer regions: 35 of the 46 multi-ancestry HFrEF loci, all 3 HFpEF loci, and 112 of the 136 all-cause HF loci overlapped at least one enhancer region in various cell types, Tables S18A-S18C. Notable examples include HFrEF loci at *ACTN2, BAG3, PHACTR1, PRKCA, MAP3K7CL*, and *SMARCB1*, as well as the HFpEF loci *FTO* and *ARSG*, all of which map to enhancer regions that are active in cardiac and vascular cell types."

13) Lines 146-150: Why was this analysis only done for all-cause HF? Were there no findings for the other subtypes? If so, please state this to avoid "cherry picking" results.

We thank the reviewer for this helpful comment. This analysis was in fact performed for all-cause HF and both subtypes (HFrEF and HFpEF). To improve clarity, we have revised the Results wording to: "HFrEF, HFpEF and all-cause HF".

14) Line 152: Use GWS as the acronym for genome-wide significant and please check the rest of the article for consistency.

We thank the reviewer for this suggestion. We have revised the entire manuscript to use GWS consistently as the acronym for genome-wide significant.

15) The interpretation of credible set analyses on line 154 is overstated. The credible set analysis simply suggests the causal variant is included in the given set of SNPs.

We thank the reviewer for this important suggestion. We have revised the text to more accurately reflect that the putative causal variant is likely to be included within the credible set, rather than that causal effects on molecular pathways are established:

"Credible set analyses for the ancestry-specific and multi-ancestry GWS loci of all-cause HF and its subtypes identified sets of SNPs with cumulative posterior probability of 0.9 or 0.95 of including the causal variant, thereby prioritizing a limited number of variants for future functional investigation, Tables S21A-S21C."

16) Line 160: Please state the PIP of FTO instead of PIP > 0.5

We appreciate this question. After examining the original table, we found an error of the shifting of rows. We have now corrected the Table S22, as shown below. We have added the exact PIPs if > 0.5. We have corrected the results statement:

"For HFpEF, 2 of the 3 GWS loci formed 95% credible sets with a 0.95 PIP, however no SNP was identified with PIP above 0.05, Table S22".

Revised Table S22. Fine Mapping of the Genome-wide Significant Loci for the Multi-ancestry Meta-analysis of GWAS on HFrEF and HFpEF (500kb Window Flanking Each Side of the Genetic Variant).

**rsID**	**Chr.**	**Position (hg19)**	**Gene**	**Potential Causal SNPs with PIP > 0.5 (PIP)**	**Shared / Ancestry-specific**	**SNPs in Credible Set Posterior Probability 0.95**
**The HFrEF Loci**						
rs945425	1	16348412	*CLCNKA*	None	EUR_AFR_HIS	11
rs1997998	1	61883027	*NFIA*	None	EUR	8
rs12740374	1	109817590	*CELSR2*	None	EUR	9
rs10429888	1	162024987	*NOS1AP*	1:162024987:G:A (0.52)	EUR_HIS	2
rs34514099	2	11571966	*E2F6*	None	EUR	26
rs62133082	2	37233937	*HEATR5B*	None	EUR	26
rs17076	2	179595117	*TTN*	2:179704973:G:A (0.61)	EUR_HIS	6
					EUR_AFR	20
					EUR_AFR_HIS	31
					AFR_HIS	1
rs6807275	3	14274451	*3p25.1*	None	EUR_AFR	14
					EUR	31
rs34989545	3	49622632	*BSN*	None	EUR_AFR	78
rs17042098	4	111664158	*4q25*	None	EUR_HIS	82
rs11242464	5	138746334	*DNAJC18*	None	EUR_AFR_HIS	77
rs9349379	6	12903957	*PHACTR1*	6:12903957:A:G (0.66)	EUR_HIS	1
rs146170154	6	36646768	*CDKN1A*	6:36647289:G:A (0.51)	EUR_AFR	1
rs1407229	6	53971921	*MLIP*	None	EUR	50
rs66577603	6	79749166	*PHIP*	None	EUR	113
rs10455872	6	161010118	*LPA*	None	EUR	3
rs2270590	7	128455916	*CCDC136*	None	EUR_AFR	14
rs7461129	8	125861374	*8q24.13*	None	EUR_AFR_HIS	7
rs4977575	9	22124744	*RP11-145E5.5*	None	EUR_HIS	2
rs10814273	9	35723234	*TLN1*	None	EUR_AFR	8
rs600038	9	136151806	*9q34.2*	None	EUR_AFR_HIS	9
rs148307915	10	49528021	*MAPK8*	10:49528021:G:A (0.74)	EUR_HIS	1
rs2242254	10	75585307	*CAMK2G*	None	EUR_AFR	53
rs375034445	10	121424815	*BAG3*	None	EUR_AFR	5
rs113123267	11	47379410	*SPI1*	11:47806206:G:A (0.51)	AFR	1
rs11169562	12	51187290	*ATF1*	None	EUR	63
rs4575361	12	124410529	*DNAH10; CCDC92*	None	EUR	59
rs62006566	15	75086534	*CSK*	None	EUR	21
rs7188250	16	53834607	*FTO*	None	EUR	26
rs2224770	17	2205923	*SMG6*	None	EUR_AFR	85
rs9907657	17	7460957	*TNFSF12; TNFSF12-TNFSF13*	None	EUR	28
rs2517953	17	37841211	*PGAP3*	None	EUR_AFR	8
rs145606454	17	65893289	*BPTF*	None	EUR	122
rs7229491	18	46516424	*18q21.1*	18:46516424:G:C (0.52)	EUR_AFR	4
rs56968346	21	30540165	*MAP3K7CL*	None	EUR_AFR	26
rs9608201	22	24166788	*SMARCB1*	None	EUR_AFR	17
**The HFpEF Loci**						
rs62048402	16	53803223	*FTO*	None	EUR_AFR	57
rs113752382	21	18617840	*21q21.1*	None	EUR	26

PIP: Posterior inclusion probability.

Discussion

17) Line 178: Genes CCDC92 and DNAH10 are included without context as to what subtype they are associated with. Please clarify this for the reader.

We thank the reviewer for this helpful comment. We have clarified the subtype association in the text. The revised sentence now reads:

"The HFrEF-associated locus at *DNAH10*/*CCDC92* plays key roles in adipocyte function and differentiation, …"

18) Line 181: Some loci associated with HFrEF are connected to Smads, not all loci. Please define which or write how many. Remind the reader what evidence connects them to Smads? Please also expand Smads to its full name before using the acronym.

Thank you for pointing this out. We have revised the text to clarify that a subset of HFrEF loci are connected to SMAD signaling and to spell out SMAD in full on first use:

"Gene-set enrichment analysis using DEPICT showed that two HFrEF loci, including *CDKN1A* and *MAP3K7*, mapped to protein-protein interaction subnetworks centered on Smad (Sma- and Mad-related) signal transduction proteins, key mediators of transforming growth factor-β (TGF-β) signaling, suggesting a potential contribution of TGF-β/SMAD pathways to HFrEF pathogenesis."

19) Several of the claims in the discussion should be better cited. Is reference 25 sufficient for lines 189-190?

We appreciate the reviewer's suggestion. The reference (PMID: 11560944) is a comprehensive review that already summarizes *CD36* as a multifunctional receptor involved in lipid metabolism, angiogenesis, atherogenesis, and inflammation, and highlights its expression on platelets and other cardiovascular cell types. To more clearly support the specific functions, we have now added additional references (PMID: 11988484, 33210138, 11560944), including recent reviews on *CD36* in cardiovascular disease and metabolic regulation, which further detail its roles.

20) Line 211: You mention CD36 but you also described it in lines 188-192. Please combine the discussion on CD36 for clarity.

We appreciate this helpful suggestion. In response, we have consolidated the discussion of *CD36* into a single, integrated paragraph to improve clarity and avoid redundancy.

Tables

21) Gene names should be italicized

We have revised this thoroughly in the tables and the manuscript body.

22) The test used for the heterogeneity p-value should be stated in the table or legend

We have added the footnote accordingly to Tables S2 and S3:
"**Heterogeneity P-values from Cochran's Q test."

Methods

23) Lines 251-256: If this physician-reviewed algorithm or NLP method have been previously published please cite or more fully describe (perhaps in supplement) for reproducibility

We thank the reviewer for this important point. As shown in our cited previous work (PMID: 29954337), the HF subtype definitions were based on previously developed and validated electronic medical record (EMR) algorithms supported by natural language processing (NLP) and physician review. We have now expanded the description of the algorithm in the Methods to improve transparency and reproducibility:

"We implemented a natural language processing method to analyze both structured and unstructured components of the electronic health records, extracting LVEF values from echocardiography reports including those within the VA system and external healthcare facilities, reducing the risk of misclassification due to missing LVEF data (PMID: 29954337). In brief, the NLP component uses rule-based pattern matching to identify and extract numeric LVEF values, measurement context (e.g., transthoracic vs. transesophageal echocardiography), and timing from free-text echocardiography reports, and then links these values to HF encounters in the structured electronic health records. This algorithm has been previously developed and validated, demonstrating high positive predictive value for HFpEF case identification in an independent cohort (PMID: 29954337)."

24) Line 291: If the age used was age at diagnosis, please specify.

Thank you for this comment. Our models were adjusted for age at enrollment, rather than age at diagnosis. We chose age at enrollment because it reflects the time point at which HF status and covariates were ascertained in the cohort, providing a consistent baseline across participants and avoiding potential variability or missingness in age at first HF diagnosis. We have revised the Methods to clarify this:

"Then, we tested imputed SNP dosages for association with each HF phenotype using logistic mixed models, adjusting for age at enrollment, sex, ..."

25) Why were genetic correlations not estimated between the subtypes and all-cause HF for African ancestry meta-analysis?

Thank you for this important point. We did not estimate genetic correlations between all-cause HF and HF subtypes in the African-ancestry meta-analysis because LD score regression, as implemented in LDSC (PMID: 25642630), relies on accurate linkage disequilibrium (LD) scores derived from a large, ancestry-matched reference panel. The default and most extensively validated LD score reference panels available for LDSC are based on European-ancestry samples, and there is currently no well-powered public LD score resource that closely matches the admixed African-ancestry composition of our MVP samples. Constructing appropriate LD scores from raw genotype data was beyond the scope of the present work, and applying LDSC with a mismatched LD reference could bias the estimates. For these reasons, we restricted LD score–based genetic correlation analyses to the European-ancestry meta-analysis. In addition, the smaller sample sizes for African-ancestry HF subtypes further limit power for reliable LDSC-based genetic correlation analyses. We therefore believe that attempting these analyses in African ancestry would not yield reliable or interpretable results, and we hope the reviewer understands that we are working within the constraints of the best available resources.

26) Line 345: Why was significance defined as a corrected q-value < 0.2 for the gene set enrichment analysis? This is inconsistent with previous thresholds used and should be justified.

Thank you for this helpful comment. In DEPICT (PMID: 25597830), gene-set and tissue-enrichment analyses are performed on a large number of highly correlated, reconstituted gene sets, so conventional multiple-testing correction can be conservative. To align more closely with recent GWAS using DEPICT, we have revised our analyses to use a more stringent false discovery rate (FDR) threshold of $q < 0.05$ (Benjamini-Hochberg) for these enrichment tests. This FDR threshold is consistent with previous DEPICT-based studies (PMID: 30061737, 36273105). Under this more stringent cutoff, our main HFrEF enrichment findings remain significant, whereas no HFpEF gene-set or tissue enrichments pass $q < 0.05$. We have revised the Methods and Results sections accordingly to reflect this change:
Methods:
"FDR (Benjamini and Hochberg, 1995) was applied, with significance defined by a corrected q value < 0.05."

Results:
"For HFpEF, no gene sets reached significance when restricting the analysis to the genome-wide significant loci. Therefore, in an exploratory analysis, we included nominally significant loci with $p < 1\times10^{-4}$, which resulted in gene sets in relation to abnormal muscle regeneration and abnormal vascular smooth muscle morphology, however not reaching statistical significance, Tables S15A-S15C."

27) Line 368: Please cite the Fenland study

We have added this citation (PMID: 34819519).

Figures

28) Figure 3: Need a third color for loci that are genome wide significant in both studies? Please use a correlation coefficient estimate with 95% confidence intervals instead of a fitted line. Loci that are off the diagonal could be labelled.

We have revised the Figure 3 (Figure 2 in the revised manuscript) accordingly:

Revised Figure 2. Scatter Plot for Comparison of the Estimates for the Genome-wide Significant Loci for the Meta-analysis of GWAS on HFrEF Among European Ancestry and African Ancestry. A: Comparison of Effect Sizes. Pearson Correlation Coefficient = 0.27 (95% CI -0.09, 0.57). Loci with Heterogeneity are Labeled. B: Comparison of Risk Allele Frequency. African-specific Loci are Labeled.

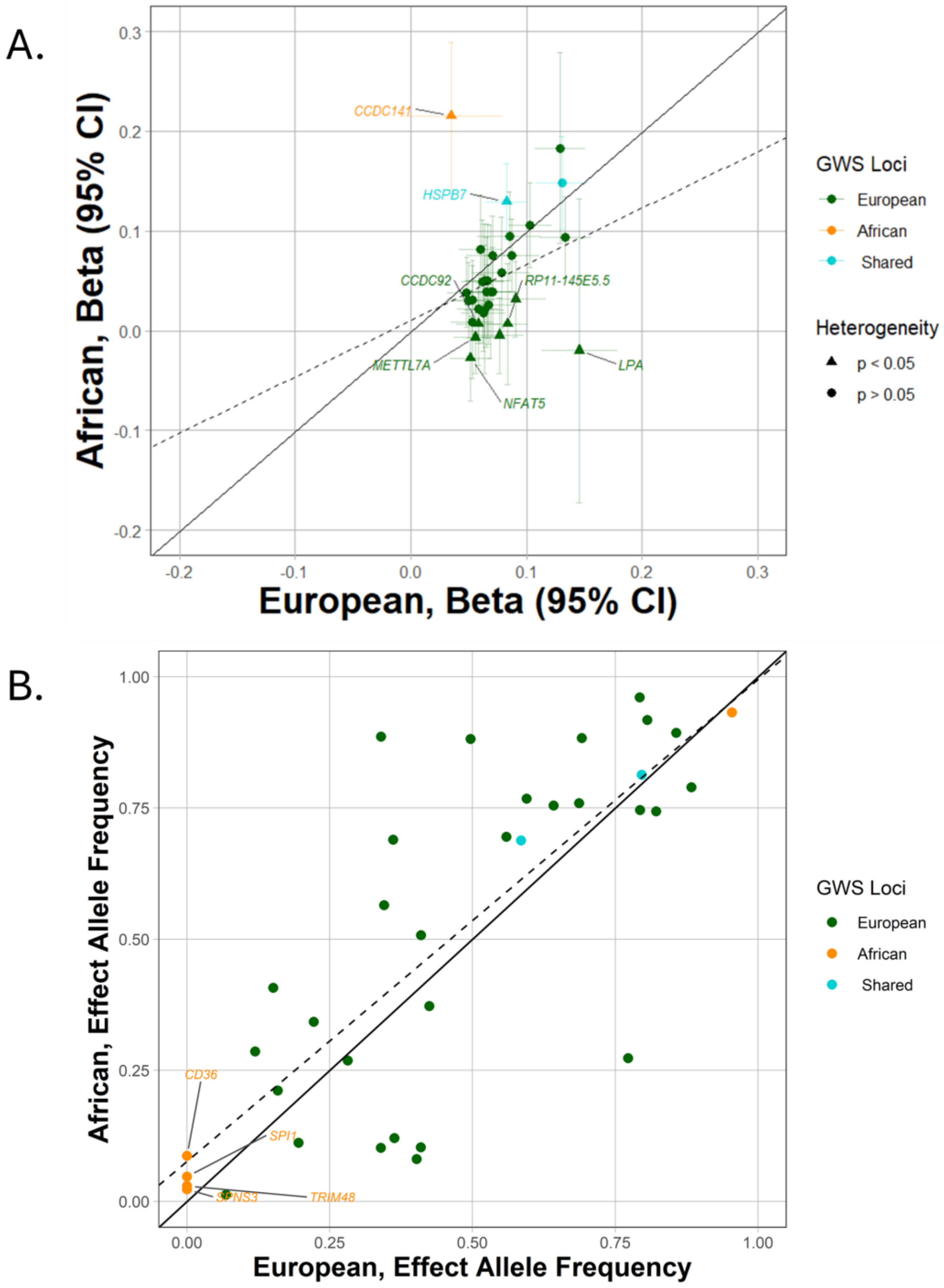

29) Figure 5: Please use colors different from Figure 3 for clarity. Loci that are off the diagonal could be labelled.

We have revised the Figure 5 (Figure 4 in the revised manuscript) accordingly:

Revised Figure 4. Scatter Plot for Comparison of The Estimates for the Genome-wide Significant Loci for the Meta-analysis of GWAS on HFrEF and HFpEF Among Multi-ancestry Cohort. Pearson Correlation Coefficient = -0.003 (95%

CI -0.107, 0.100). The Top 10 Loci with the Largest Differences in Effect Size are Labeled.

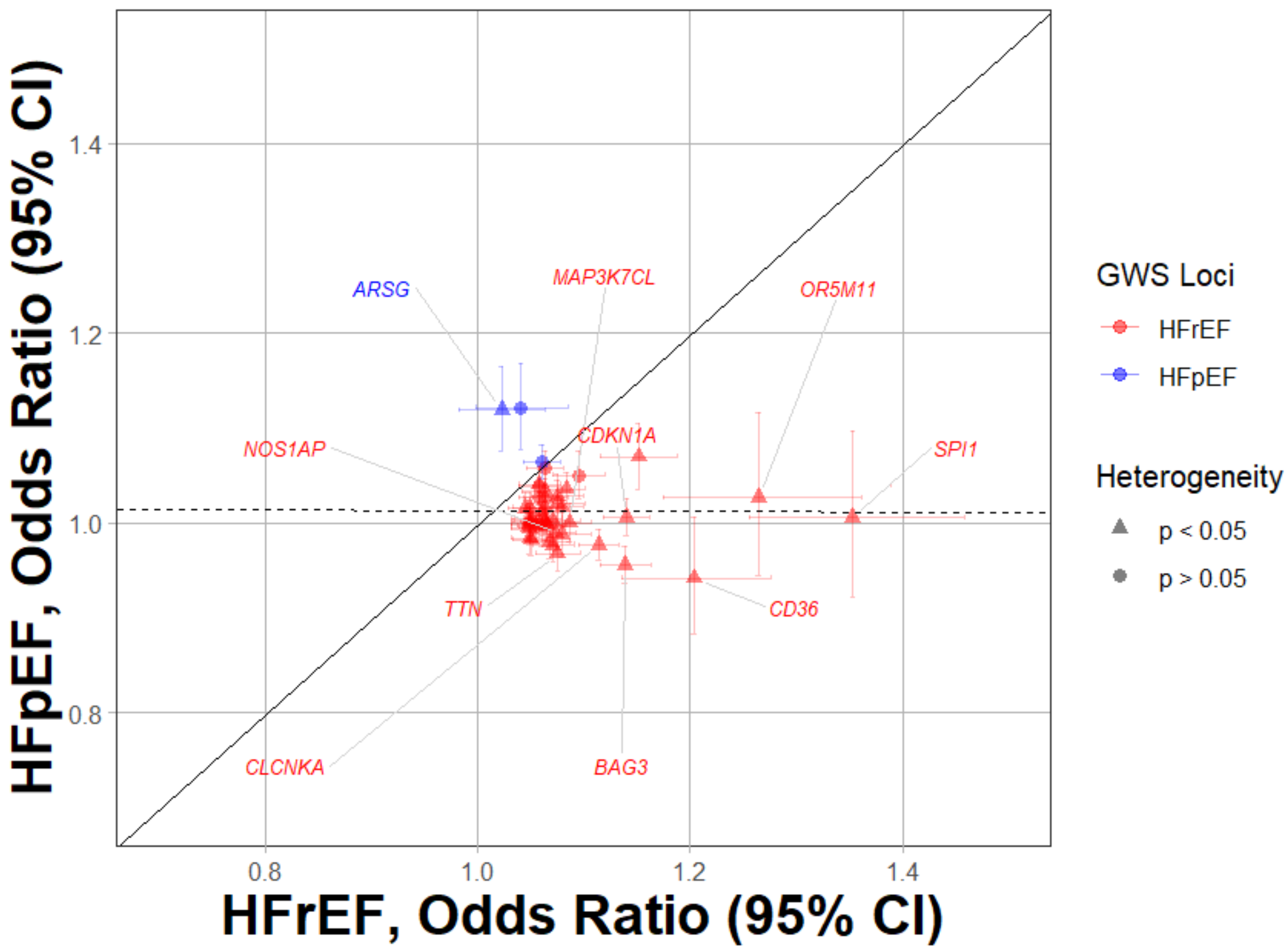

Reviewer #2 (Remarks to the Author):

Addressing the points of my previous review -

*** The h2SNP of the traits should be reported.**

The authors have presented h2snp estimated using GREML-LDMS. This approach requires individual participant data, so I expect these results are from the MVP sample, but this is not specified. I suspect that the estimates are on the observed rather than the liability scale. If so, please convert to the liability scale so that they are comparable to other studies. If not, then the estimates are usually high compared with the h2snp reported in most published GWAS. Unusually, the heritability estimate for HFpEF is higher than for HFrEF, and this should be checked.

• Section on genetic architecture describes locus discovery

The h2 SNP should be given with 95% CI in the results - I suspect these are wide.

We thank the reviewer for this helpful comment. We have clarified that the GREML-LDMS analyses were performed using individual-level genotype data from the Million Veteran Program (MVP), separately within each ancestry group. We also confirm that the reported SNP-based heritability estimates are on the liability scale, not the observed scale, and have revised the Methods and Results to state this explicitly.

For the liability-scale transformation, we used assumed disease prevalences of 5% for HFrEF, 5% for HFpEF, and 10% for all-cause HF. These values were selected to be broadly consistent with the observed burden of HF phenotypes in the MVP analytic sample. In the revised Results, we now report SNP-based heritability estimates with standard errors and 95% confidence intervals. Among individuals of European ancestry, the liability-scale h^2 was 0.31 for HFrEF (SE 0.06; 95% CI: 0.19 to 0.43), 0.26 for HFpEF (SE 0.06; 95% CI: 0.14 to 0.38), and 0.31 for all-cause HF (SE 0.04; 95% CI: 0.23 to 0.39). Among individuals of African ancestry, the corresponding estimates were 0.12 for HFrEF (SE 0.06; 95% CI: 0 to 0.25), 0.28 for HFpEF (SE 0.07; 95% CI: 0.14 to 0.42), and 0.21 for all-cause HF (SE 0.05; 95% CI: 0.12 to 0.30). We also agree with the reviewer that the higher point estimate for HFpEF than HFrEF, particularly among individuals of African ancestry, should be interpreted cautiously. The confidence intervals are wide and overlapping, and the HFrEF estimate among African ancestry participants has substantial uncertainty. In addition, differences in age and sex distributions across HF subtypes and ancestry groups in MVP, as well as differences in case ascertainment and phenotype heterogeneity, may influence heritability estimates. We have added a cautionary statement to the Discussion and avoid overinterpreting modest differences in h^2 estimates across HF subtypes.

Methods: "We estimated SNP-based heritability (h^2) for HF and HF subtypes using GREML-LDMS in GCTA among MVP participants with individual-level genotype data (PMID: 21167468, 26323059). Analyses were performed separately within ancestry groups. Variants

were stratified into minor allele frequency and linkage disequilibrium bins, and genetic relationship matrices were constructed for each bin. REML models included age, sex, and top 10 genetic PCs as covariates. Observed-scale estimates were converted to the liability scale using assumed disease prevalences of 5% for HFrEF, 5% for HFpEF, and 10% for all-cause HF, which were broadly consistent with the observed prevalence of these phenotypes in the MVP analytic sample."

Results: "Using GREML-LDMS in MVP individual-level genotype data, we estimated liability-scale SNP-based heritability for HF and HF subtypes by ancestry. Among individuals of European ancestry, the liability-scale h^2 was 0.31 for HFrEF (SE 0.06; 95% CI: 0.19 to 0.43), 0.26 for HFpEF (SE 0.06; 95% CI: 0.14 to 0.38), and 0.31 for all-cause HF (SE 0.04; 95% CI: 0.23 to 0.39). Among individuals of African ancestry, the corresponding estimates were 0.12 for HFrEF (SE 0.06; 95% CI: 0 to 0.25), 0.28 for HFpEF (SE 0.07; 95% CI: 0.14 to 0.42), and 0.21 for all-cause HF (SE 0.05; 95% CI: 0.12 to 0.30)."

Discussion: "The heritability estimate for HFpEF was higher than that for HFrEF among African-ancestry participants. However, the confidence intervals were wide and overlapping, indicating substantial uncertainty around the relative magnitude of subtype-specific heritability estimates. In addition, differences in sample size, age and sex distributions, phenotype heterogeneity, and case ascertainment across HF subtypes and ancestry groups may influence SNP-based heritability estimates. Therefore, these differences should be interpreted cautiously and should not be taken as definitive evidence that HFpEF has greater genetic contribution than HFrEF in African-ancestry participants."

• The approach to fine mapping and gene prioritisation could be improved to provide a more cohesive approach and to make use of modern tools, such as PoPs.

Thank you for this helpful suggestion. In response, we applied PoPS to prioritize candidate genes for the multi-ancestry meta-analysis results of HFrEF, HFpEF, and all-cause HF. The top PoPS-prioritized genes are now presented in Table S20 (below). To further prioritize candidate genes at HF-associated loci, we applied PoPS to the multi-ancestry meta-analysis results for HFrEF, HFpEF, and all-cause HF. The top PoPS-prioritized genes are shown in Table S20. For HFrEF, these included *NFIA, MAPK8, PHACTR1, CAMK2G, CCDC136, FTO, BPTF,* and *SMG6*. For HFpEF, overlapping genes included *FTO* and *ARSG*. For all-cause HF, overlapping genes included *NFIA, SMG6, PHACTR1*, and *FTO*. These results provide additional support for candidate genes and loci highlighted by our GWAS, TWAS/colocalization, functional annotation, and fine-mapping analyses, while preserving the main interpretation that HF genetic architecture includes both shared and subtype-specific components. We have added the following text to the revised manuscript:

"Methods: We used Polygenic Priority Score (PoPS) (PMID: 37443254) to prioritize candidate genes for HFrEF, HFpEF and all-cause HF. GWAS summary statistics from the multi-ancestry

meta-analyses were used, matching to the 1000 Genomes Phase 3 multi-ancestry reference panel, including all available ancestry populations as the LD reference panel for gene-based analysis. MAGMA (PMID: 25885710) was used to generate gene-level association statistics using the SNP-wise mean gene model. PoPS was then run separately for each HF phenotype. A total of 18,383 genes were tested, and the results were ranked by PoPS score. For each sentinel GWS locus, we identified all genes located within ±500 kb of the sentinel variant and selected the genes with the highest PoPS score as the prioritized candidate gene for that locus.

Results: To further prioritize candidate genes at HF-associated loci, we applied PoPS (PMID: 37443254) to the multi-ancestry meta-analysis results for HFrEF, HFpEF, and all-cause HF. Across 18,383 tested genes, PoPS scores were generated separately for each HF phenotype. For each sentinel GWS locus, we identified genes located within ±500 kb of the sentinel variant and selected the gene with the highest PoPS score as the prioritized candidate gene for that locus. The PoPS-prioritized genes are presented in Table S20. Several PoPS-prioritized genes overlapped with loci or candidate genes highlighted in our GWAS and downstream analyses. For HFrEF, these included *NFIA, MAPK8, PHACTR1, CAMK2G, CCDC136, FTO, BPTF*, and *SMG6*. For HFpEF, overlapping genes included *FTO* and *ARSG*. For all-cause HF, overlapping genes included *NFIA, SMG6, PHACTR1*, and *FTO*."

Table S20. Top PoPS-Prioritized Candidate Genes for Multi-ancestry Meta-analysis of GWAS on HFrEF, HFpEF and All-cause HF. Up to 50 Genes are Shown for Each Phenotype. (Full table is shown in the revised manuscript supplementary tables.)

Phenotype	Rank	Chr.	Start	End	Symbol	Name	Gene Type	PoPS Score
HFrEF	1	1	61330931	61928465	NFIA	nuclear factor I A	protein-coding	0.49
	2	2	36583069	36778278	CRIM1	cysteine rich transmembrane BMP regulator 1	protein-coding	0.35
	3	8	126103921	126379362	NSMCE2	NSE2 (MMS21) homolog, SMC5-SMC6 complex SUMO ligase	protein-coding	0.29
	4	17	1963133	2207065	SMG6	SMG6 nonsense mediated mRNA decay factor	protein-coding	0.26
	5	3	49977440	50137478	RBM6	RNA binding motif protein 6	protein-coding	0.25
	6	12	124808961	125052135	NCOR2	nuclear receptor corepressor 2	protein-coding	0.25
	7	5	137946656	138270723	CTNNA1	catenin alpha 1	protein-coding	0.23
	8	18	46065417	46389588	CTIF	cap binding complex dependent translation initiation factor	protein-coding	0.23
	9	10	75572259	75634343	CAMK2G	calcium/calmodulin dependent protein kinase II gamma	protein-coding	0.23
	10	10	75757872	75879918	VCL	vinculin	protein-coding	0.23
	11	3	12625100	12705725	RAF1	Raf-1 proto-oncogene, serine/threonine kinase	protein-coding	0.23
	12	3	49506146	49573048	DAG1	dystroglycan 1	protein-coding	0.22
	13	2	179966419	180129517	SESTD1	SEC14 and spectrin domain containing 1	protein-coding	0.20
	14	16	53737875	54155853	FTO	FTO alpha-ketoglutarate dependent dioxygenase	protein-coding	0.19
	15	10	121588972	121652068	MCMBP	minichromosome maintenance complex binding protein	protein-coding	0.19
	16	11	100558384	100862668	ARHGAP42	Rho GTPase activating protein 42	protein-coding	0.18
	17	3	49145479	49158371	USP19	ubiquitin specific peptidase 19	protein-coding	0.17
	18	18	46570039	46987717	DYM	dymeclin	protein-coding	0.17
	19	17	65821640	65980494	BPTF	bromodomain PHD finger transcription factor	protein-coding	0.17
	20	22	24407642	24574596	CABIN1	calcineurin binding protein 1	protein-coding	0.16
	21	6	90636248	91006627	BACH2	BTB domain and CNC homolog 2	protein-coding	0.16
	22	22	24666786	24813708	SPECC1L	sperm antigen with calponin homology and coiled-coil domains 1 like	protein-coding	0.16
	23	10	75910960	76469061	ADK	adenosine kinase	protein-coding	0.16
	24	2	11319887	11488456	ROCK2	Rho associated coiled-coil containing protein kinase 2	protein-coding	0.16
	25	6	79645584	79787953	PHIP	pleckstrin homology domain interacting protein	protein-coding	0.16
	26	1	109852192	109940573	SORT1	sortilin 1	protein-coding	0.15
	27	17	7788124	7816078	CHD3	chromodomain helicase DNA binding protein 3	protein-coding	0.15
	28	10	75545340	75561551	ZSWIM8	zinc finger SWIM-type containing 8	protein-coding	0.15
	29	7	80371854	80551675	SEMA3C	semaphorin 3C	protein-coding	0.15
	30	6	12717893	13288645	PHACTR1	phosphatase and actin regulator 1	protein-coding	0.15

	31	3	12938719	13114617	IQSEC1	IQ motif and Sec7 domain ArfGEF 1	protein-coding	0.14
	32	17	1682779	1733928	SMYD4	SET and MYND domain containing 4	protein-coding	0.14
	33	3	14166440	14185179	TMEM43	transmembrane protein 43	protein-coding	0.13
	34	10	120967101	121215131	GRK5	G protein-coupled receptor kinase 5	protein-coding	0.13
	35	6	53659295	53788919	LRRC1	leucine rich repeat containing 1	protein-coding	0.13
	36	17	7362685	7387582	ZBTB4	zinc finger and BTB domain containing 4	protein-coding	0.13
	37	9	35814448	35854844	TMEM8B	transmembrane protein 8B	protein-coding	0.12
	38	6	36461669	36515247	STK38	serine/threonine kinase 38	protein-coding	0.12
	39	7	128828713	128853386	SMO	smoothened, frizzled class receptor	protein-coding	0.12
	40	17	1732996	1803376	RPA1	replication protein A1	protein-coding	0.12
	41	7	128594948	128695198	TNPO3	transportin 3	protein-coding	0.12
	42	7	128430811	128462186	CCDC136	coiled-coil domain containing 136	protein-coding	0.12
	43	1	62208149	62629592	PATJ	PATJ crumbs cell polarity complex component	protein-coding	0.12
	44	11	47428683	47438047	SLC39A13	solute carrier family 39 member 13	protein-coding	0.12
	45	2	11584501	11606297	E2F6	E2F transcription factor 6	protein-coding	0.12
	46	7	127937738	127983962	RBM28	RNA binding motif protein 28	protein-coding	0.12
	47	10	49514698	49647403	MAPK8	mitogen-activated protein kinase 8	protein-coding	0.12
	48	17	65373575	65693372	PITPNC1	phosphatidylinositol transfer protein cytoplasmic 1	protein-coding	0.12
	49	17	7128660	7137864	DVL2	dishevelled segment polarity protein 2	protein-coding	0.12
	50	5	138906016	139008018	UBE2D2	ubiquitin conjugating enzyme E2 D2	protein-coding	0.12
HFpEF	1	17	65821640	65980494	BPTF	bromodomain PHD finger transcription factor	protein-coding	0.11
	2	16	53737875	54155853	FTO	FTO alpha-ketoglutarate dependent dioxygenase	protein-coding	0.10
	3	16	53631595	53737850	RPGRIP1L	RPGRIP1 like	protein-coding	0.05
	4	16	53088945	53363062	CHD9	chromodomain helicase DNA binding protein 9	protein-coding	0.05
	5	16	53524952	53538323	AKTIP	AKT interacting protein	protein-coding	0.04
	6	16	53467889	53525561	RBL2	RB transcriptional corepressor like 2	protein-coding	0.03
	7	17	66507921	66547460	PRKAR1A	protein kinase cAMP-dependent type I regulatory subunit alpha	protein-coding	0.02
	8	17	66243715	66253297	AMZ2	archaelysin family metallopeptidase 2	protein-coding	0.00
	9	17	66255323	66418872	ARSG	arylsulfatase G	protein-coding	0.00
	10	17	66417089	66453654	WIPI1	WD repeat domain, phosphoinositide interacting 1	protein-coding	-0.01
	11	17	65987217	65989765	C17orf58	chromosome 17 open reading frame 58	protein-coding	-0.02
	12	17	66863433	66951533	ABCA8	ATP binding cassette subfamily A member 8	protein-coding	-0.02
	13	21	18884700	18965897	CXADR	CXADR Ig-like cell adhesion molecule	protein-coding	-0.03
	14	17	66031635	66042958	KPNA2	karyopherin subunit alpha 2	protein-coding	-0.03
	15	21	18811208	18821503	LINC01549	long intergenic non-protein coding RNA 1549	ncRNA	-0.03
	16	17	66531254	66597530	FAM20A	FAM20A golgi associated secretory pathway pseudokinase	protein-coding	-0.04
	17	17	66263167	66287408	SLC16A6	solute carrier family 16 member 6	protein-coding	-0.06
	18	21	18965971	18985265	BTG3	BTG anti-proliferation factor 3	protein-coding	-0.06
All-cause HF	1	4	140637907	141075338	MAML3	mastermind like transcriptional coactivator 3	protein-coding	0.97
	2	16	72816784	73093597	ZFHX3	zinc finger homeobox 3	protein-coding	0.58
	3	1	61330931	61928465	NFIA	nuclear factor I A	protein-coding	0.55
	4	3	25215823	25639423	RARB	retinoic acid receptor beta	protein-coding	0.49
	5	7	31790793	32338941	PDE1C	phosphodiesterase 1C	protein-coding	0.46
	6	14	32798479	33300567	AKAP6	A-kinase anchoring protein 6	protein-coding	0.42
	7	1	78354198	78409580	NEXN	nexilin F-actin binding protein	protein-coding	0.39
	8	9	97488983	97849441	AOPEP	aminopeptidase O (putative)	protein-coding	0.38
	9	17	1963133	2207065	SMG6	SMG6 nonsense mediated mRNA decay factor	protein-coding	0.38
	10	8	126103921	126379362	NSMCE2	NSE2 (MMS21) homolog, SMC5-SMC6 complex SUMO ligase	protein-coding	0.37
	11	12	124808961	125052135	NCOR2	nuclear receptor corepressor 2	protein-coding	0.36
	12	10	21823094	22032559	MLLT10	MLLT10 histone lysine methyltransferase DOT1L cofactor	protein-coding	0.35
	13	3	49977440	50137478	RBM6	RNA binding motif protein 6	protein-coding	0.35
	14	7	116593292	116870157	ST7	suppression of tumorigenicity 7	protein-coding	0.34
	15	7	116593292	116870157	ST7-OT3	ST7 overlapping transcript 3	ncRNA	0.34
	16	3	136055077	136471220	STAG1	STAG1 cohesin complex component	protein-coding	0.34
	17	17	77085427	77613550	RBFOX3	RNA binding fox-1 homolog 3	protein-coding	0.34
	18	6	79645584	79787953	PHIP	pleckstrin homology domain interacting protein	protein-coding	0.33
	19	2	201170604	201346986	SPATS2L	spermatogenesis associated serine rich 2 like	protein-coding	0.32
	20	1	237205505	237997288	RYR2	ryanodine receptor 2	protein-coding	0.31
	21	1	50905150	51425935	FAF1	Fas associated factor 1	protein-coding	0.31
	22	3	50126341	50156454	RBM5	RNA binding motif protein 5	protein-coding	0.31
	23	4	26578059	26756973	TBC1D19	TBC1 domain family member 19	protein-coding	0.30
	24	3	27151576	27410951	NEK10	NIMA related kinase 10	protein-coding	0.30
	25	2	148778580	149275805	MBD5	methyl-CpG binding domain protein 5	protein-coding	0.30
	26	1	10696661	10856707	CASZ1	castor zinc finger 1	protein-coding	0.30
	27	3	53528683	53847760	CACNA1D	calcium voltage-gated channel subunit alpha1 D	protein-coding	0.29
	28	7	157331750	158380480	PTPRN2	protein tyrosine phosphatase receptor type N2	protein-coding	0.29
	29	6	12717893	13288645	PHACTR1	phosphatase and actin regulator 1	protein-coding	0.29
	30	18	31431064	31804916	NOL4	nucleolar protein 4	protein-coding	0.29
	31	3	12625100	12705725	RAF1	Raf-1 proto-oncogene, serine/threonine kinase	protein-coding	0.28
	32	16	24741016	24838953	TNRC6A	trinucleotide repeat containing adaptor 6A	protein-coding	0.28
	33	7	22980878	23053749	HYCC1	hyccin PI4KA lipid kinase complex subunit 1	protein-coding	0.28

	34	1	10270863	10441661	KIF1B	kinesin family member 1B	protein-coding	0.28
	35	7	156931607	157062066	UBE3C	ubiquitin protein ligase E3C	protein-coding	0.28
	36	11	43380482	43516483	TTC17	tetratricopeptide repeat domain 17	protein-coding	0.28
	37	17	46626232	46682274	HOXB3	homeobox B3	protein-coding	0.27
	38	2	76974845	77820445	LRRTM4	leucine rich repeat transmembrane neuronal 4	protein-coding	0.27
	39	10	104005289	104142656	GBF1	golgi brefeldin A resistant guanine nucleotide exchange factor 1	protein-coding	0.27
	40	1	190066792	190446759	BRINP3	BMP/retinoic acid inducible neural specific 3	protein-coding	0.27
	41	1	236849754	236927931	ACTN2	actinin alpha 2	protein-coding	0.26
	42	13	111530887	111567416	ANKRD10	ankyrin repeat domain 10	protein-coding	0.26
	43	16	53737875	54155853	FTO	FTO alpha-ketoglutarate dependent dioxygenase	protein-coding	0.26
	44	17	15932471	16121499	NCOR1	nuclear receptor corepressor 1	protein-coding	0.25
	45	10	22045466	22292698	DNAJC1	DnaJ heat shock protein family (Hsp40) member C1	protein-coding	0.25
	46	2	11319887	11488456	ROCK2	Rho associated coiled-coil containing protein kinase 2	protein-coding	0.25
	47	19	10764937	10803093	ILF3	interleukin enhancer binding factor 3	protein-coding	0.25
	48	3	183637722	183735803	ABCC5	ATP binding cassette subfamily C member 5	protein-coding	0.25
	49	10	75757872	75879918	VCL	vinculin	protein-coding	0.25
	50	5	173315283	173388979	CPEB4	cytoplasmic polyadenylation element binding protein 4	protein-coding	0.25

The authors highlight the challenge of implementing PoPs as a method for locus to gene prioritisation due to the difficulty of developing a well-specified LD reference, but then a number of the methods used also rely on an LD SNP correlation matrix (MAGMA, TWAS, colocalisation, Mendelian randomisation). It is not clear what references were used for these analyses - perhaps a within-sample LD matrix was computed from the MVP cohort, and the mapping was done in this GWAS subset?

There are similar issues in other sections of the manuscript.

We thank the reviewer for this important comment. We intended to perform post-GWAS analyses in a multi-ancestry framework where possible. However, several LD-dependent methods require well-calibrated ancestry-matched LD references, prediction weights, or genetic instruments, which were not uniformly available or sufficiently powered across all ancestry groups. Therefore, downstream analyses including TWAS, and colocalization were restricted to European-ancestry GWAS summary statistics, where only the European validated reference resources were available for the existing analysis pipeline. Mendelian randomization analyses were restricted to European-ancestry GWAS summary statistics because the available genetic instruments for the exposures were derived primarily from European or European-dominant populations. We have revised the Methods to explicitly state that we did not use a single universal LD reference for all analyses. For fine-mapping within MVP, ancestry-specific LD was estimated from MVP individual-level genotype data where possible. We also summarized these in the Response Table 1 below.

We have added the following clarification to the Methods:

MAGMA gene-based analysis:

"We used the 1000 Genomes Phase 3 all-population reference panel, including European, African, Admixed American, East Asian, and South Asian populations, as the LD reference panel."

PoPS gene prioritization:

"We used Polygenic Priority Score (PoPS) (PMID: 37443254) to prioritize candidate genes for HFrEF, HFpEF and all-cause HF. GWAS summary statistics from the multi-ancestry meta-analyses were used, matching to the 1000 Genomes Phase 3 multi-ancestry reference panel, including all available ancestry populations as the LD reference panel for gene-based analysis."

LD score regression and genetic correlation:

"LD score regression analyses, including LDSC intercept estimation and genetic correlation analyses, were restricted to European-ancestry GWAS summary statistics and used the 1000 Genomes Phase 3 European LD score reference panel."

TWAS:

"TWAS analyses were restricted to European-ancestry GWAS summary statistics and used FUSION/GTEx expression weights with matched European LD reference panels."

Mendelian randomization:

"LD clumping of genetic instruments was performed using the 1000 Genomes Phase 3 European reference panel, consistent with the ancestry of the exposure GWAS instruments."

LDlinkR proxy variant and credible-set analysis:

"To identify credible sets of causal variants associated with HF subtypes and all-cause HF, we utilized the R package LDlinkR to identify proxy and putative functional variants within a two-sided window of 500 kb of each GWS SNP with a pairwise R^2 value > 0.01 based on the 1000 Genomes reference panel."

MESuSiE fine-mapping:

"Ancestry-specific LD matrices were estimated from MVP individual-level genotype data for the corresponding European, African, and Hispanic ancestry groups."

Response Table 1. Summary of Post-GWAS Analysis and LD Reference Panel Used.

**Analysis**	**GWAS ancestry used**	**LD/reference panel used**	**Notes**
MAGMA gene-based analysis	Multi-ancestry GWAS	1000 Genomes Phase 3 all-population reference panel, including European, African, Admixed American, East Asian, and South Asian populations,	Used for SNP-to-gene aggregation and gene-level statistics.
PoPS gene prioritization	Multi-ancestry GWAS	Same MAGMA output generated using the 1000 Genomes Phase 3 all-population LD reference panel.	PoPS used MAGMA gene-level statistics, no additional LD matrix was estimated.
LDSC intercept / genetic correlation	European ancestry only	1000 Genomes Phase 3 European LD score reference.	Restricted to European ancestry because well-

			powered matched LD scores were available.
TWAS, colocalization using FUSION	European ancestry only	FUSION/GTEx European LD reference panel. No external LD matrix required for standard coloc analysis.	Restricted to European ancestry to match GTEx expression weights and LD structure.
MR instrument clumping	European ancestry only	European 1000 Genomes LD reference panel.	Exposure instruments were selected from European or European-dominant GWAS, outcome GWAS was European ancestry.
LDlinkR proxy variant search	Ancestry-specific or multi-ancestry loci	1000 Genomes ancestry-matched reference where possible; all-population reference for multi-ancestry loci.	Used to identify proxy variants around GWS SNPs.
corrcoverage credible sets	Ancestry-specific or multi-ancestry loci	1000 Genomes ancestry-matched or all-population LD reference.	Used for Bayesian credible-set construction.
MESuSiE fine-mapping	MVP European, African, and Hispanic ancestry groups	Within-sample MVP ancestry-specific genotype-derived LD matrices.	Used individual-level genotype data to account for ancestry-specific LD.
pQTL annotation / enhancer overlap / chromatin annotation	Multi-ancestry loci	No LD matrix used.	Direct SNP annotation or overlap analyses.
PheWAS	MVP African ancestry	No LD matrix used.	Tested sentinel variants directly in MVP African ancestry.

Reviewer #1

2. Thank you to the authors for looking into these pathogenic variants in clinker. It may be useful to point out in the discussion of TTN or in the limitations section the scenario in which rare pathogenic variants for cardiomyopathy are driving synthetic associations in these loci.

We thank the reviewer for this helpful suggestion. We have added this point to the Discussion:

"Several identified loci, including *TTN*, overlap genes known to harbor rare pathogenic variants for inherited cardiomyopathies, particularly dilated cardiomyopathy (PMID: 22335739). Although the identified GWAS signals were driven by common or low-frequency sentinel variants, we cannot exclude the possibility of synthetic associations, whereby unmeasured rare pathogenic variants contribute to the observed association signals (PMID: 20126254)."

3. Thank you for adding a Mendelian Randomization analysis. Typically, you use a primary method for inference and other methods are used as sensitivity analysis. Are you using the standard IVW p-value for determining significance? The methods and results should be more clear what is used for inference and if you're then requiring directional consistency and consistent effects after outlier removal in the sensitivity analyses. Weighted median estimation and weighted mode estimation would be additional sensitivity analysis methods to include. Please also make clear if fixed-effects IVW was used and where the pleiotropy test p-value in the table is coming from (e.g. MR-Egger regression intercept test)

We have revised the Methods and Results to clarify the inferential framework for the Mendelian randomization analyses. Specifically, we used fixed-effects IVW as the primary MR method. We additionally performed MR-Egger regression, weighted median, and weighted mode analyses to evaluate the robustness of the IVW findings under different assumptions about horizontal pleiotropy and invalid instruments. We also performed outlier detection/removal analyses and compared results before and after removal of potential outlier variants. We now state explicitly that MR findings were interpreted as more robust when the IVW association was statistically significant, the direction of effect was consistent across sensitivity methods, and the association was not materially changed after outlier removal. We have also clarified that the pleiotropy test P value reported in the table is from the MR-Egger regression intercept test, which evaluates evidence for directional horizontal pleiotropy.

Methods:

Mendelian randomization (MR)

Based on the results from the European ancestry, MR was performed to explore the potential bi-directional causal associations between HFrEF, HFpEF and several risk factors, including coronary artery disease, type 2 diabetes, body mass index, high-density lipoprotein cholesterol, low-density lipoprotein cholesterol, total cholesterol, triglycerides, systolic blood pressure,

diastolic blood pressure, and pulse pressure. The genetic instruments for the risk factors were selected from recent large-scale GWAS of European or European-dominant populations (PMID: 26343387; 30297969; 25673413; 24097068; 30224653). LD clumping of genetic instruments was performed using the 1000 Genomes Phase 3 European reference panel, consistent with the ancestry of the exposure GWAS instruments.

MR analyses were performed using the R package TwoSampleMR (PMID: 29846171). The primary inference was based on the fixed-effects inverse-variance weighted (IVW) method and its two-sided P value. MR-Egger regression, weighted median, and weighted mode methods were performed as sensitivity analyses to assess the robustness of the IVW estimates under different assumptions regarding horizontal pleiotropy and invalid instruments. Potential directional pleiotropy was evaluated using the MR-Egger intercept test. In addition, MR-PRESSO (Mendelian Randomization Pleiotropy RESidual Sum and Outlier) was used to detect and correct for pleiotropic outlier variants (PMID: 29686387), and MR analyses were repeated after removing genetic outliers that could bias the causal estimates. MR findings were interpreted as more robust when the IVW association was statistically significant, the direction of effect was consistent across sensitivity methods, and the estimates were not materially changed after outlier removal.

Results:

Coronary artery disease, body mass index, low-density lipoprotein cholesterol, systolic blood pressure, and diastolic blood pressure showed causal effects on HFrEF, whereas high-density lipoprotein cholesterol and total cholesterol did not. Type 2 diabetes, triglycerides, and pulse pressure demonstrated potential causal effects for HFrEF. For HFpEF, coronary artery disease, body mass index, systolic blood pressure, diastolic blood pressure, and pulse pressure showed causal effects. Lipids did not demonstrate evidence of a causal effect for HFpEF, Tables S25A-S25B.

Table S25A. Mendelian Randomization for the Causal Effect of Risk Factors on HFrEF.

**Exposure**	**Model**	**N GIVs**	**OR (95%) for Binary Outcome<br>Beta (95% CI) for Continuous Outcome**	**P**	**Pleiotropy Test P**
**CAD**	MR Egger	52	1.49 (1.38, 1.62)	5.61e-13	0.8708
	IVW		1.5 (1.44, 1.57)	1.68e-78	
	Weighted Median		1.5 (1.41, 1.59)	1.26e-38	
	Weighted Mode		1.51 (1.01, 2.27)	0.050	
	MR Egger (MR-PRESSO)	52	1.49 (1.38, 1.62)	5.61e-13	0.8708
	IVW (MR-PRESSO)		1.5 (1.44, 1.57)	1.68e-78	
	Weighted Median (MR-PRESSO)		1.5 (1.41, 1.59)	1.26e-38	
	Weighted Mode (MR-PRESSO)		1.51 (1.01, 2.27)	0.050	
**T2D**	MR Egger	313	1.04 (0.98, 1.1)	0.220	0.0005
	IVW		1.14 (1.11, 1.17)	5.98e-19	
	Weighted Median		1.12 (1.08, 1.16)	2.29e-09	
	Weighted Mode		1.07 (1.02, 1.12)	6.71e-03	

	MR Egger (MR-PRESSO)	308	1.04 (0.97, 1.11)	0.240	0.0051
	IVW (MR-PRESSO)		1.14 (1.11, 1.17)	7.05e-19	
	Weighted Median (MR-PRESSO)		1.12 (1.08, 1.16)	2.71e-10	
	Weighted Mode (MR-PRESSO)		1.12 (1.05, 1.2)	5.14e-04	
**BMI (per SD)**	MR Egger	67	1.93 (1.41, 2.64)	1.07e-04	0.5403
	IVW		1.77 (1.55, 2.01)	1.01e-17	
	Weighted Median		1.97 (1.7, 2.29)	9.18e-19	
	Weighted Mode		2.05 (1.7, 2.48)	2.71e-10	
	MR Egger (MR-PRESSO)	65	2.04 (1.56, 2.67)	2.44e-06	0.4038
	IVW (MR-PRESSO)		1.84 (1.64, 2.06)	5.77e-26	
	Weighted Median (MR-PRESSO)		1.97 (1.69, 2.3)	3.00e-18	
	Weighted Mode (MR-PRESSO)		2.04 (1.65, 2.52)	1.19e-08	
**HDL (per SD)**	MR Egger	56	1.11 (0.96, 1.28)	0.177	0.0127
	IVW		0.96 (0.87, 1.06)	0.436	
	Weighted Median		1.04 (0.96, 1.12)	0.325	
	Weighted Mode		1.06 (0.98, 1.13)	0.130	
	MR Egger (MR-PRESSO)	52	1.06 (0.95, 1.19)	0.271	0.1499
	IVW (MR-PRESSO)		1 (0.93, 1.08)	0.962	
	Weighted Median (MR-PRESSO)		1.04 (0.96, 1.12)	0.344	
	Weighted Mode (MR-PRESSO)		1.05 (0.98, 1.12)	0.178	
**LDL (per SD)**	MR Egger	25	1.16 (1.03, 1.31)	0.019	0.5975
	IVW		1.19 (1.1, 1.29)	6.81e-06	
	Weighted Median		1.22 (1.09, 1.36)	5.04e-04	
	Weighted Mode		1.01 (0.9, 1.14)	0.815	
	MR Egger (MR-PRESSO)	23	1.19 (1.02, 1.37)	0.0033	0.7801
	IVW (MR-PRESSO)		1.21 (1.11, 1.32)	2.40e-05	
	Weighted Median (MR-PRESSO)		1.25 (1.12, 1.39)	4.77e-05	
	Weighted Mode (MR-PRESSO)		1.29 (1.14, 1.45)	3.97e-04	
**TC (per SD)**	MR Egger	35	1.06 (0.75, 1.5)	0.754	0.9783
	IVW		1.06 (0.93, 1.22)	0.391	
	Weighted Median		1.04 (0.9, 1.21)	0.610	
	Weighted Mode		0.99 (0.82, 1.19)	0.920	
	MR Egger (MR-PRESSO)	34	1.08 (0.8, 1.46)	0.606	0.7032
	IVW (MR-PRESSO)		1.03 (0.91, 1.16)	0.672	
	Weighted Median (MR-PRESSO)		1.03 (0.88, 1.2)	0.685	
	Weighted Mode (MR-PRESSO)		0.99 (0.82, 1.2)	0.930	
**TG (per SD)**	MR Egger	26	1.15 (1.01, 1.31)	0.0528	0.9180
	IVW		1.15 (1.06, 1.26)	0.0016	
	Weighted Median		1.22 (1.11, 1.34)	7.02e-05	
	Weighted Mode		1.21 (1.1, 1.33)	4.96e-04	
	MR Egger (MR-PRESSO)	25	1.21 (1.07, 1.38)	6.98e-03	0.8836
	IVW (MR-PRESSO)		1.21 (1.1, 1.32)	3.05e-05	
	Weighted Median (MR-PRESSO)		1.24 (1.14, 1.36)	2.70e-06	
	Weighted Mode (MR-PRESSO)		1.24 (1.14, 1.36)	5.52e-05	
**SBP (per 10 mmHg)**	MR Egger	244	1.27 (1.08, 1.49)	3.70e-03	0.8421
	IVW		1.29 (1.21, 1.37)	3.45e-15	
	Weighted Median		1.24 (1.14, 1.35)	1.80e-07	
	Weighted Mode		1.26 (1.03, 1.53)	0.025	
	MR Egger (MR-PRESSO)	241	1.42 (1.22, 1.66)	1.36e-05	0.3000
	IVW (MR-PRESSO)		1.32 (1.24, 1.4)	1.69e-19	
	Weighted Median (MR-PRESSO)		1.27 (1.17, 1.37)	3.45e-09	
	Weighted Mode (MR-PRESSO)		1.26 (1.04, 1.51)	0.016	
**DBP (per 10 mmHg)**	MR Egger	284	1.46 (1.14, 1.89)	3.56e-03	0.9444
	IVW		1.45 (1.3, 1.62)	2.10e-11	
	Weighted Median		1.47 (1.3, 1.67)	1.93e-09	

	Weighted Mode		1.55 (1.27, 1.89)	1.75e-05	
	MR Egger (MR-PRESSO)	276	1.6 (1.28, 2.01)	5.34e-05	0.4414
	IVW (MR-PRESSO)		1.48 (1.35, 1.62)	4.62e-16	
	Weighted Median (MR-PRESSO)		1.48 (1.31, 1.67)	1.57e-10	
	Weighted Mode (MR-PRESSO)		1.55 (1.28, 1.89)	1.15e-05	
**PP (per 10 mmHg)**	MR Egger	251	0.89 (0.68, 1.16)	0.378	0.0369
	IVW		1.15 (1.03, 1.29)	0.0127	
	Weighted Median		1.04 (0.92, 1.17)	0.554	
	Weighted Mode		0.89 (0.69, 1.17)	0.409	
	MR Egger (MR-PRESSO)	244	0.94 (0.74, 1.19)	0.614	0.0637
	IVW (MR-PRESSO)		1.15 (1.05, 1.27)	4.19e-03	
	Weighted Median (MR-PRESSO)		1.04 (0.92, 1.17)	0.531	
	Weighted Mode (MR-PRESSO)		0.88 (0.68, 1.15)	0.361	

CAD, coronary artery disease; T2D, type 2 diabetes; BMI, body mass index; HDL, high-density lipoprotein cholesterol; LDL, low-density lipoprotein cholesterol; TC, total cholesterol; TG, triglycerides; SBP, systolic blood pressure; DBP, diastolic blood pressure; PP, pulse pressure; MR, Mendelian randomization; IVW, inverse-variance weighted; PRESSO, pleiotropy residual sum and outlier; OR, odds ratio; CI, confidence interval; SD, standard deviation; GIVs, genetic instrumental variables.

Table S25B. Mendelian Randomization for the Causal Effect of Risk Factors on HFpEF.

**Exposure**	**Model**	**N GIVs**	**OR (95%)**	**P**	**Pleiotropy Test P**
**CAD**	MR Egger	52	1.16 (1.06, 1.27)	2.97e-03	0.4468
	IVW		1.19 (1.14, 1.25)	4.19e-13	
	Weighted Median		1.2 (1.13, 1.27)	2.87e-09	
	Weighted Mode		1.17 (0.98, 1.38)	0.081	
	MR Egger (MR-PRESSO)	52	1.16 (1.06, 1.27)	2.97e-03	0.4468
	IVW (MR-PRESSO)		1.19 (1.14, 1.25)	4.19e-13	
	Weighted Median (MR-PRESSO)		1.2 (1.13, 1.27)	2.87e-09	
	Weighted Mode (MR-PRESSO)		1.17 (0.98, 1.38)	0.081	
**T2D**	MR Egger	313	0.97 (0.92, 1.03)	0.338	1.96e-05
	IVW		1.08 (1.05, 1.11)	6.14e-09	
	Weighted Median		1.01 (0.97, 1.04)	0.604	
	Weighted Mode		0.98 (0.94, 1.03)	0.492	
	MR Egger (MR-PRESSO)	309	0.95 (0.89, 1.02)	0.144	2.25e-05
	IVW (MR-PRESSO)		1.08 (1.05, 1.11)	1.11e-08	
	Weighted Median (MR-PRESSO)		1.02 (0.98, 1.06)	0.271	
	Weighted Mode (MR-PRESSO)		1 (0.93, 1.06)	0.910	
**BMI (per SD)**	MR Egger	67	1.73 (1.36, 2.19)	2.41e-05	0.4714
	IVW		1.87 (1.69, 2.06)	7.40e-36	
	Weighted Median		1.92 (1.66, 2.21)	1.94e-19	
	Weighted Mode		2 (1.63, 2.45)	5.76e-09	
	MR Egger (MR-PRESSO)	67	1.73 (1.36, 2.19)	2.41e-05	0.4714
	IVW (MR-PRESSO)		1.87 (1.69, 2.06)	7.40e-36	
	Weighted Median (MR-PRESSO)		1.92 (1.66, 2.21)	1.94e-19	
	Weighted Mode (MR-PRESSO)		2 (1.63, 2.45)	5.76e-09	
**HDL (per SD)**	MR Egger	56	1.11 (1, 1.23)	0.0617	0.0012
	IVW		0.97 (0.9, 1.04)	0.379	
	Weighted Median		1.03 (0.96, 1.1)	0.435	
	Weighted Mode		1.04 (0.98, 1.11)	0.221	
	MR Egger (MR-PRESSO)	54	1.08 (1, 1.17)	0.0543	0.0025

	IVW (MR-PRESSO)		0.98 (0.93, 1.04)	0.594	
	Weighted Median (MR-PRESSO)		1.03 (0.96, 1.1)	0.445	
	Weighted Mode (MR-PRESSO)		1.04 (0.97, 1.12)	0.293	
**LDL (per SD)**	MR Egger	25	1.09 (0.96, 1.23)	0.211	0.7033
	IVW		1.07 (0.98, 1.16)	0.123	
	Weighted Median		1.03 (0.94, 1.13)	0.478	
	Weighted Mode		1.04 (0.97, 1.13)	0.280	
	MR Egger (MR-PRESSO)	25	1.09 (0.96, 1.23)	0.211	0.7033
	IVW (MR-PRESSO)		1.07 (0.98, 1.16)	0.123	
	Weighted Median (MR-PRESSO)		1.03 (0.94, 1.13)	0.478	
	Weighted Mode (MR-PRESSO)		1.04 (0.97, 1.13)	0.280	
**TC (per SD)**	MR Egger	35	1.18 (0.85, 1.62)	0.328	0.1536
	IVW		0.95 (0.83, 1.08)	0.401	
	Weighted Median		0.97 (0.84, 1.12)	0.684	
	Weighted Mode		0.99 (0.83, 1.18)	0.906	
	MR Egger (MR-PRESSO)	35	1.18 (0.85, 1.62)	0.328	0.1536
	IVW (MR-PRESSO)		0.95 (0.83, 1.08)	0.401	
	Weighted Median (MR-PRESSO)		0.97 (0.84, 1.12)	0.684	
	Weighted Mode (MR-PRESSO)		0.99 (0.83, 1.18)	0.906	
**TG (per SD)**	MR Egger	26	1.12 (0.96, 1.32)	0.175	0.4405
	IVW		1.07 (0.96, 1.19)	0.223	
	Weighted Median		1.1 (1, 1.21)	0.062	
	Weighted Mode		1.09 (1.01, 1.18)	0.040	
	MR Egger (MR-PRESSO)	22	1.06 (0.94, 1.2)	0.362	0.7936
	IVW (MR-PRESSO)		1.05 (0.97, 1.14)	0.271	
	Weighted Median (MR-PRESSO)		1.09 (0.99, 1.2)	0.064	
	Weighted Mode (MR-PRESSO)		1.06 (0.97, 1.16)	0.188	
**SBP (per 10 mmHg)**	MR Egger	244	1.17 (1.01, 1.36)	0.0429	0.5269
	IVW		1.22 (1.15, 1.3)	3.95e-11	
	Weighted Median		1.22 (1.12, 1.32)	1.37e-06	
	Weighted Mode		1.03 (0.87, 1.24)	0.707	
	MR Egger (MR-PRESSO)	244	1.17 (1.01, 1.36)	0.0429	0.5269
	IVW (MR-PRESSO)		1.22 (1.15, 1.3)	3.95e-11	
	Weighted Median (MR-PRESSO)		1.22 (1.12, 1.32)	1.37e-06	
	Weighted Mode (MR-PRESSO)		1.03 (0.87, 1.24)	0.707	
**DBP (per 10 mmHg)**	MR Egger	284	1.31 (1.07, 1.61)	9.54e-03	0.1579
	IVW		1.15 (1.05, 1.25)	1.97e-03	
	Weighted Median		1.2 (1.07, 1.35)	2.31e-03	
	Weighted Mode		1.43 (1.17, 1.75)	4.70e-04	
	MR Egger (MR-PRESSO)	281	1.37 (1.13, 1.66)	1.69e-03	0.0732
	IVW (MR-PRESSO)		1.17 (1.07, 1.27)	3.40e-04	
	Weighted Median (MR-PRESSO)		1.21 (1.08, 1.35)	7.70e-04	
	Weighted Mode (MR-PRESSO)		1.43 (1.17, 1.75)	5.92e-04	
**PP (per 10 mmHg)**	MR Egger	251	1.27 (1.05, 1.53)	0.0141	0.7629
	IVW		1.3 (1.21, 1.41)	2.07e-11	
	Weighted Median		1.31 (1.18, 1.46)	8.13e-07	
	Weighted Mode		1.43 (1.12, 1.83)	4.47e-03	
	MR Egger (MR-PRESSO)	251	1.27 (1.05, 1.53)	0.0141	0.7629
	IVW (MR-PRESSO)		1.3 (1.21, 1.41)	2.07e-11	
	Weighted Median (MR-PRESSO)		1.31 (1.18, 1.46)	8.13e-07	
	Weighted Mode (MR-PRESSO)		1.43 (1.12, 1.83)	4.47e-03	

CAD, coronary artery disease; T2D, type 2 diabetes; BMI, body mass index; HDL, high-density lipoprotein cholesterol; LDL, low-density lipoprotein cholesterol; TC, total cholesterol; TG, triglycerides; SBP, systolic blood pressure; DBP, diastolic blood pressure; PP, pulse pressure; MR, Mendelian

randomization; IVW, inverse-variance weighted; PRESSO, pleiotropy residual sum and outlier; OR, odds ratio; CI, confidence interval; SD, standard deviation; GIVs, genetic instrumental variables.

4. Thank you for the additional plot to compare allele frequency. Is it possible to include some additional gene labels in panel B, to make more direct comparisons between the points across the two panels?

In the text you've added, can you please make it clear that you're talking about the allele frequency of the leadSNP at each locus, as loci themselves do not have allele frequencies.

Otherwise, the authors have addressed the concerns very well.

We have revised Figure 2 Panel B to include more gene labels. This is the updated figure in the revised manuscript, as shown below. We also changed the wording "loci" to lead SNP to avoid confusion:

Results: "Of the 7 lead SNPs in the HFrEF GWS loci in the African ancestry, 2 SNPs showed small or null effects in the European ancestry, and 4 SNPs (*CD36, SPI1, TRIM48,* and *SPNS3*) were rare in the European ancestry, with MAF ≤ 0.05%, Table 2 & Figures 2-3. The two lead SNPs in other loci, *CSK* (effect allele frequency 0.34 in Europeans vs. 0.89 in Africans) and *PSRC1* (0.77 in Europeans vs. 0.27 in Africans), demonstrated allele frequency differences of ≥ 50% between European and African populations."

Revised Figure 2. Scatter Plot for Comparison of the Estimates for the Genome-wide Significant Loci for the Meta-analysis of GWAS on HFrEF Among European Ancestry and African Ancestry. A: Comparison of Effect Sizes. Pearson Correlation Coefficient = 0.27 (95% CI -0.09, 0.57). Loci with Heterogeneity are Labeled. B: Comparison of Risk Allele Frequency.

A.

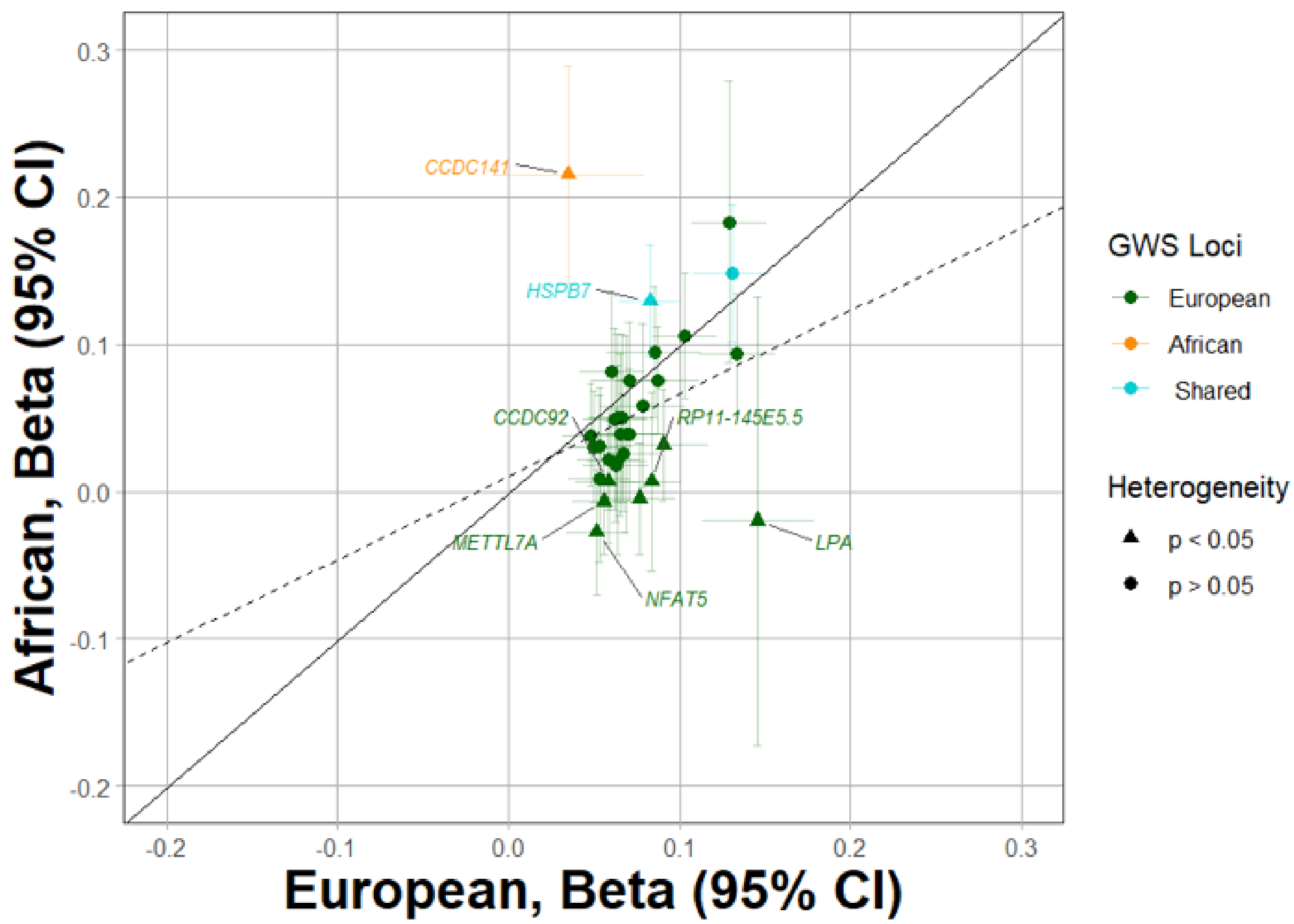

African, Beta (95% CI)
European, Beta (95% CI)
CCDC141
HSPB7
CCDC92
RP11-145E5.5
METTL7A
NFAT5
LPA
GWS Loci
European
African
Shared
Heterogeneity
p < 0.05
p > 0.05

B.

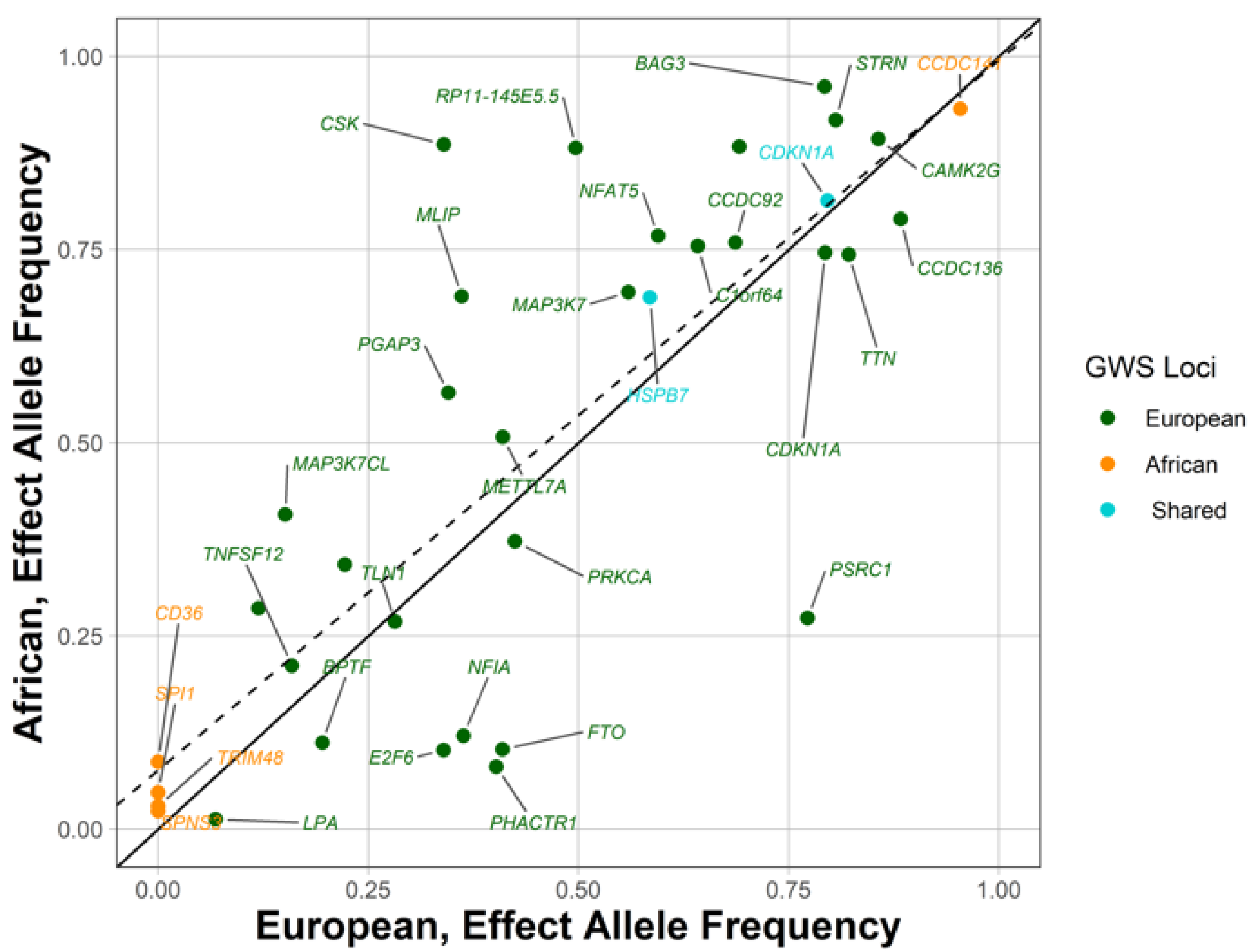

African, Effect Allele Frequency
European, Effect Allele Frequency
BAG3
STRN
CCDC141
RP11-145E5.5
CSK
CDKN1A
CAMK2G
NFAT5
CCDC92
MLIP
CCDC136
MAP3K7
C1orf64
TTN
PGAP3
HSPB7
CDKN1A
MAP3K7CL
METTL7A
TNFSF12
TLN1
PRKCA
PSRC1
CD36
BPTF
NFIA
SPI1
FTO
TRIM48
E2F6
SPNS3
LPA
PHACTR1
GWS Loci
European
African
Shared

9. Thank for your pursuing external validation. In the text, could you also include the proportion of loci you're classifying as novel which replicate in Biobank Japan?

We have added this information in the revised statements below:

Results: "Out of the 46 GWS loci identified in the multi-ancestry meta-analysis of HFrEF, 34 loci were available in Biobank Japan; among these, 21 (62%) loci replicated at $P < 0.05$ with the same direction of genetic effect. For HFpEF, 1 of the 3 GWS loci was available in Biobank Japan and replicated at $P < 0.05$ with concordant effect direction. For all-cause HF, out of the 136 GWS loci, 106 loci were available in Biobank Japan, of which 46 (43%) replicated at $P < 0.05$ with concordant effect direction, Tables 1 and S2-S3."

10. The addition to the text could include the proportion of male veterans. Please be consistent with either men/women (gender) or male/female (sex) in these sentences.

Thank you for this suggestion. We have added the proportion of male veterans to the revised manuscript and have standardized the terminology to use male/female throughout the manuscript.

Discussion: "The MVP cohort was predominantly composed of male veterans, who accounted for 92% of participants."

Reviewer #3

Major comments

8. Suggest a re-write for consistent use of sex/gender terminology:

…predominantly composed of male veterans which limits our power for sex-stratified analyses, particularly among female veterans, and may constrain the generalizability of our findings to females. Larger and more ….including those with a higher proportion of female participants,….

In this section, it would be good to be specific that the external replication in BiobankJapan was just for all-cause HF

Thank you for this suggestion. We have standardized the terminology to use male/female throughout the manuscript.

The external validation was performed in Biobank Japan for all-cause HF, HFrEF and HFpEF. We have clarified in the Methods and Results:

Methods: "External validation was performed using the independent Japanese GWAS of HF from BioBank Japan, which includes 16,251 all-cause HF cases and 197,577 controls (N = 213,828), 4,254 HFrEF cases and 197,577 controls (N = 201,831), and 7,154 HFpEF cases and 197,577 controls (N = 204,731), as reported by Enzan et al. (PMID: 41184235)."

Results: "Out of the 46 GWS loci identified in the multi-ancestry meta-analysis of HFrEF, 34 loci were available in Biobank Japan; among these, 21 (62%) loci replicated at $P < 0.05$ with the same direction of genetic effect. For HFpEF, 1 of the 3 GWS loci was available in Biobank Japan and replicated at $P < 0.05$ with concordant effect direction. For all-cause HF, out of the 136 GWS loci, 106 loci were available in Biobank Japan, of which 46 (43%) replicated at $P < 0.05$ with concordant effect direction, Tables 1 and S2-S3."

Minor comments

1. I would further clarify it is the SNP you're defining as the lead SNP which has the low risk-allele frequency.

We thank the reviewer for this helpful suggestion. We have revised the sentence for clarity and accuracy, while following the 150-word limit in the abstract:

"Four HFrEF loci were detected in African ancestry participants near *CD36, SPI1, TRIM48,* and *SPNS3*, with lead SNPs showing low risk-allele frequencies in European populations."

5. The last sentence should be 102 GWAS loci and 3 GWS loci for consistency with the previous and readability.

We have updated this writing as:

"Additionally, the all-cause HF GWAS meta-analyses revealed 102 GWS loci for European ancestry and 3 GWS loci for African ancestry."

10. When you list the rsIDs, perhaps add the gene name you're using for that locus

We have updated the writing accordingly:

Discussion: "For example, rs600038 (*9q34.2*) and rs653178 (*ATXN2*) were each associated with the levels of more than 170 protein targets, **Tables S17A-S17C**."

Reviewer #2 (Remarks to the Author):

Thank you for addressing my comments.

Reviewer #3 (Remarks to the Author):

For the MR results with HFrEF, Type 2 Diabetes and pulse pressure have persistently significant pleiotropy test and MR Egger association is non-significant after outlier removal. Therefore, I think the results sentence "Type 2 diabetes, triglycerides, and pulse pressure demonstrated potential causal effects for HFrEF. " is overstating the evidence.

We have revised the text to describe these results as suggestive rather than causal and have emphasized that they should be interpreted cautiously in light of the sensitivity analyses:

"Genetically predicted type 2 diabetes, triglycerides, and pulse pressure showed suggestive associations with HFrEF, but these findings were less consistent across sensitivity analyses and should be interpreted cautiously."

Finally, language in the results section around causality should use more careful wording such as "Genetically predicted X was associated with Y" or "MR provides evidence supporting a causal role of X on Y."

We have revised the paragraph as below:

"Genetically predicted coronary artery disease, body mass index, low-density lipoprotein cholesterol, systolic blood pressure, and diastolic blood pressure were associated with HFrEF, providing MR evidence supporting their causal roles in HFrEF. In contrast, MR analyses did not provide evidence supporting causal roles of high-density lipoprotein cholesterol or total cholesterol in HFrEF. Genetically predicted type 2 diabetes, triglycerides, and pulse pressure showed suggestive associations with HFrEF, but these findings were less consistent across sensitivity analyses and should be interpreted cautiously. For HFpEF, genetically predicted coronary artery disease, body mass index, systolic blood pressure, diastolic blood pressure, and pulse pressure were associated with HFpEF, providing MR evidence supporting their causal roles. MR analyses did not provide evidence supporting causal roles of lipid traits in HFpEF, Supplementary Data 25A-25B."

Table S25A and B should make clear that the pleiotropy test p-value is from MR Egger intercept test. Is an MR-PRESSO global test p-value available for the analyses with MR-PRESSO?

In Supplementary Data 25A-25B, we have added footnote to explain that "Pleiotropy test p values from MR Egger intercept test". We also added additional column for the MR-PRESSO global test.

In copyediting, fixed-effects should be changed to fixed-effect (I made this mistake in my review, sorry!)

We have changed "fixed-effects" to "fixed-effect" accordingly. Thank you!

Otherwise, the authors have addressed my previous comments in a satisfactory manner. Thank you!
